# Global, Regional, and National Burden of Endometrial Cancer, 1990–2017: Results From the Global Burden of Disease Study, 2017

**DOI:** 10.3389/fonc.2019.01440

**Published:** 2019-12-19

**Authors:** Shuang Zhang, Ting-Ting Gong, Fang-Hua Liu, Yu-Ting Jiang, Hui Sun, Xiao-Xin Ma, Yu-Hong Zhao, Qi-Jun Wu

**Affiliations:** ^1^Department of Clinical Epidemiology, Shengjing Hospital of China Medical University, Shenyang, China; ^2^Clinical Research Center, Shengjing Hospital of China Medical University, Shenyang, China; ^3^Department of Obstetrics and Gynecology, Shengjing Hospital of China Medical University, Shenyang, China

**Keywords:** disability-adjusted life years, disease burden, endometrial cancer, incidence, mortality, prevalence

## Abstract

Endometrial cancer (EC) is the most common malignancy affecting women in developed countries. Recently, the EC disease burden has changed; therefore, the Global Burden of Disease (GBD) 2017 was used to comprehensively analyze the global, regional, and national burden of EC between 1990 and 2017. General GBD cancer estimation methods were used with the data input from vital registration systems and cancer registries. Annual percent changes were calculated to quantify the trends of EC burden estimates during the study period. Furthermore, the sociodemographic index (SDI) was used to assess the relationship between the EC burden estimates and development level. From 1990 to 2017, the age-standardized incidence and prevalence rate of EC increased globally by 0.58 and 0.89% per year, respectively. In contrast, the age-standardized death rate and disability-adjusted-life years (DALYs) decreased by 1.19 and 1.21% per year, respectively. Increasing trends in both the incidence and prevalence were observed in all SDI quintiles, except for the low SDI quintiles, whereas decreasing trends were observed in all SDI quintiles for mortality and DALYs. Additionally, a non-linear association existed for the level of mortality rate, DALYs, and SDI. Of note, there was a strong positive association between a high body mass index and DALYs across all SDI quintiles. In conclusion, EC incidence and prevalence rates are growing globally, whereas the death rate and DALYs decreased between 1990 and 2017. Greater efforts, particularly detailed prevention strategies for reducing obesity, should be performed to reverse this phenomenon.

## Introduction

Endometrial cancer (EC) is an increasingly problematic gynecological cancer (1). According to the GLOBOCAN cancer statistics, there are an estimated 382,069 new cases and 89,929 deaths attributed to EC worldwide in 2018 (2). Moreover, EC was the second most common and the fourth leading cause of death due to gynecological cancer among women worldwide in 2018 (2). According to the International Agency for Research on Cancer, the incidence rate of EC is increasing rapidly compared with 2018, and is estimated to increase by more than 50% worldwide by 2040^1^. However, the incidence and mortality of EC differ throughout the world. The incidence rates of EC are generally higher in high-income countries compared to low and middle income countries (3, 4). For example, the highest incidence rates of EC are observed in North America as well as Northern and Western Europe, whereas the rates are the lowest in South-Central Asia and most of Africa (5). Additionally, the incidence rates have been reported to have an increasing trend in the US and several European countries since around 2000 (6, 7). Conversely, the mortality caused by EC had been found to be the highest among women of low socioeconomic status (8). Indeed, the highest mortality rate was observed in Melanesia (3, 5), but was the lowest in Northern Africa (5). Although mortality rates are increasing in line with incidence (7, 9), decreased mortality is observed in all sociodemographic index (SDI) quintiles, a composite indicator of development status. Thus, data concerning the EC burden estimates and trends in different regions and countries may be valuable for decision-making and resource allocation. However, previous studies to date have mainly focused on single countries or regions, EC trends over a limited period, and there has been only one study published on the international patterns and trends of EC incidence (10, 11). Therefore, in this study, we report the results of the Global Burden of Disease (GBD) 2017 study on primary EC incidence, prevalence, mortality, disability-adjusted life years (DALYs), years of life lived with disability (YLDs), and years of life lost (YLLs) for 195 countries or territories from 1990 to 2017 and assess the association between these indicators of the disease burden and the SDI level.

## Materials and Methods

Detailed methods for every analytic step in GBD 2017 are described elsewhere and are consistent with the Guidelines for Accurate and Transparent Health Estimates Reporting (Supplemental Table 1) (12–14). Herein, we concentrate on methods and statistical analyses to estimate the burden of EC. A description of the estimation process is provided in the supplementary methods (Supplemental File 1). Similar to previous GBD studies, the entire time series was re-estimated, and the results provided in this study replace previous GBD studies.

### Estimation Framework

In the GBD 2017, the estimation process begins with EC mortality. We used a vital registration system and cancer registry incidence data, which were converted to mortality estimates through a separate modeling of the mortality-to-incidence ratios (MIR) (Supplemental Table 2) (15). The data were processed to adjust for aggregated causes, age groups, or uninformative causes of death (16). To estimate the number of deaths attributed to EC, the mortality data were input into the Cause of Death Ensemble Model (CODEm) and analyzed (17). The CODEm predicts mortality based on the available data and covariates, such as education, cumulative cigarettes smoking, SDI, lagged distributive income, and alcohol consumption. Using codcorrect to adjust the CODEm results, an algorithm that scales the estimates to all-cause mortality estimates using the uncertainty distribution around the cause fraction estimates for each GBD cause of death for every country, year, and age. Then, these death estimates were used to calculate the YLL. The epidemiological results of EC was modeled with DisMod-MR 2.1, a Bayesian meta-regression framework that has been widely applied in GBD epidemiological modeling. EC incidence was estimated by dividing mortality by MIR. MIR were also used to calculate EC survival. The incidence and survival estimates were used to calculate the prevalence, which was divided into four stages, reflecting changes in disability during the: (1) diagnosis and treatment; (2) remission; (3) dissemination; and (4) terminal phases. YLDs were generated by multiplying the prevalence of each stage by the distinct disability weights. The sum of the YLLs and YLDs represents the DALYs (18). All International Classification of Diseases 9th Edition and International Classification of Diseases 10th Edition codes pertaining to primary EC were included in these estimates.

Annual GBD data for EC by region and country from 1990 to 2017 were collected from the Global Health Data Exchange query tool (19). There was available data for a total of 195 countries and regions. Considering the relationship between development status and disease burden, the SDI for each country was calculated in the GBD 2017 and countries were categorized into SDI quintiles. This new indicator is the geometric mean of the total fertility rate, income per capita, and mean education years for those older than 15 years, scaled from 0 to 1 (Supplemental Table 3) (16, 20). In addition, the 195 countries and regions were divided into 21 regions based on epidemiological similarity and geographical proximity (21).

### Estimation of EC Risk Factor Exposure

A comparative risk assessment approach was used to estimate the proportion of EC that could be attributable to different risk factors. The detailed methods of evaluating exposure levels and the associated burden of disease have been reported elsewhere (22). Risk factors were divided into three main categories: (1) behavioral; (2) environmental/occupational; and (3) metabolic. For each risk factor, the observed deaths were compared to those that would have been observed if a counterfactual level of exposure had occurred in the past to estimate the attributable burden. Theoretically, the minimum risk exposure level represents the risk exposure level that minimizes the disease risk at the demographic level, which was used to calculate the attributable burden of disease. Risk factors, their definitions, and the theoretical minimum risk exposure level are provided in the Appendix (Supplemental Figures 4, 5).

### Statistical Analysis

We used the age-standardized incidence rate (ASIR), age-standardized prevalence rate (ASPR), age-standardized death rate (ASDR), DALYs, percent change, and evaluated annual percentage change (APC) to quantify the trends in the EC global burden (23). The age range was restricted to between 25 and 74 years and divided into ten 5 year age groups. All measures were age-standardized using the GBD standard population. APC is a widely used measure of the annual ASR trend, and the regression line matches the natural logarithm of the rates. APC and 95% confidential interval (CI) values can also be obtained from the linear regression model (24). We used a generalized additive model with a Loess smoother on SDI to evaluate the associations between SDI and disease burden indicator using GBD estimates from all national locations for each year from 1990 to 2017 (25). Uncertainty intervals (UI) were calculated by taking the 2.5 and 97.5 centile values of 1,000 draws of the posterior distribution of that measure. All statistics were performed using SPSS (Version 23, SPSS Inc.) and R software (Version 3.4.4, R core team). A 95% CI, excluding 0, was considered to be statistically significant.

## Results

### EC Incidence and Prevalence

**Figure 1:**
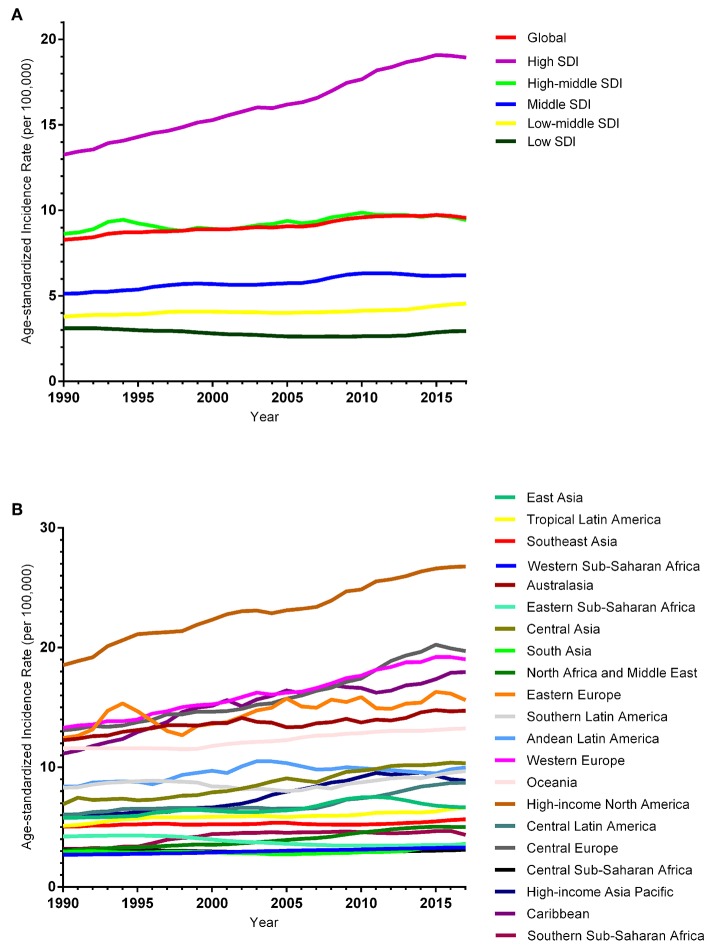
Trends in the global disease burden of endometrial cancer incidence from 1990 to 2017. **(A)** Trends in the global disease burden of endometrial cancer incidence by sociodemographic index from 1990 to 2017. **(B)** Trends in the global disease burden of endometrial cancer incidence by region from 1990 to 2017.

**Figure 2:**
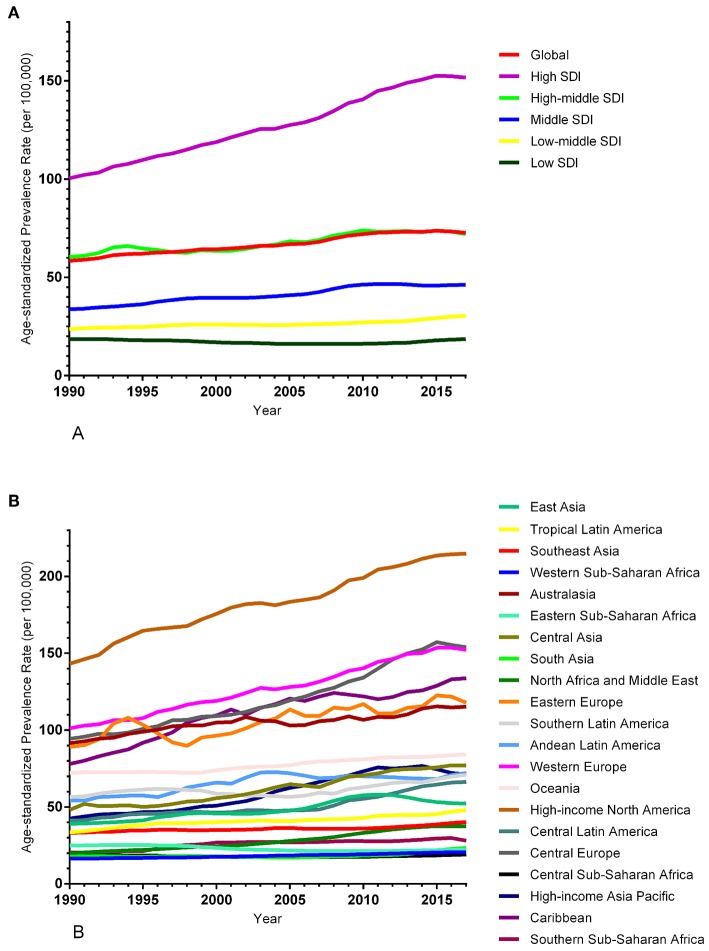
Trends in the global disease burden of endometrial cancer prevalence from 1990 to 2017. **(A)** Trends in the global disease burden of endometrial cancer prevalence by sociodemographic index from 1990 to 2017. **(B)** Trends in the global disease burden of endometrial cancer prevalence by region from 1990 to 2017.

**Figure 3:**
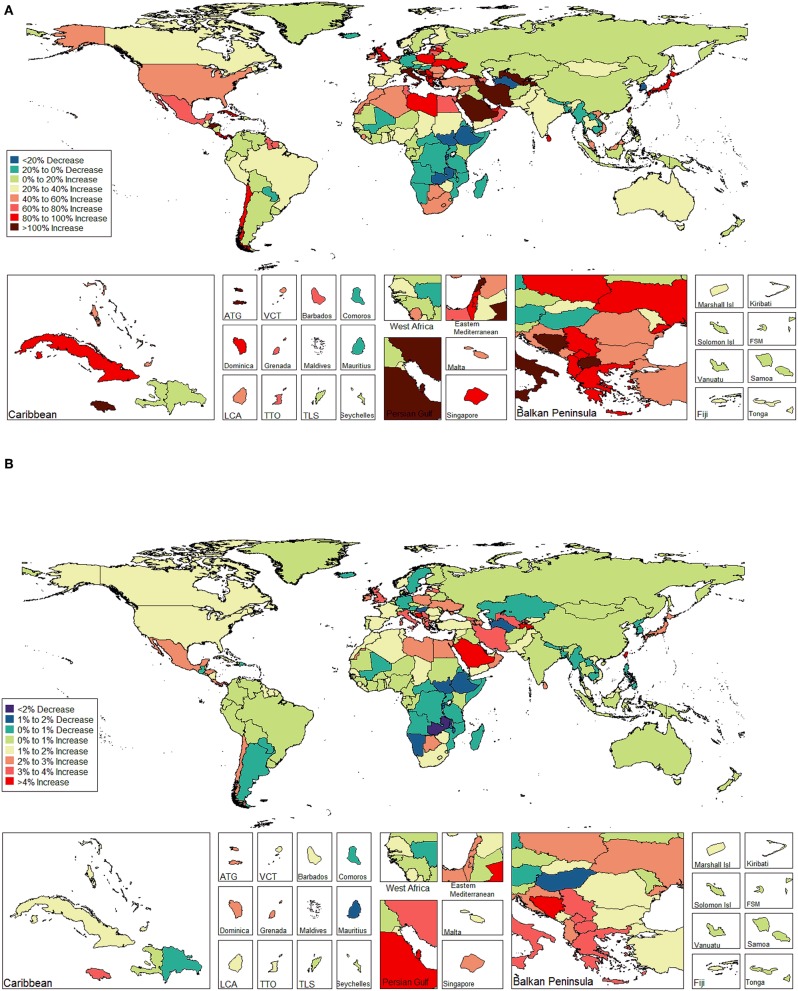
The global disease burden of endometrial cancer incidence in 195 countries and territories. **(A)** The percent change in the age-standardized incidence rate of endometrial cancer between 1990 and 2017. **(B)** The estimated annual percentage change of endometrial cancer age-standardized incidence rate from 1990 to 2017.

**Figure 4:**
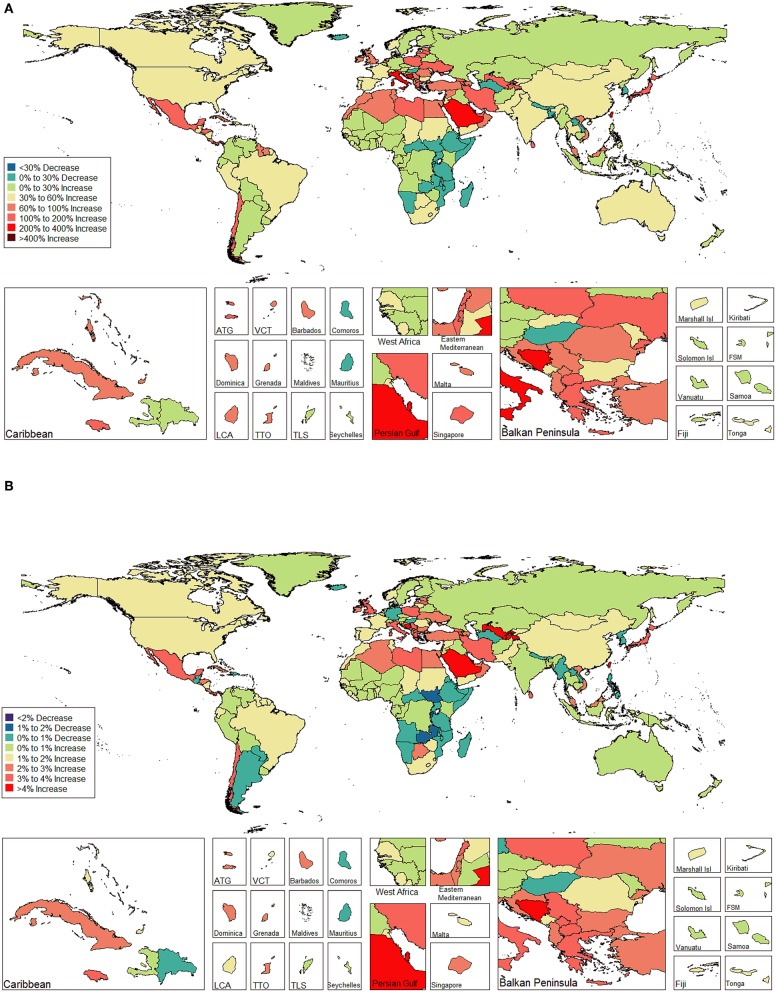
The global disease burden of endometrial cancer prevalence in 195 countries and territories. **(A)** The percent change in the age-standardized prevalence rate of endometrial cancer between 1990 and 2017. **(B)** The estimated annual percentage change of endometrial cancer age-standardized prevalence rate from 1990 to 2017.

The ASIR of EC changed from 8.28 per 100,000 women (95% UI: 8.03, 8.51) in 1990 to 9.57 per 100,000 women (95% UI: 9.33, 9.83) in 2017, with a substantial increase of 0.58% (95% CI: 0.52, 0.64%) per year (Supplemental Table 10). In addition, the ASPR changed from 58.43 per 100,000 (95% UI: 56.66, 59.98) in 1999 to 72.66 per 100,000 women (95% UI: 70.82, 74.71) in 2017, with a substantial increase of 0.89% (95% CI: 0.82, 0.96%) per year (Supplemental Table 11). For both of these two burden estimates, increasing trends were observed in all but the low SDI quintiles. Of note, the most significant increasing trend was consistently observed in the high SDI quintiles (Figures 1A, 2A and Supplemental Tables 12, 13). Of the 21 analyzed regions, increasing trends were observed in 19 regions for ASIR, with the highest level observed in high-income Asia Pacific (10.40%). In contrast, a decreasing trend was observed only in Central Sub-Saharan Africa (7.45%) and Eastern Sub-Saharan Africa (92.55%) (Figure 1B and Supplemental Table 12). For ASPR, increasing trends were observed in all regions except for Eastern Sub-Saharan Africa, with the highest in North Africa and Middle East (10.35%) (Figure 2B and Supplemental Table 13). From 1990 to 2017, there were 153 countries/territories (78.46%) and 162 countries/territories (83.08%) that showed increasing trends for ASIR and ASPR, respectively across 195 countries/territories (Figures 3, 4 and Supplemental Tables 14, 15). Taiwan (province of China) had the largest increasing trend with 6.08% (95% CI: 5.51, 6.65%) per year for ASIR and 6.53% (95% CI: 5.95, 7.11%) per year for ASPR. Whereas, Zambia had the largest decreasing trend with −2.10% (95% CI: −2.62, −1.58%) per year for ASIR and −1.73% (95% CI: −2.26, −1.20%) per year for ASPR (Supplemental Tables 14, 15). Globally, in the age stratified analysis for incidence and prevalence, the distance between the two lines has expanded among ages 45–49, 50–54, and 55–59. The maximum interval was observed between ages 45–49 and 50–54 (Supplemental Figures 6, 7).

### EC Mortality

EC was the cause of 85 239 deaths (95%UI: 83,186, 87,446) globally in 2017, which was approximately a 45.39% increase compared to 1990. The ASDR decreased from 2.69 per 100,000 women (95% UI: 2.58, 2.79) in 1990 to 2.00 per 100,000 women (95% UI: 1.95, 2.05) in 2017, representing a decrease of 1.19% (95% CI: −1.24, −1.14%) per year (Supplemental Table 16). In contrast to the incidence and prevalence, decreasing trends were observed in all SDI quintiles for ASDR, and importantly the greatest decline was recorded in the high-middle SDI quintiles with −1.89% (95% CI: −2.04, −1.74%) per year contributing 32.34% to the decreasing trend (Supplemental Tables 16, 17 and Supplemental Figure 8). Moreover, decreasing trends were observed in 15 out of the 21 regions (Supplemental Tables 16, 17 and Supplemental Figure 8). The largest decreasing trend was detected in Eastern Asia [2.62% (95% CI: −2.84, −2.41%) per year], which contributed to 16.14% of the total decreasing trend. Conversely, the greatest increase was observed in the Southern Sub-Saharan Africa region [0.83% (95% CI: 0.32, 1.34%) per year], which contributed to 55.43% of the overall increasing trend (Supplemental Tables 16, 17). In contrast to the incidence and prevalence, over half of the countries/territories (61.54%) displayed a decreasing trend across 195 countries/territories from 1990 to 2017 (Supplemental Table 18 and Supplemental Figure 9). Although South Korea had the largest decreasing trend [−5.07% (95% CI: −5.81, −4.32%) per year], Georgia had the greatest increasing trend [3.01% (95% CI: 2.01, 4.02%) per year] (Supplemental Table 18). Globally, when stratified by age group, the distance between the two lines has expanded among ages 55–59, 60–64, 65–69, and 70–74. The maximum interval was observed between the ages 55–59 and 60–64 (Supplemental Figure 10).

### EC DALYs

EC was responsible for 2140,925 DALYs (95% UI: 2056,461, 2226,155) in 2017, of which 210,919 (95% UI: 151,498, 279,691) were from YLDs (9.85%) and 1 930 006 (95% UI: 1879,870, 1983,033) were from YLLs (90.15%) (Supplemental Tables 19–27 and Supplemental Figures 11–19). Global age-standardized DALYs of EC decreased by 25.63% from 67.88 per 100,000 women in 1990 (95% UI: 64.30, 71.04) to 50.48 per 100,000 women in 2017 (95% UI: 48.48, 52.49), at a rate of −1.21% (95% CI: −1.26, −1.15%) per year (Supplemental Table 19). Decreasing trends were observed in all SDI quintiles, with the most significant decreasing trend in the high-middle SDI quintiles (Supplemental Tables 19, 20 and Supplementary Figure 11). Across the 21 regions that were analyzed, decreasing trends were observed in 16 regions and all displayed statistical significance, except for the Caribbean. Eastern Asia had the largest decreasing (17.09%) trend whereas Southern Sub-Saharan Africa had the greatest increasing (34.33%) trend (Supplemental Tables 19, 20 and Supplemental Figure 11). Among the 195 countries/territories, 119 reported a decreasing trend between 1990 and 2017 (Supplemental Table 21 and Supplemental Figure 12). South Korea had the largest decreasing trend [−5.05% (95% CI: −0.21, 0.02%) per year], whereas Taiwan [3.15% (95% CI: −0.49, 0.03%) per year] had the greatest increasing trend (Supplemental Table 21). Globally, in the age-stratified analysis, the distance between the two lines has expanded among ages 45–49, 50–54, 55–59, and 60–64. Similar to mortality, the maximum interval was observed between ages 55–59 and 60–64 (Supplemental Figure 13).

### Estimates of the Global EC Burden in Relation to SDI Levels

**Figure 5:**
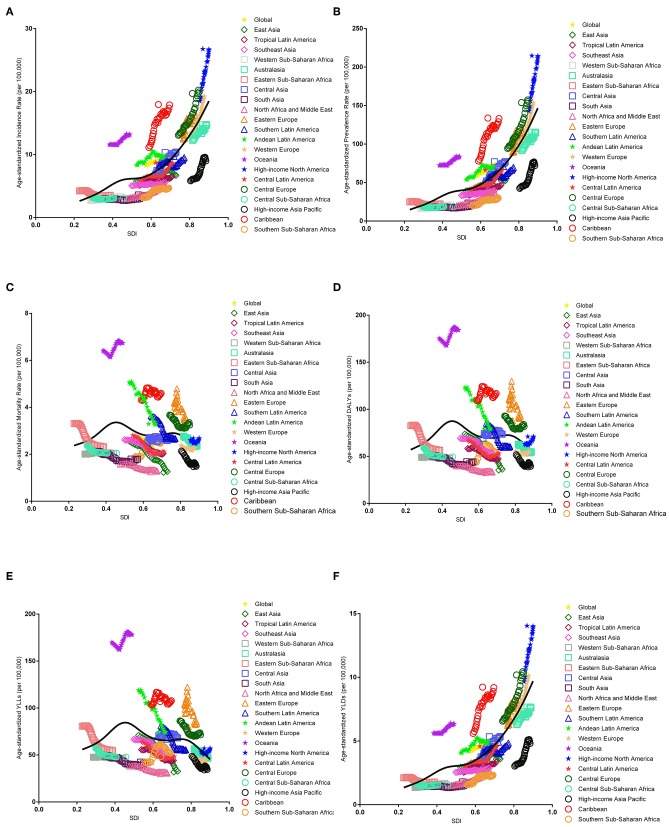
Co-evolution of the global age-standardized burden estimates with SDI and for GBD regions for endometrial cancer, 1990–2017. [**(A)** Incidence, **(B)** prevalence, **(C)** mortality, **(D)** DALYs, **(E)** YLLs, and **(F)** YLDs. Colored lines show the global and regional values for age-standardized burden estimate rates. Each point on the line represents 1 year starting at 1990 and ending at 2017. The black line represents the average expected relationship between SDI and burden estimates rates for endometrial cancer based on values from each geographical region over the 1990–2017 estimation period. DALYs, disability-adjusted life-years; SDI, Socio-demographic Index; YLLs, years of life lost; YLDs, years lived with disability].

The association between the estimates of the global EC burden and SDI levels for each of the 21 GBD regions for each individual year between 1990 and 2017 are illustrated in Figure 5. Overall, there was a linear association among the EC incidence, prevalence, and SDI level. Positive associations were observed with an increasing SDI in almost all regions; however, no significant positive association was observed in Eastern Sub-Saharan Africa, Western Sub-Saharan Africa, and South Asia. In addition, a potential non-linear association existed among mortality, DALYs, and SDI level. An inverted U-curve was observed when the SDI was limited to 0.3–0.6. Moreover, the highest values were reached when the SDI was equal to 0.45, which may be attributed to Oceania.

### Risk Factors for EC

We found that a high body mass index (BMI) was the only risk factor for EC based on the GBD 2017 from 1990 to 2017. There was a strong positive association between a high BMI and DALYs across all SDI quintiles (Supplemental Figure 20).

## Discussion

Based on the GBD 2017, this study presents the most recent trends and patterns of the worldwide burden estimates of EC from 1990 to 2017 at the global, regional, and national levels. During the entire study period, increasing trends were observed in all SDI quintiles for incidence and prevalence rate of EC, except for the low SDI quintiles. However, decreasing trends were observed in all SDI quintiles for mortality and DALYs. Additionally, a potential non-linear association was evident between mortality and the DALYs and SDI. Across the 195 countries/territories, over two-thirds of the countries/territories displayed an increasing trend of incidence and prevalence, whereas over half of these countries/territories exhibited a decreasing trend for mortality and DALYs.

Our results are consistent with several previous evaluations of regional trends of EC burden estimates. The incidence rates of EC have been increasing since ~2000 in several developed countries (6, 7). The increasing trend in incidence was observed in Singapore, Thailand, Japan, India, Belarus, Lithuania, Costa Rica, US, Spain, and New Zealand. Similarly, for mortality, we observed a similar decreasing trend in Japan and Singapore (26, 27), as well as in some historically lower-risk regions (e.g., Asia) (27). Previous studies (e.g., the GLOBOCAN 2012 from the International Agency for Research on Cancer, the report from Five Continents database and the Surveillance, Epidemiology and End Results Program) (3, 28) have indicated that the EC incidence rates were higher in Northern America and Central-Eastern Europe, compared to South-Central Asia, where the incidence rates were lower. Furthermore, Lortet-Tieulent et al. (11) showed that the highest incidence rates were observed in Europe and North America, whereas the lowest rates were found in developing countries, including South Africa and India, from 2006 to 2007. In addition, there was a decreasing trend for EC mortality in European countries and Asian regions, except for Central Asia, and higher mortality was observed in Eastern Europe and the Caribbean. Similar patterns were observed for EC mortality when compared with the data from several previous studies (3, 27, 29).

Differences in the EC burden estimates might be related with the socioeconomic level, and endogenous estrogen exposure (e.g., nulliparity, fewer pregnancies, early age at menarche, and obesity) (30). The incidence of EC increased with time and in successive generations in several countries, especially in countries with rapid socioeconomic transitions (11). Between-country variations in various factors (e.g., socioeconomic status [SES]) could further lead to heterogeneity in these burden estimates. Moreover, a lower educational level was significantly associated with a higher proportion of late stage EC among patients who were between 50 and 74 years old at the time of diagnosis (31). Furthermore, women with higher SES levels may have less unhealthy living behaviors. Studies have indicated that the consumption of meat, fat, and fried foods used to be higher whereas lower fiber, whole grains, fruit, nuts, and seeds among the richest (32–35), which may also increase the risk of EC (36–39). Previous studies have found that educated women typically use estrogen replacement, contraception, and tend to have lower parity, compared with uneducated women (40). This may increase the risk of developing EC among educated women. According to the World Fertility Report 2013, the fertility rates declined from more than 6.0 to 1.6 or fewer children per women in Singapore and Thailand, and from 3.0 to 1.3 in Japan between 2000 and 2005. Moreover, nulliparity has more than doubled since 1994 in Japan and Spain (41). A particularly high rate of HRT use (37%) in the 60–64 year age group has been reported in the US population residing in Stanford, California (42). An early age at menarche, late age at menopause, and late age at last birth are associated with an increased risk of EC in developed countries (43, 44).

Although BMI is a rough estimate of obesity, studies have indicated that an obese woman has a four-fold increased risk of developing EC compared with woman with a normal BMI (45), and EC survivors with a BMI ≥ 30 had a 2.28 relative risk of mortality compared to women with a BMI < 22.5 (46). Several reports have also indicated that the proportion of women with a high BMI increased more rapidly compared to the global average in high-income countries (except for Japan) (11). For instance, the proportion of women with a high BMI increased from 44 to 57% in the United Kingdom, 60% in New Zealand, and 62% in the United States (11). Compared with BMI, which is a rough estimate of obesity, obesity should be instead be measured using dual-energy X-ray absorptiometry. Therefore, whether such recommendations to reverse the phenomena of EC through BMI at the global level could be achieved, should be further investigated in the future.

Due to symptomatic bleeding, EC can be diagnosed at an early stage and has a generally favorable prognosis (47). In contrast to the gradually increasing incidence rates, EC mortality rates have steadily decreased, likely due to improved cancer treatment and prognosis (48). Although the cornerstone of treatment in early stage EC is surgery, there is now movement toward the use of minimally invasive techniques (e.g., laparoscopic approach or robot-assisted laparoscopic surgery) (49). Most patients with stage I and II EC will have a favorable prognosis, and the 5 year survival rate is approximately 80%. Even patients with stage III or IV EC will have a worse survival, and the 5 year survival rate is ~40% (50). Furthermore, several studies have indicated that the association between EC mortality with driving time to the nearest cancer treatment center disappeared by 1990 (51, 52). However, we observed increased mortality for the southern sub-Saharan Africa region, which may be attributed to the lack of a diagnostic approach to detect and register cases, the lack of skilled professionals in oncology to make a confirmed diagnosis.

As far as we know, the current study is the first to comprehensively analyze the burden of EC from 1990 to 2017 at the global level and further estimate the co-evolution of the global EC burden estimates and SDI to explore the relationship between EC development and social status. Additionally, the GBD study provides high quality burden estimates associated with EC. More importantly, the GBD study is now undergoing annual updates, which allows for methodological improvements with every iteration, as well as the inclusion of the most recent data sources. However, several limitations continue to exist: (1) some fluctuations in the burden estimates of EC may reflect deviations in the detection associated with modifications in screening schemes other than practical changes in the age-specific rates; (2) In many low income regions, vital registration and cancer registry data are less reliable (53, 54). Due to underreporting and the failure of diagnosis, EC estimates might suffer from underestimation. Therefore, this could be a source of bias during cancer registration in these countries (55); (3) mapping of distinct disease-coding systems into the GBD etiology list redistribution of non-informative or miscoded diseases (the “garbage codes”), and modeling the incompleteness of datasets, especially for younger age groups; (4) for the cancer registry, although non-similarities exist across countries in the region, we have not conducted detailed studies in each location, which may have a bias with missing cases in the periphery; and (5) there was no specific EC classification in our study, and we failed to elaborate on the disease burden of different histotypes. These limitations should be addressed by both improving methods, as well as expanding the vital registration systems and population-based cancer registries.

In conclusion, although the EC incidence and prevalence rates are increasing globally, the mortality and DALYs rates decreased in the past few decades. Our findings may be useful for resource allocation and health services planning for a growing number of patients with EC in aging societies, as well as taking greater effort to reduce obesity to reverse the increasing trend. Moreover, the promotion of healthy lifestyle choices and public education on disease symptoms are likely to be the best approaches for EC prevention at the present time.

## Data Availability Statement

The datasets generated for this study can be found in the GBD at http://ghdx.healthdata.org/gbd-results-tool.

## Author Contributions

SZ, T-TG, Y-HZ, and Q-JW contributed to the study conception and design, manuscript drafting, and approval of the final version of the manuscript. F-HL, Y-TJ, HS, and X-XM contributed to acquisition, analysis, or interpretation of data.

### Conflict of Interest

The authors declare that the research was conducted in the absence of any commercial or financial relationships that could be construed as a potential conflict of interest.

## Footnotes

^1^http://gco.iarc.fr/tomorrow/graphic-isotype?type=1&population=900&mode=population&sex=2&cancer=39&age_group=value&apc_male=0&apc_female=0

## Abbreviations

ASIR: age-standardized incidence rate
ASPR: age-standardized prevalence rate
ASDR: age-standardized death rate
APC: annual percentage change
CODEm: Cause of Death Ensemble Model
CI: confidential interval
DALYs: disability-adjusted-life years
EC: endometrial cancer
GBD: Global Burden of Disease
BMI: high body mass index
HRT: hormone replacement therapy
MIR: mortality-to-incidence ratios
PC: percent change
SDI: sociodemographic index
SES: socioeconomic status
TMREL: The theoretical minimum risk exposure level
UI: Uncertainty intervals
YLDs: years of life lived with disability
YLLs: years of life lost.

## References

[1] Morice P Leary A Creutzberg C Abu-Rustum N Darai E. Endometrial cancer. Lancet. (2016) 387:1094–108. 10.1016/S0140-6736(15)00130-0 26354523

[2] Bray F Ferlay J Soerjomataram I Siegel RL Torre LA Jemal A. Global cancer statistics 2018: GLOBOCAN estimates of incidence and mortality worldwide for 36 cancers in 185 countries. CA Cancer J Clin. (2018) 68:394–424. 10.3322/caac.21492 30207593

[3] Torre LA Islami F Siegel RL Ward EM Jemal A. Global cancer in women: burden and trends. Cancer Epidemiol Biomarkers Prev. (2017) 26:444–57. 10.1158/1055-9965.EPI-16-0858 28223433

[4] Ferlay J Soerjomataram I Ervik M Dikshit R Eser S Mathers C GLOBOCAN 2012 v1.0, Cancer Incidence and Mortality Worldwide. IARC CancerBase No. 11. Lyon: International Agency for Research on Cancer (2013).

[5] Ferlay J Soerjomataram I Dikshit R Eser S Mathers C Rebelo M. Cancer incidence and mortality worldwide: sources, methods and major patterns in GLOBOCAN 2012. Int J Cancer. (2015) 136:E359–86. 10.1002/ijc.29210 25220842

[6] Arnold M Karim-Kos HE Coebergh JW Byrnes G Antilla A Ferlay J. Recent trends in incidence of five common cancers in 26 European countries since 1988: analysis of the European Cancer Observatory. Eur J Cancer. (2015) 51:1164–87. 10.1016/j.ejca.2013.09.002 24120180

[7] Siegel RL Miller KD Jemal A Cancer statistics, 2016. CA Cancer J Clin. (2016) 66:7–30. 10.3322/caac.21332 26742998

[8] Kogevinas M Porta M. Socioeconomic differences in cancer survival: a review of the evidence. In: Kogevinas N Pearce N Susser M Boffetta P, editors. Social Inequalities and Cancer. IARC Scientific Publications No. 138. Lyon: International Agency for Research on Cancer (1997). p. 177–206. 9353665

[9] Weiderpass E Antoine J Bray FI Oh JK Arbyn M. Trends in corpus uteri cancer mortality in member states of the European Union. Eur J Cancer. (2014) 50:1675–84. 10.1016/j.ejca.2014.02.020 24656568

[10] Scott OW Tin TS Bigby SM Elwood JM. Rapid increase in endometrial cancer incidence and ethnic differences in New Zealand. Cancer Causes Control. (2019) 30:121–7. 10.1007/s10552-019-1129-1 30671687

[11] Lortet-Tieulent J Ferlay J Bray F Jemal A. International patterns and trends in endometrial cancer incidence, 1978–2013. J Natl Cancer Inst. (2018) 110:354–61. 10.1093/jnci/djx214 29045681

[12] Roth GA Abate D Abate KH Abay SM Abbafati C Abbasi N Global, regional, and national age-sex-specific mortality for 282 causes of death in 195 countries and territories, 1980–2017: a systematic analysis for the Global Burden of Disease Study 2017. Lancet. (2018) 392:1736–88. 10.1016/S0140-6736(18)32203-7 30496103 PMC6227606

[13] James SL Abate D Abate KH Abay SM Abbafati C Abbasi N Global, regional, and national incidence, prevalence, and years lived with disability for 354 diseases and injuries for 195 countries and territories, 1990–2017: a systematic analysis for the Global Burden of Disease Study 2017. Lancet. (2018) 392:1789–858. 10.1016/S0140-6736(18)32279-7 30496104 PMC6227754

[14] Kyu HH Abate D Abate KH Abay SM Abbafati C Abbasi N Global, regional, and national disability-adjusted life-years (DALYs) for 359 diseases and injuries and healthy life expectancy (HALE) for 195 countries and territories, 1990–2017: a systematic analysis for the Global Burden of Disease Study 2017. Lancet. (2018) 392:1859–922. 10.1016/S0140-6736(18)32335-3 30415748 PMC6252083

[15] Fitzmaurice C Allen C Barber RM Barregard L Bhutta ZA Brenner H. Global, regional, and national cancer incidence, mortality, years of life lost, years lived with disability, and disability-adjusted life-years for 32 cancer groups, 1990 to 2015: a systematic analysis for the Global Burden of Disease Study. JAMA Oncol. (2017) 3:524–48. 10.1001/jamaoncol.2016.5688 27918777 PMC6103527

[16] Wang H Naghavi M Allen C Barber RM Bhutta ZA Carter A Global, regional, and national life expectancy, all-cause mortality, and cause-specific mortality for 249 causes of death, 1980–2015: a systematic analysis for the Global Burden of Disease Study 2015. Lancet. (2016) 388:1459–544. 10.1016/S0140-6736(16)31012-1 27733281 PMC5388903

[17] Foreman KJ Lozano R Lopez AD Murray CJ. Modeling causes of death: an integrated approach using CODEm. Popul Health Metr. (2012) 10:1. 10.1186/1478-7954-10-1 22226226 PMC3315398

[18] Fitzmaurice C Akinyemiju TF Al LF Alam T Alizadeh-Navaei R Allen C. Global, regional, and national cancer incidence, mortality, years of life lost, years lived with disability, and disability-adjusted life-years for 29 cancer groups, 1990 to 2016: a systematic analysis for the Global Burden of Disease Study. JAMA Oncol. (2018) 4:1553–68. 10.1200/JCO.2018.36.15_suppl.1568 29860482 PMC6248091

[19] GBD GBD Results tool: Use the Following to Cite Data Included in This Download: Global Burden of Disease Collaborative Network. Global Burden of Disease Study 2017 (GBD 2017) Results. Seattle, WA: Institute for Health Metrics and Evaluation (IHME) (2018).

[20] Naghavi M Abajobir AA Abbafati C Abbas KM Abd-Allah F Abera SF Global, regional, and national age-sex specific mortality for 264 causes of death, 1980–2016: a systematic analysis for the Global Burden of Disease Study 2016. Lancet. (2017) 390:1151–210. 10.1016/S0140-6736(17)32152-9 28919116 PMC5605883

[21] Murray CJ Ezzati M Flaxman AD Lim S Lozano R Michaud C. GBD 2010: design, definitions, and metrics. Lancet. (2012) 380:2063–6. 10.1016/S0140-6736(12)61899-6 23245602

[22] Stanaway JD Afshin A Gakidou E Lim SS Abate D Abate KH. Global, regional, and national comparative risk assessment of 84 behavioural, environmental and occupational, and metabolic risks or clusters of risks for 195 countries and territories, 1990–2017: a systematic analysis for the Global Burden of Disease Study 2017. Lancet. (2018) 392:1923–94. 10.1016/S0140-6736(18)32225-6 30496105 PMC6227755

[23] Hankey BF Ries LA Kosary CL Feuer EJ Merrill RM Clegg LX. Partitioning linear trends in age-adjusted rates. Cancer Causes Control. (2000) 11:31–5. 10.1023/A:1008953201688 10680727

[24] Gao S Yang W Bray F Va P Zhang W Gao J. Declining rates of hepatocellular carcinoma in urban Shanghai: incidence trends in 1976–2005. Eur J Epidemiol. (2012) 27:39–46. 10.1007/s10654-011-9636-8 22160277 PMC5477645

[25] Wallin MT Culpepper WJ Nichols E Bhutta ZA Gebrehiwot TT Hay SI Global, regional, and national burden of multiple sclerosis 1990–2016: a systematic analysis for the Global Burden of Disease Study 2016. Lancet Neurol. (2019) 18:269–85. 10.1016/S1474-4422(18)30443-5 30679040 PMC6372756

[26] Aoki K Sun J Kono A Misumi J. Age-related characteristics of uterine cancer mortality in Japan. Arch Gynecol Obstet. (2005) 273:110–4. 10.1007/s00404-005-0044-8 16047181

[27] Lee JY Kim EY Jung KW Shin A Chan KK Aoki D. Trends in gynecologic cancer mortality in East Asian regions. J Gynecol Oncol. (2014) 25:174–82. 10.3802/jgo.2014.25.3.174 25045429 PMC4102735

[28] International Agency for Research on Cancer GLOBOCAN 2012: Estimated Cancer Incidence, Mortality and Prevalence Worldwide in 2012. Lyon: International Agency for Research on Cancer (2012).

[29] Bray F Loos AH Oostindier M Weiderpass E. Geographic and temporal variations in cancer of the corpus uteri: incidence and mortality in pre- and postmenopausal women in Europe. Int J Cancer. (2005) 117:123–31. 10.1002/ijc.21099 15880435

[30] Prat J Franceschi S Denny L Lazcano Ponce E Stewart BW Wild CP Endometrial cancer. In: Stewart BW Wild CP, editors. World Cancer Report 2014. Lyon: International Agency for Research on Cancer (2014). p. 465–81.

[31] Svanvik T Marcickiewicz J Sundfeldt K Holmberg E Stromberg U. Sociodemographic disparities in stage-specific incidences of endometrial cancer: a registry-based study in West Sweden, 1995–2016. Acta Oncol. (2019) 58:845–51. 10.1080/0284186X.2019.1581947 30849264

[32] Kleemola P Roos E Pietinen P Dietary changes by level of education. J Soc Med. (1996) 33:9–16.

[33] Roos E Prättälä R Lahelma E Kleemola P Pietinen P. Modern and healthy? socio-economic differences in the quality of diet. Europ J Clin Nutr. (1996) 50:753–60. 8933123

[34] Mayen AL Marques-Vidal P Paccaud F Bovet P Stringhini S. Socioeconomic determinants of dietary patterns in low- and middle-income countries: a systematic review. Am J Clin Nutr. (2014) 100:1520–31. 10.3945/ajcn.114.089029 25411287

[35] Afshin A Sur PJ Fay KA Cornaby L Ferrara G Salama JS Health effects of dietary risks in 195 countries, 1990–2017: a systematic analysis for the Global Burden of Disease Study 2017. Lancet. (2019) 393:1958–72. 10.1016/S0140-6736(19)30041-8 30954305 PMC6899507

[36] La Vecchia C Decarli A Fasoli M Gentile A. Nutrition and diet in the etiology of endometrial cancer. Cancer Am Cancer Soc. (1986) 57:1248–53. 10.1002/1097-0142(19860315)57:6<1248::AID-CNCR2820570631>3.0.CO;2-V 3002600

[37] van Lonkhuijzen L Kirsh VA Kreiger N Rohan TE. Endometrial cancer and meat consumption: a case-cohort study. Eur J Cancer Prev. (2011) 20:334–9. 10.1097/CEJ.0b013e328344747c 21422932

[38] Takayama S Monma Y Tsubota-Utsugi M Nagase S Tsubono Y Numata T. Food intake and the risk of endometrial endometrioid adenocarcinoma in Japanese women. Nutr Cancer. (2013) 65:954–60. 10.1080/01635581.2013.818158 24053697

[39] McCann SE Freudenheim JL Marshall JR Brasure JR Swanson MK Graham S. Diet in the epidemiology of endometrial cancer in western New York (United States). Cancer Causes Control. (2000) 11:965–74. 10.1023/A:1026551309873 11142531

[40] Ali AT. Risk factors for endometrial cancer. Ceska Gynekol. (2013) 78:448–59. 24313431

[41] United Nations, Department of Economic and Social Affairs Population Division World Fertility Report 2013: Fertility at the Extremes. (2014). Available online at: http://www.un.org/en/development/desa/population/publications/pdf/fertility/worldFertilityReport2013.pdf (accessed October 9, 2017).

[42] Lundberg V Tolonen H Stegmayr B Kuulasmaa K Asplund K. Use of oral contraceptives and hormone replacement therapy in the WHO MONICA project. Maturitas. (2004) 48:39–49. 10.1016/j.maturitas.2003.08.006 15223107

[43] Reis N Beji NK. Risk factors for endometrial cancer in Turkish women: results from a hospital-based case-control study. Eur J Oncol Nurs. (2009) 13:122–7. 10.1016/j.ejon.2009.01.007 19332387

[44] Dossus L Allen N Kaaks R Bakken K Lund E Tjonneland A. Reproductive risk factors and endometrial cancer: the European Prospective Investigation into Cancer and Nutrition. Int J Cancer. (2010) 127:442–51. 10.1002/ijc.25050 19924816

[45] Charneco E Ortiz AP Venegas-Rios HL Romaguera J Umpierre S. Clinic-based case-control study of the association between body mass index and endometrial cancer in Puerto Rican women. P R Health Sci J. (2010) 29:272–8. 20799515 PMC3040409

[46] Reeves GK Pirie K Beral V Green J Spencer E Bull D. Cancer incidence and mortality in relation to body mass index in the Million Women Study: cohort study. BMJ. (2007) 335:1134. 10.1136/bmj.39367.495995.AE 17986716 PMC2099519

[47] Tangjitgamol S Anderson BO See HT Lertbutsayanukul C Sirisabya N Manchana T. Management of endometrial cancer in Asia: consensus statement from the Asian Oncology Summit 2009. Lancet Oncol. (2009) 10:1119–27. 10.1016/S1470-2045(09)70290-6 19880066

[48] Lopez J Banerjee S Kaye SB. New developments in the treatment of ovarian cancer–future perspectives. Ann Oncol. (2013) 24(Suppl. 10):x69–76. 10.1093/annonc/mdt475 24265409 PMC3836570

[49] Lee YC Lheureux S Oza AM. Treatment strategies for endometrial cancer: current practice and perspective. Curr Opin Obstet Gynecol. (2017) 29:47–58. 10.1097/GCO.0000000000000338 27941361

[50] Braun MM Overbeek-Wager EA Grumbo RJ. Diagnosis and management of endometrial cancer. Am Fam Phys. (2016) 93:468–74. 26977831

[51] Siegel RL Miller KD Jemal A Cancer statistics, 2018. CA Cancer J Clin. (2018) 68:7–30. 10.3322/caac.21442 29313949

[52] Tan W Stehman FB Carter RL. Mortality rates due to gynecologic cancers in New York state by demographic factors and proximity to a Gynecologic Oncology Group member treatment center: 1979–2001. Gynecol Oncol. (2009) 114:346–52. 10.1016/j.ygyno.2009.03.033 19411096

[53] Flaxman AD Vahdatpour A James SL Birnbaum JK Murray CJ. Direct estimation of cause-specific mortality fractions from verbal autopsies: multisite validation study using clinical diagnostic gold standards. Popul Health Metr. (2011) 9:35. 10.1186/1478-7954-9-35 21816098 PMC3160928

[54] Mikkelsen L Phillips DE AbouZahr C Setel PW de Savigny D Lozano R. A global assessment of civil registration and vital statistics systems: monitoring data quality and progress. Lancet. (2015) 386:1395–406. 10.1016/S0140-6736(15)60171-4 25971218

[55] Lin L Yan L Liu Y Yuan F Li H Ni J. Incidence and death in 29 cancer groups in 2017 and trend analysis from 1990 to 2017 from the Global Burden of Disease Study. J Hematol Oncol. (2019) 12:96. 10.1186/s13045-019-0783-9 31511035 PMC6740016

