# Supplementary material for: Global, Regional, and National Burden of Endometrial Cancer, 1990–2017: Results From the Global Burden of Disease Study, 2017
Source: Front Oncol. 2019 Dec 19;9:1440. doi: 10.3389/fonc.2019.01440 (PMC6930915; doi:10.3389/fonc.2019.01440)
Supplement: Supplementary file 1 [file Table_1.DOC]

**Supplemental file 1**

[**eMethods** 3](#__RefHeading___Toc10717)

[Definition of indicator 3](#__RefHeading___Toc16090)

[**Data sources** 3](#__RefHeading___Toc4481)

[Cancer incidence data sources 3](#__RefHeading___Toc15713)

[Mortality/incidence ratio data sources 3](#__RefHeading___Toc1076)

[Cancer mortality data sources 4](#__RefHeading___Toc28309)

[Bias of categories of input data 4](#__RefHeading___Toc22611)

[**Data analysis** 4](#__RefHeading___Toc12327)

[**Cancer registry data formatting** 4](#__RefHeading___Toc31342)

[**Cause of death database formatting** 7](#__RefHeading___Toc13189)

[**CoD data types** 7](#__RefHeading___Toc22767)

[**CODEm models** 7](#__RefHeading___Toc27991)

[**CodCorrect** 8](#__RefHeading___Toc32284)

[Incidence estimation 8](#__RefHeading___Toc3587)

[**Prevalence and YLD estimation** 8](#__RefHeading___Toc21269)

[**Probability of cancer** 10](#__RefHeading___Toc13113)

[**YLL and DALY estimation** 10](#__RefHeading___Toc7090)

[**Socio-Demographic Index (SDI) Definitions & Method** 10](#__RefHeading___Toc17096)

[Overview 10](#__RefHeading___Toc31348)

[Development of revised SDI indicator 10](#__RefHeading___Toc8535)

[**Estimation of major risk factors for uterine cancer** 12](#__RefHeading___Toc1257)

[**High body-mass index** 12](#__RefHeading___Toc19384)

[**Data** 12](#__RefHeading___Toc29737)

[**Modelling strategy** 14](#__RefHeading___Toc20457)

[**Theoretical minimum risk exposure level** 16](#__RefHeading___Toc6753)

[**References** 17](#__RefHeading___Toc19199)

[**Supplementary Table 1.** GATHER：Guidelines for Accurate and Transparent Health Estimates Reporting 20](#__RefHeading___Toc19843)

[**Supplementary Table 2.** Sources for cancer incidence and mortality-to-incidence ratio data by country, year, and registry 23](#__RefHeading___Toc31633)

[**Supplementary Table 3.**Disability weights 56](#__RefHeading___Toc14475)

[**Supplementary Table 4.** Socio-Demographic Index groupings by location, based on 2017 values 58](#__RefHeading___Toc13407)

[**Supplementary Table 5.** CODEm covariates used, level of covariate, and expected direction of covariate for uterine cancer by cause, sex, and age 69](#__RefHeading___Toc20489)

[**Supplementary Table 6.** Results for CODEm model testing 71](#__RefHeading___Toc6472)

[**Supplementary Table 7.** Comparison of GBD 2016 and GBD 2017 covariates used and level of covariates 72](#__RefHeading___Toc19398)

[**Supplementary Table 8.** Total number of site years by cause and source type for GBD 2017 74](#__RefHeading___Toc8392)

[**Supplementary Table 9.** Duration of four prevalence phases by cancer 75](#__RefHeading___Toc22206)

[**Supplementary Figure 1.** Flowchart, uterine cancer mortality, YLL estimation 76](#__RefHeading___Toc26264)

[**Supplementary Figure 2.** Flowchart, uterine cancer incidence, prevalence, YLD^a^ estimation 77](#__RefHeading___Toc28616)

[**Supplementary Figure 3.** Vital Registration and Verbal Autopsy data availability by country, 1980-2017 78](#__RefHeading___Toc42)

[**Supplementary Figure 4**. Comparative risk assessment to estimate population attributable fractions for risk factors. 80](#__RefHeading___Toc2211)

[**Supplementary Figure 5.** High body-mass index: data and model flow chart. 81](#__RefHeading___Toc18957)

This supplementary material has been provided by the authors to give readers additional information about their work.

# eMethods[[1]](#footnote-2)

## Definition of indicator

In this publication estimates for uterine cancer, for female, for the time from 1990 to 2017 are presented for 195 countries or territories. Benign uterine neoplasms were defined as any non-invasive uterine growth, except for uterine fibroids. All ICD9 and ICD10 codes pertaining to uterine cancer (182-182.9, 233.2 and C54-C54.9, D07.0-D07.2, D26.1-D26.9, respectively) are being included in these estimates.

# Data sources

## Cancer incidence data sources

Cancer incidence was sought from individual cancer registries or aggregated databases of cancer registry data like “Cancer Incidence In Five Continents (CI5)”(1-10), EUREG(11), or NORDCAN(12). Data were excluded if they were not representative of the coverage population (e.g., hospital-based registries), if they did not cover all malignant neoplasms as defined in ICD9 (140-208) or ICD10 (C00-C96) (e.g. specialty cancer registry), if they did not include data for all age groups, if the data were limited to years prior to 1980, or if the source did not provide details on the population covered. Preference was given to registries with national coverage over those with only local coverage, except those from countries where the GBD study provides subnational estimates. A list of the cancer registries included in our analysis and the years covered can be found elsewhere(13). Additional metadata for each source are available in the online GBD citation tool, <http://ghdx.healthdata.org/gbd-2017/data-input-sources>.

## Mortality/incidence ratio data sources

Most cancer registries only report cancer incidence. However, if a cancer registry also reported cancer mortality, mortality data were also extracted from the source to be used in the mortality to incidence estimation. Supplementary Table 2 lists the registries used for the estimation of mortality-to-incidence ratios.

## Cancer mortality data sources

A detailed description of the data sources and processing steps for the cause of death database can be found in the supplement to the paper “Global, regional, and national age-sex specific mortality for 264 causes of death, 1980-2016: a systematic analysis for the Global Burden of Disease Study 2016”(14).

## Bias of categories of input data

Cancer registry data can be biased in multiple ways. A high proportion of ill-defined cancer cases in the registry data requires redistribution of these cases to other cancers, which introduces a potential for bias. Changes between coding systems can lead to artificial differences in disease estimates; however, we adjust for this bias by mapping the different coding systems to the GBD causes. Underreporting of cancers that require advanced diagnostic techniques can be an issue in cancer registries from low-income countries. On the other hand, misclassification of metastatic sites as primary cancer can lead to overestimation of cancer sites that are common sites for metastases, like brain or liver. Since many cancer registries are located in urban areas, the representativeness of the registry for the general population can also be problematic. The accuracy of mortality data reported in cancer registries usually depends on the quality of the vital registration system. If the vital registration system is incomplete or of poor quality, the mortality-to-incidence ratio can be biased to lower ratios.

# Data analysis

Flowcharts describing the conceptual overview of the data processing are available in Supplementary Figure 1 and Supplementary Figure 2.

# Cancer registry data formatting

Cancer registry data went through multiple processing steps before integration with the COD database.

First, the original data were transformed into standardized files, which included standardization of format, categorization, and registry names (step 1 in Supplementary Figure 1).

Second, some cancer registries report individual codes as well as aggregated totals (e.g., C18, C19, and C20 are reported individually but the aggregated group of C18-C20 (colorectal cancer) is also reported in the registry data). The data processing step “subtotal recalculation” (step 2 in the flowchart) verifies these totals and subtracts the values of any individual codes from the aggregates.

In the third step (step 3 in the flowchart), cancer registry incidence data and cancer registry mortality data are mapped to GBD causes. A different map is used for incidence and for mortality data because of the assumption that there are no deaths for certain cancers.

In the fourth data processing step (step 4 in the flowchart), cancer registry data were standardized to the GBD age groups. Age-specific incidence rates were generated using CI5, SEER, and NORDCAN data, while age-specific mortality rates were generated from the CoD data(15). Age-specific weights were then generated by applying the age-specific rates to a given registry population that required age-splitting to produce the expected number of cases/deaths for that registry by age. The expected number of cases/deaths for each age, and cancer were then normalized to 1, creating final, age-specific proportions. These proportions were then applied to the total number of cases/deaths by cancer to get the age-specific number of cases/deaths.

In the fifth step (step 5 in the flowchart), data for cause entries that are aggregates of GBD causes were redistributed. Examples of these aggregated causes include some registries reporting ICD10 codes C00-C14 together as, “lip, oral cavity, and pharyngeal cancer.” These groups were broken down into subcauses that could be mapped to single GBD causes. In this example, those include lip and oral cavity cancer (C00‐C08), nasopharyngeal cancer(C11),cancer of other parts of the pharynx (C09‐C10, C12-C13), and “Malignant neoplasm of other and ill-defined sites in the lip, oral cavity, and pharynx” (C14). To redistribute the data, weights were created using the same method employed in age-sex splitting (see step four above). For the undefined code (C14 in the example) an “average all cancer” weight was used, which was generated by adding all cases rom SEER/NORDCAN/CI5 and dividing those by the combined population. Then, proportions were generated by subcause for each aggregate cause as in the sex splitting example above (see step four). The total number of cases from the aggregated group (C00-C14) was recalculated for each subgroup and the undefined code (C14). C14 was then redistributed as a “garbage code” in step six. Distinct proportions were used for C46 (Kaposi sarcoma). C46 entries were redistributed as “other cancer” and HIV.

In the sixth step (step 6 in the flowchart), unspecified codes (“garbage code”) were redistributed. Redistribution of cancer registry incidence and mortality data mirrored the process of the redistribution used in the cause of death database and has not changed compared to GBD 2013(16).

In the seventh step (step 7 in the flowchart), duplicate or redundant sources were removed from the processed cancer registry dataset. Duplicate sources were present if, for example, the cancer registry was part of the CI5 database but we also had data from the registry directly. Redundancies occurred and were removed as described in “Inclusion and Exclusion Criteria,” where more detailed data were available, or when national registry data could replace regionally representative data. From here, two parallel selection processes were run to generate input data for the MI models and to generate incidence for final mortality estimation. Higher priority was given to registry data from the most standardized source when creating the final incidence input, whereas for the MI model input, only sources that reported incidence and mortality were used. This is different from GBD 2015, where mortality and incidence could come from different sources as long as they covered the same population.

In the eighth step (step 8 in the flowchart) the processed incidence and mortality data from cancer registries were matched by cancer, age, year, and location to generate MI ratios. These MI ratios were used as input for a three-step modelling approach using the updated GBD 2017 ST-GPR approach. With the Healthcare Access and Quality (HAQ) Index as a covariate in the linear step mixed effects model using a logit link function(17).

logit (*MI ratio_c,a,s,t_*) = ɑ+β_1_*HAQI_c,t_*+$\mathop{\Sigma}\limits_{a}^{A}$β_2_*I_a_*+ β_3_*I_s_*+ ϵ*_c,a,s,t_*

c: country, a: age group, t: time (years); s: sex

HAQI: Healthcare Access and Quality index

I: indicator variable

ϵc,a,s,t: error term

This is different from GBD 2016, where we used Socio-demographic Index (SDI) as a predictive covariate. Predictions were made without the random effects. The ST-GPR model has three main hyper-parameters that control for smoothing across time, age, and geography. The time adjustment parameter (λ) was set to 2, which aims to borrow strength from neighbouring time points (ie, the exposure in this year is highly correlated with exposure in the previous year but less so further back in time). The age adjustment parameter ω was set to 0.5, which borrows strength from data in neighbouring age groups. The space adjustment parameter ξ was set to 0.95 in locations with data and to 0.5 in locations without data (the higher space adjustment parameter ξ was applied when at least one age-sex group in the country of estimation had at least five unique data points. The lower space adjustment parameter ξ was applied when estimating data-scarce countries). Zeta aims to borrow strength across the hierarchy of geographical locations(18). For the amplitude parameter in the Gaussian process regression we used 2, and for the scale we used a value of 15.

For GBD 2017 we slightly changed the data cleaning process and used HAQ rather than SDI to exclude data. For each cancer, MI ratios from locations in HAQ quintiles 1-4 were dropped if they were below the median of MI ratios from locations in HAQ quintile 5. We also dropped MI ratios from locations in HAQ quintiles 1-4 if the MI ratios were above the third quartile + 1.5 * IQR (inter-quartile range). We dropped all MIR that were based on less than 25 cases to avoid noise due to small numbers except for mesothelioma and acute lymphoid leukaemia, where we dropped MIR that were based on fewer than ten cases because of lower data availability for these two cancers. We also aggregated incidence and mortality to the youngest five-year age bin where we had at least 50 data points to avoid MIR predictions in young age groups that were based on few data points. The MIR in the age-bin that was used to aggregate MIR was used to backfill the MIR for younger age groups.

Since MI ratios can be above 1, especially in older age groups and cancers with low cure rates, we used the 95^th^ percentile of the cleaned dataset that only included MIR that were based on 50 or more cases, to cap the MIR input data. This “upper cap” was used to allow MIR over 1 but to constrain the MIR to a maximum level. To run the logit model, the input data were divided by the upper caps and model predictions after ST-GPR was rescaled by multiplying them by the upper caps. To constrain the model at the lower end, we used the fifth percentile of the cancer-specific cleaned MIR input data to replace all model predictions with this lower cap.

Final MI ratios were matched with the cancer registry incidence dataset in the ninth step (step 9 in the flowchart) to generate mortality estimates (Incidence * Mortality/Incidence = Mortality) (step 10 in the flowchart). The final mortality estimates were then uploaded into the COD database (step 11 in the flowchart). Cancer-specific mortality modelling then followed the general CODEm process.

# Cause of death database formatting

Formatting of data sources for the cause of death database has been described in detail elsewhere (step 11 in the flowchart)(14).

# CoD data types

The CoD database contains seven types of data sources(Supplementary Table 8): vital registration (VR), verbal autopsy (VA), cancer registry (CR), police records, sibling history, surveillance, and survey/census. The highest-quality data have detailed demographic group characteristics and detailed CoD across the time series. Data from countries with complete VR systems are considered to be high-quality. For countries with incomplete VR systems, vital statistics for causes of death may be supplemented with other data types to provide cause-specific estimates.(Supplementary Figure 3)

# CODEm models

Mortality estimates for each cancer were generated using CODEm (step 12 in the flowchart). Methods describing the CODEm approach have been described elsewhere(14, 19). In brief, the CODEm modeling approach is based on the principles that all types of available data should be used even if data quality varies; that individual models but also ensemble models should be tested for their predictive validity; and that the best model or sets of models should be chosen based on the out of sample predictive validity. Models were run separately for countries with extensive and complete vital registration data and countries with less VR data to prevent an inflation in the uncertainty around the estimates in “data-rich” countries. Covariates were selected based on a possible predictive relationship between the covariate and the specific cancer mortality. Level 1 covariates have a proven strong relationship with the outcome such as etiological or biological roles. Level 2 covariates have a strong relationship but not a direct biological link. Covariates that are more distal in the causal chain or are mediated through Level 1 or 2 covariates are categorized as Level 3.(19) Differences in covariate selection between GBD 2016 and GBD 2017 by cause and direction of the covariate can be found in Supplementary Table 7.

# CodCorrect

CODEm models estimate the individual cause-level mortality without taking into account the all-cause mortality (step 13 in the flowchart). To ensure that all single causes add up to the all-cause mortality and that all child-causes add up to the parent cause, an algorithm called “CodCorrect” is used (step 14 and step 15 in the flowchart). Details regarding the algorithm can be found elsewhere(14).

## Incidence estimation

GBD cancer incidence estimates were generated by dividing final liver cancer mortality estimates (after CodCorrect adjustment and etiology splits) by the liver cancer MI ratios (step 1 in Supplementary Figure 2). To propagate uncertainty from the MI ratios and the mortality estimates to incidence, this process was done at the 1,000-draw level. It was assumed that uncertainty in the MI ratio is independent of uncertainty in the estimated age-specific death rates.

# Prevalence and YLD estimation

Prevalence is estimated as 10-year prevalence for all cancers. After transforming the final GBD cancer mortality estimates to incidence estimates (step 1 in the flowchart), incidence was combined with the relative yearly survival estimates up to 10 years (step 7 in the flowchart). For GBD 2017 we updated our methods to more directly utilize MIRs to generate these yearly cancer relative survival estimates.

Previous reports suggest that the value of (1 – MIR) may serve as a proxy for 5-year relative survival, with the exact correlation varying slightly by cancer type(20). We used SEER*Stat to obtain national mortality, incidence, and relative survival statistics from the nine SEER registries reporting from 1980 to 2014 (step 2), by cancer type, sex, 5-year blocks (i.e., 1980–1984, 1985–1989, etc.), and 5-year age groups (except combining 80+). For each cancer, we modeled 5-year relative survival with the SEER MIRs using Poisson regression, weighted by the number of incident cases (step 3). To reduce variability due to small samples, we only included MIRs based on at least 25 incident cases (except for the rarer cancers mesothelioma, nasopharyngeal cancer, and acute myeloid leukemia, where MIRs based on at least 10 cases were included). These models were then applied to the GBD MIR estimates to predict an estimated 5-year survival for each age/sex/year/location (winsorized to between 0 and 100% survival;

step 4). To obtain yearly survival estimates up to 10 years, we compared these estimates to the SEER sex-specific all-ages relative survival statistics from 2004 (the latest year with 10-year survival available). The proportion of the predicted GBD survival estimate to the SEER survival statistic was used to scale the SEER 10-year relative survival curve for each country (step 5).

To transform relative to absolute survival (adjusting for background mortality), GBD 2017 lifetables were used (step 6 and 7 in the flowchart) to calculate lambda values: lambda= (ln(nLxn/nLxn+1))/5, where nLx=person years lived between ages x and x+n (from GBD lifetable). Absolute survival was then calculated using an exponential survival function (absolute survival = relative survival * elambda*t). Survivors beyond 10 years were considered cured. The survivor population prevalence was divided into two sequelae (1. diagnosis and primary therapy; 2. controlled phase). The yearly prevalence of the population that did not survive beyond 10 years was divided into the four sequelae by assigning the fixed durations for each of the diagnosis and primary therapy phase, metastatic phase, and terminal phase, and assigning the remaining prevalence to the controlled phase (step 9 in the flowchart). Duration of these four sequelae remained the same as for GBD 2016. Supplementary Table 9 lists the duration of each, along with the sources used to determine their length. YLDs were calculated by multiplying each phase with the respective disability weight(Supplementary Table 3). To generate the total YLDs for each cancer (with the exception of cancers where additional disability is added due to procedures – see next paragraph) the YLDs for each cancer sequela were added (step 13 in Supplementary Figure 2).

Additional disability was estimated for breast cancer (disability due to mastectomy), larynx cancer (disability due to laryngectomy), colon and rectum cancer (disability due to stoma), bladder cancer (disability due to incontinence), and prostatectomy (disability due to incontinence and impotence) (#10 in eFigure 2). Hospital data were used to estimate the number of cancer patients undergoing mastectomy, laryngectomy, stoma, prostatectomy, and cystectomy. These proportions remained the same as in GBD 2013, GBD 2015, and GBD 2016 and were used as input for proportion models that were run in DisMod-MR 2.1 (#9 in eFigure 2)(20). The procedure proportion (proportion of cancer population that undergoes procedures) from hospital data was used as input for a proportion model in DisMod-MR 2.1 in order to estimate the proportions for all locations, by age, and by sex.

The final procedure proportions were applied to the incidence cases of the respective cancers and multiplied with the proportion of the incidence population surviving for 10 years to determine the incident cases of the cancer population that underwent procedures and that survived beyond 10 years. These incident cases were used again as an input for DisMod-MR 2.1, with a remission specification of zero and an excess mortality rate prior of 0 to 0.1, as well as with increasing the age of the population

and the year by 10 years to reflect prevalence after that population has survived 10 years. This approach was updated compared to GBD 2016, where we did not include an age or time shift. The results from this model are incidence and lifetime prevalent cases of persons with these cancer-related sequelae who have survived beyond 10 years.

We assumed that for the population surviving up to 10 years, only the prevalence population being in remission experiences additional disability due to procedures (e.g., a woman suffering from metastatic breast cancer does not experience additional disability due to a mastectomy during this phase). To estimate the prevalence of the cancer population in remission during the first 10 years after diagnosis with and without procedure-related disability, we multiplied the prevalence of the population in the remission phase with the proportion of the population undergoing a procedure. This step allowed us to estimate disability during the remission phase for both the population experiencing disability due to the remission phase alone, as well as the population experiencing disability from the remission phase and the additional procedure-related disability.

Lastly, the procedure sequelae prevalence and general sequelae prevalence were multiplied with their respective disability weights (Supplementary Table 3) to obtain the number of YLDs (steps 11, 12, 13 in the flowchart). The sum of these YLDs is the final YLD estimate associated with each cancer.

# Probability of cancer

The cumulative probability of developing cancer for certain age groups and an approximated lifetime risk for all cancer groups (age 0 to 79) as well as the odds of developing cancer for 2017 were calculated. The method use does not take into account competing risks of death. The cancer risk is approximated

using the following formula(21).

$$Cumulativerisk=1-e^{-cumulativerate}$$

# YLL and DALY estimation

The number of years of life lost (YLLs) due to cancer deaths was calculated by multiplying the number of deaths in each age group by the normative standard life expectancy for the corresponding age group.

# Socio-Demographic Index (SDI) Definitions & Method

## Overview

The Socio-demographic Index (SDI) is a composite indicator of development status strongly correlated with health outcomes. In short, it is the geometric mean of 0 to 1 indices of total fertility under 25 (TFU25), mean education for those aged 15 and older (EDU15+), and lag distributed income (LDI) per capita.

## Development of revised SDI indicator

SDI was originally constructed for GBD 2015 using the Human Development Index (HDI) methodology, wherein a 0 to 1 index value was determined for each of the original three covariate inputs (TFR in ages 15 to 49, EDU15+, and LDI per capita) using the observed minima and maxima over the estimation period to set the scales.

In response to feedback from collaborators and the evolution of the GBD, we have refined the indicator with each GBD cycle. For GBD 2017, in conjunction with our expanded estimation of age-specific fertility, we replaced total fertility rate (TFR) with TFU25 as one of the three component indices. The TFU25 provides a better measure of women’s status in society, as it focuses on ages where childbearing disrupts the pursuit of education and entrance into the workforce. The concordance correlation coefficient between SDI using the GBD 2016 method and the updated method for GBD 2017 was 0.981.

During GBD 2016 we moved from using relative index scales to absolute scales to enhance the stability of SDI’s interpretation over time, as we noticed that the measure was highly sensitive to the addition of subnational units that tended to stretch the empirical minima and maxima. We selected the minima and maxima of the scales by examining the relationships each of the inputs had with life expectancy at birth and under-5 mortality and identifying points of limiting returns at both high and low values, if they occurred prior to theoretical limits (e.g., a TFU25 of 0).

Thus, an index score of 0 represents the minimum level of each covariate input past which selected health outcomes can get no worse, while an index score of 1 represents the maximum level of each covariate input past which selected health outcomes cease to improve. As a composite, a location with an SDI of 0 would have a theoretical minimum level of development relevant to these health outcomes, while a location with an SDI of 1 would have a theoretical maximum level of development relevant to these health outcomes.

The final scales for GBD 2017 are summarized in Supplementary Table below.

| Input | Lower Bound | Upper Bound |
| --- | --- | --- |
| TFU25 | 0 | 3 |
| LDI per capita 60,000 | 250 USD (5.52 log USD) ^a^ | USD (11.00 log USD) |
| EDU15+ | 0 years | 17 years |

^a^ The minimum for the LDI scale was originally set at the theoretical limit of 0 USD, as we did not observe an asymptotic relationship between log(LDI) and E 0 or 5q0 at lower values of log(LDI). Empirically, however, we also did not observe an LDI below 350 USD (5.86 log USD) for the estimation period 1970-2016. In log-space, this meant that approximately half of our scale was not being utilized, compressing the observed variation in LDI and diminishing its meaningful contribution to SDI. Accordingly, we set the lower limit on LDI to 250 USD (5.52 log USD) to ensure we were fully utilizing the range of the scale to capture its variation across space and time, as is the case with the other two inputs.

Using scales described above, we computed the index scores underlying SDI as follows:

$$\mathrm{I}_{\mathrm{Cly}}=\frac{C_{ly}-C_{low}}{C_{high}-C_{low}}$$

Where – the index for covariate C, location l, and year y – is equal to the difference between the value of that covariate in that location-year and the lower bound of the covariate divided by the difference between the upper and lower bounds for that covariate. If the values of input covariates fell outside the upper or lower bounds (e.g. LDI per capita greater than 60,000 USD), they were mapped to the respective upper or lower bounds. The index value for TFU25 was computed as 1 − , as lower TFU25s correspond to higher levels of development, and thus higher index scores. For GBD 2017 we expanded the computation of SDI to 890 national and subnational locations spanning the time period 1950-2017.

The composite SCI was the geometric mean of these three indices for a given location-year. The cutoff values used to determine quintiles for analysis were then computed using country-level estimates of SDI for the year 2017, excluding countries with populations less than 1 million. Socio-Demographic Index groupings by location, based on 2017 value provided in Supplementary Table 4.

# Estimation of major risk factors for uterine cancer

GBD classifies risk factors into three major categories: behavioral, environmental/occupational, and metabolic. For each risk factor, the attributable burden was estimated by comparing observed deaths to those that would have been observed if a counterfactual level of exposure had occurred in the past. Theoretical minimum risk exposure level (TMREL), which is the level of risk exposure that minimizes risk of diseases at the population level, was used to compute attributable disease burden.

The approach used in GBD 2017 for comparative risk assessment to estimate population attributable fractions for risk factors is shown in the Supplementary Figure 4.

We describe details of one major risk factor related to uterine cancer, high body-mass index. Description of other risk factor can be found in the GBD 2017 risk factor paper(22).

# High body-mass index

For the purpose of attributing disease burden to high body-mass index (BMI), the theoretical minimum risk exposure level for BMI in adults (ages 20+ years) was estimated to range between 20 to 25 kg/m^2^ (mean 22.5 kg/m^2^) based on the BMI level that was associated with the lowest risk of all-cause mortality in prospective cohort studies, and for children (age up to 19 years) was based on International Obesity Task Force (IOTF) cut-offs for normal weight. The risk-outcome pairs to attribute burden of specific conditions to high BMI were defined based on the strength of available evidence supporting a causal effect of BMI in meta-analysis. To include the uncertainty in the TMREL, we took a random draw from the uniform distribution of the interval between 20 and 25 kg/m^2^ each time the population attribuSupplementary Table burden was calculated.

The steps in the estimation of disease burden attribuSupplementary Table to high BMI are shown in the Supplementary Figure 5 flowchart for adults and children.

# Data

We systematically searched Medline to identify studies providing nationally or subnationally representative estimates of overweight prevalence, obesity prevalence, or mean BMI. We included representative studies providing data on mean BMI or prevalence of overweight or obesity among adults or children. For adults, studies were included if they defined overweight as BMI≥25 kg/m^2^ and obesity as BMI≥30 kg/m^2^, or if estimates using those cut-offs could be back-calculated from reported categories. For children, studies were included if they used IOTF standards to define overweight and obesity thresholds. Studies were excluded if using nonrandom samples (e.g., case-control studies or convenience samples); conducted among specific subpopulations (e.g., pregnant women, racial or ethnic minorities, immigrants, or individuals with specific diseases); using alternative methods to assess adiposity (e.g., waist-circumference, skin-fold thickness, or hydrodensitometry); having sample sizes of less than 20 per age-sex group; or providing inadequate information on any of the inclusion criteria.

Where individual-level survey data were available, we computed mean BMI using weight and height and then used BMI to determine the prevalence of overweight and obesity. For individuals aged over 18 years, we considered them to be overweight if their BMI was greater than or equal to 25 kg/m^2^, and obese if their BMI was greater than or equal to 30 kg/m^2^. For individuals aged 2-18 years, we used monthly IOTF cut-offs to determine overweight and obese status when age in months was available. When only age in years was available, we used the cut-off for the 6 month of that year. Individuals who were obese were also considered to be overweight. We excluded studies using the World Health Organization (WHO) standards or country-specific cut-offs to define childhood overweight and obesity. At the individual-level, we considered BMI<10 kg/m^2^ and BMI>70 kg/m^2^ to be biologically implausible and excluded those observations.

The rationale for choosing to use the IOTF cut-offs over the WHO standards was that the IOTF cut-offs provide consistent child-specific standards for ages 2-18 derived surveys covering multiple countries. On the other hand, the WHO growth standards apply to children under 5 and the WHO growth reference applies to children ages 5-19. The WHO growth reference for children ages 5-19 was derived from United States data which is less representative than the multinational data used by IOTF. Additionally, the switch between references at age 5 can produce artificial discontinuities. Given that we estimate global childhood overweight and obesity for ages 2-19 (with ages 19 using standard adult cut-offs), the IOTF cut-offs were preferable. Additionally, we found that IOTF cut-offs were more commonly used in scientific literature covering childhood obesity.

From report and literature data, we extracted data on mean BMI, prevalence of overweight, and prevalence of obesity, measures of uncertainty for each, and sample size, by the most granular age and sex groups available. Additionally, we extracted the same study-level covariates as were extracted from microdata (measurement, urbanicity, and representativeness), as well as location and year.

We included both measured and self-reported data. Of the 72.6 million person-years of data globally, 18.8 million (26%) were self-reported. We tested for bias in self-report data compared to measured data, which is considered to be the gold-standard. There was no clear direction of bias for children ages 2-14, so for data for overweight prevalence, obesity prevalence, and mean BMI using the following nested hierarchical mixed-effects regression models, fit using restricted maximum likelihood separately by sex:

logit_(overweight)c,a,t_ = β_0_ + β_1m_ + $\sum_{k=2}^{19} \beta_{k}IA\left[a\right]$ + $\sum_{i=20}^{55} \beta1IA\left[a\right]IM\left[m\right]$ + αs + αsm + αr + αrm + αc + αcm + αt + αtm + ϵ_c,a,t_

logit_(obseity)c,a,t_ = β_0_ + β_1m_ + $\sum_{k=2}^{19} \beta_{k}IA\left[a\right]$ + $\sum_{i=20}^{55} \beta1IA\left[a\right]IM\left[m\right]$ + αs + αsm + αr + αrm + αc + αcm + αt + αtm + ϵ_c,a,t_

logit_(BMI)c,a,t_ = β_0_ + β_1m_ + $\sum_{k=2}^{19} \beta_{k}IA\left[a\right]$ + $\sum_{i=20}^{55} \beta1IA\left[a\right]IM\left[m\right]$ + αs + αsm + αr + αrm + αc + αcm + αt + αtm + ϵ_c,a,t_

Where m is a fixed effect on measurement (binary, either measured (1) or self-report (0)), IA[a] is an indicator variable for specific age group A, IA[a]IM[m] is an interaction term between age and measurement, αs, αr, and αc are random effects at the super region, region, country, and subnational level respectively, and αt is a random effect by time-period (1980-1989, 1990-1999, 2000-2009, 2010-2017). Random effects at the country- or state-level and time-period level were used to fit the models, but were taken as noise and were not used in adjustment of self-reported data. We propagated the uncertainty in the self-report adjustment model by adding the variance of each of the regression coefficients used in adjustment to the data variance in delta-transformed space. After adjustment, regressions confirmed that self-reported data was no longer significantly different from measured data.

# Modelling strategy

After adjusting for self-report bias and splitting aggregated data into 5-year age-sex groups, we used ST-GPR to estimate the prevalence of overweight and obesity.

The linear model, which when added to the smoothed residuals forms the mean prior for GPR is as follows:

logit_(overweight)c,a,t_ = β_0_ + β_1energyc, t_ + β_2SDIc_, t + β_3vehiclesc, t_ + β_4agriculture_ +$\sum_{k=5}^{22} \beta_{k}IA\left[\left.a\right]\right.$ + αs + αr +αc

logit_(obesity/overweight)c,a,t_ = β_0_ + β_1energyc, t_ + β_2SDIc, t_ + β_3vehiclesc, t_ + $\sum_{k=4}^{22} \beta_{k}IA\left[\left.a\right]\right.$ + αs + αr +αc

Where energy is ten-year lag-distributed energy consumption per capita, Socio-demographic Index (SDI) is a composite index of development including lag-distributed income per capita, education, and fertility, vehicles is the number of two or four-wheel vehicles per capita, and agriculture is the proportion of the population working in agriculture. IA[a] is a dummy variable indicating specific age group A that the prevalence point captures, and αs, αr, and αc are super region, region, country, and subnational random intercepts, respectively. Random effects were used in model fitting but were not used in prediction.

We tested all combinations of the following covariates to see which performed best in terms of in-sample AIC for the overweight linear model and the obesity as a proportion of overweight linear model: ten-year lag distributed energy per capita, proportion of the population living in urban areas, SDI, lag-distributed income per capita, educational attainment (years) per capita, proportion of the population working in agriculture, grams of sugar adjusted for energy per capita, grams of sugar not adjusted for energy per capita, and the number of two or four-wheeled vehicles per capita. We selected these candidate covariates based on theory as well as reviewing covariates used in other publications. The final linear model was selected based on: 1) if the direction of covariates matched what is expected from theory, 2) all the included covariates were significant, and 3) minimizing in-sample AIC. The covariate selection process was performed using the dredge package in R.

To estimate the mean BMI for adults in each country, age, sex, and time period 1980-2017, we first used the following nested hierarchical mixed-effects model, fit using restricted maximum likelihood on data from sources containing estimates of all three indicators (prevalence of overweight, prevalence of obesity, and mean BMI), in order to characterize the relationship between overweight, obesity, and mean BMI:

log_(BMIc,a,s,t)_ = β_0_ + β_1owc,a,s,t_ + β_2obc,a,s,t_ + β_3sex_ + $\sum_{k-4}^{20} \beta_{k}IA\left.\left[a\right.\right]$ + αs(1 + ow_c,a,s,t_ + ob_c,a,s,t_) + αr(1 + ow_c,a,s,t_ + ob_c,a,s,t_) + αc(1 + ow_c,a,s,t_ + ob_c,a,s,t_) + ϵ_c,a,s,t_

Where owc,a,s,t is the prevalence of overweight in country c, age a, sex s, and year t, obc,a,s,t is the prevalence of obesity in country c, age a, sex s, and year t, sex is a fixed effect on sex,$IA\left[\left.a\right]\right.$ is an indicator variable for age, and αs, αr, and αc are random effects at the super region, region, country, and subnational, respectively. The model was run in Stata 13.

We applied 1,000 draws of the regression coefficients to the 1,000 draws of overweight prevalence and obesity prevalence produced through ST-GPR to estimate 1,000 draws of mean BMI for each country or state, year, age, and sex. This approach ensured that overweight prevalence, obesity prevalence, and mean BMI were correlated at the draw level and uncertainty was propagated.

We used the ensemble distribution approach in which we fit ensemble weights by source and sex, with sourceand sex-specific weights averaged across all sources included to produce the final global weights. The ensemble weights were fit on measured microdata. The final ensemble weights were: exponential = 0.002, gamma = 0.028, inverse gamma = 0.085, log logistic = 0.187, gumbel = 0.220, inverse Weibull = 0.141, Weibull = 0.011, lognormal = 0.058, normal = 0.012, beta = 0.136, mirror gamma = 0.008, and mirror gumbel = 0.113.

One thousand draws of BMI distributions for each location, year, age group, and sex estimated were produced by fitting an ensemble distribution using 1,000 draws of estimated mean BMI, 1,000 draws of estimated standard deviation, and the ensemble weights. Estimated standard deviation was produced by optimizing a standard deviation to fit estimated overweight prevalence draws and estimated obesity prevalence draws. We used Dismod-MR 2.1 to pool effect sizes from included studies and generate a dose-response curve for each of the outcomes associated with high body mass index. The tool enabled us to incorporate random effects across studies and include data with different age ranges. RRs were used universally for all countries and the meta-regression only helped to pool the three major sources and produce RRs with uncertainty and covariance across ages taking into account the uncertainty of the data points.

# Theoretical minimum risk exposure level

For adults (ages 20+), the theoretical minimum risk exposure level (TMREL) of BMI (20-25 kg/m^2^) was determined based on the BMI level that was associated with the lowest risk of all-cause mortality in prospective cohort studies.(23)

# References:

1. Doll R, Payne P, Waterhouse J. Cancer Incidence in Five Continents I. Geneva: Union Internationale Contre le Cancer; 1966;352.

2. Doll R, Muir C, Waterhouse J. Cancer Incidence in Five Continents II. Geneva: Union Internationale Contre le Cancer, Geneva; 1970;354.

3. Waterhouse j, muir c, correa p, powell j. Cancer incidence in five continents III. Lyon: IARC;1976.

4. Waterhouse J, Muir C, Shanmugaratnam K, Powell J. Cancer Incidence in Five Continents IV. Lyon: IARC; 1982;357.

5. Muir c, mack t, powell j, whelan s. Cancer incidence in five continents v. Lyon: IARC;1987..

6. parkin d, muir c, whelan s, gao y, ferlay j, powell j. Cancer incidence in five continents VI. Lyon: IARC;1992.

7. Parkin d, whelan s, ferlay j, raymond l, young j. Cancer incidence in five continents VII. Lyon: IARC;1997.

8. Parkin d, whelan s, ferlay j, teppo l, thomas d. Cancer incidence in five continents VIII. Lyon: IARC;2002.

9. Curado M, Edwards B, Shin H, et al. Cancer Incidence in Five Continents IX. Lyon: IARC;2007. Http://www.iarc.fr/en/publications/pdfs-online/epi/sp160/CI5vol9-A.pdf.

10. Forman D, Bray F, Brewster D, et al. Cancer Incidence in Five Continents X. Http://ci5.iarc.fr.Published 2013.

1. Steliarova-Foucher E, O’Callaghan M, Ferlay J, Masuyer E, Forman D, Comber H, Bray F. European Cancer Observatory: Cancer Incidence, Mortality, Prevalence and Survival in Europe. International Agency for Research on Cancer. http://eco.iarc.fr. Accessed August 10, 2016.
2. Engholm G, Ferlay J, Christensen N, Bray F, Gjerstorff ML, Klint Å et al. NORDCAN – a Nordic tool for cancer information, planning, quality control and research. *ACTA ONCOL*. (2010) 49(5):725-36.doi:10.3109/02841861003782017.PubMed PMID:20491528.
3. Wang H, Naghavi M, Allen C, Barber RM, Bhutta ZA, Carter A et al. Global, regional, and national life expectancy, all-cause mortality, and cause-specific mortality for 249 causes of death, 1980–2015: a systematic analysis for the Global Burden of Disease Study 2015. *The Lancet*. (2016) 388(10053):1459-544.doi:10.1016/S0140-6736(16)31012-1.PubMed PMID:27733281.
4. Naghavi M, Abajobir AA, Abbafati C, Abbas KM, Abd-Allah F, Abera SF et al. Global, regional, and national age-sex specific mortality for 264 causes of death, 1980–2016: a systematic analysis for the Global Burden of Disease Study 2016. *The Lancet*. (2017) 390(10100):1151-210.doi:10.1016/S0140-6736(17)32152-9.PubMed PMID:28919116.
5. Wang H, Dwyer-Lindgren L, Lofgren KT, Rajaratnam JK, Marcus JR, Levin-Rector A et al. Age-specific and sex-specific mortality in 187 countries, 1970–2010: a systematic analysis for the Global Burden of Disease Study 2010. *The Lancet*. (2012) 380(9859):2071-94.doi:10.1016/S0140-6736(12)61719-X.PubMed PMID:23245603.
6. Forouzanfar MH, Afshin A, Alexander LT, Anderson HR, Bhutta ZA, Biryukov S et al. Global, regional, and national comparative risk assessment of 79 behavioural, environmental and occupational, and metabolic risks or clusters of risks, 1990–2015: a systematic analysis for the Global Burden of Disease Study 2015. *The Lancet*. (2016) 388(10053):1659-724.doi:10.1016/S0140-6736(16)31679-8.PubMed PMID:27733284.
7. Barber RM, Fullman N, Sorensen RJD, Bollyky T, McKee M, Nolte E et al. Healthcare Access and Quality Index based on mortality from causes amenable to personal health care in 195 countries and territories, 1990–2015: a novel analysis from the Global Burden of Disease Study 2015. *The Lancet*. (2017) 390(10091):231-66.doi:10.1016/S0140-6736(17)30818-8.PubMed PMID:28528753.
8. Stanaway JD, Flaxman AD, Naghavi M, Fitzmaurice C, Vos T, Abubakar I et al. The global burden of viral hepatitis from 1990 to 2013: findings from the Global Burden of Disease Study 2013. *LANCET*. (2016) 388(10049):1081-8.doi:10.1016/S0140-6736(16)30579-7.PubMed PMID:27394647.
9. Foreman KJ, Lozano R, Lopez AD, Murray CJ. Modeling causes of death: an integrated approach using CODEm. *POPUL HEALTH METR*. (2012) 10:1.doi:10.1186/1478-7954-10-1.PubMed PMID:22226226.
10. Asadzadeh VF, Karim-Kos HE, Janssen-Heijnen ML, Visser O, Verbeek AL, Kiemeney LA. The validity of the mortality to incidence ratio as a proxy for site-specific cancer survival. *EUR J PUBLIC HEALTH*. (2011) 21(5):573-7.doi:10.1093/eurpub/ckq120.PubMed PMID:20813895.
11. Esteve J, Benhamou E, Raymond L. Descriptive Epidemiology. Vol VI. IARC Scientific Publications No.128. Lyon, France: IARC Publications; 1994.
12. Stanaway JD, Afshin A, Gakidou E, Lim SS, Abate D, Abate KH et al. Global, regional, and national comparative risk assessment of 84 behavioural, environmental and occupational, and metabolic risks or clusters of risks for 195 countries and territories, 1990–2017: a systematic analysis for the Global Burden of Disease Study 2017. *The Lancet*. (2018) 392(10159):1923-94.doi:10.1016/S0140-6736(18)32225-6.PubMed PMID:30496105.

23. Global BMC, Di Angelantonio E, Bhupathiraju S, Wormser D, Gao P, Kaptoge S et al. Body-mass index and all-cause mortality: individual-participant-data meta-analysis of 239 prospective studies in four continents. *LANCET*. (2016) 388(10046):776-86.doi:10.1016/S0140-6736(16)30175-1.PubMed PMID:27423262.

# Supplementary Table 1. GATHER：Guidelines for Accurate and Transparent Health Estimates Reporting

| **Item #** | **Checklist item** | **Reported on page #** |
| --- | --- | --- |
| **Objectives and funding** | | |
| **1** | Define the indicator(s), populations (including age, sex, and geographic entities), and time period(s) for which estimates were made. | See eMethods: Definition of indicator |
| **2** | List the funding sources for the work. | Funding sources listed in paper |
| **Data Inputs** | | |
| *For all data inputs from multiple sources that are synthesized as part of the study:* | | |
| **3** | Describe how the data were identified and how the data were accessed. | See eMethods: “Data sources” |
| **4** | Specify the inclusion and exclusion criteria. Identify all ad-hoc exclusions. | See eMethods: “Data sources” |
| **5** | Provide information on all included data sources and their main characteristics. For each data source used, report reference information or contact name/institution, population represented, data collection method, year(s) of data collection, sex and age range, diagnostic criteria or measurement method, and sample size, as relevant. | <http://ghdx.healthdata.org/gbd-2017/data-input-sources> |
| **6** | Identify and describe any categories of input data that have potentially important biases (e.g., based on characteristics listed in item 5). | See eMethods: “Bias of categories of input data” |
| *For data inputs that contribute to the analysis but were not synthesized as part of the study:* | | |
| **7** | Describe and give sources for any other data inputs. | <http://ghdx.healthdata.org/gbd-2017/data-input-sources> |
| *For all data inputs:* | | |
| **8** | Provide all data inputs in a file format from which data can be efficiently extracted (e.g., a spreadsheet rather than a PDF), including all relevant meta-data listed in item 5. For any data inputs that cannot be shared because of ethical or legal reasons, such as third-party ownership, provide a contact name or the name of the institution that retains the right to the data. | <http://ghdx.healthdata.org/gbd-2017/data-input-sources> |
| **Data analysis** | | |
| **9** | Provide a conceptual overview of the data analysis method. A diagram may be helpful. | See Supplementary Figure 1: Flowchart, uterine cancer mortality estimation  See Supplementary Figure 2: Flowchart, uterine cancer incidence, prevalence, YLD^a^ estimation |
| **10** | Provide a detailed description of all steps of the analysis, including mathematical formulae. This description should cover, as relevant, data cleaning, data pre-processing, data adjustments and weighting of data sources, and mathematical or statistical model(s). | See eMethods: “Data analysis” |
| **11** | Describe how candidate models were evaluated and how the final model(s) were selected. | See eMethods “CODEm models”  See Supplementary Table 5: CODEm covariates used, level of covariate, and expected direction of covariate for uterine cancer by cause, sex, and age |
| **12** | Provide the results of an evaluation of model performance, if done, as well as the results of any relevant sensitivity analysis. | See Supplementary Table 6: Results for CODEm model testing |
| **13** | Describe methods for calculating uncertainty of the estimates. State which sources of uncertainty were, and were not, accounted for in the uncertainty analysis. | See eMethods: “Data analysis” |
| **14** | State how analytic or statistical source code used to generate estimates can be accessed. | <http://ghdx.healthdata.org/gbd-2017/code> |
| **Results and Discussion** | | |
| **15** | Provide published estimates in a file format from which data can be efficiently extracted. | GBD 2017 estimates are available online (<https://vizhub.healthdata.org/gbd-compare/>). Web Supplementary Tables specific to the GBD 2017 cancer results are available online (see link in main paper). |
| **16** | Report a quantitative measure of the uncertainty of the estimates (e.g. uncertainty intervals). | Done |
| **17** | Interpret results in light of existing evidence. If updating a previous set of estimates, describe the reasons for changes in estimates. | Supplementary Table 2: Sources for cancer incidence and mortality-to-incidence ratio data by country, year, and registry  Supplementary Table 7: Comparison of GBD 2016 and GBD 2017 covariates used and level of covariates |
| **18** | Discuss limitations of the estimates. Include a discussion of any modelling assumptions or data limitations that affect interpretation of the estimates. | See main manuscript “Limitations” |

**Supplementary Table 2. Sources for cancer incidence and mortality-to-incidence ratio data by country, year, and registry**

| **Location** | **Registry** | **Years available from**  **registry** | **Years used for**  **incidence** | **Years available for**  **MI ratio** | **Years used for MI**  **ratio** |
| --- | --- | --- | --- | --- | --- |
| Algeria | Algiers | 1993‐1997 | 5 | 1993‐1997 | 0 |
| Algeria | Batna | 2000‐2006 | 7 | 2000‐2006 | 0 |
| Algeria | Oran | 2005‐2006 | 2 | 2005‐2006 | 0 |
| Algeria | Setif | 1986‐2007 | 18 | 1986‐2007 | 0 |
| Antilles except  Aruba | Antilles except Aruba | 1973‐1982 | 0 | NA | NA |
| Argentina | Bahia Blanca | 1993‐2007 | 15 | 1993‐2007 | 0 |
| Argentina | Bahia Blanca | 1993‐2007 | 15 | 1993‐2007 | 0 |
| Argentina | Concordia | 1990‐1997 | 10 | 1990‐1997 | 0 |
| Argentina | Cordoba | 2003‐2007 | 4 | 2004‐2007 | 0 |
| Argentina | Mendoza | 2003‐2007 | 5 | 2003‐2007 | 0 |
| Argentina | Tierra del Fuego | 2003‐2007 | 5 | 2003‐2007 | 0 |
| Australia | Capital Territory | 1978‐2007 | 25 | 1983‐2007 | 0 |
| Australia | National Registry | 1982‐2007 | 26 | 1968‐2007 | 26 |
| Australia | New South Wales | 1973‐2007 | 25 | 1983‐2007 | 0 |
| Australia | Northern Territory | 1993‐2007 | 10 | 1998‐2007 | 0 |
| Australia | Queensland | 1982‐2007 | 15 | 1993‐2007 | 0 |
| Australia | South Australia | 1977‐2007 | 31 | 1977‐2007 | 0 |
| Australia | Tasmania | 1978‐2007 | 30 | 1978‐2007 | 0 |
| Australia | Victoria | 1982‐2007 | 25 | 1983‐2007 | 0 |
| Australia | Western Australia | 1982‐2007 | 25 | 1983‐2007 | 0 |
| Austria | National Registry | 1983‐2010 | 31 | 1983‐2010 | 7 |

| **Location** | **Registry** | **Years available from**  **registry** | **Years used for**  **incidence** | **Years available for**  **MI ratio** | **Years used for MI**  **ratio** |
| --- | --- | --- | --- | --- | --- |
| Austria | Salzburg | NA | NA | 1999‐2006 | 0 |
| Austria | Tyrol | 1988‐2007 | 0 | 1988‐2007 | 0 |
| Austria | Vorarlberg | 1993‐2007 | 0 | 1993‐2007 | 0 |
| Bahrain | National Registry | 1998‐2007 | 10 | 1998‐2007 | 0 |
| Belarus | National Registry | 1983‐2007 | 25 | 1983‐2007 | 0 |
| Belgium | Antwerp | 1998‐2002 | 5 | 1998‐2002 | 0 |
| Belgium | Flanders | 1998‐2001 | 4 | 1998‐2001 | 0 |
| Belgium | Flanders except Limburg | 1997‐1998 | 2 | 1997‐1998 | 0 |
| Belgium | Limburg | 1997‐1998 | 2 | 1997‐1998 | 0 |
| Belgium | National Registry | 2003‐2010 | 7 | 2003‐2010 | 0 |
| Bermuda | Bermuda | 1983‐1987 | 5 | 1983‐1987 | 0 |
| Brazil | Aracaju | 1996‐2012 | 17 | 1996‐2012 | 0 |
| Brazil | Barretos | 2008‐2013 | 6 | 2008‐2013 | 0 |
| Brazil | Belem | 1989‐2009 | 17 | 1989‐2009 | 0 |
| Brazil | Belo Horizonte | 2000‐2008 | 9 | 2000‐2008 | 0 |
| Brazil | Brasilia | 1998‐2001 | 4 | 1998‐2001 | 0 |
| Brazil | Campinas | 1991‐2005 | 15 | 1991‐2005 | 0 |
| Brazil | Campo Grande | 2000‐2009 | 6 | 2000‐2009 | 0 |
| Brazil | Cuiaba | 2000‐2007 | 8 | 2000‐2007 | 0 |
| Brazil | Curitiba | 1998‐2010 | 13 | 1998‐2010 | 0 |
| Brazil | Distrito Federal | 1999‐2002 | 4 | 1999‐2002 | 0 |
| Brazil | Espirito Santo | 1997‐2012 | 16 | 1997‐2012 | 0 |
| Brazil | Florianopolis | 2008‐2012 | 5 | 2008‐2012 | 0 |
| **Location** | **Registry** | **Years available from**  **registry** | **Years used for**  **incidence** | **Years available for**  **MI ratio** | **Years used for MI**  **ratio** |
| Brazil | Fortaleza | 1978‐2006 | 25 | 1978‐2006 | 0 |
| Brazil | Goiania | 1988‐2009 | 22 | 1988‐2009 | 0 |
| Brazil | Jahu | 1996‐2013 | 18 | 1996‐2013 | 0 |
| Brazil | Joao Pessoa | 1999‐2010 | 12 | 1999‐2010 | 0 |
| Brazil | Manaus | 1999‐2006 | 8 | 1999‐2006 | 0 |
| Brazil | Mato Grosso Interior | 2001‐2005 | 5 | 2001‐2005 | 0 |
| Brazil | Natal | 1999‐2005 | 7 | 1999‐2005 | 0 |
| Brazil | Palmas | 2000‐2012 | 13 | 2000‐2012 | 0 |
| Brazil | Pocos de Caldas | 2007‐2011 | 5 | 2007‐2011 | 0 |
| Brazil | Porto Alegre | 1979‐2006 | 22 | 1979‐2006 | 0 |
| Brazil | Recife | 1968‐2010 | 17 | 1968‐2010 | 0 |
| Brazil | Roraima | 2003‐2010 | 8 | 2003‐2010 | 0 |
| Brazil | Salvador | 1996‐2005 | 10 | 1996‐2005 | 0 |
| Brazil | Santos | 2008‐2009 | 2 | 2008‐2009 | 0 |
| Brazil | Sao Paulo | 1969‐2011 | 17 | 1969‐2011 | 0 |
| Brazil | Teresina | 2000‐2006 | 7 | 2000‐2006 | 0 |
| Bulgaria | National Registry | 1993‐2010 | 12 | 1993‐2010 | 12 |
| Canada | Alberta | 1960‐2007 | 32 | 1960‐2007 | 0 |
| Canada | British Columbia | 1969‐2007 | 32 | 1969‐2007 | 0 |
| Canada | Manitoba | 1958‐2007 | 32 | 1958‐2007 | 0 |
| Canada | Maritime | 1969‐1987 | 9 | 1969‐1987 | 0 |
| Canada | National Registry | 1978‐2007 | 30 | 1978‐2007 | 0 |
| Canada | New Brunswick | 1962‐2007 | 25 | 1962‐2007 | 0 |
| **Location** | **Registry** | **Years available from**  **registry** | **Years used for**  **incidence** | **Years available for**  **MI ratio** | **Years used for MI**  **ratio** |
| Canada | Newfoundland | 1969‐2002 | 26 | 1969‐2002 | 0 |
| Canada | Newfoundland and Labrador | 1960‐2007 | 9 | 1960‐2007 | 0 |
| Canada | Northwest Territories | 1983‐2007 | 0 | 1983‐2007 | 0 |
| Canada | Northwest Territories and Yukon | 1973‐1987 | 5 | 1973‐1987 | 0 |
| Canada | Nova Scotia | 1978‐2007 | 20 | 1978‐2007 | 0 |
| Canada | Ontario | 1969‐2007 | 23 | 1969‐2007 | 0 |
| Canada | Prince Edward Island | 1978‐2007 | 16 | 1978‐2007 | 0 |
| Canada | Quebec | 1963‐2007 | 13 | 1963‐2007 | 0 |
| Canada | Saskatchewan | 1960‐2007 | 34 | 1960‐2007 | 0 |
| Canada | Yukon | 1983‐2007 | 0 | 1983‐2007 | 0 |
| Chile | Antofagasta | 2003‐2007 | 0 | 2003‐2007 | 0 |
| Chile | Bio Bio | 2003‐2007 | 5 | 2003‐2007 | 0 |
| Chile | Los Rios | 2003‐2007 | 5 | 2003‐2007 | 0 |
| Chile | National Registry | 1959‐1961 | 0 | 1959‐1961 | 0 |
| Chile | Valdivia | 1998‐2007 | 10 | 1998‐2007 | 0 |
| China | Anshan | 1998‐2011 | 11 | 1998‐2011 | 13 |
| China | Baoding | 2009‐2011 | 2 | 2009‐2011 | 2 |
| China | Beijing | 1990‐2011 | 22 | 1990‐2011 | 21 |
| China | Beijing Rural Areas | 2011 | 0 | 2011 | 0 |
| China | Beiliu | 2011 | 1 | 2011 | 1 |
| China | Bengbu | 2011 | 1 | 2011 | 1 |
| China | Benxi | 2003‐2011 | 8 | 2003‐2011 | 8 |
| China | Bijiang District, Tongren | 2011 | 1 | 2011 | 1 |
| **Location** | **Registry** | **Years available from**  **registry** | **Years used for**  **incidence** | **Years available for**  **MI ratio** | **Years used for MI**  **ratio** |
| China | Bincheng District, Binzhou | 2011 | 1 | 2011 | 1 |
| China | Binghai | 2011 | 1 | 2011 | 1 |
| China | Boli | 2011 | 1 | 2011 | 1 |
| China | Cangwu | 2011 | 1 | 2011 | 1 |
| China | Cangzhou | 2011 | 1 | 2011 | 1 |
| China | Changfeng | 2011 | 1 | 2011 | 1 |
| China | Changle | 1990‐2011 | 21 | 1990‐2011 | 21 |
| China | Changning | 2011 | 1 | 2011 | 1 |
| China | Changzhou | 2011 | 1 | 2011 | 1 |
| China | Chifeng | 2009‐2011 | 2 | 2009‐2011 | 2 |
| China | Chuzhou District, Huai'an | 2004‐2007 | 3 | 2004‐2007 | 0 |
| China | Ci County | 1990‐2009 | 23 | 1990‐2009 | 12 |
| China | Cili | 2011 | 1 | 2011 | 1 |
| China | Cixi | 2011 | 1 | 2011 | 1 |
| China | Cixian | 2011 | 1 | 2011 | 1 |
| China | Daan | 2011 | 1 | 2011 | 1 |
| China | Dafeng | 2003‐2011 | 6 | 2003‐2011 | 8 |
| China | Dalian City | 1998‐2011 | 12 | 1998‐2011 | 13 |
| China | Dancheng | 2011 | 1 | 2011 | 1 |
| China | Dandong | 2008‐2011 | 3 | 2008‐2011 | 3 |
| China | Daoli District, Harbin City | 2005‐2011 | 5 | 2005‐2011 | 6 |
| China | Dawukou | 2011 | 1 | 2011 | 1 |
| China | Dazhu | 2011 | 1 | 2011 | 1 |
| **Location** | **Registry** | **Years available from**  **registry** | **Years used for**  **incidence** | **Years available for**  **MI ratio** | **Years used for MI**  **ratio** |
| China | Decheng District, Dezhou | 2011 | 1 | 2011 | 1 |
| China | Dehui | 2009‐2011 | 2 | 2009‐2011 | 2 |
| China | Dingan | 2011 | 1 | 2011 | 1 |
| China | Donggang | 2009‐2011 | 2 | 2009‐2011 | 2 |
| China | Donghai County | 2004‐2011 | 3 | 2004‐2011 | 2 |
| China | Dunhuang | 2011 | 1 | 2011 | 1 |
| China | Faku | 2011 | 1 | 2011 | 1 |
| China | Feicheng | 1998‐2011 | 1998‐2011 | 13 | 13 |
| China | Feidong | 2011 | 1 | 2011 | 1 |
| China | Feixi County | 2009‐2011 | 2 | 2009‐2011 | 2 |
| China | Fusui County | 1990‐2011 | 14 | 1990‐2011 | 8 |
| China | Fuyuan | 2011 | 1 | 2011 | 1 |
| China | Ganyu | 2004‐2011 | 2 | 2004‐2011 | 1 |
| China | Ganzhou District, Zhangye | 2011 | 1 | 2011 | 1 |
| China | Gaomi | 2011 | 1 | 2011 | 1 |
| China | Gaotang | 2011 | 1 | 2011 | 1 |
| China | Gejiu | 2004‐2011 | 3 | 2004‐2011 | 2 |
| China | Gongan | 2011 | 1 | 2011 | 1 |
| China | Guangrao | 2011 | 1 | 2011 | 1 |
| China | Guangzhou City | 2000‐2011 | 9 | 2000‐2011 | 11 |
| China | Guannan | 2011 | 1 | 2011 | 1 |
| China | Guanyun County | 2004‐2011 | 3 | 2004‐2011 | 3 |
| China | Guilin | 2011 | 1 | 2011 | 1 |
| **Location** | **Registry** | **Years available from**  **registry** | **Years used for**  **incidence** | **Years available for**  **MI ratio** | **Years used for MI**  **ratio** |
| China | Guyuan | 2011 | 1 | 2011 | 1 |
| China | Hai'an County | 2009‐2011 | 2 | 2009‐2011 | 2 |
| China | Haimen | 2003‐2011 | 7 | 2003‐2011 | 8 |
| China | Hainan | 2011 | 1 | 2011 | 1 |
| China | Haining | 1998‐2011 | 11 | 1998‐2011 | 13 |
| China | Hangzhou City | 2000‐2011 | 9 | 2000‐2011 | 11 |
| China | Hanjiang District, Putian | 2011 | 1 | 2011 | 1 |
| China | Hefei | 2011 | 1 | 2011 | 1 |
| China | Hengdong County | 2009‐2011 | 2 | 2009‐2011 | 2 |
| China | Hepu | 2011 | 1 | 2011 | 1 |
| China | Hetian | 2011 | 1 | 2011 | 1 |
| China | Hong Kong Special Administrative Region of China | 1974‐2013 | 43 | 1974‐2013 | 8 |
| China | Hongta District, Yuxi | 2011 | 1 | 2011 | 1 |
| China | Hongtong | 2011 | 1 | 2011 | 1 |
| China | Hongze | 2011 | 1 | 2011 | 1 |
| China | Huai'an District, Huai'an | 1998‐2009 | 12 | 1998‐2009 | 12 |
| China | Huaiyin District, Huai'an | 2009‐2011 | 2 | 2009‐2011 | 2 |
| China | Huangdao District, Qingdao | 2011 | 1 | 2011 | 1 |
| China | Huian | 2011 | 1 | 2011 | 1 |
| China | Huichuan District, Zunyi | 2011 | 1 | 2011 | 1 |
| China | Huinong | 2011 | 1 | 2011 | 1 |
| China | Huixian | 2011 | 1 | 2011 | 1 |
| **Location** | **Registry** | **Years available from**  **registry** | **Years used for**  **incidence** | **Years available for**  **MI ratio** | **Years used for MI**  **ratio** |
| China | Huzhu | 2011 | 1 | 2011 | 1 |
| China | Jiangmen | 2011 | 1 | 2011 | 1 |
| China | Jianhu County | 2003‐2011 | 6 | 2003‐2011 | 8 |
| China | Jianou | 2011 | 1 | 2011 | 1 |
| China | Jianping | 2011 | 1 | 2011 | 1 |
| China | Jiashan County | 1990‐2011 | 22 | 1990‐2011 | 21 |
| China | Jiaxing | 2000‐2011 | 10 | 2000‐2011 | 11 |
| China | Jilin | 2011 | 1 | 2011 | 1 |
| China | Jinan | 2011 | 1 | 2011 | 1 |
| China | Jingan | 2011 | 1 | 2011 | 1 |
| China | Jingtai County | 2009‐2011 | 2 | 2009‐2011 | 2 |
| China | Jingxian | 2011 | 1 | 2011 | 1 |
| China | Jingyang | 2011 | 1 | 2011 | 1 |
| China | Jinhu County | 2007‐2011 | 3 | 2007‐2011 | 4 |
| China | Jintan District | 2003‐2011 | 6 | 2003‐2011 | 7 |
| China | Jinzhai | 2011 | 1 | 2011 | 1 |
| China | Jiulongpo District, Chongqing | 2004‐2011 | 3 | 2004‐2011 | 4 |
| China | Jiyuan | 2011 | 1 | 2011 | 1 |
| China | Junan | 2011 | 1 | 2011 | 1 |
| China | Kaihua | 2011 | 1 | 2011 | 1 |
| China | Kailu | 2011 | 1 | 2011 | 1 |
| China | Kaiyang | 2011 | 1 | 2011 | 1 |
| China | Kangping | 2011 | 1 | 2011 | 1 |
| **Location** | **Registry** | **Years available from**  **registry** | **Years used for**  **incidence** | **Years available for**  **MI ratio** | **Years used for MI**  **ratio** |
| China | Kunes County | 2009 | 1 | 2009 | 1 |
| China | Lanping | 2011 | 1 | 2011 | 1 |
| China | Lanzhou | 2011 | 1 | 2011 | 1 |
| China | Leishan | 2011 | 1 | 2011 | 1 |
| China | Leshan | 2011 | 1 | 2011 | 1 |
| China | Lhasa | 2011 | 1 | 2011 | 1 |
| China | Liangzhou District | 2008‐2011 | 3 | 2008‐2011 | 3 |
| China | Lianhu District, Xi'an | 2011 | 1 | 2011 | 1 |
| China | Lianshui | 2011 | 1 | 2011 | 1 |
| China | Lianyungang | 2004‐2011 | 4 | 2004‐2011 | 4 |
| China | Lingbi | 2011 | 1 | 2011 | 1 |
| China | Linhe District, Bayannaoer | 2011 | 1 | 2011 | 1 |
| China | Linqu County | 1998‐2011 | 11 | 1998‐2011 | 13 |
| China | Lintan | 2011 | 1 | 2011 | 1 |
| China | Linxian | 2011 | 1 | 2011 | 1 |
| China | Linzhou | 1990‐2011 | 19 | 1990‐2011 | 21 |
| China | Liuzhou | 2009‐2011 | 2 | 2009‐2011 | 2 |
| China | Liyang | 2011 | 1 | 2011 | 1 |
| China | Longnan | 2011 | 1 | 2011 | 1 |
| China | Longquanyi District, Chengdu | 2011 | 1 | 2011 | 1 |
| China | Lujiang | 2011 | 1 | 2011 | 1 |
| China | Luoshan | 2011 | 1 | 2011 | 1 |
| China | Luoyang | 2011 | 1 | 2011 | 1 |
| **Location** | **Registry** | **Years available from**  **registry** | **Years used for**  **incidence** | **Years available for**  **MI ratio** | **Years used for MI**  **ratio** |
| China | Lushan | 2011 | 1 | 2011 | 1 |
| China | Ma'anshan | 2003‐2011 | 6 | 2003‐2011 | 8 |
| China | Macao Special Administrative Region of China | 2003‐2007 | 5 | 2003‐2007 | 0 |
| China | Macheng | 2011 | 1 | 2011 | 1 |
| China | Maiji District, Tianshui | 2011 | 1 | 2011 | 1 |
| China | Mayang | 2011 | 1 | 2011 | 1 |
| China | Meixian | 2011 | 1 | 2011 | 1 |
| China | Minhe | 2011 | 1 | 2011 | 1 |
| China | Naidong | 2011 | 1 | 2011 | 1 |
| China | Nangang District, Harbin City | 1992‐2011 | 20 | 1992‐2011 | 14 |
| China | Nantong | 2011 | 1 | 2011 | 1 |
| China | Neixiang | 2011 | 1 | 2011 | 1 |
| China | Ningyang | 2011 | 1 | 2011 | 1 |
| China | Nongqishi | 2011 | 1 | 2011 | 1 |
| China | Pengzhou | 2011 | 1 | 2011 | 1 |
| China | Pingluo | 2011 | 1 | 2011 | 1 |
| China | Qianxi County | 2009‐2011 | 2 | 2009‐2011 | 2 |
| China | Qidong County | 1983‐2011 | 35 | 1983‐2011 | 21 |
| China | Qingdao | 2011 | 1 | 2011 | 1 |
| China | Qinghe | 2011 | 1 | 2011 | 1 |
| China | Qingpu | 2011 | 1 | 2011 | 1 |
| China | Qingyang District, Chengdu | 2009‐2011 | 2 | 2009‐2011 | 2 |
| **Location** | **Registry** | **Years available from**  **registry** | **Years used for**  **incidence** | **Years available for**  **MI ratio** | **Years used for MI**  **ratio** |
| China | Qinhuangdao | 2011 | 1 | 2011 | 1 |
| China | Qionghai | 2011 | 1 | 2011 | 1 |
| China | Renhe District, Panzhihua | 2011 | 1 | 2011 | 1 |
| China | Rushan | 2011 | 1 | 2011 | 1 |
| China | Sanmenxia | 2011 | 1 | 2011 | 1 |
| China | Sanya | 2011 | 1 | 2011 | 1 |
| China | Shangdong | 1993‐1997 | 0 | NA | NA |
| China | Shanggao | 2011 | 1 | 2011 | 1 |
| China | Shanghai | 1975‐2011 | 31 | 1975‐2011 | 21 |
| China | Shangyu | 2009‐2011 | 2 | 2009‐2011 | 2 |
| China | Shangzhi | 2009‐2011 | 2 | 2009‐2011 | 2 |
| China | Shangzhou District, Shangluo | 2011 | 1 | 2011 | 1 |
| China | Shapingba District, Chongqing | 2011 | 1 | 2011 | 1 |
| China | Shenqiu | 2011 | 1 | 2011 | 1 |
| China | Shenyang City | 2003‐2011 | 7 | 2003‐2011 | 8 |
| China | Shenzen City | 2004‐2011 | 2 | 2004‐2011 | 1 |
| China | Shexian County | 2003‐2011 | 8 | 2003‐2011 | 8 |
| China | Sheyang County | 2008‐2011 | 3 | 2008‐2011 | 3 |
| China | Shifeng District, Zhuzhou | 2011 | 1 | 2011 | 1 |
| China | Shihezi | 2011 | 1 | 2011 | 1 |
| China | Shouxian | 2011 | 1 | 2011 | 1 |
| China | Shouyang | 2011 | 1 | 2011 | 1 |
| China | Sihui | 1998‐2011 | 11 | 1998‐2011 | 13 |
| **Location** | **Registry** | **Years available from**  **registry** | **Years used for**  **incidence** | **Years available for**  **MI ratio** | **Years used for MI**  **ratio** |
| China | Suzhou | 2006‐2011 | 4 | 2006‐2011 | 5 |
| China | Taixing | 2004‐2011 | 4 | 2004‐2011 | 6 |
| China | Tengchong | 2011 | 1 | 2011 | 1 |
| China | Tengzhou | 2011 | 1 | 2011 | 1 |
| China | Tianchang | 2011 | 1 | 2011 | 1 |
| China | Tianjin | 1981‐2011 | 22 | 1981‐2011 | 16 |
| China | Tianjin Rural Areas | 2011 | 0 | 2011 | 0 |
| China | Tianshan District, Urumqi | 2011 | 1 | 2011 | 1 |
| China | Tong'an District, Xiamen | 2011 | 1 | 2011 | 1 |
| China | Tongguan | 2011 | 1 | 2011 | 1 |
| China | Tonghua | 2011 | 1 | 2011 | 1 |
| China | Tongling | 2008‐2011 | 3 | 2008‐2011 | 3 |
| China | Wanzhouqu District, Chongqing | 2011 | 1 | 2011 | 1 |
| China | Wenshang County | 2009‐2011 | 2 | 2009‐2011 | 2 |
| China | Wuan | 2011 | 1 | 2011 | 1 |
| China | Wufeng | 2011 | 1 | 2011 | 1 |
| China | Wuhan City | 1990‐2011 | 22 | 1990‐2011 | 18 |
| China | Wuhu | 2011 | 1 | 2011 | 1 |
| China | Wuning | 2011 | 1 | 2011 | 1 |
| China | Wuwei | 2004 | 1 | 2004 | 0 |
| China | Wuxi | 2006‐2011 | 2 | 2006‐2011 | 2 |
| China | Xiamen City | 2009‐2011 | 2 | 2009‐2011 | 2 |
| China | Xiang'an District, Xiamen | 2011 | 1 | 2011 | 1 |
| **Location** | **Registry** | **Years available from**  **registry** | **Years used for**  **incidence** | **Years available for**  **MI ratio** | **Years used for MI**  **ratio** |
| China | Xiangfang District, Harbin | 2011 | 1 | 2011 | 1 |
| China | Xianju County | 2009‐2011 | 2 | 2009‐2011 | 2 |
| China | Xilinhaote | 2011 | 1 | 2011 | 1 |
| China | Xinghualing District, Taiyuan | 2011 | 1 | 2011 | 1 |
| China | Xining | 2009‐2011 | 2 | 2009‐2011 | 2 |
| China | Xinyuan | 2011 | 1 | 2011 | 1 |
| China | Xinzhou District, Shangrao | 2011 | 1 | 2011 | 1 |
| China | Xiping County | 2009‐2011 | 2 | 2009‐2011 | 2 |
| China | Xuanwei | 2011 | 1 | 2011 | 1 |
| China | Xuyi County | 2009‐2011 | 2 | 2009‐2011 | 2 |
| China | Xuzhou | 2011 | 1 | 2011 | 1 |
| China | Yakeshi | 2011 | 1 | 2011 | 1 |
| China | Yancheng | 2011 | 1 | 2011 | 1 |
| China | Yancheng District, Luohe | 2011 | 1 | 2011 | 1 |
| China | Yangcheng County | 2003‐2011 | 6 | 2003‐2011 | 8 |
| China | Yangquan | 2009‐2011 | 2 | 2009‐2011 | 2 |
| China | Yangshan | 2011 | 1 | 2011 | 1 |
| China | Yangzhong | 1998‐2011 | 12 | 1998‐2011 | 13 |
| China | Yanji | 2009‐2011 | 2 | 2009‐2011 | 2 |
| China | Yanshi | 2009‐2011 | 2 | 2009‐2011 | 2 |
| China | Yantai | 2011 | 1 | 2011 | 1 |
| China | Yanting County | 1998‐2011 | 10 | 1998‐2011 | 13 |
| China | Yinchuan | 2011 | 1 | 2011 | 1 |
| **Location** | **Registry** | **Years available from**  **registry** | **Years used for**  **incidence** | **Years available for**  **MI ratio** | **Years used for MI**  **ratio** |
| China | Yingdong District, Fuyang | 2011 | 1 | 2011 | 1 |
| China | Yingshan | 2011 | 1 | 2011 | 1 |
| China | Yiyuan | 2011 | 1 | 2011 | 1 |
| China | Yongding | 2011 | 1 | 2011 | 1 |
| China | Yongqiao District, Suzhou | 2011 | 1 | 2011 | 1 |
| China | Yuanhui District, Luohe | 2011 | 1 | 2011 | 1 |
| China | Yuanqu | 2011 | 1 | 2011 | 1 |
| China | Yucheng | 2011 | 1 | 2011 | 1 |
| China | Yuci District, Jinzhong | 2011 | 1 | 2011 | 1 |
| China | Yueyanglou | 2011 | 1 | 2011 | 1 |
| China | Yunmeng County | 2009‐2011 | 2 | 2009‐2011 | 2 |
| China | Yuzhong District, Chongqing | 2011 | 1 | 2011 | 1 |
| China | Yuzhou | 2011 | 1 | 2011 | 1 |
| China | Zanhuang | 2011 | 1 | 2011 | 1 |
| China | Zhanggong District | 2009 | 1 | 2009 | 1 |
| China | Zhanggong District, Ganzhou | 2011 | 1 | 2011 | 1 |
| China | Zhangqiu | 2011 | 1 | 2011 | 1 |
| China | Zhaoling District, Luohe | 2011 | 1 | 2011 | 1 |
| China | Zhaoyuan | 2011 | 1 | 2011 | 1 |
| China | Zhongshan | 1998‐2011 | 12 | 1998‐2011 | 13 |
| China | Zhongshan County | 2004‐2007 | 3 | 2004‐2007 | 0 |
| China | Zhongwei | 2011 | 1 | 2011 | 1 |
| China | Zhongxiang | 2011 | 1 | 2011 | 1 |
| **Location** | **Registry** | **Years available from**  **registry** | **Years used for**  **incidence** | **Years available for**  **MI ratio** | **Years used for MI**  **ratio** |
| China | Zhuanghe | 2009‐2011 | 2 | 2009‐2011 | 2 |
| China | Zhuhai | 2011 | 1 | 2011 | 1 |
| China | Ziliujing District | 2009 | 1 | 2009 | 1 |
| China | Ziliujing District, Zigong | 2011 | 1 | 2011 | 1 |
| China | Zixing | 2011 | 1 | 2011 | 1 |
| China | Zoucheng | 2011 | 1 | 2011 | 1 |
| Colombia | Bucaramanga | 2003‐2007 | 5 | 2003‐2007 | 0 |
| Colombia | Cali | 1962‐2007 | 40 | 1962‐2007 | 0 |
| Colombia | Manizales | 2003‐2007 | 5 | 2003‐2007 | 0 |
| Colombia | National Registry | 2003‐2010 | 0 | 2003‐2010 | 0 |
| Colombia | Pasto | 2003‐2007 | 5 | 2003‐2007 | 0 |
| Costa Rica | National Registry | 1980‐2011 | 33 | 1980‐2013 | 0 |
| Cote d'Ivoire | National Registry | 1995‐1997 | 3 | 1995‐1997 | 0 |
| Croatia | National Registry | 1988‐2010 | 23 | 1988‐2010 | 8 |
| Cuba | National Registry | 1968‐1987 | 11 | 1968‐1986 | 0 |
| Cuba | Villa Clara | 1995‐2007 | 7 | 1995‐2007 | 0 |
| Cyprus | National Registry | 1998‐2007 | 9 | 1998‐2007 | 4 |
| Czech Republic | National Registry | 1983‐2010 | 27 | 1983‐2010 | 5 |
| Denmark | National Registry | 1953‐2014 | 50 | 1953‐2014 | 35 |
| Ecuador | Cuenca | 2003‐2007 | 5 | 2003‐2007 | 0 |
| Ecuador | Quito | 1985‐2007 | 25 | 1985‐2007 | 0 |
| Egypt | Aswan | 2008 | 1 | 2008 | 0 |
| Egypt | Damietta | 2009 | 1 | 2009 | 0 |
| **Location** | **Registry** | **Years available from**  **registry** | **Years used for**  **incidence** | **Years available for**  **MI ratio** | **Years used for MI**  **ratio** |
| Egypt | Gharbiah | 1999‐2007 | 9 | 1999‐2007 | 0 |
| Egypt | Minia | 2009 | 1 | 2009 | 0 |
| Estonia | National Registry | 1968‐2011 | 44 | 1968‐2009 | 5 |
| Fiji | National Registry | 1998‐2010 | 13 | 1998‐2010 | 11 |
| Finland | National Registry | 1953‐2014 | 50 | 1953‐2014 | 35 |
| France | Bas Rhin | 1975‐2007 | 33 | 1975‐2007 | 0 |
| France | Calvados | 1978‐2007 | 30 | 1978‐2007 | 0 |
| France | Calvados Digestive | 1978‐2009 | 6 | 1978‐2009 | 0 |
| France | Cote d'Or | 1980‐2009 | 30 | 1980‐2009 | 0 |
| France | Doubs | 1977‐2009 | 36 | 1977‐2009 | 0 |
| France | Finistere Digestive | 1984‐2009 | 26 | 1984‐2009 | 0 |
| France | Haut Rhin | 1988‐2009 | 21 | 1988‐2009 | 0 |
| France | Herault | 1987‐2009 | 26 | 1987‐2009 | 0 |
| France | Isere | 1979‐2009 | 30 | 1979‐2009 | 0 |
| France | Loire Atlantique | 1991‐2009 | 18 | 1991‐2009 | 0 |
| France | Manche | 1994‐2009 | 19 | 1994‐2009 | 0 |
| France | Nord | 2005‐2009 | 3 | 2005‐2009 | 0 |
| France | Normandy | 2002‐2009 | 8 | 2002‐2009 | 0 |
| France | Somme | 1982‐2009 | 27 | 1982‐2009 | 0 |
| France | Tarn | 1982‐2009 | 27 | 1982‐2009 | 0 |
| France | Vendee | 1998‐2007 | 10 | 1998‐2007 | 0 |
| French  Polynesia | French Polynesia | 1988‐2002 | 0 | NA | NA |
| **Location** | **Registry** | **Years available from**  **registry** | **Years used for**  **incidence** | **Years available for**  **MI ratio** | **Years used for MI**  **ratio** |
| Germany | Bavaria | 2002‐2007 | 0 | 2002‐2007 | 0 |
| Germany | Berlin | 1998‐2007 | 2 | 1998‐2007 | 0 |
| Germany | Brandenburg | 1998‐2007 | 2 | 1998‐2007 | 0 |
| Germany | Bremen | 2000‐2008 | 0 | 2000‐2008 | 0 |
| Germany | Eastern States (former GDR) | 1964‐1989 | 22 | 1964‐1989 | 0 |
| Germany | Free State of Saxony | 1998‐2007 | 2 | 1998‐2007 | 0 |
| Germany | Hamburg | 1969‐2009 | 16 | 1969‐2010 | 2 |
| Germany | Lower Saxony | 2003‐2007 | 0 | 2003‐2007 | 0 |
| Germany | Mecklenburg | 1998‐2007 | 2 | 1998‐2007 | 0 |
| Germany | Mecklenburg‐West Pomerania | 1998‐2007 | 0 | 1998‐2007 | 0 |
| Germany | Munich | 1998‐2007 | 0 | 1998‐2007 | 0 |
| Germany | National Registry | 2000‐2010 | 11 | 2000‐2010 | 11 |
| Germany | North Rhine Westphalia | 1998‐2007 | 2 | 1994‐2007 | 2 |
| Germany | Rhineland Palatinate | 2000‐2007 | 0 | 2000‐2007 | 0 |
| Germany | Saarland | 1968‐2007 | 30 | 1968‐2007 | 30 |
| Germany | Saxony‐Anhalt | 1998‐2007 | 2 | 1998‐2007 | 0 |
| Germany | Schleswig Holstein | 1998‐2007 | 2 | 1998‐2007 | 2 |
| Germany | Thuringen | 1998‐2007 | 2 | 1998‐2007 | 0 |
| Germany | Westphalia | 1998‐2007 | 0 | 1998‐2007 | 0 |
| Greece | National Registry | 1990‐1991 | 0 | 1990‐1991 | 0 |
| Greenland | Greenland | 1980‐2014 | 35 | 1980‐2014 | 32 |
| Grenada | St. George's Central Hospital | 1996‐2000 | 5 | 1996‐2000 | 0 |
| Guinea | Conakry | 1992‐1995 | 4 | 1992‐1995 | 0 |
| **Location** | **Registry** | **Years available from**  **registry** | **Years used for**  **incidence** | **Years available for**  **MI ratio** | **Years used for MI**  **ratio** |
| Hungary | County Szabolcs‐Szatmar | 1962‐1987 | 18 | 1962‐1987 | 0 |
| Hungary | County Vas | 1962‐1987 | 20 | 1962‐1987 | 0 |
| Hungary | Miskolc | 1962‐1966 | 0 | 1962‐1966 | 0 |
| Hungary | National Registry | 2001‐2011 | 11 | 2001‐2011 | 0 |
| Iceland | National Registry | 1955‐2014 | 50 | 1955‐2014 | 35 |
| India | Ahmedabad | 1983‐2005 | 12 | NA | NA |
| India | Ahmedabad Rural | 2006‐2010 | 5 | NA | NA |
| India | Ahmedabad Urban | 2006‐2013 | 7 | NA | NA |
| India | Aizawl | 2005‐2014 | 5 | NA | NA |
| India | Aurangabad | 2005‐2014 | 9 | NA | NA |
| India | Bangalore | 1982‐2012 | 24 | NA | NA |
| India | Barshi Expanded | 2009‐2012 | 2 | NA | NA |
| India | Barshi Rural | 1988‐2014 | 20 | NA | NA |
| India | Bhopal | 2004‐2013 | 11 | NA | NA |
| India | Cachar | 2007‐2014 | 7 | NA | NA |
| India | Chandigarh Union Territory | 2013 | 1 | NA | NA |
| India | Chennai | 1982‐2013 | 34 | NA | NA |
| India | Delhi | 1993‐2012 | 21 | NA | NA |
| India | Dibrugarh | 2005‐2014 | 11 | NA | NA |
| India | Dindigul Ambilikkai | 2003‐2013 | 7 | NA | NA |
| India | Imphal | 2005‐2014 | 5 | NA | NA |
| India | Kamrup Urban | 2005‐2014 | 11 | NA | NA |
| India | Karunagappally | 1991‐2007 | 17 | NA | NA |
| **Location** | **Registry** | **Years available from**  **registry** | **Years used for**  **incidence** | **Years available for**  **MI ratio** | **Years used for MI**  **ratio** |
| India | Kolkata | 2005‐2012 | 6 | NA | NA |
| India | Kollam | 2006‐2014 | 8 | NA | NA |
| India | Manipur | 2006‐2010 | 5 | NA | NA |
| India | Manipur Excl Imphal West | 2009‐2014 | 5 | NA | NA |
| India | Mansa District | 2013 | 1 | NA | NA |
| India | Meghalaya | 2010‐2014 | 5 | NA | NA |
| India | Mizoram | 2003‐2010 | 10 | NA | NA |
| India | Mizoram Excl Aizawl | 2005‐2014 | 5 | NA | NA |
| India | Mumbai | 1964‐2012 | 50 | NA | NA |
| India | Nagaland | 2010‐2014 | 4 | NA | NA |
| India | Nagpur | 1980‐2013 | 20 | NA | NA |
| India | Naharlagun Excl Papum Pare | 2012‐2014 | 3 | NA | NA |
| India | Papum Pare | 2012‐2014 | 3 | NA | NA |
| India | Pasighat | 2012‐2014 | 3 | NA | NA |
| India | Patiala District | 2012‐2014 | 3 | NA | NA |
| India | Pune | 1973‐2013 | 32 | NA | NA |
| India | S.A.S Nagar District | 2013 | 1 | NA | NA |
| India | Sangrur District | 2013 | 1 | NA | NA |
| India | Sikkim | 2003‐2014 | 14 | NA | NA |
| India | Silchar | 2005‐2006 | 2 | NA | NA |
| India | Tamil Nadu | 2012‐2013 | 2 | NA | NA |
| India | Tripura | 2010‐2014 | 4 | NA | NA |
| India | Trivandrum | 1991‐2014 | 21 | NA | NA |
| **Location** | **Registry** | **Years available from**  **registry** | **Years used for**  **incidence** | **Years available for**  **MI ratio** | **Years used for MI**  **ratio** |
| India | Wardha | 2010‐2014 | 5 | NA | NA |
| Iran | Ardabil | 1985‐2008 | 8 | 1985‐2008 | 0 |
| Iran | Golestan | 1996‐2007 | 8 | 1996‐2007 | 0 |
| Iran | National Registry | 2003‐2007 | 8 | 2003‐2007 | 0 |
| Iraq | National Registry | 2007‐2011 | 3 | 2007‐2011 | 0 |
| Ireland | National Registry | 1994‐2010 | 16 | 1994‐2010 | 0 |
| Ireland | Southern Ireland | 1980‐1992 | 12 | 1980‐1992 | 0 |
| Israel | National Registry | 1960‐2010 | 13 | 1960‐2010 | 0 |
| Italy | Alto Adige | 2003‐2006 | 4 | 2003‐2006 | 0 |
| Italy | Biella | 1995‐2007 | 13 | 1995‐2007 | 0 |
| Italy | Brescia | 1999‐2007 | 7 | 1999‐2006 | 0 |
| Italy | Catania and Messina | 2003‐2005 | 3 | 2003‐2005 | 0 |
| Italy | Catanzaro | 2003‐2007 | 5 | 2003‐2007 | 0 |
| Italy | Como | 2003‐2007 | 5 | 2003‐2007 | 0 |
| Italy | Ferrara | 1991‐2007 | 17 | 1991‐2007 | 0 |
| Italy | Florence | 1985‐2002 | 18 | 1985‐2002 | 0 |
| Italy | Florence and Prato | 1985‐2007 | 21 | 1985‐2005 | 0 |
| Italy | Friuli Venezia Giulia | 2003‐2007 | 5 | 2003‐2007 | 0 |
| Italy | Genoa | 1986‐2007 | 18 | 1986‐2006 | 0 |
| Italy | Latina | 1983‐2007 | 12 | 1983‐2007 | 0 |
| Italy | Lecco | 2003‐2007 | 5 | 2003‐2007 | 0 |
| Italy | Macerata | 1991‐2000 | 10 | 1991‐2000 | 0 |
| Italy | Mantua | 2003‐2007 | 3 | 2003‐2005 | 0 |
| **Location** | **Registry** | **Years available from**  **registry** | **Years used for**  **incidence** | **Years available for**  **MI ratio** | **Years used for MI**  **ratio** |
| Italy | Milan | 1999‐2007 | 8 | 1999‐2006 | 0 |
| Italy | Modena | 1988‐2007 | 23 | 1988‐2007 | 0 |
| Italy | Naples | 1998‐2007 | 10 | 1998‐2007 | 0 |
| Italy | National Registry | 2006‐2009 | 4 | 2006‐2009 | 4 |
| Italy | North East Italy | 1995‐2002 | 8 | 1995‐2002 | 0 |
| Italy | Nuoro | 2003‐2007 | 5 | 2003‐2007 | 0 |
| Italy | Palermo | 2003‐2007 | 4 | 2003‐2006 | 0 |
| Italy | Parma | 1978‐2007 | 30 | 1978‐2007 | 0 |
| Italy | Ragusa | 1978‐2007 | 31 | 1981‐2007 | 0 |
| Italy | Reggio Emilia | 1998‐2007 | 10 | 1998‐2007 | 0 |
| Italy | Romanga | 1985‐2007 | 22 | 1986‐2007 | 0 |
| Italy | Salerno | 1998‐2007 | 9 | 1998‐2007 | 0 |
| Italy | Sassari | 1993‐2007 | 15 | 1993‐2007 | 0 |
| Italy | Sondrio | 1998‐2007 | 14 | 1998‐2007 | 0 |
| Italy | South Lombard | 2003‐2005 | 3 | 2003‐2005 | 0 |
| Italy | South Tyrol | 2003‐2007 | 0 | NA | NA |
| Italy | Syracuse | 1999‐2007 | 9 | 1999‐2007 | 0 |
| Italy | Torino | 1984‐2007 | 23 | 1985‐2007 | 0 |
| Italy | Trapani | 2003‐2006 | 4 | 2003‐2006 | 0 |
| Italy | Trento | 2003‐2006 | 4 | 2003‐2006 | 0 |
| Italy | Trieste | 1983‐1992 | 6 | 1984‐1992 | 0 |
| Italy | Umbria | 1994‐2007 | 13 | 1994‐2007 | 1 |
| Italy | Varese | 1976‐2007 | 32 | 1976‐2007 | 0 |
| **Location** | **Registry** | **Years available from**  **registry** | **Years used for**  **incidence** | **Years available for**  **MI ratio** | **Years used for MI**  **ratio** |
| Italy | Veneto | 1988‐2007 | 17 | 1988‐2006 | 0 |
| Jamaica | National Registry | 1958‐2007 | 25 | 1958‐2007 | 0 |
| Japan | Aichi | 1998‐2007 | 11 | 1998‐2007 | 1 |
| Japan | Fukui | 1998‐2007 | 10 | 1998‐2007 | 0 |
| Japan | Fukuoka | 1974‐1975 | 2 | 1974‐1975 | 0 |
| Japan | Hiroshima | 1978‐2007 | 28 | 1978‐2007 | 0 |
| Japan | Miyagi | 1959‐2007 | 35 | 1959‐2007 | 0 |
| Japan | Nagasaki | 1973‐2007 | 35 | 1973‐2007 | 0 |
| Japan | National Registry | 1975‐2010 | 36 | 1958‐2013 | 36 |
| Japan | Niigata | 2003‐2007 | 5 | 2003‐2007 | 0 |
| Japan | Okayama | 1966‐1969 | 0 | 1966‐1969 | 0 |
| Japan | Osaka | 1963‐2007 | 38 | 1963‐2007 | 0 |
| Japan | Saga | 1984‐2007 | 18 | 1984‐2007 | 0 |
| Japan | Yamagata | 1983‐2002 | 23 | 1983‐2002 | 0 |
| Jordan | National Registry | 2001‐2008 | 8 | 2001‐2008 | 0 |
| Kenya | Nairobi | 2000‐2002 | 3 | 2000‐2002 | 0 |
| Kuwait | National Registry | 1979‐2007 | 28 | 1979‐2007 | 0 |
| Kyrgyzstan | National Registry | 1986‐1987 | 2 | 1986‐1987 | 0 |
| La Martinique | La Martinique | 1981‐2002 | 0 | NA | NA |
| La Reunion | La Reunion | 1988‐1994 | 0 | NA | NA |
| Latvia | National Registry | 1983‐2007 | 24 | 1983‐2007 | 5 |
| Lebanon | National Registry | 1998‐2007 | 4 | 1998‐2007 | 0 |
| Libya | Benghazi | 2003‐2005 | 3 | 2003‐2005 | 0 |
| **Location** | **Registry** | **Years available from**  **registry** | **Years used for**  **incidence** | **Years available for**  **MI ratio** | **Years used for MI**  **ratio** |
| Lithuania | National Registry | 1978‐2011 | 33 | 1978‐2011 | 2 |
| Malawi | Blantyre | 1994-2007 | 10 | 1994-2007 | 0 |
| Malaysia | National Registry | 2003 | 1 | 2003 | 0 |
| Malaysia | Penang | 1998-2007 | 9 | 1998-2007 | 0 |
| Malaysia | Sarawak | 1998-2002 | 5 | 1998-2002 | 0 |
| Mali | Bamako | 1987-1996 | 11 | 1987-1996 | 0 |
| Malta | National Registry | 1969-2010 | 27 | 1969-2010 | 15 |
| Mongolia | National Registry | 2003-2007 | 0 | 2003-2007 | 0 |
| Morocco | Greater Casablanca | 2004 | 1 | 2004 | 0 |
| Mozambique | Lourenco Marques | 1956-1960 | 0 | 1956-1960 | 0 |
| Namibia | National Registry | 2000-2009 | 10 | 2000-2009 | 0 |
| Netherlands | Eindhoven | 1973-2007 | 27 | 1973-2007 | 0 |
| Netherlands | Maastricht | 1986-2002 | 3 | 1986-2002 | 0 |
| Netherlands | National Registry | 1989-2015 | 19 | 1989-2007 | 0 |
| Netherlands | Three Provinces | 1960-1962 | 0 | 1960-1962 | 0 |
| New Zealand | National Registry | 1968-2014 | 32 | 1983-2014 | 5 |
| Nigeria | Calabar | 2009-2013 | 5 | 2009-2013 | 0 |
| Nigeria | Ibadan | 1960-1969 | 0 | 1960-1969 | 0 |
| Nigeria | Midwestern Nigeria | NA | NA | 2008-2009 | 0 |
| Norway | National Registry | 1953-2014 | 50 | 1953-2014 | 35 |
| Oman | National Registry | 1993-2012 | 20 | 1993-2012 | 0 |
| Pakistan | South Karachi | 1995-2002 | 8 | 1995-2002 | 0 |
| Palestine | West Bank | 2010-2011 | 2 | 2010-2011 | 0 |
| **Location** | **Registry** | **Years available from**  **registry** | **Years used for**  **incidence** | **Years available for**  **MI ratio** | **Years used for MI**  **ratio** |
| Panama | National Registry | 1988-2011 | 23 | 1988-2011 | 12 |
| Paraguay | Asuncion Region | 1988-1989 | 2 | 1988-1989 | 0 |
| Peru | Lima | 1990-1991 | 2 | 1990-1991 | 0 |
| Peru | Trujillo | 1984-2002 | 19 | 1984-2002 | 1 |
| Philippines | Manila | 1983-2007 | 25 | 1983-2007 | 0 |
| Philippines | Rizal | 1978-2007 | 20 | 1978-2007 | 0 |
| Poland | Cieszyn | 1968-1977 | 5 | 1973-1977 | 0 |
| Poland | Cieszyn and Nowy Sacz | 1968-1972 | 5 | 1968-1972 | 0 |
| Poland | Cracow | 1968-2006 | 26 | 1968-2006 | 0 |
| Poland | Cracow City and District | 1965-1972 | 5 | 1965-1972 | 0 |
| Poland | Four Rural Areas | 1965-1966 | 0 | 1965-1966 | 0 |
| Poland | Katowice | 1965-1977 | 5 | 1965-1974 | 0 |
| Poland | Kielce | 1988-2007 | 13 | 1988-2007 | 0 |
| Poland | Lower Silesia | 1984-2007 | 14 | 1984-2007 | 0 |
| Poland | National Registry | 1999-2011 | 13 | 1999-2011 | 13 |
| Poland | Nowy Sacz | 1973-1986 | 13 | 1973-1986 | 0 |
| Poland | Opole | 1985-1987 | 3 | 1985-1987 | 0 |
| Poland | Rzeszow | 2003-2007 | 0 | 2003-2007 | 0 |
| Poland | Warsaw | 1988-2002 | 11 | 1988-2002 | 0 |
| Poland | Warsaw Rural | 1968-1987 | 15 | 1968-1987 | 0 |
| Poland | Warsaw Urban | 1965-2002 | 27 | 1965-2002 | 0 |
| Portugal | Azores | 1997-2011 | 14 | 1981-2012 | 15 |
| Portugal | Centre | 2003-2007 | 5 | 2003-2007 | 5 |
| **Location** | **Registry** | **Years available from**  **registry** | **Years used for**  **incidence** | **Years available for**  **MI ratio** | **Years used for MI**  **ratio** |
| Portugal | North Portugal | 2000-2006 | 7 | 2000-2006 | 7 |
| Portugal | Porto | 1998-2002 | 5 | 1998-2002 | 0 |
| Portugal | South Portugal | 1998-2007 | 10 | 1998-2007 | 0 |
| Portugal | Vila Nova de Gaia | 1983-1997 | 10 | 1983-1997 | 0 |
| Puerto Rico | National Registry | NA | 26 | NA | NA |
| Qatar | National Registry | 2003-2007 | 5 | 2003-2007 | 0 |
| Romania | Banat Region | 1967 | 0 | 1967 | 0 |
| Romania | Cluj | 2007 | 1 | 2007 | 1 |
| Romania | County Cluj | 1974-1987 | 14 | 1974-1987 | 0 |
| Romania | County Timis | 1970-1972 | 3 | 1970-1972 | 0 |
| Romania | Timisoara | 2008 | 1 | 2008 | 0 |
| Russia | St Petersburg | 1983-2007 | 19 | 1983-2007 | 0 |
| Samoa | National Registry | 1980-1988 | 9 | 1980-1988 | 0 |
| Saudi Arabia | National Registry | 1994-2012 | 10 | 1994-2012 | 0 |
| Saudi Arabia | Riyadh | 2003-2007 | 5 | 2003-2007 | 0 |
| Senegal | Dakar | 1969-1974 | 6 | 1969-1974 | 0 |
| Serbia | Central Serbia | 2003-2007 | 4 | 2003-2007 | 5 |
| Serbia | National Registry | 1999-2002 | 4 | 1999-2002 | 0 |
| Serbia | Vojvodina | 1988-1997 | 10 | 1988-1997 | 0 |
| Seychelles | National Registry | 2009-2011 | 3 | 2009-2011 | 0 |
| Singapore | National Registry | 1950-2008 | 44 | 1950-2008 | 0 |
| Slovakia | National Registry | 1968-2007 | 42 | 1968-2007 | 30 |
| Slovenia | National Registry | 1956-2010 | 49 | 1956-2010 | 8 |
| **Location** | **Registry** | **Years available from**  **registry** | **Years used for**  **incidence** | **Years available for**  **MI ratio** | **Years used for MI**  **ratio** |
| South Africa | Johannesburg, Bantu | 1953-1955 | 0 | 1953-1955 | 0 |
| South Africa | National Registry | 2003-2011 | 4 | 2003-2011 | 0 |
| South Africa | PROMEC | 1998-2007 | 10 | 1998-2007 | 0 |
| South Korea | Busan | 1996-2007 | 2 | 1996-2007 | 0 |
| South Korea | Daegu | 1997-2007 | 2 | 1997-2007 | 0 |
| South Korea | Daejeon | 1998-2007 | 0 | 1998-2007 | 0 |
| South Korea | Gwangju | 1998-2007 | 0 | 1998-2007 | 0 |
| South Korea | Incheon | 1998-2007 | 0 | 1998-2007 | 0 |
| South Korea | Jejudo | 2000-2007 | 3 | 2000-2007 | 0 |
| South Korea | Kangwha County | 1986-1997 | 12 | 1986-1997 | 0 |
| South Korea | National Registry | 1999-2012 | 12 | 1999-2012 | 0 |
| South Korea | Seoul | 1993-2007 | 5 | 1993-2007 | 0 |
| South Korea | Ulsan | 1999-2007 | 0 | 1999-2007 | 0 |
| Spain | Albacete | 1991-2007 | 16 | 1991-2007 | 6 |
| Spain | Asturias | 1988-2007 | 17 | 1988-2007 | 5 |
| Spain | Balears | 1988-2005 | 18 | 1988-2005 | 0 |
| Spain | Basque Country | 1986-2007 | 20 | 1986-2008 | 4 |
| Spain | Canary Islands | 1993-2006 | 10 | 1993-2006 | 0 |
| Spain | Ciudad Real | 2004-2007 | 4 | 2004-2007 | 0 |
| Spain | Cuenca | 1993-2007 | 15 | 1993-2007 | 5 |
| Spain | Girona | 1980-2007 | 27 | 1980-2008 | 23 |
| Spain | Granada | 1985-2007 | 22 | 1985-2007 | 23 |
| Spain | La Rioja | 1993-2007 | 12 | 1993-2007 | 13 |
| **Location** | **Registry** | **Years available from**  **registry** | **Years used for**  **incidence** | **Years available for**  **MI ratio** | **Years used for MI**  **ratio** |
| Spain | Mallorca | 1988-2007 | 14 | 1988-2007 | 0 |
| Spain | Murcia | 1983-2007 | 24 | 1983-2007 | 25 |
| Spain | Navarra | 1973-2007 | 32 | 1973-2008 | 31 |
| Spain | Tarragona | 1980-2007 | 25 | 1980-2007 | 24 |
| Spain | Zaragoza | 1968-2000 | 33 | 1968-2000 | 0 |
| Sri Lanka | National Registry | 2001-2005 | 6 | 2001-2005 | 0 |
| Sweden | National Registry | 1958-2014 | 50 | 1958-2014 | 35 |
| Sweden | Stockholm | 1990-2010 | 21 | 1990-2010 | 0 |
| Sweden | Sweden except Stockholm | 1990-2010 | 21 | 1990-2010 | 0 |
| Switzerland | Basel | 1981-2007 | 7 | 1981-2007 | 0 |
| Switzerland | Geneva | 1970-2008 | 19 | 1970-2008 | 29 |
| Switzerland | Graubunden | 1989-1997 | 0 | 1989-1997 | 0 |
| Switzerland | Graubunden and Glarus | 1989-2009 | 0 | 1989-2009 | 20 |
| Switzerland | National Registry | 1986-2013 | 25 | 1986-2013 | 0 |
| Switzerland | Neuchatel | 1974-2007 | 13 | 1974-2008 | 5 |
| Switzerland | St Gallen ‐ Appenzell | 1980-2009 | 9 | 1980-2009 | 29 |
| Switzerland | Ticino | 1996-2007 | 0 | 1996-2007 | 0 |
| Switzerland | Valais | 1989-2007 | 0 | 1989-2007 | 0 |
| Switzerland | Vaud | 1975-2007 | 13 | 1975-2008 | 5 |
| Switzerland | Zurich | 1980-2009 | 9 | 1980-2009 | 0 |
| Taiwan | National Registry | 1980-2007 | 28 | 1980-2007 | 28 |
| Thailand | Bangkok | 1995-2007 | 11 | 1995-2007 | 0 |
| Thailand | Chiang Mai | 1983-2007 | 29 | 1983-2007 | 0 |
| **Location** | **Registry** | **Years available from**  **registry** | **Years used for**  **incidence** | **Years available for**  **MI ratio** | **Years used for MI**  **ratio** |
| Thailand | Chonburi | 2001-2007 | 8 | 2001-2007 | 0 |
| Thailand | Khon Kaen | 1988-2007 | 19 | 1988-2007 | 0 |
| Thailand | Lampang | 1993-2007 | 18 | 1993-2007 | 0 |
| Thailand | Lop Buri | 2001-2003 | 3 | 2001-2003 | 0 |
| Thailand | Nakhon Phanom | 2001-2003 | 3 | 2001-2003 | 0 |
| Thailand | Prachuap Khiri | 2001-2003 | 3 | 2001-2003 | 0 |
| Thailand | Rayong | 2001-2003 | 3 | 2001-2003 | 0 |
| Thailand | Songkhla | 1993-2007 | 16 | 1993-2007 | 0 |
| Thailand | Surat Thani | 2001-2003 | 3 | 2001-2003 | 0 |
| Thailand | Ubon Ratchathani | 2001-2003 | 3 | 2001-2003 | 0 |
| Thailand | Udon Thani | 2001-2003 | 3 | 2001-2003 | 0 |
| The Gambia | National Registry | 1987-1998 | 15 | 1987-1998 | 0 |
| Trinidad and Tobago | National Registry | 1995-2006 | 12 | 1995-2006 | 12 |
| Tunisia | Centre Sousse | 1998-2002 | 5 | 1998-2002 | 0 |
| Tunisia | North Tunisia | 2003-2005 | 3 | 2003-2005 | 0 |
| Turkey | Ankara | 2002-2005 | 4 | 2002-2005 | 0 |
| Turkey | Antalya | 1998-2008 | 13 | 1998-2008 | 0 |
| Turkey | Edirne | 2002-2007 | 6 | 2002-2007 | 0 |
| Turkey | Eight Provinces | 2006-2007 | 2 | 2006-2007 | 0 |
| Turkey | Erzurum | 2002-2005 | 4 | 2002-2005 | 0 |
| Turkey | Eskisehir | 2002-2005 | 4 | 2002-2005 | 0 |
| Turkey | Izmir | 1998-2008 | 14 | 1998-2008 | 0 |
| **Location** | **Registry** | **Years available from**  **registry** | **Years used for**  **incidence** | **Years available for**  **MI ratio** | **Years used for MI**  **ratio** |
| Turkey | Nine Provinces | 2008 | 1 | 2008 | 0 |
| Turkey | Samsun | 2002-2005 | 4 | 2002-2005 | 0 |
| Turkey | Trabzon | 2002-2007 | 5 | 2002-2007 | 0 |
| Uganda | Kampala | 1954-2013 | 24 | 1954-2013 | 0 |
| Ukraine | National Registry | 2000-2010 | 7 | 2000-2010 | 4 |
| United Kingdom | Aryshire | 1970-1972 | 3 | 1970-1972 | 0 |
| United Kingdom | East Anglia | 1988-1997 | 0 | 1988-1997 | 0 |
| United Kingdom | East Midlands | 1981-2014 | 34 | 1981-2014 | 20 |
| United Kingdom | East Scotland | 1973-1987 | 5 | 1973-1987 | 0 |
| United Kingdom | East of England | 1981-2014 | 33 | 1981-2014 | 20 |
| United Kingdom | England | 1993-2007 | 10 | 1993-2007 | 0 |
| United Kingdom | England and Wales | 1979-1990 | 0 | 1979-1990 | 0 |
| United Kingdom | Greater London | 1981-2014 | 34 | 1981-2014 | 20 |
| United Kingdom | Merseyside and Cheshire | 1959-2002 | 11 | 1959-2002 | 0 |
| United Kingdom | North East England | 1981-2014 | 34 | 1981-2014 | 20 |
| United Kingdom | North East Scotland | 1973-1987 | 5 | 1973-1987 | 0 |
| United Kingdom | North Scotland | 1973-1987 | 5 | 1973-1987 | 0 |
| United Kingdom | North West England | 1973-2014 | 40 | 1973-2014 | 20 |
| United Kingdom | Northern England and Yorkshire | 1998-2007 | 0 | 1998-2007 | 0 |
| United Kingdom | Northern Ireland | 1993-2011 | 18 | 1993-2011 | 0 |
| United Kingdom | Oxford | 1963-2007 | 18 | 1963-2007 | 0 |
| United Kingdom | Scotland | 1963-2014 | 40 | 1963-2014 | 25 |
| United Kingdom | South East England | 1981-2014 | 34 | 1981-2014 | 20 |
| **Location** | **Registry** | **Years available from**  **registry** | **Years used for**  **incidence** | **Years available for**  **MI ratio** | **Years used for MI**  **ratio** |
| United Kingdom | South East Scotland | 1973-1987 | 5 | 1973-1987 | 0 |
| United Kingdom | South Thames | 1960-2007 | 20 | 1960-1997 | 0 |
| United Kingdom | South West England | 1960-2014 | 37 | 1960-2014 | 20 |
| United Kingdom | Thames | 1998-2007 | 0 | 1991-2007 | 0 |
| United Kingdom | Trent | 1963-2007 | 3 | 1963-2007 | 0 |
| United Kingdom | Wales | 1981-2011 | 31 | 1981-2011 | 20 |
| United Kingdom | Wessex | 1988-1992 | 0 | 1988-1992 | 0 |
| United Kingdom | West Midlands | 1960-2014 | 44 | 1960-2014 | 20 |
| United Kingdom | West Scotland | 1975-1992 | 0 | 1975-1992 | 0 |
| United Kingdom | Yorkshire | 1983-2002 | 7 | 1983-2002 | 0 |
| United Kingdom | Yorkshire and the Humber | 1981-2014 | 34 | 1981-2014 | 20 |
| United States | Alabama | 1998-2011 | 10 | 1998-2007 | 0 |
| United States | Alameda County | 1969-1987 | 5 | 1983-1987 | 0 |
| United States | Alaska | 1998-2011 | 10 | 1998-2007 | 0 |
| United States | Alaska Natives | 1992-2013 | 0 | NA | NA |
| United States | Arizona | 1998-2011 | 10 | 1998-2007 | 0 |
| United States | Arkansas | 2001-2011 | 5 | 2003-2007 | 0 |
| United States | Atlanta | 1973-2013 | 39 | 1973-2013 | 36 |
| United States | California | 1998-2011 | 10 | 1998-2007 | 0 |
| United States | California except SF,SJ-M, & LA | 2000-2013 | 14 | 2000-2013 | 9 |
| United States | Central California | 1988-1992 | 5 | 1988-1992 | 0 |
| United States | Colorado | 1998-2011 | 10 | 1998-2007 | 0 |
| United States | Connecticut | 1960-2013 | 41 | 1960-2013 | 37 |
| **Location** | **Registry** | **Years available from**  **registry** | **Years used for**  **incidence** | **Years available for**  **MI ratio** | **Years used for MI**  **ratio** |
| United States | Delaware | 1999-2011 | 5 | 2003-2007 | 0 |
| United States | Detroit | 1969-2013 | 41 | 1973-2013 | 37 |
| United States | District of Columbia | 1998-2011 | 5 | 1998-2002 | 0 |
| United States | El Paso | 1960-1970 | 0 | 1960-1970 | 0 |
| United States | Florida | 1998-2011 | 10 | 1998-2007 | 0 |
| United States | Georgia | 1998-2010 | 10 | 1998-2007 | 0 |
| United States | Greater Georgia | 1973-2013 | 14 | 1973-2013 | 13 |
| United States | Hawaii | 1960-2013 | 46 | 1960-2013 | 37 |
| United States | Idaho | 1998-2011 | 10 | 1998-2007 | 0 |
| United States | Illinois | 1998-2011 | 10 | 1998-2007 | 0 |
| United States | Indiana | 1998-2011 | 10 | 1998-2007 | 0 |
| United States | Iowa | 1969-2013 | 41 | 1973-2013 | 37 |
| United States | Kansas | 1999-2011 | 0 | NA | NA |
| United States | Kentucky | 1973-2013 | 19 | 1973-2013 | 19 |
| United States | Los Angeles | 1973-2013 | 41 | 1973-2013 | 24 |
| United States | Louisiana | 1973-2013 | 29 | 1973-2013 | 18 |
| United States | Maine | 1998-2011 | 10 | 1998-2007 | 0 |
| United States | Maryland | 1999-2011 | 0 | NA | NA |
| United States | Massachusetts | 1998-2011 | 10 | 1998-2007 | 0 |
| United States | Michigan | 1998-2011 | 10 | 1998-2007 | 0 |
| United States | Minnesota | 1998-2011 | 0 | NA | NA |
| United States | Mississippi | 2003-2011 | 5 | 2003-2007 | 0 |
| United States | Missouri | 1998-2011 | 10 | 1998-2007 | 0 |
| **Location** | **Registry** | **Years available from**  **registry** | **Years used for**  **incidence** | **Years available for**  **MI ratio** | **Years used for MI**  **ratio** |
| United States | Montana | 1998-2011 | 10 | 1998-2007 | 0 |
| United States | National Registry | 1962-2007 | 0 | 1962-2007 | 0 |
| United States | Nebraska | 1999-2011 | 5 | 2003-2007 | 0 |
| United States | Nevada | 1959-2010 | 0 | 1959-1966 | 0 |
| United States | New Hampshire | 1999-2011 | 5 | 2003-2007 | 0 |
| United States | New Jersey | 1973-2013 | 24 | 1973-2013 | 19 |
| United States | New Mexico | 1969-2013 | 45 | 1969-2013 | 37 |
| United States | New Orleans | 1974-2007 | 17 | 1983-2007 | 0 |
| United States | New York | 1993-2011 | 15 | 1993-2007 | 0 |
| United States | New York City | 1978-1987 | 0 | NA | NA |
| United States | New York except New York City | 1959-1987 | 0 | 1959-1961 | 0 |
| United States | North Carolina | 1999-2011 | 5 | 2003-2007 | 0 |
| United States | North Dakota | 1999-2011 | 5 | 2003-2007 | 0 |
| United States | Ohio | 1998-2011 | 10 | 1998-2007 | 0 |
| United States | Oklahoma | 1998-2011 | 10 | 1998-2007 | 0 |
| United States | Oregon | 1998-2011 | 10 | 1998-2007 | 0 |
| United States | Pennsylvania | 1998-2011 | 10 | 1998-2007 | 0 |
| United States | Rhode Island | 1998-2011 | 10 | 1998-2007 | 0 |
| United States | Rural Georgia | 1973-2013 | 22 | 1973-2013 | 24 |
| United States | San Francisco | 1969-2007 | 29 | 1973-2007 | 0 |
| United States | San Francisco, Oakland, San Mateo, andSurrounding Area | 1973-2013 | 41 | 1973-2013 | 36 |
| United States | San Jose Monterey | 1973-2013 | 22 | 1973-2013 | 24 |
| **Location** | **Registry** | **Years available from**  **registry** | **Years used for**  **incidence** | **Years available for**  **MI ratio** | **Years used for MI**  **ratio** |
| United States | Seattle | 1973-2013 | 40 | 1973-2013 | 36 |
| United States | South Carolina | 1998-2010 | 10 | 1998-2007 | 0 |
| United States | South Dakota | 2001-2011 | 5 | 2003-2007 | 0 |
| United States | Tennessee | 2003-2011 | 5 | 2003-2007 | 0 |
| United States | Texas | 1998-2011 | 10 | 2998-2007 | 0 |
| United States | Utah | 1966-2013 | 41 | 1973-2013 | 37 |
| United States | Vermont | 1998-2010 | 10 | 1998-2007 | 0 |
| United States | Virginia | 2003-2011 | 5 | 2003-2007 | 0 |
| United States | Washington | 1998-2011 | 10 | 1998-2007 | 0 |
| United States | West Virginia | 1998-2011 | 10 | 1998-2007 | 0 |
| United States | Wisconsin | 1998-2011 | 10 | 1998-2007 | 0 |
| United States | Wyoming | 1999-2011 | 5 | 2003-2007 | 0 |
| Uruguay | Montevideo | 1990-1995 | 6 | 1990-1995 | 0 |
| Uruguay | National Registry | 2002-2007 | 8 | 2002-2007 | 0 |
| Vietnam | Hanoi | 1991-1997 | 8 | 1991-1997 | 0 |
| Vietnam | Ho Chi Minh | 1995-1998 | 4 | 1995-1998 | 0 |
| Zimbabwe | Bulawayo | 1963-1972 | 5 | 1963-1972 | 0 |
| Zimbabwe | Harare | 1990-2006 | 17 | 1990-2006 | 0 |
| Zimbabwe | National Registry | 2005-2006 | 2 | 2005-2006 | 0 |

# Supplementary Table 3.Disability weights

| Health state | Lay description | Estimate | Uncertainty interval | |
| --- | --- | --- | --- | --- |
| Cancer, diagnosis and primary therapy | Has pain, nausea, fatigue, weight loss and high anxiety. | 0.288 | 0.193 | 0.399 |
| Cancer, controlled phase | Has a chronic disease that requires medication every day and causes some worry but minimal interference with daily activities. | 0.049 | 0.031 | 0.072 |
| Cancer, metastatic | Has severe pain, extreme fatigue, weight loss and high anxiety. | 0.451 | 0.307 | 0.600 |
| Terminal phase, with medication | Has lost a lot of weight and regularly uses strong medication to avoid constant pain. The person has no appetite, feels nauseous, and needs to spend most of the day in bed | 0.540 | 0.377 | 0.687 |
| Mastectomy | Had one of the breasts removed and sometimes has pain or swelling in the arms. | 0.036 | 0.020 | 0.057 |
| Stoma | Has a pouch attached to an opening in the belly to collect and empty stools. | 0.095 | 0.063 | 0.131 |
| Laryngectomy | Has difficulty speaking, and others find it difficult to understand. | 0.051 | 0.032 | 0,078 |
| Urinary incontinence | Cannot control urinating. | 0.139 | 0.094 | 0.198 |
| Impotence | Has difficulty in obtaining or maintaining an erection. | 0.017 | 0.009 | 0.030 |
| Cutaneous squamous cell carcinoma, mild | Has a slight, visible physical deformity that others notice, which causes some worry and discomfort. | 0.011 | 0.005 | 0.021 |
| Cutaneous squamous cell carcinoma,  moderate | Has a visible physical deformity that causes others to stare and comment. As a result, the person is worried and has trouble sleeping and concentrating | 0.067 | 0.044 | 0.096 |
| Cutaneous squamous cell carcinoma, severe | Has an obvious physical deformity that is very painful and itchy. The physical deformity makes others uncomfortable, which causes the person to avoid social contact, feel worried, sleep poorly, and think about suicide. | 0.576 | 0.401 | 0.731 |
| Disfigurement due to basal cell  carcinoma | Has a slight, visible physical deformity that others notice, which causes some worry and discomfort. | 0.011 | 0.005 | 0.021 |

# Supplementary Table 4. Socio-Demographic Index groupings by location, based on 2017 values

| **Location Name** | **2017 SDI Index Value** | **SDI Quintile** |
| --- | --- | --- |
| Global | 0.652205351 |  |
| Central Europe, Eastern Europe, and Central Asia | 0.765735064 |  |
| Central Asia | 0.672778523 |  |
| Armenia | 0.702021479 | High-middle SDI |
| Azerbaijan | 0.701169598 | High-middle SDI |
| Georgia | 0.699719344 | High-middle SDI |
| Kazakhstan | 0.735474229 | High-middle SDI |
| Kyrgyzstan | 0.606646902 | Low-middle SDI |
| Mongolia | 0.661854015 | Middle SDI |
| Tajikistan | 0.522612209 | Low-middle SDI |
| Turkmenistan | 0.696418617 | Middle SDI |
| Uzbekistan | 0.629546531 | Middle SDI |
| Central Europe | 0.813976167 |  |
| Albania | 0.684614242 | Middle SDI |
| Bosnia and Herzegovina | 0.712609905 | High-middle SDI |
| Bulgaria | 0.79173721 | High-middle SDI |
| Croatia | 0.824844721 | High SDI |
| Czech Republic | 0.850980459 | High SDI |
| Hungary | 0.816804322 | High-middle SDI |
| Macedonia | 0.75436361 | High-middle SDI |
| Montenegro | 0.788188778 | High-middle SDI |
| Poland | 0.84377326 | High SDI |
| Romania | 0.784193905 | High-middle SDI |
| Serbia | 0.75179332 | High-middle SDI |
| Slovakia | 0.841690487 | High SDI |
| Slovenia | 0.860279598 | High SDI |
| Eastern Europe | 0.785420363 |  |
| Belarus | 0.772665439 | High-middle SDI |
| Estonia | 0.857709406 | High SDI |
| Latvia | 0.825131484 | High SDI |
| Lithuania | 0.840877452 | High SDI |
| Moldova | 0.675572758 | Middle SDI |
| Russian Federation | 0.791738063 | High-middle SDI |
| Ukraine | 0.740061596 | High-middle SDI |
| High-income | 0.854428248 |  |
| Australasia | 0.868509969 |  |
| Australia | 0.873188291 | High SDI |
| **Location Name** | **2017 SDI Index Value** | **SDI Quintile** |
| New Zealand | 0.842273544 | High SDI |
| High-income Asia Pacific | 0.86894981 |  |
| Brunei | 0.856240565 | High SDI |
| Japan | 0.865093512 | High SDI |
| Aichi | 0.874998978 | High SDI |
| Akita | 0.829009097 | High SDI |
| Aomori | 0.825175188 | High SDI |
| Chiba | 0.859238574 | High SDI |
| Ehime | 0.838399264 | High SDI |
| Fukui | 0.852281964 | High SDI |
| Fukuoka | 0.855307883 | High SDI |
| Fukushima | 0.830930555 | High SDI |
| Gifu | 0.84923591 | High SDI |
| Gunma | 0.850963336 | High SDI |
| Hiroshima | 0.862595627 | High SDI |
| Hokkaido | 0.841522308 | High SDI |
| Hyogo | 0.859765235 | High SDI |
| Ibaraki | 0.850665189 | High SDI |
| Ishikawa | 0.856039392 | High SDI |
| Iwate | 0.825241842 | High SDI |
| Kagawa | 0.849935485 | High SDI |
| Kagoshima | 0.829680279 | High SDI |
| Kanagawa | 0.874939342 | High SDI |
| Kochi | 0.825446834 | High SDI |
| Kumamoto | 0.831536501 | High SDI |
| Kyoto | 0.87256007 | High SDI |
| Mie | 0.853567757 | High SDI |
| Miyagi | 0.850313137 | High SDI |
| Miyazaki | 0.823112655 | High SDI |
| Nagano | 0.851209245 | High SDI |
| Nagasaki | 0.826141869 | High SDI |
| Nara | 0.847998888 | High SDI |
| Niigata | 0.843300137 | High SDI |
| Oita | 0.845989117 | High SDI |
| Okayama | 0.855866898 | High SDI |
| Okinawa | 0.817915416 | High SDI |
| Osaka | 0.872366437 | High SDI |
| Saga | 0.833665065 | High SDI |
| Saitama | 0.8520121 | High SDI |
| Shiga | 0.870844353 | High SDI |
| **Location Name** | **2017 SDI Index Value** | **SDI Quintile** |
| Shimane | 0.831040466 | High SDI |
| Shizuoka | 0.858790953 | High SDI |
| Tochigi | 0.853264467 | High SDI |
| Tokushima | 0.845285 | High SDI |
| Tokyo | 0.924328028 | High SDI |
| Tottori | 0.83436659 | High SDI |
| Toyama | 0.859824207 | High SDI |
| Wakayama | 0.839775092 | High SDI |
| Yamagata | 0.831923683 | High SDI |
| Yamaguchi | 0.849441807 | High SDI |
| Yamanashi | 0.854296098 | High SDI |
| South Korea | 0.871955704 | High SDI |
| Singapore | 0.872215248 | High SDI |
| High-income North America | 0.868169406 |  |
| Canada | 0.882086227 | High SDI |
| Greenland | 0.760075292 | High-middle SDI |
| United States | 0.86662166 | High SDI |
| Alabama | 0.837233514 | High SDI |
| Alaska | 0.86060992 | High SDI |
| Arizona | 0.845107314 | High SDI |
| Arkansas | 0.826148933 | High SDI |
| California | 0.872398094 | High SDI |
| Colorado | 0.882128544 | High SDI |
| Connecticut | 0.906486727 | High SDI |
| Delaware | 0.873744053 | High SDI |
| District of Columbia | 0.890203139 | High SDI |
| Florida | 0.863631092 | High SDI |
| Georgia | 0.848426298 | High SDI |
| Hawaii | 0.872290363 | High SDI |
| Idaho | 0.840713155 | High SDI |
| Illinois | 0.879386003 | High SDI |
| Indiana | 0.84792909 | High SDI |
| Iowa | 0.8704793 | High SDI |
| Kansas | 0.864464964 | High SDI |
| Kentucky | 0.83130395 | High SDI |
| Louisiana | 0.834894869 | High SDI |
| Maine | 0.872309993 | High SDI |
| Maryland | 0.895667105 | High SDI |
| Massachusetts | 0.913307727 | High SDI |
| Michigan | 0.867717003 | High SDI |
| **Location Name** | **2017 SDI Index Value** | **SDI Quintile** |
| Minnesota | 0.892987345 | High SDI |
| Mississippi | 0.818942009 | High SDI |
| Missouri | 0.85325798 | High SDI |
| Montana | 0.863383139 | High SDI |
| Nebraska | 0.87308561 | High SDI |
| Nevada | 0.847315003 | High SDI |
| New Hampshire | 0.904304115 | High SDI |
| New Jersey | 0.899124902 | High SDI |
| New Mexico | 0.835274776 | High SDI |
| New York | 0.893442339 | High SDI |
| North Carolina | 0.84978326 | High SDI |
| North Dakota | 0.879820384 | High SDI |
| Ohio | 0.858271211 | High SDI |
| Oklahoma | 0.838181089 | High SDI |
| Oregon | 0.870700326 | High SDI |
| Pennsylvania | 0.878553277 | High SDI |
| Rhode Island | 0.890036984 | High SDI |
| South Carolina | 0.846024965 | High SDI |
| South Dakota | 0.860188872 | High SDI |
| Tennessee | 0.836985155 | High SDI |
| Texas | 0.837777472 | High SDI |
| Utah | 0.855766922 | High SDI |
| Vermont | 0.89559193 | High SDI |
| Virginia | 0.885122306 | High SDI |
| Washington | 0.88440099 | High SDI |
| West Virginia | 0.824706332 | High SDI |
| Wisconsin | 0.87773172 | High SDI |
| Wyoming | 0.869345173 | High SDI |
| Southern Latin America | 0.720171023 |  |
| Argentina | 0.710150584 | High-middle SDI |
| Chile | 0.748081344 | High-middle SDI |
| Uruguay | 0.706753401 | High-middle SDI |
| Western Europe | 0.856820142 |  |
| Andorra | 0.901838419 | High SDI |
| Austria | 0.866029424 | High SDI |
| Belgium | 0.886479194 | High SDI |
| Cyprus | 0.86457342 | High SDI |
| Denmark | 0.917864091 | High SDI |
| Finland | 0.892872363 | High SDI |
| France | 0.864667258 | High SDI |
| **Location Name** | **2017 SDI Index Value** | **SDI Quintile** |
| Germany | 0.869902009 | High SDI |
| Greece | 0.816993531 | High SDI |
| Iceland | 0.907023083 | High SDI |
| Ireland | 0.882181159 | High SDI |
| Israel | 0.81594436 | High-middle SDI |
| Italy | 0.843401161 | High SDI |
| Luxembourg | 0.915748227 | High SDI |
| Malta | 0.835898842 | High SDI |
| Netherlands | 0.911855053 | High SDI |
| Norway | 0.910905362 | High SDI |
| Portugal | 0.777927627 | High-middle SDI |
| Spain | 0.824616837 | High SDI |
| Sweden | 0.883490275 | High SDI |
| Stockholm | 0.914447593 | High SDI |
| Sweden except Stockholm | 0.872833379 | High SDI |
| Switzerland | 0.888752501 | High SDI |
| United Kingdom | 0.843093074 | High SDI |
| England | 0.848869853 | High SDI |
| East Midlands | 0.83007704 | High SDI |
| East of England | 0.840300066 | High SDI |
| Greater London | 0.894369062 | High SDI |
| North East England | 0.820735615 | High SDI |
| North West England | 0.833664296 | High SDI |
| South East England | 0.856169812 | High SDI |
| South West England | 0.841270041 | High SDI |
| West Midlands | 0.829368047 | High SDI |
| Yorkshire and the Humber | 0.829690925 | High SDI |
| Northern Ireland | 0.835352065 | High SDI |
| Scotland | 0.805372811 | High SDI |
| Wales | 0.805748561 | High SDI |
| Latin America and Caribbean | 0.639865451 |  |
| Andean Latin America | 0.628313955 |  |
| Bolivia | 0.587409304 | Low-middle SDI |
| Ecuador | 0.635566909 | Middle SDI |
| Peru | 0.635787809 | Middle SDI |
| Caribbean | 0.637604561 |  |
| Antigua and Barbuda | 0.715130979 | High-middle SDI |
| The Bahamas | 0.75556215 | High-middle SDI |
| Barbados | 0.739423177 | High-middle SDI |
| Belize | 0.602243591 | Low-middle SDI |
| **Location Name** | **2017 SDI Index Value** | **SDI Quintile** |
| Bermuda | 0.80545317 | High-middle SDI |
| Cuba | 0.687667664 | Middle SDI |
| Dominica | 0.68658657 | Middle SDI |
| Dominican Republic | 0.592640504 | Low-middle SDI |
| Grenada | 0.640418422 | Middle SDI |
| Guyana | 0.583747015 | Low-middle SDI |
| Haiti | 0.441665969 | Low SDI |
| Jamaica | 0.678532504 | Middle SDI |
| Puerto Rico | 0.812984477 | High-middle SDI |
| Saint Lucia | 0.652614198 | Middle SDI |
| Saint Vincent and the Grenadines | 0.608304473 | Middle SDI |
| Suriname | 0.64099299 | Middle SDI |
| Trinidad and Tobago | 0.698405348 | Middle SDI |
| Virgin Islands, U.S. | 0.806568682 | High-middle SDI |
| Central Latin America | 0.623192305 |  |
| Colombia | 0.633692252 | Middle SDI |
| Costa Rica | 0.662129526 | Middle SDI |
| El Salvador | 0.59309467 | Low-middle SDI |
| Guatemala | 0.524214498 | Low-middle SDI |
| Honduras | 0.512339813 | Low-middle SDI |
| Mexico | 0.628360997 | Middle SDI |
| Aguascalientes | 0.659089353 | Middle SDI |
| Baja California | 0.656785464 | Middle SDI |
| Baja California Sur | 0.658976353 | Middle SDI |
| Campeche | 0.615914899 | Middle SDI |
| Chiapas | 0.53276266 | Middle SDI |
| Chihuahua | 0.638589391 | Middle SDI |
| Coahuila | 0.645326148 | Middle SDI |
| Colima | 0.65420353 | Middle SDI |
| Durango | 0.623979236 | Middle SDI |
| Guanajuato | 0.62129178 | Middle SDI |
| Guerrero | 0.562442968 | Middle SDI |
| Hidalgo | 0.587458446 | Middle SDI |
| Jalisco | 0.648991934 | Middle SDI |
| Mexico | 0.635428465 | Middle SDI |
| Mexico City | 0.715772109 | Middle SDI |
| Michoacan de Ocampo | 0.58646838 | Middle SDI |
| Morelos | 0.635471941 | Middle SDI |
| Nayarit | 0.620025881 | Middle SDI |
| **Location Name** | **2017 SDI Index Value** | **SDI Quintile** |
| Nuevo Leon | 0.677420872 | Middle SDI |
| Oaxaca | 0.560543467 | Middle SDI |
| Puebla | 0.584252823 | Middle SDI |
| Queretaro | 0.639127345 | Middle SDI |
| Quintana Roo | 0.626303085 | Middle SDI |
| San Luis Potosi | 0.620944629 | Middle SDI |
| Sinaloa | 0.648534168 | Middle SDI |
| Sonora | 0.650495685 | Middle SDI |
| Tabasco | 0.611463527 | Middle SDI |
| Tamaulipas | 0.647006129 | Middle SDI |
| Tlaxcala | 0.604441163 | Middle SDI |
| Veracruz de Ignacio de la Llave | 0.591994 | Middle SDI |
| Yucatan | 0.63033024 | Middle SDI |
| Zacatecas | 0.607654208 | Middle SDI |
| Nicaragua | 0.529616174 | Low-middle SDI |
| Panama | 0.677043867 | Middle SDI |
| Venezuela | 0.655413104 | Middle SDI |
| Tropical Latin America | 0.662126282 |  |
| Brazil | 0.663312473 | Middle SDI |
| Acre | 0.601605235 | Low-middle SDI |
| Alagoas | 0.555715012 | Low-middle SDI |
| Amapa | 0.658517629 | Middle SDI |
| Amazonas | 0.629315711 | Middle SDI |
| Bahia | 0.591019766 | Low-middle SDI |
| Ceara | 0.599501511 | Low-middle SDI |
| Distrito Federal | 0.79189036 | High-middle SDI |
| Espirito Santo | 0.676646695 | Middle SDI |
| Goias | 0.650146424 | Middle SDI |
| Maranhao | 0.507040138 | Low-middle SDI |
| Mato Grosso | 0.662454796 | Middle SDI |
| Mato Grosso do Sul | 0.650210546 | Middle SDI |
| Minas Gerais | 0.660795264 | Middle SDI |
| Para | 0.578664243 | Low-middle SDI |
| Paraiba | 0.574462555 | Low-middle SDI |
| Parana | 0.682436727 | Middle SDI |
| Pernambuco | 0.593552542 | Low-middle SDI |
| Piaui | 0.551619925 | Low-middle SDI |
| Rio de Janeiro | 0.708855843 | High-middle SDI |
| Rio Grande do Norte | 0.605294307 | Low-middle SDI |
| **Location Name** | **2017 SDI Index Value** | **SDI Quintile** |
| Rio Grande do Sul | 0.6927427 | Middle SDI |
| Rondonia | 0.621702361 | Middle SDI |
| Roraima | 0.646354751 | Middle SDI |
| Santa Catarina | 0.702495682 | High-middle SDI |
| Sao Paulo | 0.7200519 | High-middle SDI |
| Sergipe | 0.615627706 | Middle SDI |
| Tocantins | 0.610879077 | Middle SDI |
| Paraguay | 0.618769591 | Middle SDI |
| North Africa and Middle East | 0.638603537 |  |
| North Africa and Middle East | 0.638603537 |  |
| Afghanistan | 0.290254968 | Low SDI |
| Algeria | 0.695849021 | Middle SDI |
| Bahrain | 0.712258604 | High-middle SDI |
| Egypt | 0.604307711 | Low-middle SDI |
| Iran | 0.700086759 | High-middle SDI |
| Iraq | 0.584823813 | Low-middle SDI |
| Jordan | 0.696845045 | Middle SDI |
| Kuwait | 0.785593198 | High-middle SDI |
| Lebanon | 0.729621127 | High-middle SDI |
| Libya | 0.760934217 | High-middle SDI |
| Morocco | 0.579231309 | Low-middle SDI |
| Palestine | 0.541353069 | Low-middle SDI |
| Oman | 0.743531097 | High-middle SDI |
| Qatar | 0.765715882 | High-middle SDI |
| Saudi Arabia | 0.7790137 | High-middle SDI |
| Sudan | 0.477915229 | Low-middle SDI |
| Syria | 0.611084286 | Middle SDI |
| Tunisia | 0.675428611 | Middle SDI |
| Turkey | 0.729481001 | High-middle SDI |
| United Arab Emirates | 0.794722025 | High-middle SDI |
| Yemen | 0.429504407 | Low SDI |
| South Asia | 0.533975763 |  |
| South Asia | 0.533975763 |  |
| Bangladesh | 0.457988721 | Low SDI |
| Bhutan | 0.569907913 | Low-middle SDI |
| India | 0.550242018 | Low-middle SDI |
| Nepal | 0.428511471 | Low SDI |
| Pakistan | 0.492158484 | Low-middle SDI |
| Southeast Asia, East Asia, and Oceania | 0.685403755 |  |
| **Location Name** | **2017 SDI Index Value** | **SDI Quintile** |
| East Asia | 0.708630758 |  |
| China | 0.707319288 | High-middle SDI |
| North Korea | 0.537679957 | Low-middle SDI |
| Taiwan | 0.86418562 | High SDI |
| Oceania | 0.470985744 |  |
| American Samoa | 0.701859796 | High-middle SDI |
| Federated States of Micronesia | 0.575251612 | Low-middle SDI |
| Fiji | 0.641435501 | Middle SDI |
| Guam | 0.794193119 | High-middle SDI |
| Kiribati | 0.426768011 | Low SDI |
| Marshall Islands | 0.550457832 | Low-middle SDI |
| Northern Mariana Islands | 0.75781722 | High-middle SDI |
| Papua New Guinea | 0.418998443 | Low SDI |
| Samoa | 0.576375166 | Low-middle SDI |
| Solomon Islands | 0.425018528 | Low SDI |
| Tonga | 0.624951156 | Middle SDI |
| Vanuatu | 0.475309121 | Low-middle SDI |
| Southeast Asia | 0.640717246 |  |
| Cambodia | 0.481619391 | Low-middle SDI |
| Indonesia | 0.647611359 | Middle SDI |
| Aceh | 0.640414411 | Middle SDI |
| Bali | 0.646777358 | Middle SDI |
| Bangka-Belitung Islands | 0.637063919 | Middle SDI |
| Banten | 0.636136405 | Middle SDI |
| Bengkulu | 0.605588458 | Low-middle SDI |
| Gorontalo | 0.556881893 | Low-middle SDI |
| Jakarta | 0.795041917 | High-middle SDI |
| Jambi | 0.640546524 | Middle SDI |
| West Java | 0.635672599 | Middle SDI |
| Central Java | 0.606724047 | Middle SDI |
| East Java | 0.64169154 | Middle SDI |
| West Kalimantan | 0.589201584 | Low-middle SDI |
| South Kalimantan | 0.623798672 | Middle SDI |
| Central Kalimantan | 0.641894718 | Middle SDI |
| East Kalimantan | 0.746595227 | High-middle SDI |
| North Kalimantan | 0.755952734 | High-middle SDI |
| Riau Islands | 0.727599596 | High-middle SDI |
| Lampung | 0.616299987 | Middle SDI |
| Maluku | 0.555610326 | Low-middle SDI |
| **Location Name** | **2017 SDI Index Value** | **SDI Quintile** |
| North Maluku | 0.546157963 | Low-middle SDI |
| West Nusa Tenggara | 0.556566054 | Low-middle SDI |
| East Nusa Tenggara | 0.518912804 | Low-middle SDI |
| Papua | 0.587862719 | Low-middle SDI |
| West Papua | 0.683007739 | Middle SDI |
| Riau | 0.713955299 | High-middle SDI |
| West Sulawesi | 0.559336878 | Low-middle SDI |
| South Sulawesi | 0.610967812 | Middle SDI |
| Central Sulawesi | 0.612199879 | Middle SDI |
| Southeast Sulawesi | 0.596388581 | Low-middle SDI |
| North Sulawesi | 0.651649236 | Middle SDI |
| West Sumatra | 0.640858055 | Middle SDI |
| South Sumatra | 0.642344679 | Middle SDI |
| North Sumatra | 0.653390877 | Middle SDI |
| Yogyakarta | 0.65012062 | Middle SDI |
| Laos | 0.518788871 | Low-middle SDI |
| Malaysia | 0.759248836 | High-middle SDI |
| Maldives | 0.655286841 | Middle SDI |
| Mauritius | 0.720190502 | High-middle SDI |
| Myanmar | 0.555817824 | Low-middle SDI |
| Philippines | 0.617174396 | Middle SDI |
| Sri Lanka | 0.679706328 | Middle SDI |
| Seychelles | 0.692334035 | Middle SDI |
| Thailand | 0.684276785 | Middle SDI |
| Timor-Leste | 0.504842989 | Low-middle SDI |
| Vietnam | 0.606829222 | Middle SDI |
| Sub-Saharan Africa | 0.445980066 |  |
| Central Sub-Saharan Africa | 0.45690943 |  |
| Angola | 0.460535938 | Low-middle SDI |
| Central African Republic | 0.334449009 | Low SDI |
| Congo | 0.574129526 | Low-middle SDI |
| Democratic Republic of the Congo | 0.364453165 | Low SDI |
| Equatorial Guinea | 0.62522322 | Middle SDI |
| Gabon | 0.650559028 | Middle SDI |
| Eastern Sub-Saharan Africa | 0.387060963 |  |
| Burundi | 0.309705632 | Low SDI |
| Comoros | 0.434289553 | Low SDI |
| Djibouti | 0.484750347 | Low-middle SDI |
| Eritrea | 0.408790995 | Low SDI |
| **Location Name** | **2017 SDI Index Value** | **SDI Quintile** |
| Ethiopia | 0.334181415 | Low SDI |
| Kenya | 0.499471993 | Low-middle SDI |
| Madagascar | 0.330760552 | Low SDI |
| Malawi | 0.349345085 | Low SDI |
| Mozambique | 0.340470577 | Low SDI |
| Rwanda | 0.40744149 | Low SDI |
| Somalia | 0.234806633 | Low SDI |
| South Sudan | 0.274705978 | Low SDI |
| Tanzania | 0.412207128 | Low SDI |
| Uganda | 0.387738241 | Low SDI |
| Zambia | 0.472213354 | Low-middle SDI |
| Southern Sub-Saharan Africa | 0.639979771 |  |
| Botswana | 0.663238118 | Middle SDI |
| Lesotho | 0.493356884 | Low-middle SDI |
| Namibia | 0.615792035 | Middle SDI |
| South Africa | 0.676542582 | Middle SDI |
| Swaziland | 0.577699713 | Low-middle SDI |
| Zimbabwe | 0.463195841 | Low-middle SDI |
| Western Sub-Saharan Africa | 0.441032713 |  |
| Benin | 0.373374857 | Low SDI |
| Burkina Faso | 0.283938202 | Low SDI |
| Cameroon | 0.482039386 | Low-middle SDI |
| Cape Verde | 0.549086441 | Low-middle SDI |
| Chad | 0.252901641 | Low SDI |
| Cote d'Ivoire | 0.412139874 | Low SDI |
| The Gambia | 0.404759628 | Low SDI |
| Ghana | 0.536972566 | Low-middle SDI |
| Guinea | 0.324710505 | Low SDI |
| Guinea-Bissau | 0.348986787 | Low SDI |
| Liberia | 0.328416338 | Low SDI |
| Mali | 0.266900909 | Low SDI |
| Mauritania | 0.470565798 | Low-middle SDI |
| Niger | 0.190617687 | Low SDI |
| Nigeria | 0.49339389 | Low-middle SDI |
| Sao Tome and Principe | 0.488258275 | Low-middle SDI |
| Senegal | 0.373026564 | Low SDI |
| Sierra Leone | 0.357159036 | Low SDI |
| Togo | 0.413313302 | Low SDI |

# Supplementary Table 5. CODEm covariates used, level of covariate, and expected direction of covariate for uterine cancer by cause, sex, and age

| Sex | Age | Model Type | Direction | Level | Covariate | Number of Draws |
| --- | --- | --- | --- | --- | --- | --- |
| female | 15-95+years | Data Rich | 1 | 1 | Mean BMI | 369 |
| female | 15-95+years | Data Rich | 1 | 1 | Log-transformed SEV scalar:Uterus C | 626 |
| female | 15-95+years | Data Rich | -1 | 2 | Fruits adjusted (g) | 35 |
| female | 15-95+years | Data Rich | -1 | 2 | Healthcare access and quality index | 43 |
| female | 15-95+years | Data Rich | -1 | 2 | vegeSupplementary Tables adjusted (g) | 655 |
| female | 15-95+years | Data Rich | 0 | 2 | Total Fertility Rate | 674 |
| female | 15-95+years | Data Rich | 1 | 2 | Cumulative Cigarettes (10 Years) | 2 |
| female | 15-95+years | Data Rich | 1 | 2 | Smoking Prevalence | 2 |
| female | 15-95+years | Data Rich | 1 | 2 | Cumulative Cigarettes (5 Years) | 5 |
| female | 15-95+years | Data Rich | 1 | 2 | Tobacco (cigarettes per capita) | 52 |
| female | 15-95+years | Data Rich | 1 | 2 | Diabetes Age-Standardized Prevalence (proportion) | -- |
| female | 15-95+years | Data Rich | -1 | 3 | Education (years per capita) | 1 |
| female | 15-95+years | Data Rich | 0 | 3 | Socio‐demographic Index | 20 |
| female | 15-95+years | Data Rich | 0 | 3 | LDI (I$ per capita) | -- |
| female | 15-95+years | Global | 1 | 1 | Log‐transformed SEV scalar:Uterus C | 257 |
| female | 15-95+years | Global | 1 | 1 | Mean BMI | 368 |
| female | 15-95+years | Global | -1 | 2 | Fruits adjusted (g) | 265 |
| female | 15-95+years | Global | -1 | 2 | Healthcare access and quality index | 267 |
| female | 15-95+years | Global | -1 | 2 | vegeSupplementary Tables adjusted (g) | 269 |
| female | 15-95+years | Global | 0 | 2 | Total Fertility Rate | 340 |
| female | 15-95+years | Global | 1 | 2 | Smoking Prevalence | 14 |
| female | 15-95+years | Global | 1 | 2 | Cumulative Cigarettes (10 Years) | 17 |
| female | 15-95+years | Global | 1 | 2 | Cumulative Cigarettes (5 Years) | 28 |
| female | 15-95+years | Global | 1 | 2 | Tobacco (cigarettes per capita) | 294 |
| female | 15-95+years | Global | 1 | 2 | Diabetes Age-Standardized Prevalence (proportion) | -- |
| female | 15-95+years | Global | -1 | 3 | Education (years per capita) | 33 |
| female | 15-95+years | Global | 0 | 3 | Socio‐demographic Index | 144 |
| female | 15-95+years | Global | 0 | 3 | LDI (I$ per capita) | -- |

# Supplementary Table 6. Results for CODEm model testing

| **Cause** | **Sex** | **Age Start** | **Age End** | **RMSE In** | **RMSE out** | **Trend In** | **Trend Out** | **Coverage In** | **Coverage Out** |
| --- | --- | --- | --- | --- | --- | --- | --- | --- | --- |
| Uterine cancer [Data Rich] | Female | 15-19yeas | 95+years | 0.284045 | 0.333571 | 0.237777 | 0.241792 | 0.996148 | 0.993771 |
| Uterine cancer [Global] | Female | 15-19years | 95+years | 0.340379 | 0.427043 | 0.275573 | 0.266054 | 0.996863 | 0.990423 |

# Supplementary Table 7. Comparison of GBD 2016 and GBD 2017 covariates used and level of covariates

| **Cause** | **Sex** | **Covariate** | **Level 1 2017** | **Level 2 2017** | **Level 3 2017** | **Level 1 2016** | **Level 2 2016** | **Level 3 2016** |
| --- | --- | --- | --- | --- | --- | --- | --- | --- |
| Uterin cancer | Female | Log-transformed SEV scalar: Uterus C | X |  |  | X |  |  |
| Uterin cancer | Female | Socio-demographic Index |  |  | X |  |  | X |
| Uterin cancer | Female | Tobacco (cigarettes per capita) |  | X |  |  | X |  |
| Uterin cancer | Female | Tobacco (cigarettes per capita) |  | X |  |  | X |  |
| Uterin cancer | Female | Total Fertility Rate |  | X |  |  | X |  |
| Uterin cancer | Female | Total Fertility Rate |  | X |  |  | X |  |
| Uterin cancer | Female | Cumulative Cigarettes (5 Years) |  | X |  |  | X |  |
| Uterin cancer | Female | Smoking Prevalence |  | X |  |  | X |  |
| Uterin cancer | Female | Cumulative Cigarettes (10 Years) |  |  | X |  |  | X |
| Uterin cancer | Female | Education (years per capita) |  |  | X |  |  | X |
| Uterin cancer | Female | fruits adjusted(g) |  | X |  |  | X |  |
| Uterin cancer | Female | Healthcare access and quality inde |  | X |  |  | X |  |
| Uterin cancer | Female | Diabetes Age-Standardized Prevalence (proportion) |  | X |  |  | X |  |
| Uterin cancer | Female | Mean BMI | X |  |  | X |  |  |
| Uterin cancer | Female | vegeSupplementary Tables adjusted(g) |  | X |  |  | X |  |
| Uterin cancer | Female | LDI (I$ per capita) |  |  | X |  |  | X |

# Supplementary Table 8. Total number of site years by cause and source type for GBD 2017

| **Cause** | **Level** | **Vital Registration** | **Vital**  **Registration-Sample** | **Verbal Autopsy** | **Surveillance** | **Survey/Census** | **Sibling History** | **Cancer Registry** | **Police Records** |
| --- | --- | --- | --- | --- | --- | --- | --- | --- | --- |
| Uterine cancer | 3 | 19313 | 793 | 185 |  |  |  | 5382 |  |

9

|  | **Diagnosis/Treatment**  **(months)** | **Remission** | **Disseminated/metastatic**  **(months)** | **Note** | **Terminal (months)** |
| --- | --- | --- | --- | --- | --- |
| Uterine cancer | 4.6^12^ | Calculated based on remainder  of time after attributing other sequelae. | 11.6^10^ | SEER Summary Stage 1997 (Distant site/node involved) 1995-2000 | 1 month |

# Supplementary Figure 1. Flowchart, uterine cancer mortality, YLL estimation


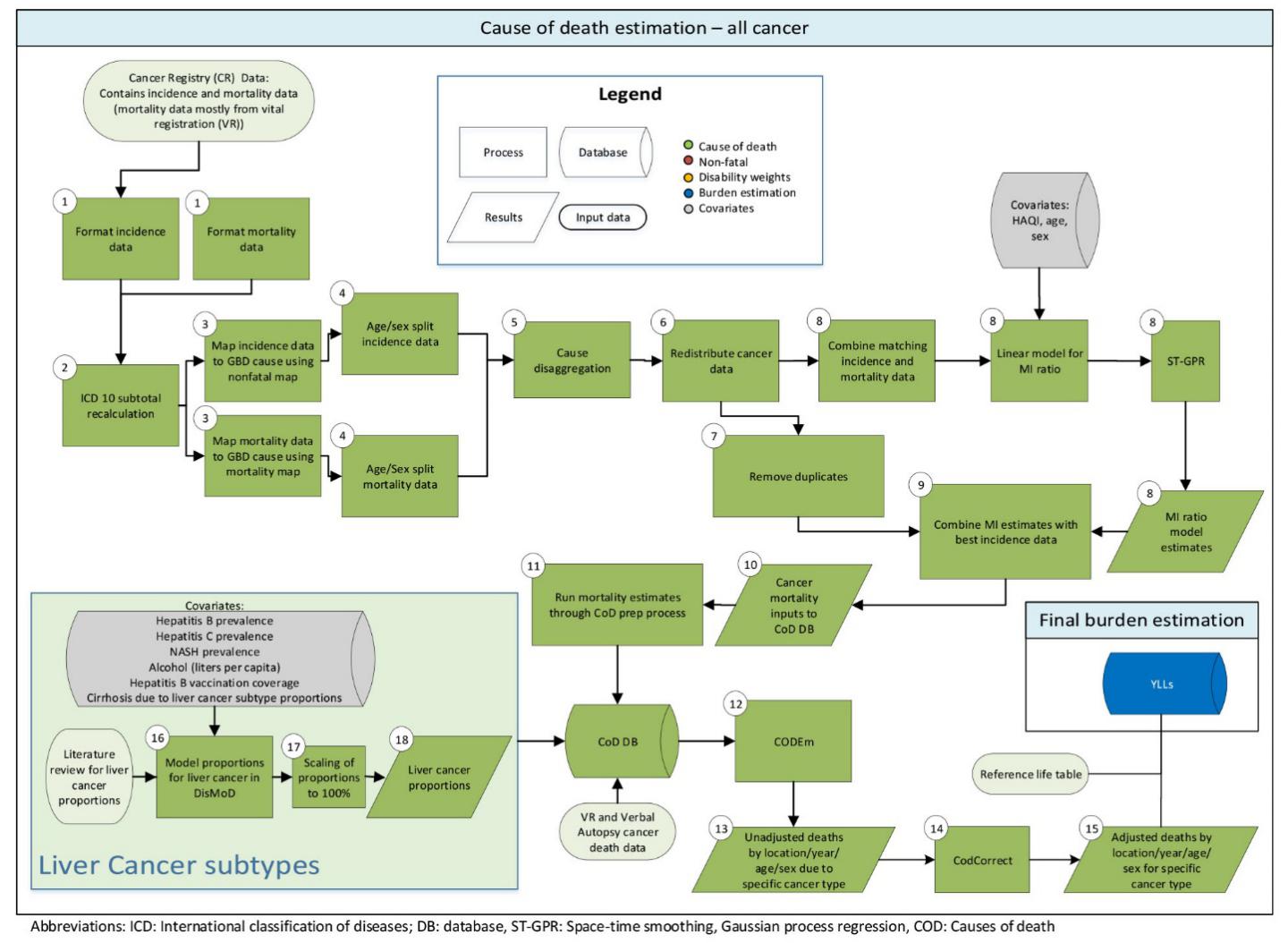


Supplementary Figure 2. Flowchart, uterine cancer incidence, prevalence, YLD^a^ estimation


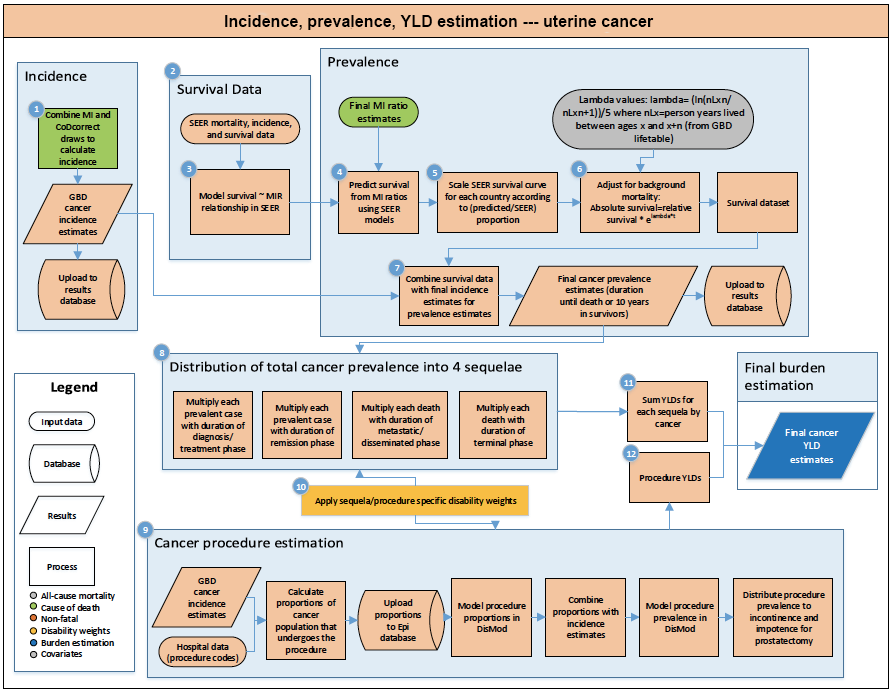


a: years of life lived with disability

# Supplementary Figure 3. Vital Registration and Verbal Autopsy data availability by country, 1980-2017


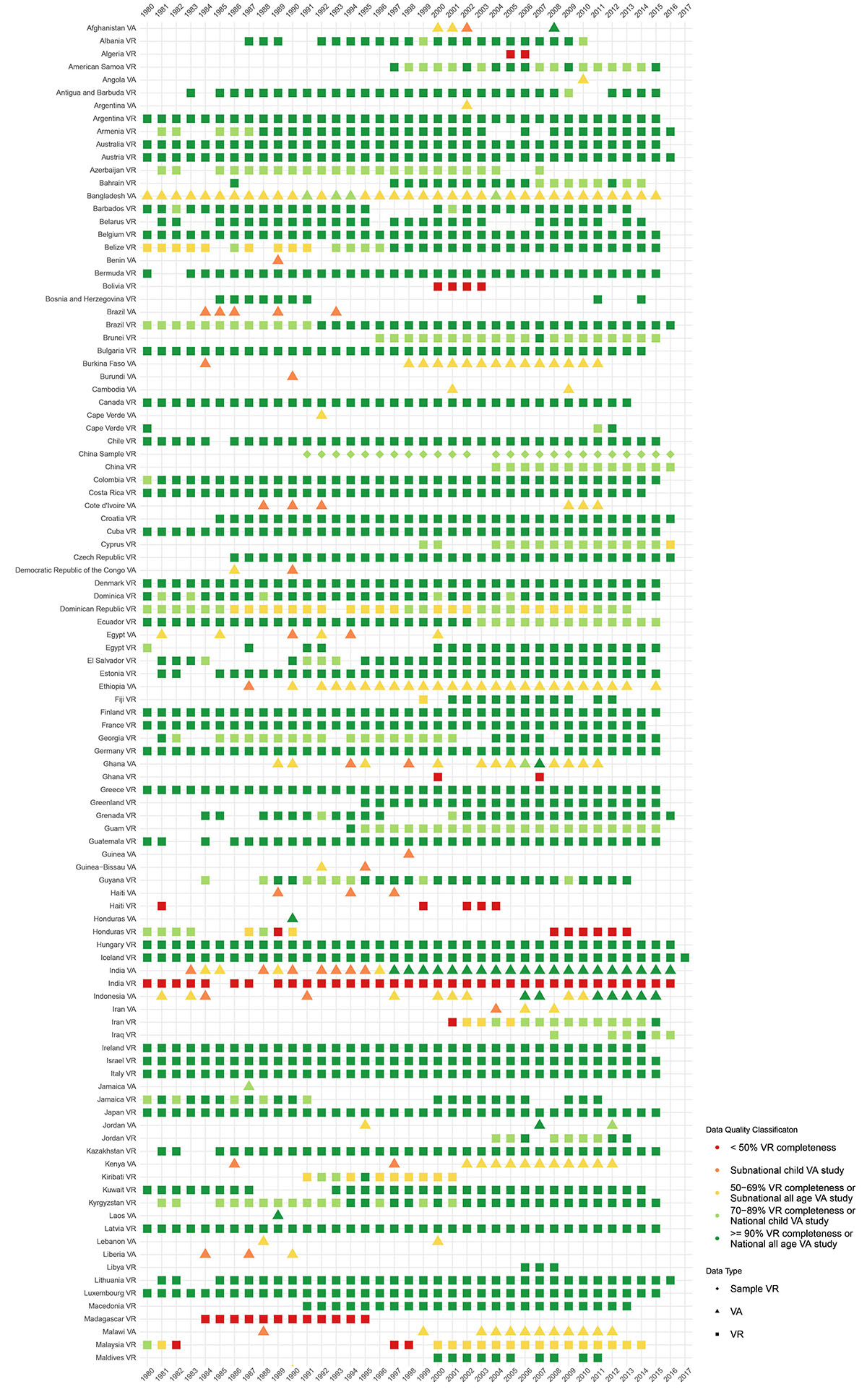


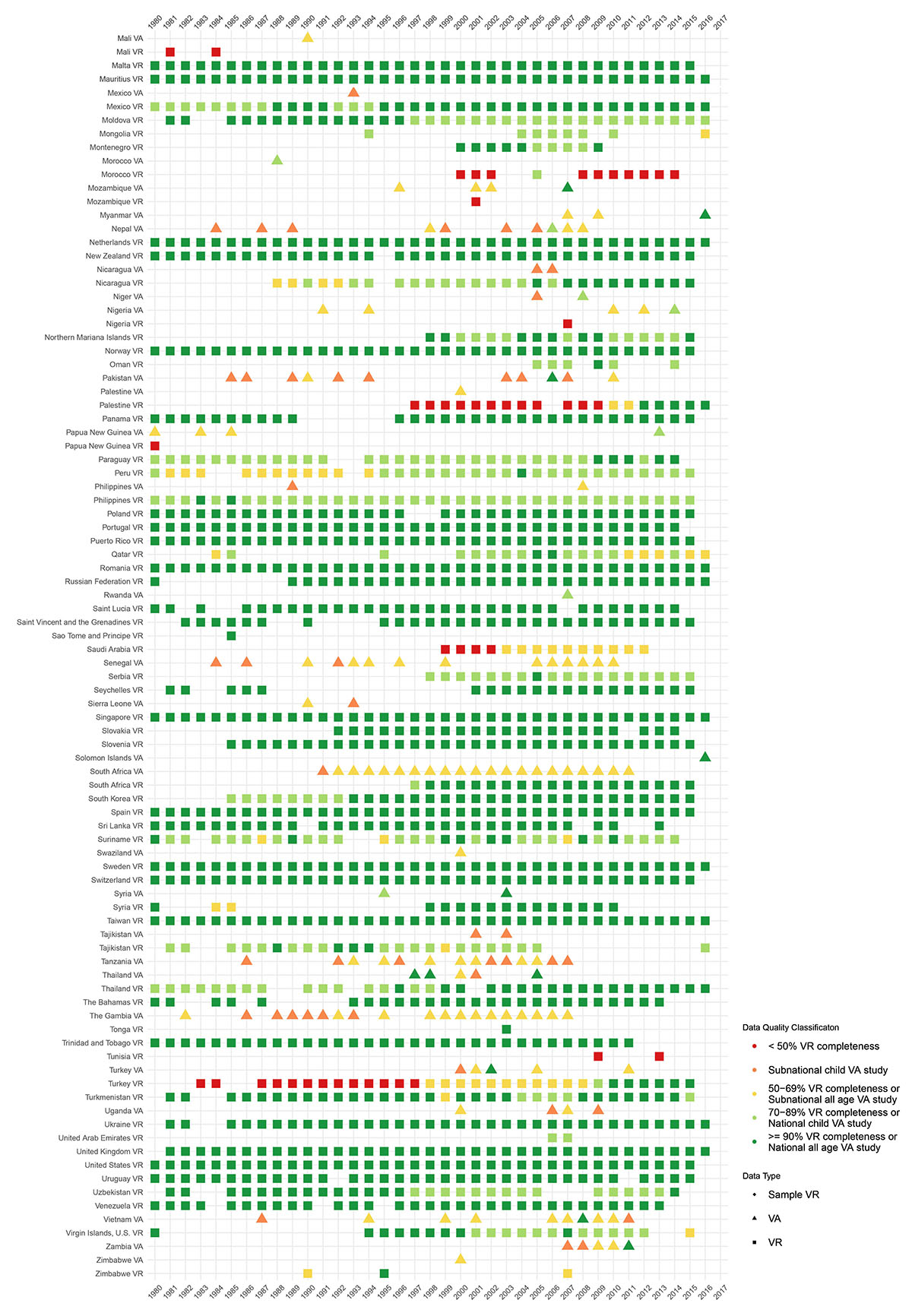


# Supplementary Figure 4. Comparative risk assessment to estimate population attributable fractions for risk factors.


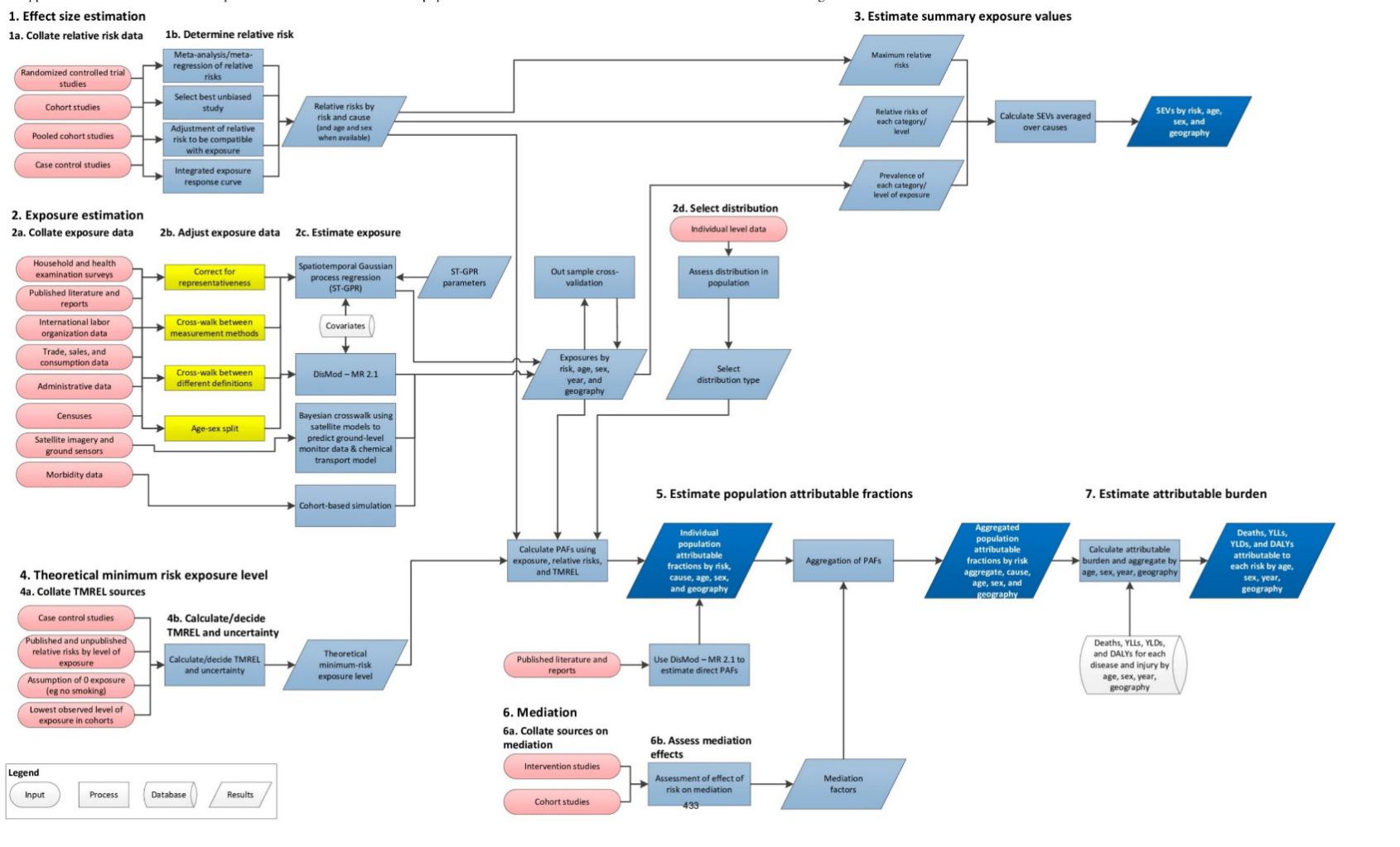


GBD is Global Burden of Disease. SEVs is summary exposure values. TMREL is theoretical minimum-risk exposure level. PAFs is population attribuSupplementary Table fractions. YLLs is years of life lost. YLDs is years lived with disability. DALYs is disability-adjusted life-years. Ovals represent data inputs, rectangular boxes represent analytical steps, cylinders represent databases, and parallelograms represent intermediate and final results.

# Supplementary Figure 5. High body-mass index: data and model flow chart.

**a. Adult (Ages 20+)**


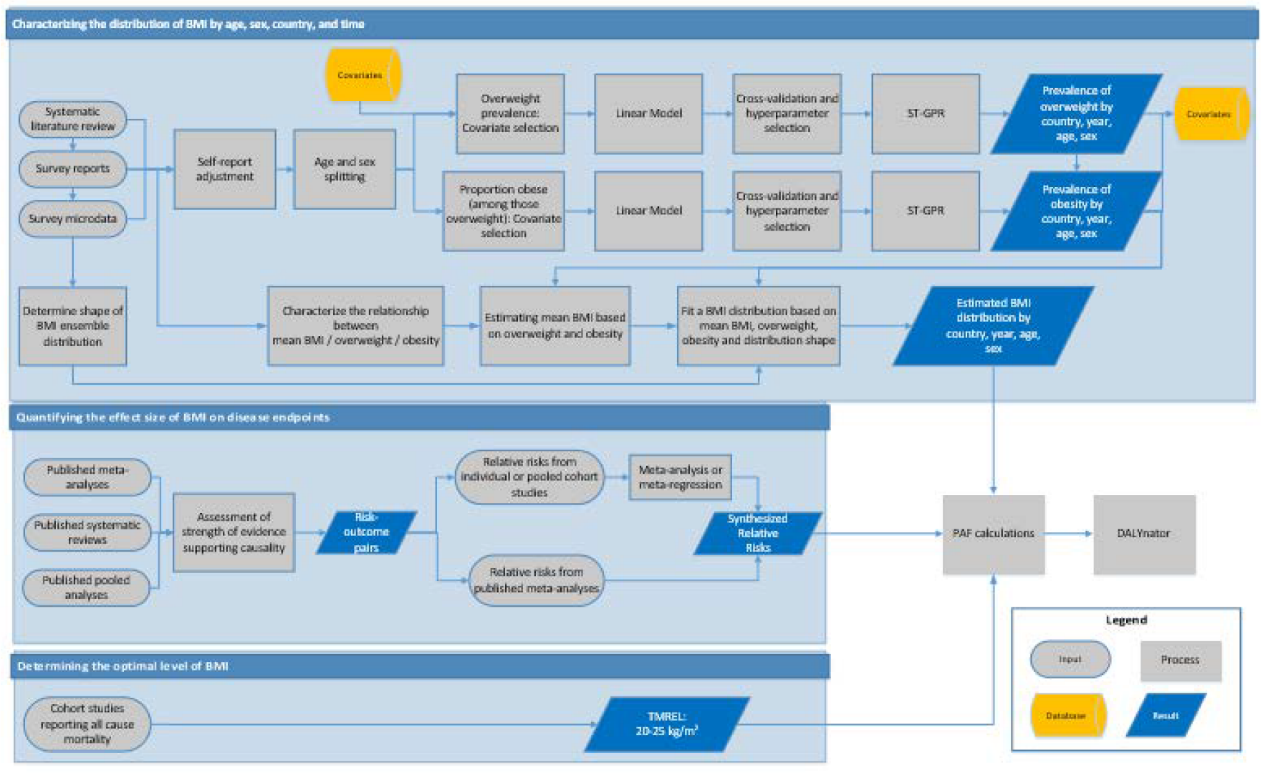
**b. Childhood (Ages 2-19)**

**
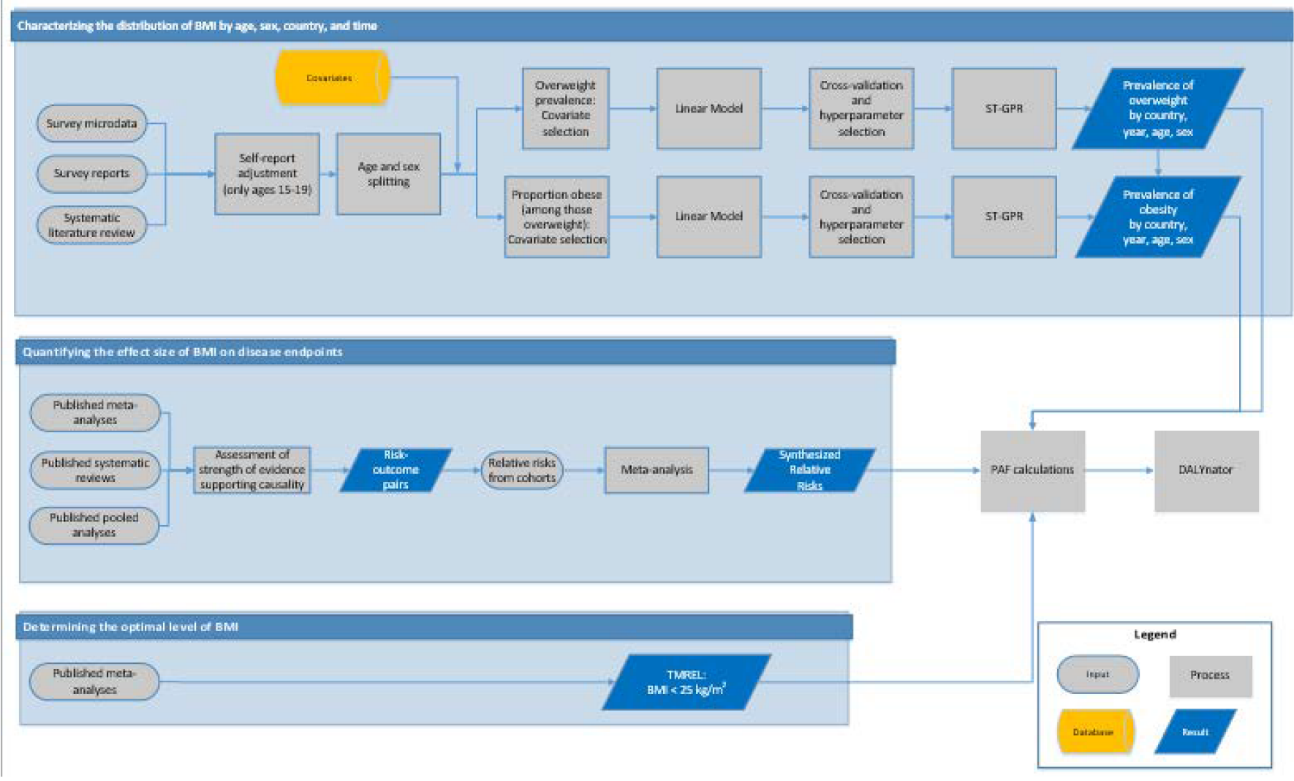
**

1. The material presented here is adapted from the following sources:

   1. GBD 2017 Disease and Injury Incidence and Prevalence Collaborators. Global, regional, and national incidence, prevalence, and years lived with disability for 354 diseases and injuries for 195 countries and territories, 1990–2017: a systematic analysis for the Global Burden of Disease Study 2017. Lancet 2018; 392: 1789–858.

   2. GBD 2017 Causes of Death Collaborators. Global, regional, and national age-sex-specific mortality for 282 causes of death in 195 countries and territories, 1980–2017: a systematic analysis for the Global Burden of Disease Study 2017. Lancet 2018; 392: 1736–88.

   3. GBD 2017 Risk Factors Collaborators. Global, regional, and national comparative risk assessment of 84 behavioural, environmental and occupational, and metabolic risks or clusters of risks, 1990–2017: a systematic analysis for the Global Burden of Disease Study 2017. Lancet 2018; 392: 1923-1994. [↑](#footnote-ref-2)
