# Supplementary material for: Global, Regional, and National Burden of Endometrial Cancer, 1990–2017: Results From the Global Burden of Disease Study, 2017
Source: Front Oncol. 2019 Dec 19;9:1440. doi: 10.3389/fonc.2019.01440 (PMC6930915; doi:10.3389/fonc.2019.01440)
Supplement: Supplementary file 2 [file Table_2.DOC]

**Supplemental file 2**

[**Supplemental Table 10.** Trends in endometrial cancer age-standardized incidence rate by sociodemographic index and region 1990-2017 3](#__RefHeading___Toc27665)

[**Supplemental Table 11.** Trends in endometrial cancer age-standardized prevalence rate by sociodemographic index and region 1990-2017 4](#__RefHeading___Toc30376)

[**Supplemental Table 12.** The relative contributions of each geographical locations in trends of endometrial cancer incidence from 1990-2017 5](#__RefHeading___Toc1822)

[**Supplemental Table 13.** The relative contributions of each geographical locations in trends of endometrial cancer prevalence from 1990-2017 6](#__RefHeading___Toc15548)

[**Supplemental Table 14.**Trends in endometrial cancer age-standardized incidence rate of 195 countries and territories from 1990-2017 7](#__RefHeading___Toc16334)

[**Supplemental Table 15.**Trends in endometrial cancer age-standardized prevalence rate of 195 countries and territories from 1990-2017 12](#__RefHeading___Toc7)

[**Supplemental Table 16.** Trends in endometrial cancer age-standardized mortality rate by sociodemographic index and region 1990-2017 17](#__RefHeading___Toc899)

[**Supplemental Table 17.** The relative contributions of each geographical locations in trends of endometrial cancer mortality from 1990-2017 18](#__RefHeading___Toc2886)

[**Supplemental Table 18.** Trends in endometrial cancer age-standardized mortality rate of 195 countries and territories from 1990-2017 19](#__RefHeading___Toc24900)

[**Supplemental Table 19.** Trends in endometrial cancer age-standardized DALYs^a^ rate by sociodemographic index and region 1990-2017 24](#__RefHeading___Toc27557)

[**Supplemental Table 20.** The relative contributions of each geographical locations in trends of endometrial cancer DALYs^a^ from 1990-2017 25](#__RefHeading___Toc27364)

[**Supplemental Table 21.** Trends in endometrial cancer age-standardized DALYs^a^ rate of 195 countries and territories from 1990-2017 25](#__RefHeading___Toc1970)

[**Supplemental Table 22.** Trends in endometrial cancer age-standardized YLDs^a^ rate by sociodemographic index and region 1990-2017 31](#__RefHeading___Toc16534)

[**Supplemental Table 23.** The relative contributions of each geographical locations in trends of endometrial cancer YLDs^a^ from 1990-2017 32](#__RefHeading___Toc2863)

[**Supplemental Table 24.** Trends in endometrial cancer age-standardized YLDs^a^ rate of 195 countries and territories from 1990-2017 33](#__RefHeading___Toc8716)

[**Supplemental Table 25.** Trends in endometrial cancer age-standardized YLLs^a^ rate by sociodemographic index and region 1990-2017 38](#__RefHeading___Toc13870)

[**Supplemental Table 26.** The relative contributions of each geographical locations in trends of endometrial cancer YLLs^a^ from 1990-2017 39](#__RefHeading___Toc31113)

[**Supplemental Table 27.** Trends in endometrial cancer age-standardized YLLs^a^ rate of 195 countries and territories from 1990-2017 40](#__RefHeading___Toc21088)

[**Supplemental Figure 6.** Trend of global incidence of endometrial cancer by age group, 1990−2017 45](#__RefHeading___Toc14485)

[**Supplemental Figure 7.** Trend of global prevalence of endometrial cancer by age group, 1990−2017 46](#__RefHeading___Toc26490)

[**Supplemental Figure 8.** Trends in the global disease burden of endometrial cancer mortality from 1990−2017. 47](#__RefHeading___Toc23342)

[**Supplemental Figure 9.** The global disease burden of endometrial cancer mortality in 195 countries and territories. 48](#__RefHeading___Toc20506)

[**Supplemental Figure 10.** Trend of global mortality of endometrial cancer by age group, 1990−2017 49](#__RefHeading___Toc19886)

[**Supplemental Figure 11.** Trends in global disease burden of endometrial cancer DALYs^a^ from 1990−2017. 50](#__RefHeading___Toc6812)

[**Supplemental Figure 12.** The global disease burden of endometrial cancer DALYs^a^ in 195 countries and territories. 51](#__RefHeading___Toc4802)

[**Supplemental Figure 13.** Trend of global DALYs^a^ of endometrial cancer by age group,1990−2017 52](#__RefHeading___Toc15797)

[**Supplemental Figure 14.** Trends in the global disease burden of endometrial cancer YLDs^a^ from 1990−2017. 53](#__RefHeading___Toc12828)

[**Supplemental Figure 15.** The global disease burden of endometrial cancer YLDs^a^ in 195 countries and territories. 54](#__RefHeading___Toc9940)

[**Supplemental Figure 16.** Trend of global YLDs^a^ of endometrial cancer by age group, 1990−2017 55](#__RefHeading___Toc32210)

[**Supplemental Figure 17.** Trends in the global disease burden of endometrial cancer YLLs^a^ from 1990 to 2017. 56](#__RefHeading___Toc6295)

[**Supplemental Figure 18.** The global disease burden of endometrial cancer YLLs^a^ in 195 countries and territories. 57](#__RefHeading___Toc27519)

[**Supplemental Figure 19.** Trend of global YLLs^a^ of endometrial cancer by age group, 1990−2017 58](#__RefHeading___Toc16850)

[**Supplemental Figure 20.** Co-evolution of global age-standardized DALYs^a^ with high body-mass index and five SDI quintiles and for GBD regions for endometrial cancer, 1990−2017. 59](#__RefHeading___Toc26530)

This supplementary material has been provided by the authors to give readers additional information about their work.

# Supplemental Table 10. Trends in endometrial cancer age-standardized incidence rate by sociodemographic index and region 1990-2017

| **Characteristics** | **PC^a^(%)** | | | **APC^b^(%)** | | | | |
| --- | --- | --- | --- | --- | --- | --- | --- | --- |
|  | **Value** | **Rank** | | **Value** | **95%CI^c^** | **95%CI** | **Rank** | |
| **Global** | 15.505 |  |  | 0.578 | 0.518 | 0.638 |  |  |
| **Sociodemographic index** |  |  |  |  |  |  |  |  |
| Low | -5.326 | 1 | ↓ | -0.361 | -0.606 | -0.116 | 1 | ↓ |
| Low-middle | 20.494 | 3 | ↑ | 0.507 | 0.395 | 0.619 | 3 | ↑ |
| Middle | 21.057 | 2 | ↑ | 0.750 | 0.636 | 0.865 | 2 | ↑ |
| High-Middle | 9.204 | 4 | ↑ | 0.372 | 0.257 | 0.488 | 4 | ↑ |
| High | 42.811 | 1 | ↑ | 1.425 | 1.366 | 1.484 | 1 | ↑ |
| **Region** |  |  |  |  |  |  |  |  |
| Central Asia | 49.471 | 5 | ↑ | 1.639 | 1.513 | 1.764 | 4 | ↑ |
| East Asia | 14.782 | 17 | ↑ | 0.797 | 0.544 | 1.051 | 11 | ↑ |
| High-income Asia Pacific | 52.129 | 3 | ↑ | 2.097 | 1.831 | 2.363 | 1 | ↑ |
| South Asia | 22.954 | 12 | ↑ | 0.598 | 0.274 | 0.924 | 15 | ↑ |
| Southeast Asia | 11.832 | 19 | ↑ | 0.256 | 0.168 | 0.345 | 19 | ↑ |
| Central Europe | 50.771 | 4 | ↑ | 1.756 | 1.618 | 1.894 | 3 | ↑ |
| Eastern Europe | 25.293 | 11 | ↑ | 0.718 | 0.478 | 0.958 | 12 | ↑ |
| Western Europe | 42.960 | 8 | ↑ | 1.452 | 1.391 | 1.513 | 6 | ↑ |
| Andean Latin America | 19.191 | 15 | ↑ | 0.436 | 0.199 | 0.674 | 17 | ↑ |
| Central Latin America | 44.433 | 7 | ↑ | 1.408 | 1.153 | 1.663 | 7 | ↑ |
| Southern Latin America | 16.781 | 16 | ↑ | 0.358 | 0.140 | 0.577 | 18 | ↑ |
| Tropical Latin America | 29.948 | 10 | ↑ | 0.665 | 0.559 | 0.771 | 13 | ↑ |
| High income North America | 44.518 | 6 | ↑ | 1.278 | 1.195 | 1.362 | 8 | ↑ |
| Central Sub-Saharan Africa | -0.344 | 2 | ↓ | -0.078 | -0.188 | 0.032 | 2 | ↓ |
| Eastern Sub-Saharan Africa | -16.287 | 1 | ↓ | -0.970 | -1.128 | -0.811 | 1 | ↓ |
| Southern Sub-Saharan Africa | 37.299 | 9 | ↑ | 1.179 | 0.805 | 1.555 | 9 | ↑ |
| Western Sub-Saharan Africa | 22.211 | 13 | ↑ | 0.803 | 0.782 | 0.824 | 10 | ↑ |
| North Africa and Middle East | 65.017 | 1 | ↑ | 2.091 | 1.960 | 2.223 | 2 | ↑ |
| Oceania | 14.373 | 18 | ↑ | 0.625 | 0.565 | 0.686 | 14 | ↑ |
| Australasia | 20.024 | 14 | ↑ | 0.532 | 0.432 | 0.633 | 16 | ↑ |
| Caribbean | 61.159 | 2 | ↑ | 1.470 | 1.233 | 1.708 | 5 | ↑ |

a: percent change; b: annual percent change; c: confidence interval

1

| **Characteristics** | **PC^a^(%)** | | | **APC^b^(%)** | | | | |
| --- | --- | --- | --- | --- | --- | --- | --- | --- |
|  | **Value** | **Rank** | | **Value** | **95%CI^c^** | **95%CI** | **Rank** | |
| **Global** | 24.357 |  |  | 0.887 | 0.818 | 0.956 |  |  |
| **Sociodemographic index** |  |  |  |  |  |  |  |  |
| Low | -0.285 | 1 | ↓ | -0.151 | -0.402 | 0.099 | 1 | ↓ |
| Low-middle | 28.373 | 3 | ↑ | 0.775 | 0.660 | 0.890 | 3 | ↑ |
| Middle | 36.948 | 2 | ↑ | 1.231 | 1.093 | 1.368 | 2 | ↑ |
| High-Middle | 19.334 | 4 | ↑ | 0.764 | 0.636 | 0.892 | 4 | ↑ |
| High | 51.022 | 1 | ↑ | 1.640 | 1.578 | 1.702 | 1 | ↑ |
| **Region** |  |  |  |  |  |  |  |  |
| Central Asia | 59.615 | 6 | ↑ | 1.932 | 1.798 | 2.066 | 4 | ↑ |
| East Asia | 33.861 | 11 | ↑ | 1.393 | 1.105 | 1.682 | 9 | ↑ |
| High-income Asia Pacific | 67.214 | 3 | ↑ | 2.457 | 2.171 | 2.743 | 2 | ↑ |
| South Asia | 31.243 | 14 | ↑ | 0.863 | 0.528 | 1.200 | 14 | ↑ |
| Southeast Asia | 21.047 | 18 | ↑ | 0.557 | 0.456 | 0.658 | 19 | ↑ |
| Central Europe | 63.120 | 4 | ↑ | 2.069 | 1.939 | 2.199 | 3 | ↑ |
| Eastern Europe | 32.257 | 13 | ↑ | 0.990 | 0.753 | 1.228 | 12 | ↑ |
| Western Europe | 50.332 | 7 | ↑ | 1.640 | 1.578 | 1.702 | 7 | ↑ |
| Andean Latin America | 33.301 | 12 | ↑ | 0.859 | 0.593 | 1.126 | 15 | ↑ |
| Central Latin America | 61.362 | 5 | ↑ | 1.853 | 1.608 | 2.098 | 5 | ↑ |
| Southern Latin America | 25.594 | 16 | ↑ | 0.629 | 0.398 | 0.860 | 18 | ↑ |
| Tropical Latin America | 43.276 | 9 | ↑ | 1.027 | 0.923 | 1.131 | 11 | ↑ |
| High income North America | 50.053 | 8 | ↑ | 1.424 | 1.338 | 1.511 | 8 | ↑ |
| Central Sub-Saharan Africa | 2.611 | 20 | ↑ | 0.045 | -0.094 | 0.185 | 20 | ↑ |
| Eastern Sub-Saharan Africa | -10.544 | 1 | ↓ | -0.660 | -0.823 | -0.496 | 1 | ↓ |
| Southern Sub-Saharan Africa | 38.844 | 10 | ↑ | 1.199 | 0.942 | 1.457 | 10 | ↑ |
| Western Sub-Saharan Africa | 25.198 | 17 | ↑ | 0.901 | 0.871 | 0.930 | 13 | ↑ |
| North Africa and Middle East | 88.411 | 1 | ↑ | 2.643 | 2.505 | 2.781 | 1 | ↑ |
| Oceania | 16.268 | 19 | ↑ | 0.679 | 0.619 | 0.739 | 17 | ↑ |
| Australasia | 25.944 | 15 | ↑ | 0.704 | 0.599 | 0.810 | 16 | ↑ |
| Caribbean | 71.510 | 2 | ↑ | 1.682 | 1.424 | 1.940 | 6 | ↑ |

a: percent change; b: annual percent change; c: confidence interval

2

|  | **Increasing trend** | **Decreasing trend** |
| --- | --- | --- |
| **Characteristics** | **Contribution rate (%)** | **Contribution rate (%)** |
| **Sociodemographic index** |  |  |
| Low | - | 100 |
| Low-middle | 16.59 | - |
| Middle | 24.57 | - |
| Middle-High | 12.19 |  |
| High | 46.65 |  |
| **Region** |  |  |
| Central Asia | 8.13 | - |
| Eastern Asia | 3.96 | - |
| High-income Asia Pacific | 10.40 | - |
| South Asia | 2.97 | - |
| Southeast Asia | 1.27 | - |
| Central Europe | 8.71 | - |
| Eastern Europe | 3.56 | - |
| Western Europe | 7.20 | - |
| Andean Latin America | 2.16 | - |
| Central Latin America | 6.98 | - |
| Southern Latin America | 1.78 | - |
| Tropical Latin America | 3.30 | - |
| North America | 6.34 | - |
| Central Sub-Saharan Africa | - | 7.45 |
| Eastern Sub-Saharan Africa | - | 92.55 |
| Southern Sub-Saharan Africa | 5.85 | - |
| Western Sub-Saharan Africa | 3.98 | - |
| North Africa and Middle East | 10.37 | - |
| Oceania | 3.10 | - |
| Australasia | 2.64 | - |
| Caribbean | 7.29 | - |

# Supplemental Table 13. The relative contributions of each geographical locations in trends of endometrial cancer prevalence from 1990-2017

|  | **Increasing trend** | **Decreasing trend** |
| --- | --- | --- |
| **Characteristics** | **Contribution rate (%)** | **Contribution rate (%)** |
| **Sociodemographic index** |  |  |
| Low | - | 100.00 |
| Low-middle | 17.58 | - |
| Middle | 27.90 | - |
| Middle-High | 17.33 | - |
| High | 37.19 | - |
| **Region** | - | - |
| Central Asia | 7.56 | - |
| Eastern Asia | 5.45 | - |
| High-income Asia Pacific | 9.62 | - |
| South Asia | 3.38 | - |
| Southeast Asia | 2.18 | - |
| Central Europe | 8.10 | - |
| Eastern Europe | 3.88 | - |
| Western Europe | 6.42 | - |
| Andean Latin America | 3.36 | - |
| Central Latin America | 7.25 | - |
| Southern Latin America | 2.46 | - |
| Tropical Latin America | 4.02 | - |
| North America | 5.58 | - |
| Central Sub-Saharan Africa | 0.18 | - |
| Eastern Sub-Saharan Africa | - | 100.00 |
| Southern Sub-Saharan Africa | 4.69 | - |
| Western Sub-Saharan Africa | 3.53 | - |
| North Africa and Middle East | 10.35 | - |
| Oceania | 2.66 | - |
| Australasia | 2.76 | - |
| Caribbean | 6.58 | - |

# Supplemental Table 14.Trends in endometrial cancer age-standardized incidence rate of 195 countries and territories from 1990-2017

| **Countries and territories** | **PC^a^** | | | **APC^b^** | | | | |
| --- | --- | --- | --- | --- | --- | --- | --- | --- |
|  | **Value** | **Rank** | | **Value** | **95%CI^c^** | **95%CI** | **Rank** | |
| Afghanistan | 10.437 | 141 | ↑ | 0.610 | 0.476 | 0.744 | 119 | ↑ |
| Albania | 80.851 | 33 | ↑ | 2.381 | 2.152 | 2.612 | 34 | ↑ |
| Algeria | 52.250 | 63 | ↑ | 1.929 | 1.767 | 2.090 | 50 | ↑ |
| American Samoa | 84.380 | 30 | ↑ | 3.011 | 2.568 | 3.456 | 18 | ↑ |
| Andorra | 43.915 | 72 | ↑ | 1.297 | 1.206 | 1.388 | 75 | ↑ |
| Angola | -4.714 | 27 | ↓ | -0.304 | -0.379 | -0.230 | 31 | ↓ |
| Antigua and Barbuda | 100.166 | 15 | ↑ | 2.184 | 1.960 | 2.408 | 37 | ↑ |
| Argentina | 3.100 | 157 | ↑ | -0.299 | -0.544 | -0.054 | 32 | ↓ |
| Armenia | 111.461 | 11 | ↑ | 3.455 | 3.082 | 3.829 | 10 | ↑ |
| Australia | 24.599 | 106 | ↑ | 0.611 | 0.496 | 0.726 | 118 | ↑ |
| Austria | -1.168 | 32 | ↓ | -0.092 | -0.191 | 0.008 | 37 | ↓ |
| Azerbaijan | 78.507 | 36 | ↑ | 2.002 | 1.805 | 2.199 | 43 | ↑ |
| Bahrain | 58.110 | 55 | ↑ | 1.158 | 0.889 | 1.427 | 89 | ↑ |
| Bangladesh | -15.723 | 9 | ↓ | -0.264 | -0.537 | 0.009 | 33 | ↓ |
| Barbados | 74.631 | 41 | ↑ | 1.836 | 1.459 | 2.215 | 53 | ↑ |
| Belarus | 15.397 | 127 | ↑ | 0.587 | -0.069 | 1.248 | 123 | ↑ |
| Belgium | 25.128 | 103 | ↑ | 0.740 | 0.562 | 0.918 | 107 | ↑ |
| Belize | 36.694 | 80 | ↑ | 0.951 | 0.661 | 1.243 | 97 | ↑ |
| Benin | 28.976 | 96 | ↑ | 1.164 | 1.079 | 1.250 | 88 | ↑ |
| Bermuda | 16.615 | 123 | ↑ | -0.063 | -0.558 | 0.435 | 40 | ↓ |
| Bhutan | -10.204 | 18 | ↓ | -0.514 | -0.733 | -0.295 | 25 | ↓ |
| Bolivia | 17.694 | 118 | ↑ | 0.517 | 0.477 | 0.558 | 126 | ↑ |
| Bosnia and Herzegovina | 173.602 | 6 | ↑ | 4.210 | 3.611 | 4.812 | 3 | ↑ |
| Botswana | 52.135 | 64 | ↑ | 2.586 | 2.062 | 3.113 | 25 | ↑ |
| Brazil | 31.159 | 91 | ↑ | 0.723 | 0.623 | 0.823 | 108 | ↑ |
| Brunei | 40.849 | 76 | ↑ | 1.975 | 1.641 | 2.311 | 45 | ↑ |
| Bulgaria | 43.665 | 73 | ↑ | 1.967 | 1.498 | 2.437 | 46 | ↑ |
| Burkina Faso | 15.046 | 128 | ↑ | 0.721 | 0.560 | 0.882 | 109 | ↑ |
| Burundi | -29.528 | 3 | ↓ | -1.605 | -1.761 | -1.449 | 2 | ↓ |
| Cambodia | -5.973 | 26 | ↓ | -0.241 | -0.435 | -0.047 | 34 | ↓ |
| Cameroon | 9.490 | 143 | ↑ | 0.399 | 0.246 | 0.552 | 135 | ↑ |
| Canada | 32.147 | 89 | ↑ | 1.348 | 1.178 | 1.519 | 74 | ↑ |
| Cape Verde | 77.635 | 37 | ↑ | 2.197 | 2.094 | 2.299 | 36 | ↑ |
| Central African Republic | -11.043 | 17 | ↓ | -0.431 | -0.522 | -0.341 | 27 | ↓ |
| Chad | 30.007 | 93 | ↑ | 1.209 | 1.137 | 1.282 | 83 | ↑ |
| Chile | 97.030 | 17 | ↑ | 2.876 | 2.691 | 3.061 | 22 | ↑ |
| China | 12.215 | 134 | ↑ | 0.695 | 0.430 | 0.962 | 113 | ↑ |
| Colombia | 5.536 | 151 | ↑ | 0.011 | -0.431 | 0.455 | 152 | ↑ |
| Comoros | -15.550 | 10 | ↓ | -0.746 | -0.867 | -0.624 | 18 | ↓ |
| Congo | 8.640 | 146 | ↑ | 0.242 | 0.142 | 0.343 | 142 | ↑ |
| Costa Rica | 84.482 | 29 | ↑ | 2.433 | 2.175 | 2.691 | 31 | ↑ |
| Cote d'Ivoire | 10.935 | 140 | ↑ | 0.402 | 0.289 | 0.514 | 133 | ↑ |
| Croatia | 53.828 | 62 | ↑ | 2.393 | 2.069 | 2.718 | 33 | ↑ |
| Cuba | 84.639 | 28 | ↑ | 1.887 | 1.585 | 2.189 | 52 | ↑ |
| Cyprus | 58.888 | 53 | ↑ | 1.998 | 1.562 | 2.435 | 44 | ↑ |
| Czech Republic | 5.387 | 152 | ↑ | 0.079 | -0.063 | 0.221 | 149 | ↑ |
| Democratic Republic of the Congo | -0.001 | 34 | ↓ | -0.065 | -0.225 | 0.096 | 39 | ↓ |
| Denmark | 13.560 | 132 | ↑ | 0.264 | 0.087 | 0.441 | 140 | ↑ |
| Djibouti | -9.847 | 20 | ↓ | -0.650 | -0.790 | -0.510 | 21 | ↓ |
| Dominica | 80.624 | 34 | ↑ | 2.044 | 1.798 | 2.291 | 42 | ↑ |
| Dominican Republic | 7.325 | 148 | ↑ | -0.554 | -0.949 | -0.158 | 24 | ↓ |
| Ecuador | 13.464 | 133 | ↑ | 0.916 | 0.462 | 1.371 | 99 | ↑ |
| Egypt | 72.135 | 42 | ↑ | 2.079 | 1.976 | 2.182 | 40 | ↑ |
| El Salvador | 63.656 | 48 | ↑ | 0.976 | 0.690 | 1.263 | 96 | ↑ |
| Equatorial Guinea | 25.853 | 101 | ↑ | 1.214 | 1.046 | 1.383 | 82 | ↑ |
| Eritrea | -3.951 | 28 | ↓ | -0.477 | -0.571 | -0.382 | 26 | ↓ |
| Estonia | 62.507 | 49 | ↑ | 1.960 | 1.638 | 2.284 | 47 | ↑ |
| Ethiopia | -25.267 | 5 | ↓ | -1.388 | -1.518 | -1.258 | 4 | ↓ |
| Federated States of Micronesia | 16.829 | 122 | ↑ | 0.602 | 0.535 | 0.668 | 120 | ↑ |
| Fiji | 20.354 | 113 | ↑ | 1.064 | 0.719 | 1.409 | 92 | ↑ |
| Finland | 21.897 | 111 | ↑ | 0.597 | 0.388 | 0.807 | 122 | ↑ |
| France | 29.510 | 94 | ↑ | 1.206 | 1.040 | 1.371 | 84 | ↑ |
| Gabon | 4.397 | 153 | ↑ | 0.080 | -0.121 | 0.281 | 148 | ↑ |
| Georgia | 148.253 | 8 | ↑ | 3.496 | 2.436 | 4.568 | 9 | ↑ |
| Germany | -1.721 | 31 | ↓ | -0.313 | -0.726 | 0.101 | 30 | ↓ |
| Ghana | 25.003 | 104 | ↑ | 0.747 | 0.706 | 0.789 | 106 | ↑ |
| Greece | 95.873 | 20 | ↑ | 3.053 | 2.759 | 3.347 | 15 | ↑ |
| Greenland | 14.755 | 129 | ↑ | 0.279 | -0.030 | 0.589 | 139 | ↑ |
| Grenada | 71.718 | 44 | ↑ | 2.397 | 2.194 | 2.599 | 32 | ↑ |
| Guam | 15.564 | 126 | ↑ | 0.601 | 0.292 | 0.911 | 121 | ↑ |
| Guatemala | 23.800 | 108 | ↑ | -0.821 | -1.385 | -0.254 | 17 | ↓ |
| Guinea | 16.174 | 124 | ↑ | 0.645 | 0.581 | 0.708 | 115 | ↑ |
| Guinea-Bissau | 19.774 | 114 | ↑ | 0.911 | 0.803 | 1.019 | 100 | ↑ |
| Guyana | 74.815 | 40 | ↑ | 1.644 | 1.188 | 2.102 | 61 | ↑ |
| Haiti | 13.674 | 131 | ↑ | 0.461 | 0.437 | 0.486 | 129 | ↑ |
| Honduras | 107.062 | 14 | ↑ | 2.435 | 2.190 | 2.681 | 30 | ↑ |
| Hungary | -11.974 | 15 | ↓ | -1.014 | -1.576 | -0.450 | 9 | ↓ |
| Iceland | -9.019 | 21 | ↓ | -0.560 | -0.779 | -0.340 | 23 | ↓ |
| India | 26.643 | 100 | ↑ | 0.673 | 0.223 | 1.125 | 114 | ↑ |
| Indonesia | 4.052 | 154 | ↑ | 0.100 | 0.050 | 0.150 | 147 | ↑ |
| Iran | 110.304 | 12 | ↑ | 3.562 | 2.978 | 4.149 | 7 | ↑ |
| Iraq | 8.243 | 147 | ↑ | 0.204 | -0.009 | 0.418 | 144 | ↑ |
| Ireland | 56.563 | 57 | ↑ | 2.567 | 2.237 | 2.898 | 26 | ↑ |
| Israel | 88.235 | 24 | ↑ | 2.278 | 1.895 | 2.663 | 35 | ↑ |
| Italy | 217.504 | 1 | ↑ | 3.283 | 2.435 | 4.139 | 12 | ↑ |
| Jamaica | 179.345 | 4 | ↑ | 3.530 | 3.041 | 4.022 | 8 | ↑ |
| Japan | 91.624 | 23 | ↑ | 2.894 | 2.594 | 3.195 | 21 | ↑ |
| Jordan | 33.315 | 86 | ↑ | 0.430 | 0.062 | 0.799 | 132 | ↑ |
| Kazakhstan | 11.319 | 138 | ↑ | -0.109 | -0.539 | 0.322 | 36 | ↓ |
| Kenya | 11.542 | 136 | ↑ | 0.313 | 0.262 | 0.364 | 136 | ↑ |
| Kiribati | 0.498 | 160 | ↑ | 0.250 | 0.073 | 0.427 | 141 | ↑ |
| Kuwait | 27.267 | 98 | ↑ | 1.717 | 1.038 | 2.400 | 58 | ↑ |
| Kyrgyzstan | 35.128 | 82 | ↑ | 1.295 | 0.886 | 1.706 | 76 | ↑ |
| Laos | -13.808 | 11 | ↓ | -0.713 | -0.862 | -0.565 | 19 | ↓ |
| Latvia | 96.577 | 18 | ↑ | 3.019 | 2.674 | 3.366 | 17 | ↑ |
| Lebanon | 112.643 | 10 | ↑ | 2.994 | 2.836 | 3.152 | 19 | ↑ |
| Lesotho | 42.225 | 74 | ↑ | 1.797 | 1.333 | 2.263 | 54 | ↑ |
| Liberia | 24.600 | 105 | ↑ | 1.232 | 1.017 | 1.447 | 81 | ↑ |
| Libya | 87.615 | 25 | ↑ | 2.728 | 2.431 | 3.025 | 24 | ↑ |
| Lithuania | 79.994 | 35 | ↑ | 1.771 | 1.490 | 2.052 | 55 | ↑ |
| Luxembourg | 28.612 | 97 | ↑ | 0.770 | 0.528 | 1.013 | 105 | ↑ |
| Macedonia | 124.257 | 9 | ↑ | 3.452 | 3.038 | 3.868 | 11 | ↑ |
| Madagascar | -16.111 | 8 | ↓ | -0.844 | -0.952 | -0.735 | 15 | ↓ |
| Malawi | -7.893 | 22 | ↓ | -1.013 | -1.326 | -0.700 | 10 | ↓ |
| Malaysia | 54.641 | 60 | ↑ | 1.937 | 1.608 | 2.268 | 49 | ↑ |
| Maldives | -6.160 | 25 | ↓ | -0.664 | -0.893 | -0.435 | 20 | ↓ |
| Mali | -3.724 | 29 | ↓ | -0.082 | -0.214 | 0.049 | 38 | ↓ |
| Malta | 57.866 | 56 | ↑ | 1.585 | 1.444 | 1.726 | 64 | ↑ |
| Marshall Islands | 36.204 | 81 | ↑ | 1.129 | 1.025 | 1.233 | 90 | ↑ |
| Mauritania | 18.084 | 117 | ↑ | 0.784 | 0.677 | 0.891 | 104 | ↑ |
| Mauritius | -6.913 | 24 | ↓ | -1.024 | -1.275 | -0.773 | 8 | ↓ |
| Mexico | 77.495 | 38 | ↑ | 2.538 | 2.272 | 2.804 | 28 | ↑ |
| Moldova | 33.343 | 85 | ↑ | 0.618 | 0.261 | 0.977 | 116 | ↑ |
| Mongolia | 32.518 | 88 | ↑ | 0.715 | 0.258 | 1.174 | 111 | ↑ |
| Montenegro | 50.113 | 66 | ↑ | 1.508 | 1.357 | 1.658 | 67 | ↑ |
| Morocco | 44.943 | 70 | ↑ | 1.439 | 1.355 | 1.523 | 69 | ↑ |
| Mozambique | -3.637 | 30 | ↓ | -0.155 | -0.363 | 0.053 | 35 | ↓ |
| Myanmar | -7.213 | 23 | ↓ | -0.375 | -0.462 | -0.289 | 28 | ↓ |
| Namibia | -11.423 | 16 | ↓ | -1.178 | -1.785 | -0.568 | 6 | ↓ |
| Nepal | -9.917 | 19 | ↓ | -0.325 | -0.792 | 0.143 | 29 | ↓ |
| Netherlands | 55.418 | 58 | ↑ | 1.690 | 1.573 | 1.808 | 59 | ↑ |
| New Zealand | 2.087 | 158 | ↑ | 0.179 | 0.092 | 0.267 | 145 | ↑ |
| Nicaragua | 19.102 | 115 | ↑ | 1.377 | 0.801 | 1.956 | 73 | ↑ |
| Niger | 11.381 | 137 | ↑ | 0.503 | 0.416 | 0.590 | 127 | ↑ |
| Nigeria | 21.505 | 112 | ↑ | 0.828 | 0.732 | 0.925 | 101 | ↑ |
| North Korea | 0.292 | 161 | ↑ | -0.019 | -0.238 | 0.201 | 42 | ↓ |
| Northern Mariana Islands | 34.270 | 83 | ↑ | 1.284 | 0.856 | 1.714 | 79 | ↑ |
| Norway | 32.852 | 87 | ↑ | 1.099 | 0.801 | 1.397 | 91 | ↑ |
| Oman | 76.084 | 39 | ↑ | 2.065 | 1.917 | 2.214 | 41 | ↑ |
| Pakistan | 38.747 | 77 | ↑ | 1.010 | 0.916 | 1.103 | 94 | ↑ |
| Palestine | 17.538 | 120 | ↑ | 0.706 | 0.442 | 0.970 | 112 | ↑ |
| Panama | 97.106 | 16 | ↑ | 3.067 | 2.799 | 3.337 | 14 | ↑ |
| Papua New Guinea | 11.143 | 139 | ↑ | 0.480 | 0.425 | 0.535 | 128 | ↑ |
| Paraguay | -0.822 | 33 | ↓ | -0.991 | -1.294 | -0.686 | 11 | ↓ |
| Peru | 22.387 | 110 | ↑ | 0.059 | -0.396 | 0.515 | 150 | ↑ |
| Philippines | 2.001 | 159 | ↑ | -0.563 | -0.888 | -0.237 | 22 | ↓ |
| Poland | 92.766 | 22 | ↑ | 2.849 | 2.703 | 2.996 | 23 | ↑ |
| Portugal | 29.334 | 95 | ↑ | 0.806 | 0.668 | 0.943 | 103 | ↑ |
| Puerto Rico | 71.747 | 43 | ↑ | 2.104 | 1.897 | 2.312 | 38 | ↑ |
| Qatar | 175.669 | 5 | ↑ | 4.136 | 3.644 | 4.630 | 4 | ↑ |
| Romania | 58.893 | 52 | ↑ | 1.578 | 1.402 | 1.753 | 66 | ↑ |
| Russian Federation | 7.312 | 149 | ↑ | 0.125 | -0.170 | 0.420 | 146 | ↑ |
| Rwanda | -27.433 | 4 | ↓ | -1.588 | -1.831 | -1.344 | 3 | ↓ |
| Saint Lucia | 54.786 | 59 | ↑ | 1.284 | 1.107 | 1.462 | 78 | ↑ |
| Saint Vincent and the Grenadines | 52.018 | 65 | ↑ | 1.177 | 0.948 | 1.408 | 87 | ↑ |
| Samoa | 13.720 | 130 | ↑ | 0.438 | 0.338 | 0.539 | 131 | ↑ |
| Sao Tome and Principe | 54.037 | 61 | ↑ | 1.583 | 1.525 | 1.641 | 65 | ↑ |
| Saudi Arabia | 194.296 | 3 | ↑ | 5.044 | 4.679 | 5.411 | 2 | ↑ |
| Senegal | 27.051 | 99 | ↑ | 1.042 | 0.945 | 1.139 | 93 | ↑ |
| Serbia | 85.147 | 27 | ↑ | 3.096 | 2.790 | 3.403 | 13 | ↑ |
| Seychelles | 19.042 | 116 | ↑ | 0.291 | 0.103 | 0.479 | 138 | ↑ |
| Sierra Leone | 44.854 | 71 | ↑ | 1.749 | 1.611 | 1.887 | 57 | ↑ |
| Singapore | 94.928 | 21 | ↑ | 2.493 | 2.007 | 2.981 | 29 | ↑ |
| Slovakia | 37.092 | 79 | ↑ | 1.187 | 1.083 | 1.291 | 85 | ↑ |
| Slovenia | 17.571 | 119 | ↑ | 0.306 | 0.141 | 0.472 | 137 | ↑ |
| Solomon Islands | 3.223 | 156 | ↑ | 0.050 | -0.011 | 0.112 | 151 | ↑ |
| Somalia | -13.278 | 13 | ↓ | -0.827 | -0.993 | -0.660 | 16 | ↓ |
| South Africa | 41.887 | 75 | ↑ | 1.181 | 0.817 | 1.547 | 86 | ↑ |
| South Korea | -32.611 | 2 | ↓ | -0.906 | -1.740 | -0.064 | 14 | ↓ |
| South Sudan | -20.119 | 7 | ↓ | -1.144 | -1.306 | -0.983 | 7 | ↓ |
| Spain | 38.467 | 78 | ↑ | 1.462 | 1.316 | 1.609 | 68 | ↑ |
| Sri Lanka | 83.552 | 31 | ↑ | 2.964 | 2.749 | 3.178 | 20 | ↑ |
| Sudan | 22.558 | 109 | ↑ | 0.926 | 0.745 | 1.108 | 98 | ↑ |
| Suriname | 64.758 | 46 | ↑ | 1.616 | 1.374 | 1.859 | 63 | ↑ |
| Swaziland | 11.908 | 135 | ↑ | 0.401 | -0.018 | 0.821 | 134 | ↑ |
| Sweden | 5.810 | 150 | ↑ | -0.020 | -0.262 | 0.222 | 41 | ↓ |
| Switzerland | 3.277 | 155 | ↑ | 0.003 | -0.321 | 0.329 | 153 | ↑ |
| Syria | 45.124 | 69 | ↑ | 1.384 | 0.900 | 1.871 | 72 | ↑ |
| Taiwan | 211.768 | 2 | ↑ | 6.076 | 5.510 | 6.645 | 1 | ↑ |
| Tajikistan | 164.593 | 7 | ↑ | 4.007 | 3.523 | 4.493 | 5 | ↑ |
| Tanzania | -13.738 | 12 | ↓ | -0.973 | -1.247 | -0.698 | 13 | ↓ |
| Thailand | 30.806 | 92 | ↑ | 0.537 | 0.358 | 0.717 | 125 | ↑ |
| The Bahamas | 59.376 | 51 | ↑ | 1.626 | 1.284 | 1.969 | 62 | ↑ |
| The Gambia | 25.424 | 102 | ↑ | 0.988 | 0.914 | 1.062 | 95 | ↑ |
| Timor-Leste | 8.997 | 145 | ↑ | 0.545 | 0.293 | 0.798 | 124 | ↑ |
| Togo | 16.157 | 125 | ↑ | 0.613 | 0.557 | 0.670 | 117 | ↑ |
| Tonga | 24.324 | 107 | ↑ | 0.828 | 0.762 | 0.894 | 102 | ↑ |
| Trinidad and Tobago | 61.344 | 50 | ↑ | 1.757 | 1.495 | 2.020 | 56 | ↑ |
| Tunisia | 64.320 | 47 | ↑ | 1.420 | 1.226 | 1.614 | 70 | ↑ |
| Turkey | 58.500 | 54 | ↑ | 1.959 | 1.756 | 2.162 | 48 | ↑ |
| Turkmenistan | -22.758 | 6 | ↓ | -1.268 | -2.135 | -0.393 | 5 | ↓ |
| Uganda | -12.582 | 14 | ↓ | -0.974 | -1.181 | -0.766 | 12 | ↓ |
| Ukraine | 96.535 | 19 | ↑ | 2.554 | 2.114 | 2.997 | 27 | ↑ |
| United Arab Emirates | 86.114 | 26 | ↑ | 1.924 | 1.527 | 2.322 | 51 | ↑ |
| United Kingdom | 82.033 | 32 | ↑ | 3.027 | 2.714 | 3.340 | 16 | ↑ |
| United States | 46.199 | 68 | ↑ | 1.290 | 1.200 | 1.381 | 77 | ↑ |
| Uruguay | 10.094 | 142 | ↑ | 0.210 | 0.073 | 0.346 | 143 | ↑ |
| Uzbekistan | 107.851 | 13 | ↑ | 3.826 | 3.269 | 4.385 | 6 | ↑ |
| Vanuatu | 17.144 | 121 | ↑ | 0.715 | 0.668 | 0.761 | 110 | ↑ |
| Venezuela | 9.180 | 144 | ↑ | 0.445 | 0.049 | 0.841 | 130 | ↑ |
| Vietnam | 48.622 | 67 | ↑ | 1.683 | 1.551 | 1.814 | 60 | ↑ |
| Virgin Islands, US | 71.500 | 45 | ↑ | 2.088 | 1.682 | 2.495 | 39 | ↑ |
| Yemen | 34.059 | 84 | ↑ | 1.255 | 1.176 | 1.334 | 80 | ↑ |
| Zambia | -33.542 | 1 | ↓ | -2.101 | -2.616 | -1.583 | 1 | ↓ |
| Zimbabwe | 32.145 | 90 | ↑ | 1.396 | 0.671 | 2.125 | 71 | ↑ |

a: percent change; b: annual percent change; c: confidence interval

# Supplemental Table 15.Trends in endometrial cancer age-standardized prevalence rate of 195 countries and territories from 1990-2017

| **Countries and territories** | **PC^a^** | | | **APC^b^** | | | | |
| --- | --- | --- | --- | --- | --- | --- | --- | --- |
|  | **Value** | **Rank** | | **Value** | **95%CI^c^** | **95%CI** | **Rank** | |
| Afghanistan | 14.983 | 142 | ↑ | 0.795 | 0.645 | 0.946 | 120 | ↑ |
| Albania | 98.423 | 30 | ↑ | 2.721 | 2.479 | 2.963 | 31 | ↑ |
| Algeria | 69.090 | 56 | ↑ | 2.315 | 2.152 | 2.478 | 47 | ↑ |
| American Samoa | 90.588 | 37 | ↑ | 3.147 | 2.698 | 3.597 | 23 | ↑ |
| Andorra | 48.403 | 74 | ↑ | 1.389 | 1.308 | 1.470 | 83 | ↑ |
| Angola | 0.219 | 170 | ↑ | -0.089 | -0.171 | -0.006 | 31 | ↓ |
| Antigua and Barbuda | 107.953 | 23 | ↑ | 2.333 | 2.124 | 2.544 | 45 | ↑ |
| Argentina | 9.087 | 154 | ↑ | -0.113 | -0.374 | 0.150 | 28 | ↓ |
| Armenia | 126.544 | 12 | ↑ | 3.774 | 3.397 | 4.152 | 9 | ↑ |
| Australia | 30.609 | 105 | ↑ | 0.782 | 0.663 | 0.901 | 122 | ↑ |
| Austria | 3.682 | 163 | ↑ | 0.089 | -0.013 | 0.191 | 157 | ↑ |
| Azerbaijan | 95.329 | 32 | ↑ | 2.441 | 2.202 | 2.680 | 39 | ↑ |
| Bahrain | 83.750 | 48 | ↑ | 1.786 | 1.505 | 2.069 | 67 | ↑ |
| Bangladesh | -8.521 | 13 | ↓ | 0.002 | -0.286 | 0.291 | 162 | ↑ |
| Barbados | 85.069 | 45 | ↑ | 2.004 | 1.595 | 2.415 | 55 | ↑ |
| Belarus | 21.771 | 125 | ↑ | 0.800 | 0.164 | 1.439 | 119 | ↑ |
| Belgium | 29.699 | 108 | ↑ | 0.874 | 0.697 | 1.052 | 113 | ↑ |
| Belize | 46.224 | 77 | ↑ | 1.197 | 0.917 | 1.477 | 96 | ↑ |
| Benin | 31.484 | 102 | ↑ | 1.221 | 1.142 | 1.300 | 94 | ↑ |
| Bermuda | 27.038 | 114 | ↑ | 0.225 | -0.275 | 0.728 | 152 | ↑ |
| Bhutan | -1.413 | 23 | ↓ | -0.157 | -0.383 | 0.071 | 26 | ↓ |
| Bolivia | 28.400 | 111 | ↑ | 0.872 | 0.832 | 0.912 | 114 | ↑ |
| Bosnia and Herzegovina | 205.590 | 5 | ↑ | 4.650 | 3.987 | 5.318 | 4 | ↑ |
| Botswana | 57.641 | 67 | ↑ | 2.700 | 2.165 | 3.238 | 32 | ↑ |
| Brazil | 44.725 | 78 | ↑ | 1.089 | 0.991 | 1.187 | 101 | ↑ |
| Brunei | 56.505 | 69 | ↑ | 2.333 | 2.033 | 2.634 | 46 | ↑ |
| Bulgaria | 48.670 | 73 | ↑ | 2.116 | 1.613 | 2.621 | 53 | ↑ |
| Burkina Faso | 18.918 | 130 | ↑ | 0.865 | 0.695 | 1.036 | 115 | ↑ |
| Burundi | -25.199 | 2 | ↓ | -1.290 | -1.417 | -1.162 | 2 | ↓ |
| Cambodia | 1.428 | 168 | ↑ | 0.058 | -0.160 | 0.276 | 158 | ↑ |
| Cameroon | 11.822 | 151 | ↑ | 0.474 | 0.365 | 0.583 | 140 | ↑ |
| Canada | 39.324 | 91 | ↑ | 1.557 | 1.392 | 1.723 | 75 | ↑ |
| Cape Verde | 85.057 | 46 | ↑ | 2.393 | 2.286 | 2.500 | 42 | ↑ |
| Central African Republic | -9.886 | 11 | ↓ | -0.381 | -0.438 | -0.323 | 21 | ↓ |
| Chad | 30.770 | 104 | ↑ | 1.237 | 1.167 | 1.307 | 93 | ↑ |
| Chile | 122.489 | 15 | ↑ | 3.348 | 3.166 | 3.531 | 15 | ↑ |
| China | 31.317 | 103 | ↑ | 1.297 | 0.994 | 1.601 | 89 | ↑ |
| Colombia | 18.288 | 134 | ↑ | 0.496 | 0.051 | 0.944 | 138 | ↑ |
| Comoros | -11.886 | 9 | ↓ | -0.560 | -0.677 | -0.444 | 16 | ↓ |
| Congo | 12.591 | 150 | ↑ | 0.446 | 0.346 | 0.546 | 143 | ↑ |
| Costa Rica | 97.485 | 31 | ↑ | 2.755 | 2.491 | 3.020 | 30 | ↑ |
| Cote d'Ivoire | 13.288 | 147 | ↑ | 0.523 | 0.448 | 0.599 | 137 | ↑ |
| Croatia | 62.354 | 63 | ↑ | 2.614 | 2.288 | 2.941 | 34 | ↑ |
| Cuba | 94.642 | 33 | ↑ | 2.074 | 1.757 | 2.392 | 54 | ↑ |
| Cyprus | 75.992 | 51 | ↑ | 2.395 | 1.912 | 2.880 | 41 | ↑ |
| Czech Republic | 14.164 | 144 | ↑ | 0.355 | 0.203 | 0.508 | 146 | ↑ |
| Democratic Republic of the Congo | 1.952 | 166 | ↑ | 0.012 | -0.177 | 0.202 | 161 | ↑ |
| Denmark | 17.790 | 137 | ↑ | 0.439 | 0.275 | 0.603 | 144 | ↑ |
| Djibouti | -7.097 | 14 | ↓ | -0.516 | -0.660 | -0.371 | 17 | ↓ |
| Dominica | 89.025 | 38 | ↑ | 2.197 | 1.920 | 2.475 | 50 | ↑ |
| Dominican Republic | 19.848 | 127 | ↑ | -0.242 | -0.660 | 0.178 | 25 | ↓ |
| Ecuador | 25.719 | 120 | ↑ | 1.301 | 0.848 | 1.757 | 87 | ↑ |
| Egypt | 93.856 | 35 | ↑ | 2.542 | 2.433 | 2.652 | 36 | ↑ |
| El Salvador | 86.622 | 42 | ↑ | 1.418 | 1.108 | 1.730 | 81 | ↑ |
| Equatorial Guinea | 42.594 | 85 | ↑ | 1.715 | 1.543 | 1.887 | 69 | ↑ |
| Eritrea | 4.141 | 162 | ↑ | -0.312 | -0.434 | -0.190 | 23 | ↓ |
| Estonia | 75.212 | 53 | ↑ | 2.277 | 1.946 | 2.610 | 49 | ↑ |
| Ethiopia | -15.635 | 6 | ↓ | -0.858 | -0.989 | -0.726 | 7 | ↓ |
| Federated States of Micronesia | 23.511 | 122 | ↑ | 0.825 | 0.745 | 0.904 | 118 | ↑ |
| Fiji | 21.595 | 126 | ↑ | 1.123 | 0.741 | 1.507 | 99 | ↑ |
| Finland | 28.253 | 112 | ↑ | 0.769 | 0.539 | 0.999 | 124 | ↑ |
| France | 36.883 | 96 | ↑ | 1.407 | 1.248 | 1.566 | 82 | ↑ |
| Gabon | 8.857 | 155 | ↑ | 0.228 | 0.082 | 0.375 | 151 | ↑ |
| Georgia | 157.628 | 8 | ↑ | 3.567 | 2.488 | 4.657 | 11 | ↑ |
| Germany | 2.648 | 164 | ↑ | -0.136 | -0.542 | 0.272 | 27 | ↓ |
| Ghana | 28.619 | 110 | ↑ | 0.862 | 0.813 | 0.910 | 116 | ↑ |
| Greece | 103.624 | 24 | ↑ | 3.201 | 2.920 | 3.482 | 21 | ↑ |
| Greenland | 25.914 | 119 | ↑ | 0.644 | 0.324 | 0.965 | 128 | ↑ |
| Grenada | 82.277 | 49 | ↑ | 2.591 | 2.425 | 2.758 | 35 | ↑ |
| Guam | 16.984 | 141 | ↑ | 0.612 | 0.320 | 0.904 | 131 | ↑ |
| Guatemala | 42.117 | 86 | ↑ | -0.283 | -0.866 | 0.304 | 24 | ↓ |
| Guinea | 17.402 | 138 | ↑ | 0.668 | 0.597 | 0.739 | 127 | ↑ |
| Guinea-Bissau | 23.155 | 123 | ↑ | 0.996 | 0.891 | 1.100 | 105 | ↑ |
| Guyana | 87.139 | 41 | ↑ | 1.894 | 1.447 | 2.343 | 59 | ↑ |
| Haiti | 18.866 | 131 | ↑ | 0.622 | 0.516 | 0.728 | 130 | ↑ |
| Honduras | 124.593 | 14 | ↑ | 2.815 | 2.570 | 3.060 | 29 | ↑ |
| Hungary | -3.878 | 20 | ↓ | -0.696 | -1.250 | -0.140 | 13 | ↓ |
| Iceland | -5.873 | 17 | ↓ | -0.434 | -0.656 | -0.211 | 19 | ↓ |
| India | 36.616 | 98 | ↑ | 0.975 | 0.516 | 1.435 | 108 | ↑ |
| Indonesia | 10.689 | 153 | ↑ | 0.326 | 0.249 | 0.403 | 148 | ↑ |
| Iran | 125.166 | 13 | ↑ | 3.790 | 3.221 | 4.361 | 8 | ↑ |
| Iraq | 18.783 | 133 | ↑ | 0.641 | 0.429 | 0.855 | 129 | ↑ |
| Ireland | 68.082 | 58 | ↑ | 2.831 | 2.492 | 3.172 | 28 | ↑ |
| Israel | 101.458 | 26 | ↑ | 2.527 | 2.126 | 2.929 | 37 | ↑ |
| Italy | 232.786 | 3 | ↑ | 3.451 | 2.598 | 4.312 | 13 | ↑ |
| Jamaica | 193.511 | 6 | ↑ | 3.722 | 3.233 | 4.213 | 10 | ↑ |
| Japan | 109.176 | 19 | ↑ | 3.233 | 2.905 | 3.562 | 20 | ↑ |
| Jordan | 47.232 | 76 | ↑ | 0.857 | 0.527 | 1.189 | 117 | ↑ |
| Kazakhstan | 19.515 | 128 | ↑ | 0.222 | -0.246 | 0.693 | 153 | ↑ |
| Kenya | 17.021 | 140 | ↑ | 0.555 | 0.495 | 0.616 | 134 | ↑ |
| Kiribati | 1.601 | 167 | ↑ | 0.293 | 0.109 | 0.477 | 150 | ↑ |
| Kuwait | 36.827 | 97 | ↑ | 1.987 | 1.318 | 2.661 | 56 | ↑ |
| Kyrgyzstan | 44.001 | 82 | ↑ | 1.548 | 1.136 | 1.962 | 77 | ↑ |
| Laos | -6.842 | 15 | ↓ | -0.422 | -0.585 | -0.260 | 20 | ↓ |
| Latvia | 108.133 | 22 | ↑ | 3.274 | 2.926 | 3.624 | 18 | ↑ |
| Lebanon | 143.325 | 10 | ↑ | 3.504 | 3.360 | 3.648 | 12 | ↑ |
| Lesotho | 37.073 | 95 | ↑ | 1.555 | 1.228 | 1.882 | 76 | ↑ |
| Liberia | 27.001 | 115 | ↑ | 1.300 | 1.075 | 1.526 | 88 | ↑ |
| Libya | 103.545 | 25 | ↑ | 3.068 | 2.755 | 3.382 | 24 | ↑ |
| Lithuania | 87.446 | 40 | ↑ | 1.962 | 1.690 | 2.235 | 57 | ↑ |
| Luxembourg | 34.192 | 99 | ↑ | 0.900 | 0.644 | 1.156 | 112 | ↑ |
| Macedonia | 145.959 | 9 | ↑ | 3.834 | 3.406 | 4.263 | 7 | ↑ |
| Madagascar | -14.207 | 7 | ↓ | -0.745 | -0.851 | -0.639 | 11 | ↓ |
| Malawi | -1.559 | 22 | ↓ | -0.625 | -0.905 | -0.343 | 14 | ↓ |
| Malaysia | 71.099 | 55 | ↑ | 2.386 | 2.047 | 2.726 | 43 | ↑ |
| Maldives | 11.018 | 152 | ↑ | 0.026 | -0.194 | 0.246 | 159 | ↑ |
| Mali | 2.274 | 165 | ↑ | 0.150 | 0.027 | 0.273 | 154 | ↑ |
| Malta | 68.654 | 57 | ↑ | 1.830 | 1.673 | 1.987 | 64 | ↑ |
| Marshall Islands | 40.828 | 88 | ↑ | 1.279 | 1.210 | 1.348 | 90 | ↑ |
| Mauritania | 25.072 | 121 | ↑ | 0.995 | 0.893 | 1.098 | 106 | ↑ |
| Mauritius | -1.662 | 21 | ↓ | -0.869 | -1.130 | -0.606 | 6 | ↓ |
| Mexico | 101.293 | 27 | ↑ | 3.003 | 2.756 | 3.250 | 25 | ↑ |
| Moldova | 43.115 | 84 | ↑ | 0.907 | 0.572 | 1.243 | 110 | ↑ |
| Mongolia | 47.719 | 75 | ↑ | 1.252 | 0.808 | 1.697 | 92 | ↑ |
| Montenegro | 56.828 | 68 | ↑ | 1.700 | 1.548 | 1.851 | 71 | ↑ |
| Morocco | 61.586 | 64 | ↑ | 1.881 | 1.788 | 1.974 | 61 | ↑ |
| Mozambique | -1.304 | 24 | ↓ | -0.093 | -0.336 | 0.150 | 30 | ↓ |
| Myanmar | 1.167 | 169 | ↑ | -0.047 | -0.176 | 0.082 | 32 | ↓ |
| Namibia | -6.786 | 16 | ↓ | -0.940 | -1.427 | -0.451 | 5 | ↓ |
| Nepal | -4.339 | 19 | ↓ | -0.110 | -0.554 | 0.336 | 29 | ↓ |
| Netherlands | 60.539 | 65 | ↑ | 1.832 | 1.715 | 1.950 | 63 | ↑ |
| New Zealand | 7.123 | 157 | ↑ | 0.340 | 0.251 | 0.430 | 147 | ↑ |
| Nicaragua | 31.508 | 101 | ↑ | 1.812 | 1.239 | 2.388 | 65 | ↑ |
| Niger | 13.918 | 145 | ↑ | 0.586 | 0.500 | 0.673 | 133 | ↑ |
| Nigeria | 25.931 | 118 | ↑ | 0.988 | 0.869 | 1.107 | 107 | ↑ |
| North Korea | 0.016 | 171 | ↑ | -0.017 | -0.300 | 0.267 | 33 | ↓ |
| Northern Mariana Islands | 38.775 | 93 | ↑ | 1.359 | 0.910 | 1.810 | 85 | ↑ |
| Norway | 39.669 | 90 | ↑ | 1.259 | 0.945 | 1.575 | 91 | ↑ |
| Oman | 99.721 | 29 | ↑ | 2.515 | 2.336 | 2.694 | 38 | ↑ |
| Pakistan | 40.069 | 89 | ↑ | 1.090 | 1.017 | 1.162 | 100 | ↑ |
| Palestine | 22.056 | 124 | ↑ | 0.789 | 0.555 | 1.023 | 121 | ↑ |
| Panama | 112.987 | 17 | ↑ | 3.358 | 3.094 | 3.622 | 14 | ↑ |
| Papua New Guinea | 13.401 | 146 | ↑ | 0.537 | 0.490 | 0.584 | 135 | ↑ |
| Paraguay | 5.635 | 160 | ↑ | -0.801 | -1.117 | -0.483 | 8 | ↓ |
| Peru | 38.662 | 94 | ↑ | 0.526 | 0.048 | 1.006 | 136 | ↑ |
| Philippines | 5.795 | 159 | ↑ | -0.436 | -0.757 | -0.113 | 18 | ↓ |
| Poland | 109.971 | 18 | ↑ | 3.167 | 3.018 | 3.317 | 22 | ↑ |
| Portugal | 38.923 | 92 | ↑ | 1.062 | 0.913 | 1.211 | 102 | ↑ |
| Puerto Rico | 86.195 | 43 | ↑ | 2.415 | 2.187 | 2.644 | 40 | ↑ |
| Qatar | 225.294 | 4 | ↑ | 4.868 | 4.341 | 5.397 | 3 | ↑ |
| Romania | 74.906 | 54 | ↑ | 1.960 | 1.800 | 2.120 | 58 | ↑ |
| Russian Federation | 12.979 | 148 | ↑ | 0.384 | 0.095 | 0.674 | 145 | ↑ |
| Rwanda | -19.720 | 4 | ↓ | -1.024 | -1.277 | -0.770 | 3 | ↓ |
| Saint Lucia | 67.331 | 59 | ↑ | 1.563 | 1.387 | 1.740 | 74 | ↑ |
| Saint Vincent and the Grenadines | 60.209 | 66 | ↑ | 1.357 | 1.141 | 1.573 | 86 | ↑ |
| Samoa | 14.849 | 143 | ↑ | 0.463 | 0.369 | 0.558 | 141 | ↑ |
| Sao Tome and Principe | 53.452 | 70 | ↑ | 1.599 | 1.537 | 1.662 | 73 | ↑ |
| Saudi Arabia | 234.377 | 2 | ↑ | 5.604 | 5.221 | 5.989 | 2 | ↑ |
| Senegal | 30.544 | 106 | ↑ | 1.141 | 1.047 | 1.236 | 98 | ↑ |
| Serbia | 94.511 | 34 | ↑ | 3.306 | 2.991 | 3.621 | 16 | ↑ |
| Seychelles | 26.459 | 116 | ↑ | 0.496 | 0.314 | 0.678 | 139 | ↑ |
| Sierra Leone | 44.293 | 81 | ↑ | 1.713 | 1.589 | 1.837 | 70 | ↑ |
| Singapore | 119.090 | 16 | ↑ | 2.912 | 2.388 | 3.439 | 26 | ↑ |
| Slovakia | 50.688 | 72 | ↑ | 1.539 | 1.434 | 1.644 | 78 | ↑ |
| Slovenia | 26.204 | 117 | ↑ | 0.592 | 0.421 | 0.762 | 132 | ↑ |
| Solomon Islands | 5.086 | 161 | ↑ | 0.100 | 0.032 | 0.168 | 156 | ↑ |
| Somalia | -10.389 | 10 | ↓ | -0.700 | -0.873 | -0.528 | 12 | ↓ |
| South Africa | 43.902 | 83 | ↑ | 1.191 | 0.914 | 1.469 | 97 | ↑ |
| South Korea | -22.800 | 3 | ↓ | -0.329 | -1.172 | 0.521 | 22 | ↓ |
| South Sudan | -17.708 | 5 | ↓ | -1.018 | -1.182 | -0.853 | 4 | ↓ |
| Spain | 44.412 | 80 | ↑ | 1.630 | 1.481 | 1.779 | 72 | ↑ |
| Sri Lanka | 100.084 | 28 | ↑ | 3.305 | 3.097 | 3.513 | 17 | ↑ |
| Sudan | 31.963 | 100 | ↑ | 1.212 | 1.008 | 1.416 | 95 | ↑ |
| Suriname | 77.173 | 50 | ↑ | 1.885 | 1.661 | 2.111 | 60 | ↑ |
| Swaziland | 12.640 | 149 | ↑ | 0.319 | 0.069 | 0.569 | 149 | ↑ |
| Sweden | 8.238 | 156 | ↑ | 0.025 | -0.224 | 0.276 | 160 | ↑ |
| Switzerland | 6.771 | 158 | ↑ | 0.109 | -0.222 | 0.441 | 155 | ↑ |
| Syria | 64.175 | 62 | ↑ | 1.803 | 1.332 | 2.275 | 66 | ↑ |
| Taiwan | 246.967 | 1 | ↑ | 6.529 | 5.948 | 7.114 | 1 | ↑ |
| Tajikistan | 191.058 | 7 | ↑ | 4.451 | 3.960 | 4.944 | 5 | ↑ |
| Tanzania | -9.866 | 12 | ↓ | -0.757 | -1.045 | -0.468 | 10 | ↓ |
| Thailand | 44.496 | 79 | ↑ | 0.912 | 0.729 | 1.095 | 109 | ↑ |
| The Bahamas | 66.810 | 60 | ↑ | 1.771 | 1.408 | 2.135 | 68 | ↑ |
| The Gambia | 27.689 | 113 | ↑ | 1.046 | 0.979 | 1.113 | 103 | ↑ |
| Timor-Leste | 18.067 | 135 | ↑ | 0.902 | 0.622 | 1.183 | 111 | ↑ |
| Togo | 18.864 | 132 | ↑ | 0.690 | 0.620 | 0.760 | 126 | ↑ |
| Tonga | 30.131 | 107 | ↑ | 1.007 | 0.942 | 1.072 | 104 | ↑ |
| Trinidad and Tobago | 75.720 | 52 | ↑ | 2.156 | 1.874 | 2.439 | 51 | ↑ |
| Tunisia | 84.728 | 47 | ↑ | 1.843 | 1.646 | 2.041 | 62 | ↑ |
| Turkey | 87.492 | 39 | ↑ | 2.673 | 2.442 | 2.905 | 33 | ↑ |
| Turkmenistan | -13.890 | 8 | ↓ | -0.791 | -1.686 | 0.112 | 9 | ↓ |
| Uganda | -5.589 | 18 | ↓ | -0.592 | -0.752 | -0.432 | 15 | ↓ |
| Ukraine | 108.703 | 20 | ↑ | 2.878 | 2.415 | 3.343 | 27 | ↑ |
| United Arab Emirates | 108.270 | 21 | ↑ | 2.299 | 1.900 | 2.701 | 48 | ↑ |
| United Kingdom | 91.933 | 36 | ↑ | 3.235 | 2.916 | 3.556 | 19 | ↑ |
| United States | 51.590 | 71 | ↑ | 1.431 | 1.338 | 1.524 | 80 | ↑ |
| Uruguay | 17.360 | 139 | ↑ | 0.453 | 0.316 | 0.591 | 142 | ↑ |
| Uzbekistan | 127.958 | 11 | ↑ | 4.253 | 3.670 | 4.840 | 6 | ↑ |
| Vanuatu | 17.927 | 136 | ↑ | 0.740 | 0.690 | 0.790 | 125 | ↑ |
| Venezuela | 19.036 | 129 | ↑ | 0.780 | 0.396 | 1.165 | 123 | ↑ |
| Vietnam | 64.476 | 61 | ↑ | 2.118 | 1.972 | 2.265 | 52 | ↑ |
| Virgin Islands, US | 85.939 | 44 | ↑ | 2.377 | 1.919 | 2.838 | 44 | ↑ |
| Yemen | 42.092 | 87 | ↑ | 1.506 | 1.416 | 1.596 | 79 | ↑ |
| Zambia | -29.680 | 1 | ↓ | -1.730 | -2.261 | -1.196 | 1 | ↓ |
| Zimbabwe | 29.410 | 109 | ↑ | 1.380 | 0.846 | 1.917 | 84 | ↑ |

a: percent change; b: annual percent change; c: confidence interval

# Supplemental Table 16. Trends in endometrial cancer age-standardized mortality rate by sociodemographic index and region 1990-2017

| **Characteristics** | **PC^a^ (%)** | | | **APC^b^ (%)** | | | | |
| --- | --- | --- | --- | --- | --- | --- | --- | --- |
|  | **Value** | **Rank** | | **Value** | **95%CI^c^** | **95%CI** | **Rank** | |
| Global | -25.775 |  |  | -1.189 | -1.238 | -1.140 |  |  |
| **Sociodemographic index** |  |  |  |  |  |  |  |  |
| Low | -25.647 | 3 | ↓ | -1.285 | -1.471 | -1.099 | 3 | ↓ |
| Low-middle | -13.307 | 4 | ↓ | -0.764 | -0.863 | -0.665 | 4 | ↓ |
| Middle | -34.057 | 2 | ↓ | -1.618 | -1.708 | -1.527 | 2 | ↓ |
| High-Middle | -36.789 | 1 | ↓ | -1.890 | -2.037 | -1.743 | 1 | ↓ |
| High | -10.913 | 5 | ↓ | -0.288 | -0.400 | -0.175 | 5 | ↓ |
| **Region** |  |  |  |  |  |  |  |  |
| Central Asia | 0.158 | 6 | ↑ | 0.022 | -0.096 | 0.140 | 6 | ↑ |
| East Asia | -52.027 | 1 | ↓ | -2.621 | -2.835 | -2.406 | 1 | ↓ |
| High-income Asia Pacific | -31.044 | 3 | ↓ | -1.001 | -1.240 | -0.761 | 8 | ↓ |
| South Asia | -14.634 | 11 | ↓ | -0.834 | -1.040 | -0.627 | 11 | ↓ |
| Southeast Asia | -23.021 | 7 | ↓ | -1.126 | -1.241 | -1.010 | 7 | ↓ |
| Central Europe | -13.723 | 13 | ↓ | -0.447 | -0.626 | -0.269 | 14 | ↓ |
| Eastern Europe | -11.982 | 14 | ↓ | -0.858 | -1.178 | -0.538 | 10 | ↓ |
| Western Europe | -7.193 | 15 | ↓ | -0.117 | -0.213 | -0.021 | 15 | ↓ |
| Andean Latin America | -34.865 | 2 | ↓ | -1.771 | -1.890 | -1.651 | 2 | ↓ |
| Central Latin America | -25.384 | 6 | ↓ | -1.166 | -1.414 | -0.918 | 5 | ↓ |
| Southern Latin America | -26.450 | 5 | ↓ | -1.330 | -1.496 | -1.163 | 4 | ↓ |
| Tropical Latin America | -21.147 | 8 | ↓ | -1.135 | -1.250 | -1.020 | 6 | ↓ |
| High income North America | 2.903 | 4 | ↑ | 0.180 | 0.067 | 0.292 | 3 | ↑ |
| Central Sub-Saharan Africa | -13.957 | 12 | ↓ | -0.603 | -0.653 | -0.553 | 13 | ↓ |
| Eastern Sub-Saharan Africa | -30.354 | 4 | ↓ | -1.661 | -1.817 | -1.505 | 3 | ↓ |
| Southern Sub-Saharan Africa | 18.643 | 1 | ↑ | 0.828 | 0.315 | 1.344 | 1 | ↑ |
| Western Sub-Saharan Africa | 1.103 | 5 | ↑ | 0.066 | 0.031 | 0.100 | 4 | ↑ |
| North Africa and Middle East | -20.505 | 9 | ↓ | -0.888 | -0.999 | -0.777 | 9 | ↓ |
| Oceania | 4.656 | 3 | ↑ | 0.375 | 0.279 | 0.471 | 2 | ↑ |
| Australasia | -15.125 | 10 | ↓ | -0.681 | -0.767 | -0.594 | 12 | ↓ |
| Caribbean | 5.510 | 2 | ↑ | 0.024 | -0.129 | 0.177 | 5 | ↑ |

a: percent change; b: annual percent change; c: confidence interval

# Supplemental Table 17. The relative contributions of each geographical locations in trends of endometrial cancer mortality from 1990-2017

|  | **Increasing trend** | **Decreasing trend** |
| --- | --- | --- |
| **Characteristics** | **Contribution rate (%)** | **Contribution rate (%)** |
| **Sociodemographic index** |  |  |
| Low | - | 21.99 |
| Low-middle | - | 13.08 |
| Middle | - | 27.68 |
| Middle-High | - | 32.34 |
| High | - | 4.92 |
| **Region** |  |  |
| Central Asia | 1.46 | - |
| Eastern Asia | - | 16.14 |
| High-income Asia Pacific | - | 6.16 |
| South Asia | - | 5.13 |
| Southeast Asia | - | 6.93 |
| Central Europe | - | 2.75 |
| Eastern Europe | - | 5.28 |
| Western Europe | - | 0.72 |
| Andean Latin America | - | 10.91 |
| Central Latin America | - | 7.18 |
| Southern Latin America | - | 8.19 |
| Tropical Latin America | - | 6.99 |
| North America | 12.03 | - |
| Central Sub-Saharan Africa | - | 3.71 |
| Eastern Sub-Saharan Africa | - | 10.23 |
| Southern Sub-Saharan Africa | 55.43 | - |
| Western Sub-Saharan Africa | 4.40 | - |
| North Africa and Middle East | - | 5.47 |
| Oceania | 25.10 | - |
| Australasia | - | 4.19 |
| Caribbean | 1.59 | - |

# Supplemental Table 18. Trends in endometrial cancer age-standardized mortality rate of 195 countries and territories from 1990-2017

| **Countries and territories** | **PC^a^** | | | **APC^b^** | | | | |
| --- | --- | --- | --- | --- | --- | --- | --- | --- |
|  | **Value** | **Rank** | | **Value** | **95%CI^c^** | **95%CI** | **Rank** | |
| Afghanistan | -3.387 | 113 | ↓ | -0.007 | -0.070 | 0.056 | 120 | ↓ |
| Albania | -12.606 | 94 | ↓ | -0.406 | -0.601 | -0.211 | 96 | ↓ |
| Algeria | -12.965 | 92 | ↓ | -0.156 | -0.300 | -0.012 | 110 | ↓ |
| American Samoa | 38.659 | 7 | ↑ | 1.894 | 1.502 | 2.286 | 5 | ↑ |
| Andorra | -0.774 | 127 | ↓ | 0.111 | -0.220 | 0.444 | 70 | ↑ |
| Angola | -22.884 | 57 | ↓ | -1.071 | -1.166 | -0.975 | 59 | ↓ |
| Antigua and Barbuda | 43.290 | 5 | ↑ | 0.966 | 0.696 | 1.237 | 22 | ↑ |
| Argentina | -29.583 | 34 | ↓ | -1.586 | -1.764 | -1.408 | 33 | ↓ |
| Armenia | 37.823 | 9 | ↑ | 1.584 | 1.253 | 1.916 | 8 | ↑ |
| Australia | -13.525 | 91 | ↓ | -0.685 | -0.792 | -0.578 | 78 | ↓ |
| Austria | -34.178 | 22 | ↓ | -1.548 | -1.647 | -1.449 | 36 | ↓ |
| Azerbaijan | 2.514 | 62 | ↑ | -0.215 | -0.425 | -0.004 | 104 | ↓ |
| Bahrain | -20.153 | 69 | ↓ | -1.466 | -1.738 | -1.193 | 39 | ↓ |
| Bangladesh | -50.497 | 6 | ↓ | -2.411 | -2.580 | -2.242 | 11 | ↓ |
| Barbados | 18.605 | 32 | ↑ | 0.645 | 0.433 | 0.858 | 38 | ↑ |
| Belarus | -20.677 | 65 | ↓ | -0.706 | -1.440 | 0.033 | 76 | ↓ |
| Belgium | -12.607 | 93 | ↓ | -0.596 | -0.766 | -0.425 | 84 | ↓ |
| Belize | -2.060 | 120 | ↓ | -0.195 | -0.498 | 0.109 | 107 | ↓ |
| Benin | 11.102 | 44 | ↑ | 0.628 | 0.548 | 0.708 | 41 | ↑ |
| Bermuda | -36.286 | 19 | ↓ | -2.083 | -2.504 | -1.660 | 19 | ↓ |
| Bhutan | -44.233 | 8 | ↓ | -2.398 | -2.560 | -2.237 | 12 | ↓ |
| Bolivia | -24.151 | 52 | ↓ | -1.125 | -1.192 | -1.058 | 54 | ↓ |
| Bosnia and Herzegovina | 36.957 | 10 | ↑ | 1.415 | 1.059 | 1.773 | 14 | ↑ |
| Botswana | 11.587 | 43 | ↑ | 1.313 | 0.840 | 1.789 | 16 | ↑ |
| Brazil | -20.586 | 66 | ↓ | -1.088 | -1.201 | -0.976 | 58 | ↓ |
| Brunei | -19.661 | 71 | ↓ | -0.216 | -0.700 | 0.270 | 103 | ↓ |
| Bulgaria | 4.188 | 54 | ↑ | 0.811 | 0.465 | 1.158 | 28 | ↑ |
| Burkina Faso | -2.889 | 117 | ↓ | 0.034 | -0.163 | 0.232 | 75 | ↑ |
| Burundi | -38.401 | 15 | ↓ | -2.195 | -2.394 | -1.996 | 14 | ↓ |
| Cambodia | -29.407 | 35 | ↓ | -1.404 | -1.478 | -1.329 | 42 | ↓ |
| Cameroon | -2.576 | 119 | ↓ | -0.009 | -0.149 | 0.132 | 119 | ↓ |
| Canada | -20.156 | 68 | ↓ | -0.633 | -0.926 | -0.340 | 81 | ↓ |
| Cape Verde | 28.836 | 18 | ↑ | 0.777 | 0.593 | 0.961 | 30 | ↑ |
| Central African Republic | -12.138 | 97 | ↓ | -0.493 | -0.563 | -0.423 | 92 | ↓ |
| Chad | 19.597 | 29 | ↑ | 0.912 | 0.815 | 1.009 | 23 | ↑ |
| Chile | -1.555 | 122 | ↓ | 0.153 | -0.056 | 0.363 | 63 | ↑ |
| China | -53.553 | 4 | ↓ | -2.735 | -2.963 | -2.507 | 6 | ↓ |
| Colombia | -45.566 | 7 | ↓ | -2.621 | -2.956 | -2.286 | 8 | ↓ |
| Comoros | -30.371 | 30 | ↓ | -1.568 | -1.706 | -1.431 | 34 | ↓ |
| Congo | -10.660 | 100 | ↓ | -0.557 | -0.674 | -0.440 | 89 | ↓ |
| Costa Rica | 12.518 | 41 | ↑ | 0.274 | 0.070 | 0.480 | 53 | ↑ |
| Cote d'Ivoire | -0.630 | 128 | ↓ | -0.058 | -0.197 | 0.080 | 115 | ↓ |
| Croatia | -2.910 | 116 | ↓ | 0.356 | 0.053 | 0.660 | 51 | ↑ |
| Cuba | 22.600 | 27 | ↑ | 0.447 | 0.183 | 0.711 | 48 | ↑ |
| Cyprus | -26.252 | 41 | ↓ | -1.123 | -1.277 | -0.969 | 55 | ↓ |
| Czech Republic | -38.672 | 14 | ↓ | -1.822 | -1.895 | -1.749 | 23 | ↓ |
| Democratic Republic of the Congo | -11.239 | 98 | ↓ | -0.472 | -0.578 | -0.366 | 93 | ↓ |
| Denmark | -25.449 | 47 | ↓ | -1.521 | -1.804 | -1.237 | 37 | ↓ |
| Djibouti | -25.663 | 45 | ↓ | -1.416 | -1.539 | -1.292 | 41 | ↓ |
| Dominica | 34.682 | 12 | ↑ | 1.026 | 0.916 | 1.137 | 21 | ↑ |
| Dominican Republic | -42.833 | 10 | ↓ | -2.569 | -2.879 | -2.258 | 9 | ↓ |
| Ecuador | -37.369 | 17 | ↓ | -1.320 | -1.768 | -0.870 | 45 | ↓ |
| Egypt | -6.584 | 107 | ↓ | -0.156 | -0.258 | -0.054 | 111 | ↓ |
| El Salvador | -22.036 | 58 | ↓ | -1.737 | -2.002 | -1.470 | 27 | ↓ |
| Equatorial Guinea | -29.344 | 36 | ↓ | -1.248 | -1.362 | -1.133 | 50 | ↓ |
| Eritrea | -19.194 | 73 | ↓ | -0.934 | -0.989 | -0.879 | 69 | ↓ |
| Estonia | -9.997 | 101 | ↓ | -0.600 | -0.886 | -0.313 | 82 | ↓ |
| Ethiopia | -40.041 | 11 | ↓ | -2.185 | -2.295 | -2.076 | 15 | ↓ |
| Federated States of Micronesia | -5.621 | 109 | ↓ | -0.200 | -0.228 | -0.172 | 105 | ↓ |
| Fiji | 11.060 | 45 | ↑ | 0.763 | 0.510 | 1.018 | 32 | ↑ |
| Finland | -14.241 | 89 | ↓ | -0.572 | -0.693 | -0.450 | 86 | ↓ |
| France | -21.361 | 60 | ↓ | -0.659 | -0.872 | -0.445 | 79 | ↓ |
| Gabon | -17.116 | 80 | ↓ | -0.729 | -1.036 | -0.420 | 74 | ↓ |
| Georgia | 96.285 | 2 | ↑ | 3.008 | 2.008 | 4.019 | 1 | ↑ |
| Germany | -29.789 | 33 | ↓ | -1.445 | -1.824 | -1.064 | 40 | ↓ |
| Ghana | -2.911 | 115 | ↓ | -0.173 | -0.235 | -0.110 | 109 | ↓ |
| Greece | 29.306 | 17 | ↑ | 1.449 | 1.143 | 1.756 | 12 | ↑ |
| Greenland | -21.163 | 62 | ↓ | -0.973 | -1.206 | -0.739 | 66 | ↓ |
| Grenada | 27.814 | 19 | ↑ | 1.271 | 0.984 | 1.559 | 18 | ↑ |
| Guam | 2.239 | 64 | ↑ | 0.501 | -0.028 | 1.033 | 47 | ↑ |
| Guatemala | -30.099 | 31 | ↓ | -2.875 | -3.419 | -2.327 | 5 | ↓ |
| Guinea | 2.804 | 60 | ↑ | 0.169 | 0.097 | 0.242 | 60 | ↑ |
| Guinea-Bissau | 7.690 | 49 | ↑ | 0.520 | 0.411 | 0.629 | 44 | ↑ |
| Guyana | 27.664 | 20 | ↑ | 0.631 | 0.216 | 1.048 | 40 | ↑ |
| Haiti | -7.623 | 104 | ↓ | -0.255 | -0.283 | -0.227 | 102 | ↓ |
| Honduras | 30.958 | 14 | ↑ | 0.859 | 0.687 | 1.032 | 25 | ↑ |
| Hungary | -50.582 | 5 | ↓ | -3.207 | -3.830 | -2.581 | 4 | ↓ |
| Iceland | -34.612 | 21 | ↓ | -1.743 | -1.858 | -1.627 | 26 | ↓ |
| India | -13.878 | 90 | ↓ | -0.843 | -1.148 | -0.538 | 72 | ↓ |
| Indonesia | -17.574 | 78 | ↓ | -0.725 | -0.821 | -0.629 | 75 | ↓ |
| Iran | 23.117 | 25 | ↑ | 1.541 | 0.863 | 2.224 | 9 | ↑ |
| Iraq | -16.493 | 81 | ↓ | -0.815 | -1.034 | -0.596 | 73 | ↓ |
| Ireland | -15.406 | 84 | ↓ | 0.156 | -0.194 | 0.506 | 62 | ↑ |
| Israel | 23.567 | 24 | ↑ | 0.820 | 0.536 | 1.105 | 26 | ↑ |
| Italy | 79.465 | 3 | ↑ | 1.355 | 0.616 | 2.098 | 15 | ↑ |
| Jamaica | 99.314 | 1 | ↑ | 2.469 | 2.021 | 2.919 | 2 | ↑ |
| Japan | -12.392 | 95 | ↓ | -0.152 | -0.312 | 0.009 | 112 | ↓ |
| Jordan | -19.708 | 70 | ↓ | -1.296 | -1.700 | -0.891 | 46 | ↓ |
| Kazakhstan | -28.778 | 37 | ↓ | -1.931 | -2.248 | -1.613 | 20 | ↓ |
| Kenya | -9.878 | 102 | ↓ | -0.466 | -0.520 | -0.412 | 95 | ↓ |
| Kiribati | -1.407 | 123 | ↓ | 0.178 | 0.043 | 0.314 | 59 | ↑ |
| Kuwait | -17.482 | 79 | ↓ | 0.144 | -0.562 | 0.854 | 64 | ↑ |
| Kyrgyzstan | -0.970 | 125 | ↓ | 0.185 | -0.188 | 0.560 | 58 | ↑ |
| Laos | -34.659 | 20 | ↓ | -1.748 | -1.831 | -1.664 | 25 | ↓ |
| Latvia | 31.883 | 13 | ↑ | 1.208 | 0.898 | 1.518 | 20 | ↑ |
| Lebanon | -21.744 | 59 | ↓ | -1.064 | -1.297 | -0.830 | 61 | ↓ |
| Lesotho | 38.291 | 8 | ↑ | 1.810 | 1.288 | 2.334 | 7 | ↑ |
| Liberia | 9.468 | 47 | ↑ | 0.669 | 0.472 | 0.865 | 37 | ↑ |
| Libya | 3.721 | 57 | ↑ | 0.156 | -0.007 | 0.319 | 61 | ↑ |
| Lithuania | 30.609 | 15 | ↑ | 0.259 | -0.085 | 0.604 | 56 | ↑ |
| Luxembourg | -20.853 | 63 | ↓ | -0.854 | -1.009 | -0.699 | 71 | ↓ |
| Macedonia | 17.392 | 36 | ↑ | 0.820 | 0.508 | 1.133 | 27 | ↑ |
| Madagascar | -25.957 | 43 | ↓ | -1.291 | -1.413 | -1.170 | 47 | ↓ |
| Malawi | -24.507 | 49 | ↓ | -1.774 | -2.099 | -1.448 | 24 | ↓ |
| Malaysia | -15.695 | 82 | ↓ | -0.555 | -0.796 | -0.313 | 90 | ↓ |
| Maldives | -56.577 | 2 | ↓ | -3.707 | -3.957 | -3.456 | 2 | ↓ |
| Mali | -25.851 | 44 | ↓ | -1.068 | -1.212 | -0.923 | 60 | ↓ |
| Malta | -6.043 | 108 | ↓ | -0.396 | -0.481 | -0.311 | 98 | ↓ |
| Marshall Islands | 14.837 | 40 | ↑ | 0.519 | 0.317 | 0.721 | 45 | ↑ |
| Mauritania | -9.323 | 103 | ↓ | -0.198 | -0.290 | -0.105 | 106 | ↓ |
| Mauritius | -33.171 | 26 | ↓ | -1.909 | -2.085 | -1.733 | 21 | ↓ |
| Mexico | -18.543 | 74 | ↓ | -0.383 | -0.727 | -0.038 | 100 | ↓ |
| Moldova | -1.257 | 124 | ↓ | -0.559 | -0.960 | -0.156 | 88 | ↓ |
| Mongolia | -15.367 | 86 | ↓ | -1.230 | -1.716 | -0.742 | 51 | ↓ |
| Montenegro | -5.087 | 111 | ↓ | -0.386 | -0.564 | -0.209 | 99 | ↓ |
| Morocco | -12.386 | 96 | ↓ | -0.470 | -0.504 | -0.436 | 94 | ↓ |
| Mozambique | -21.314 | 61 | ↓ | -0.938 | -1.166 | -0.708 | 68 | ↓ |
| Myanmar | -33.392 | 25 | ↓ | -1.615 | -1.793 | -1.437 | 31 | ↓ |
| Namibia | -33.612 | 24 | ↓ | -2.087 | -2.746 | -1.423 | 18 | ↓ |
| Nepal | -34.122 | 23 | ↓ | -1.608 | -2.003 | -1.211 | 32 | ↓ |
| Netherlands | 3.941 | 56 | ↑ | 0.084 | -0.050 | 0.217 | 72 | ↑ |
| New Zealand | -20.318 | 67 | ↓ | -0.634 | -0.706 | -0.561 | 80 | ↓ |
| Nicaragua | -32.667 | 27 | ↓ | -1.132 | -1.679 | -0.582 | 53 | ↓ |
| Niger | -3.067 | 114 | ↓ | -0.040 | -0.130 | 0.050 | 117 | ↓ |
| Nigeria | -7.034 | 106 | ↓ | -0.330 | -0.392 | -0.268 | 101 | ↓ |
| North Korea | 2.330 | 63 | ↑ | 0.063 | -0.077 | 0.204 | 73 | ↑ |
| Northern Mariana Islands | 4.327 | 53 | ↑ | 0.750 | 0.445 | 1.056 | 33 | ↑ |
| Norway | -24.214 | 51 | ↓ | -1.000 | -1.152 | -0.847 | 65 | ↓ |
| Oman | -17.750 | 77 | ↓ | -0.701 | -0.854 | -0.547 | 77 | ↓ |
| Pakistan | 15.152 | 39 | ↑ | 0.264 | 0.097 | 0.430 | 55 | ↑ |
| Palestine | -5.617 | 110 | ↓ | 0.114 | -0.127 | 0.355 | 69 | ↑ |
| Panama | 9.509 | 46 | ↑ | 0.892 | 0.631 | 1.154 | 24 | ↑ |
| Papua New Guinea | 2.796 | 61 | ↑ | 0.288 | 0.186 | 0.390 | 52 | ↑ |
| Paraguay | -31.446 | 28 | ↓ | -2.130 | -2.382 | -1.877 | 17 | ↓ |
| Peru | -38.270 | 16 | ↓ | -2.398 | -2.771 | -2.023 | 13 | ↓ |
| Philippines | -19.246 | 72 | ↓ | -1.393 | -1.746 | -1.039 | 43 | ↓ |
| Poland | 6.118 | 51 | ↑ | 0.512 | 0.333 | 0.692 | 46 | ↑ |
| Portugal | -28.702 | 39 | ↓ | -1.278 | -1.406 | -1.150 | 48 | ↓ |
| Puerto Rico | -10.831 | 99 | ↓ | -0.399 | -0.535 | -0.263 | 97 | ↓ |
| Qatar | 19.229 | 30 | ↑ | 0.632 | 0.209 | 1.056 | 39 | ↑ |
| Romania | -20.761 | 64 | ↓ | -1.122 | -1.369 | -0.875 | 56 | ↓ |
| Russian Federation | -23.581 | 54 | ↓ | -1.366 | -1.749 | -0.983 | 44 | ↓ |
| Rwanda | -43.803 | 9 | ↓ | -2.634 | -2.877 | -2.390 | 7 | ↓ |
| Saint Lucia | 3.068 | 58 | ↑ | -0.149 | -0.317 | 0.020 | 113 | ↓ |
| Saint Vincent and the Grenadines | 16.255 | 38 | ↑ | 0.224 | -0.055 | 0.503 | 57 | ↑ |
| Samoa | 1.446 | 65 | ↑ | 0.059 | -0.067 | 0.185 | 74 | ↑ |
| Sao Tome and Principe | 23.971 | 22 | ↑ | 0.737 | 0.687 | 0.787 | 34 | ↑ |
| Saudi Arabia | 23.651 | 23 | ↑ | 1.420 | 1.093 | 1.749 | 13 | ↑ |
| Senegal | 11.652 | 42 | ↑ | 0.602 | 0.507 | 0.698 | 42 | ↑ |
| Serbia | 22.097 | 28 | ↑ | 1.294 | 1.066 | 1.523 | 17 | ↑ |
| Seychelles | -23.904 | 53 | ↓ | -1.187 | -1.381 | -0.993 | 52 | ↓ |
| Sierra Leone | 25.777 | 21 | ↑ | 1.219 | 1.083 | 1.355 | 19 | ↑ |
| Singapore | -14.659 | 87 | ↓ | -0.566 | -0.809 | -0.323 | 87 | ↓ |
| Slovakia | -25.168 | 48 | ↓ | -1.089 | -1.168 | -1.010 | 57 | ↓ |
| Slovenia | -29.904 | 32 | ↓ | -1.715 | -1.897 | -1.533 | 28 | ↓ |
| Solomon Islands | 0.111 | 67 | ↑ | 0.121 | 0.010 | 0.231 | 68 | ↑ |
| Somalia | -18.178 | 75 | ↓ | -1.021 | -1.164 | -0.877 | 64 | ↓ |
| South Africa | 17.800 | 33 | ↑ | 0.684 | 0.206 | 1.165 | 36 | ↑ |
| South Korea | -74.234 | 1 | ↓ | -5.068 | -5.808 | -4.321 | 1 | ↓ |
| South Sudan | -26.596 | 40 | ↓ | -1.490 | -1.673 | -1.307 | 38 | ↓ |
| Spain | -7.073 | 105 | ↓ | -0.102 | -0.193 | -0.011 | 114 | ↓ |
| Sri Lanka | 3.985 | 55 | ↑ | 0.765 | 0.463 | 1.067 | 31 | ↑ |
| Sudan | -15.613 | 83 | ↓ | -0.505 | -0.625 | -0.385 | 91 | ↓ |
| Suriname | 17.764 | 35 | ↑ | 0.423 | 0.112 | 0.735 | 49 | ↑ |
| Swaziland | -2.654 | 118 | ↓ | 0.142 | -0.420 | 0.707 | 65 | ↑ |
| Sweden | -17.781 | 76 | ↓ | -0.591 | -0.703 | -0.479 | 85 | ↓ |
| Switzerland | -23.343 | 55 | ↓ | -0.964 | -1.187 | -0.740 | 67 | ↓ |
| Syria | -24.267 | 50 | ↓ | -1.047 | -1.552 | -0.540 | 62 | ↓ |
| Taiwan | 29.600 | 16 | ↑ | 2.142 | 1.743 | 2.542 | 4 | ↑ |
| Tajikistan | 69.042 | 4 | ↑ | 2.180 | 1.740 | 2.622 | 3 | ↑ |
| Tanzania | -28.741 | 38 | ↓ | -1.670 | -1.929 | -1.409 | 30 | ↓ |
| Thailand | -31.258 | 29 | ↓ | -1.845 | -2.006 | -1.684 | 22 | ↓ |
| The Bahamas | 16.745 | 37 | ↑ | 0.570 | 0.375 | 0.765 | 43 | ↑ |
| The Gambia | 7.085 | 50 | ↑ | 0.391 | 0.335 | 0.447 | 50 | ↑ |
| Timor-Leste | -23.170 | 56 | ↓ | -1.029 | -1.138 | -0.920 | 63 | ↓ |
| Togo | 0.900 | 66 | ↑ | 0.085 | 0.056 | 0.115 | 71 | ↑ |
| Tonga | 2.953 | 59 | ↑ | 0.130 | 0.039 | 0.221 | 66 | ↑ |
| Trinidad and Tobago | 9.046 | 48 | ↑ | 0.125 | -0.091 | 0.342 | 67 | ↑ |
| Tunisia | -15.382 | 85 | ↓ | -0.886 | -1.029 | -0.743 | 70 | ↓ |
| Turkey | -40.014 | 12 | ↓ | -2.138 | -2.297 | -1.979 | 16 | ↓ |
| Turkmenistan | -55.641 | 3 | ↓ | -3.464 | -4.194 | -2.728 | 3 | ↓ |
| Uganda | -25.644 | 46 | ↓ | -1.551 | -1.747 | -1.355 | 35 | ↓ |
| Ukraine | 36.372 | 11 | ↑ | 0.796 | 0.445 | 1.149 | 29 | ↑ |
| United Arab Emirates | -0.878 | 126 | ↓ | -0.053 | -0.274 | 0.169 | 116 | ↓ |
| United Kingdom | 22.835 | 26 | ↑ | 1.533 | 1.210 | 1.858 | 10 | ↑ |
| United States | 5.673 | 52 | ↑ | 0.267 | 0.166 | 0.367 | 54 | ↑ |
| Uruguay | -26.160 | 42 | ↓ | -1.264 | -1.385 | -1.143 | 49 | ↓ |
| Uzbekistan | 18.974 | 31 | ↑ | 1.501 | 1.053 | 1.950 | 11 | ↑ |
| Vanuatu | 17.765 | 34 | ↑ | 0.696 | 0.582 | 0.811 | 35 | ↑ |
| Venezuela | -36.740 | 18 | ↓ | -1.677 | -2.104 | -1.249 | 29 | ↓ |
| Vietnam | -14.383 | 88 | ↓ | -0.597 | -0.634 | -0.561 | 83 | ↓ |
| Virgin Islands, US | -1.839 | 121 | ↓ | -0.016 | -0.110 | 0.078 | 118 | ↓ |
| Yemen | -4.819 | 112 | ↓ | -0.181 | -0.217 | -0.145 | 108 | ↓ |
| Zambia | -39.705 | 13 | ↓ | -2.543 | -2.975 | -2.109 | 10 | ↓ |
| Zimbabwe | 42.044 | 6 | ↑ | 1.870 | 0.940 | 2.808 | 6 | ↑ |

a: percent change; b: annual percent change; c: confidence interval

# Supplemental Table 19. Trends in endometrial cancer age-standardized DALYs^a^ rate by sociodemographic index and region 1990-2017

| **Characteristics** | **PC^b^(%)** | | | **APC^c^ (%)** | | | | |
| --- | --- | --- | --- | --- | --- | --- | --- | --- |
|  | **Value** | **Rank** | | **Value** | **95%CI^d^** | **95%CI** | **Rank** | |
| Global | -25.635 |  |  | -1.205 | -1.262 | -1.147 |  |  |
| **Sociodemographic index** |  |  |  |  |  |  |  |  |
| Low | -25.927 | 3 | ↓ | -1.328 | -1.525 | -1.130 | 3 | ↓ |
| Low-middle | -12.685 | 4 | ↓ | -0.762 | -0.873 | -0.651 | 4 | ↓ |
| Middle | -35.067 | 2 | ↓ | -1.697 | -1.804 | -1.589 | 2 | ↓ |
| High-Middle | -38.093 | 1 | ↓ | -1.982 | -2.130 | -1.835 | 1 | ↓ |
| High | -4.789 | 5 | ↓ | -0.015 | -0.129 | 0.100 | 5 | ↓ |
| **Region** |  |  |  |  |  |  |  |  |
| Central Asia | 4.444 | 5 | ↑ | 0.147 | 0.015 | 0.279 | 4 | ↑ |
| East Asia | -52.026 | 1 | ↓ | -2.665 | -2.882 | -2.448 | 1 | ↓ |
| High-income Asia Pacific | -24.667 | 5 | ↓ | -0.565 | -0.833 | -0.296 | 13 | ↓ |
| South Asia | -12.059 | 14 | ↓ | -0.748 | -0.966 | -0.529 | 9 | ↓ |
| Southeast Asia | -22.865 | 6 | ↓ | -1.122 | -1.259 | -0.985 | 5 | ↓ |
| Central Europe | -13.754 | 11 | ↓ | -0.463 | -0.638 | -0.289 | 14 | ↓ |
| Eastern Europe | -12.231 | 13 | ↓ | -0.924 | -1.281 | -0.565 | 7 | ↓ |
| Western Europe | -2.935 | 15 | ↓ | 0.050 | -0.046 | 0.147 | 5 | ↑ |
| Andean Latin America | -35.387 | 2 | ↓ | -1.762 | -1.882 | -1.643 | 3 | ↓ |
| Central Latin America | -21.046 | 7 | ↓ | -0.906 | -1.174 | -0.637 | 8 | ↓ |
| Southern Latin America | -28.830 | 4 | ↓ | -1.432 | -1.664 | -1.200 | 4 | ↓ |
| Tropical Latin America | -18.106 | 8 | ↓ | -1.003 | -1.124 | -0.882 | 6 | ↓ |
| High income North America | 12.413 | 2 | ↑ | 0.525 | 0.434 | 0.615 | 2 | ↑ |
| Central Sub-Saharan Africa | -16.091 | 10 | ↓ | -0.729 | -0.785 | -0.673 | 11 | ↓ |
| Eastern Sub-Saharan Africa | -32.212 | 3 | ↓ | -1.807 | -1.982 | -1.632 | 2 | ↓ |
| Southern Sub-Saharan Africa | 13.629 | 1 | ↑ | 0.581 | 0.013 | 1.152 | 1 | ↑ |
| Western Sub-Saharan Africa | -2.488 | 16 | ↓ | -0.103 | -0.139 | -0.066 | 15 | ↓ |
| North Africa and Middle East | -17.732 | 9 | ↓ | -0.743 | -0.864 | -0.623 | 10 | ↓ |
| Oceania | 4.785 | 4 | ↑ | 0.389 | 0.288 | 0.490 | 3 | ↑ |
| Australasia | -13.447 | 12 | ↓ | -0.619 | -0.704 | -0.533 | 12 | ↓ |
| Caribbean | 6.085 | 3 | ↑ | -0.002 | -0.176 | 0.173 | 16 | ↓ |

a: disability adjusted life years b: percent change.

c: annual percent change d: confidence interval

Supplemental Table 20. The relative contributions of each geographical locations in trends of endometrial cancer DALYs^a^ from 1990-2017

|  | **Increasing trend** | **Decreasing trend** |
| --- | --- | --- |
| **Characteristics** | **Contribution rate (%)** | **Contribution rate (%)** |
| **Sociodemographic index** |  |  |
| Low | - | 22.95 |
| Low-middle | - | 13.18 |
| Middle | - | 29.34 |
| Middle-High | - | 34.27 |
| High | - | 0.26 |
| **Region** |  |  |
| Central Asia | 8.69 | - |
| Eastern Asia |  | 17.09 |
| High-income Asia Pacific | - | 3.62 |
| South Asia | - | 4.80 |
| Southeast Asia | - | 7.20 |
| Central Europe | - | 2.97 |
| Eastern Europe | - | 5.92 |
| Western Europe | 2.97 | - |
| Andean Latin America | - | 11.30 |
| Central Latin America | - | 5.81 |
| Southern Latin America | - | 9.18 |
| Tropical Latin America | - | 6.43 |
| North America | 31.01 |  |
| Central Sub-Saharan Africa | - | 4.68 |
| Eastern Sub-Saharan Africa | - | 11.59 |
| Southern Sub-Saharan Africa | 34.33 | - |
| Western Sub-Saharan Africa | - | 0.66 |
| North Africa and Middle East | - | 4.77 |
| Oceania | 23.00 | - |
| Australasia | - | 3.97 |
| Caribbean | - | 0.01 |

# a: disability adjusted life years

# **Supplemental Table 21. Trends in endometrial cancer age-standardized DALYs^a^ rate of 195 countries and territories from 1990-2017**

| **Countries and territories** | **PC^b^** | | | **APC^c^** | | | | |
| --- | --- | --- | --- | --- | --- | --- | --- | --- |
|  | **Value** | **Rank** | | **Value** | **95%CI^d^** | **95%CI** | **Rank** | |
| Afghanistan | -3.692 | 114 | ↓ | -0.022 | -0.089 | 0.044 | 118 | ↓ |
| Albania | -11.613 | 92 | ↓ | -0.276 | -0.457 | -0.095 | 99 | ↓ |
| Algeria | -11.359 | 94 | ↓ | -0.072 | 1.728 | 2.500 | 115 | ↓ |
| American Samoa | 46.463 | 6 | ↑ | 2.113 | -2.081 | -1.232 | 6 | ↑ |
| Andorra | 5.058 | 58 | ↑ | 0.279 | -0.180 | 0.541 | 56 | ↑ |
| Angola | -25.786 | 49 | ↓ | -1.244 | -1.344 | -1.144 | 51 | ↓ |
| Antigua and Barbuda | 34.344 | 13 | ↑ | 0.752 | -0.163 | -0.089 | 32 | ↑ |
| Argentina | -32.963 | 25 | ↓ | -1.770 | -2.010 | -1.528 | 25 | ↓ |
| Armenia | 30.228 | 18 | ↑ | 1.326 | -1.307 | -1.018 | 15 | ↑ |
| Australia | -11.566 | 93 | ↓ | -0.610 | 1.016 | 1.637 | 80 | ↓ |
| Austria | -32.274 | 30 | ↓ | -1.451 | -1.593 | -1.069 | 41 | ↓ |
| Azerbaijan | 8.526 | 49 | ↑ | 0.000 | -1.530 | 0.053 | 119 | ↓ |
| Bahrain | -17.539 | 74 | ↓ | -1.331 | -0.728 | -0.425 | 47 | ↓ |
| Bangladesh | -50.708 | 5 | ↓ | -2.396 | 0.425 | 0.548 | 14 | ↓ |
| Barbados | 19.566 | 31 | ↑ | 0.658 | -1.957 | -1.570 | 35 | ↑ |
| Belarus | -21.731 | 59 | ↓ | -0.742 | -2.690 | -1.912 | 76 | ↓ |
| Belgium | -13.395 | 84 | ↓ | -0.577 | -1.285 | -1.165 | 84 | ↓ |
| Belize | 4.915 | 59 | ↑ | -0.029 | 1.141 | 1.879 | 117 | ↓ |
| Benin | 8.629 | 47 | ↑ | 0.486 | -0.168 | -0.109 | 45 | ↑ |
| Bermuda | -38.820 | 15 | ↓ | -2.302 | -2.628 | -2.193 | 17 | ↓ |
| Bhutan | -45.313 | 8 | ↓ | -2.492 | -1.490 | -1.355 | 10 | ↓ |
| Bolivia | -26.141 | 48 | ↓ | -1.225 | -0.361 | -0.057 | 52 | ↓ |
| Bosnia and Herzegovina | 40.051 | 9 | ↑ | 1.510 | -0.572 | -0.322 | 13 | ↑ |
| Botswana | 8.155 | 50 | ↑ | 1.267 | 0.524 | 0.665 | 16 | ↑ |
| Brazil | -17.500 | 75 | ↓ | -0.953 | 0.332 | 0.597 | 66 | ↓ |
| Brunei | -15.807 | 77 | ↓ | -0.065 | -0.697 | -0.523 | 116 | ↓ |
| Bulgaria | 5.607 | 55 | ↑ | 0.892 | -0.716 | -0.505 | 26 | ↑ |
| Burkina Faso | -5.627 | 110 | ↓ | -0.105 | 0.680 | 0.861 | 109 | ↓ |
| Burundi | -41.323 | 12 | ↓ | -2.411 | 0.156 | 0.633 | 13 | ↓ |
| Cambodia | -29.700 | 34 | ↓ | -1.423 | -3.022 | -2.562 | 43 | ↓ |
| Cameroon | -6.286 | 107 | ↓ | -0.209 | 0.185 | 0.611 | 103 | ↓ |
| Canada | -12.390 | 90 | ↓ | -0.226 | -1.914 | -1.406 | 101 | ↓ |
| Cape Verde | 17.729 | 35 | ↑ | 0.464 | -1.881 | -1.731 | 46 | ↑ |
| Central African Republic | -14.364 | 81 | ↓ | -0.610 | 0.906 | 1.126 | 81 | ↓ |
| Chad | 16.020 | 37 | ↑ | 0.771 | -0.585 | -0.121 | 31 | ↑ |
| Chile | 2.485 | 65 | ↑ | 0.394 | -0.502 | -0.435 | 51 | ↑ |
| China | -53.685 | 3 | ↓ | -2.792 | -2.858 | -2.180 | 6 | ↓ |
| Colombia | -44.162 | 9 | ↓ | -2.472 | -1.889 | -0.901 | 11 | ↓ |
| Comoros | -32.641 | 29 | ↓ | -1.692 | -0.249 | -0.019 | 32 | ↓ |
| Congo | -13.033 | 86 | ↓ | -0.704 | -1.986 | -1.451 | 77 | ↓ |
| Costa Rica | 14.462 | 40 | ↑ | 0.398 | -1.969 | -1.530 | 50 | ↑ |
| Cote d'Ivoire | -1.339 | 120 | ↓ | -0.096 | -1.591 | -1.300 | 110 | ↓ |
| Croatia | -0.823 | 122 | ↓ | 0.453 | -0.204 | 0.204 | 47 | ↑ |
| Cuba | 23.345 | 26 | ↑ | 0.398 | -0.302 | 0.093 | 49 | ↑ |
| Cyprus | -20.846 | 62 | ↓ | -0.781 | -0.882 | -0.256 | 74 | ↓ |
| Czech Republic | -38.196 | 16 | ↓ | -1.806 | 0.503 | 1.047 | 23 | ↓ |
| Democratic Republic of the Congo | -13.178 | 85 | ↓ | -0.589 | -0.738 | -0.424 | 82 | ↓ |
| Denmark | -28.511 | 37 | ↓ | -1.660 | -0.680 | -0.249 | 36 | ↓ |
| Djibouti | -27.287 | 43 | ↓ | -1.513 | -1.168 | -0.518 | 38 | ↓ |
| Dominica | 35.017 | 12 | ↑ | 1.016 | 0.757 | 1.779 | 24 | ↑ |
| Dominican Republic | -40.264 | 13 | ↓ | -2.520 | -1.671 | -0.878 | 9 | ↓ |
| Ecuador | -39.300 | 14 | ↓ | -1.396 | -0.257 | -0.141 | 44 | ↓ |
| Egypt | -6.236 | 108 | ↓ | -0.134 | -1.372 | -0.875 | 107 | ↓ |
| El Salvador | -21.702 | 60 | ↓ | -1.719 | 0.887 | 1.467 | 29 | ↓ |
| Equatorial Guinea | -32.748 | 26 | ↓ | -1.446 | -3.247 | -2.192 | 42 | ↓ |
| Eritrea | -22.188 | 56 | ↓ | -1.102 | 0.057 | 0.218 | 61 | ↓ |
| Estonia | -6.771 | 106 | ↓ | -0.569 | 0.269 | 0.466 | 85 | ↓ |
| Ethiopia | -42.938 | 10 | ↓ | -2.424 | 0.293 | 1.166 | 12 | ↓ |
| Federated States of Micronesia | -4.566 | 111 | ↓ | -0.139 | -0.404 | -0.343 | 106 | ↓ |
| Fiji | 11.128 | 44 | ↑ | 0.775 | -2.014 | -1.674 | 30 | ↑ |
| Finland | -14.189 | 82 | ↓ | -0.581 | 0.503 | 0.887 | 83 | ↓ |
| France | -16.469 | 76 | ↓ | -0.464 | -1.805 | -1.588 | 89 | ↓ |
| Gabon | -19.587 | 68 | ↓ | -0.844 | -1.181 | -0.515 | 70 | ↓ |
| Georgia | 96.583 | 3 | ↑ | 2.960 | 0.512 | 1.136 | 2 | ↑ |
| Germany | -26.724 | 45 | ↓ | -1.275 | 0.809 | 2.176 | 49 | ↓ |
| Ghana | -3.677 | 115 | ↓ | -0.199 | -0.003 | 0.644 | 105 | ↓ |
| Greece | 38.377 | 10 | ↑ | 1.701 | -1.217 | -1.067 | 9 | ↑ |
| Greenland | -22.998 | 55 | ↓ | -1.124 | 1.035 | 2.496 | 59 | ↓ |
| Grenada | 23.661 | 24 | ↑ | 1.177 | -0.842 | -0.565 | 17 | ↑ |
| Guam | 5.506 | 56 | ↑ | 0.589 | 0.548 | 1.116 | 40 | ↑ |
| Guatemala | -27.924 | 40 | ↓ | -2.721 | 2.108 | 3.001 | 8 | ↓ |
| Guinea | 1.875 | 66 | ↑ | 0.137 | -0.062 | 0.236 | 68 | ↑ |
| Guinea-Bissau | 4.130 | 60 | ↑ | 0.368 | -3.183 | -2.270 | 52 | ↑ |
| Guyana | 30.660 | 17 | ↑ | 0.729 | -0.945 | -0.617 | 33 | ↑ |
| Haiti | -10.557 | 98 | ↓ | -0.373 | -2.376 | -1.721 | 93 | ↓ |
| Honduras | 24.912 | 22 | ↑ | 0.695 | -0.001 | 0.559 | 34 | ↑ |
| Hungary | -49.607 | 6 | ↓ | -3.167 | -0.543 | -0.397 | 4 | ↓ |
| Iceland | -33.733 | 24 | ↓ | -1.697 | -0.713 | 0.738 | 31 | ↓ |
| India | -12.942 | 87 | ↓ | -0.848 | -0.272 | 0.582 | 69 | ↓ |
| Indonesia | -19.457 | 69 | ↓ | -0.812 | -1.805 | -1.623 | 73 | ↓ |
| Iran | 21.666 | 28 | ↑ | 1.490 | 0.747 | 1.417 | 14 | ↑ |
| Iraq | -14.986 | 78 | ↓ | -0.817 | -1.215 | -0.750 | 72 | ↓ |
| Ireland | -11.292 | 95 | ↓ | 0.320 | -0.028 | 0.537 | 54 | ↑ |
| Israel | 23.550 | 25 | ↑ | 0.832 | 1.622 | 2.634 | 27 | ↑ |
| Italy | 95.213 | 4 | ↑ | 1.763 | -1.471 | -1.162 | 8 | ↑ |
| Jamaica | 105.191 | 1 | ↑ | 2.554 | 0.136 | 0.771 | 4 | ↑ |
| Japan | 3.832 | 62 | ↑ | 0.548 | 0.432 | 0.884 | 41 | ↑ |
| Jordan | -26.250 | 47 | ↓ | -1.657 | 1.116 | 2.218 | 37 | ↓ |
| Kazakhstan | -28.884 | 35 | ↓ | -2.049 | 0.107 | 0.365 | 19 | ↓ |
| Kenya | -9.376 | 101 | ↓ | -0.470 | 0.866 | 1.478 | 87 | ↓ |
| Kiribati | -3.792 | 113 | ↓ | 0.087 | -2.084 | -1.247 | 71 | ↑ |
| Kuwait | -19.782 | 66 | ↓ | 0.010 | 0.814 | 1.371 | 76 | ↑ |
| Kyrgyzstan | -0.400 | 123 | ↓ | 0.154 | 0.083 | 0.406 | 65 | ↑ |
| Laos | -33.958 | 23 | ↓ | -1.714 | -4.011 | -3.551 | 30 | ↓ |
| Latvia | 30.952 | 16 | ↑ | 1.081 | -3.777 | -2.553 | 22 | ↑ |
| Lebanon | -20.384 | 65 | ↓ | -0.983 | -0.310 | -0.113 | 64 | ↓ |
| Lesotho | 33.463 | 14 | ↑ | 1.665 | -1.840 | -1.544 | 10 | ↑ |
| Liberia | 5.797 | 54 | ↑ | 0.500 | -0.583 | 0.612 | 43 | ↑ |
| Libya | 6.297 | 52 | ↑ | 0.236 | 0.978 | 1.236 | 62 | ↑ |
| Lithuania | 28.077 | 20 | ↑ | 0.180 | 0.050 | 0.473 | 63 | ↑ |
| Luxembourg | -18.069 | 72 | ↓ | -0.769 | 0.366 | 0.841 | 75 | ↓ |
| Macedonia | 27.231 | 21 | ↑ | 1.172 | -2.853 | -2.090 | 18 | ↑ |
| Madagascar | -27.614 | 42 | ↓ | -1.390 | -0.448 | -0.284 | 45 | ↓ |
| Malawi | -24.443 | 53 | ↓ | -1.836 | -2.168 | -1.786 | 22 | ↓ |
| Malaysia | -12.591 | 89 | ↓ | -0.306 | -0.220 | 0.515 | 97 | ↓ |
| Maldives | -58.153 | 2 | ↓ | -3.782 | -0.980 | -0.104 | 2 | ↓ |
| Mali | -26.954 | 44 | ↓ | -1.163 | -1.712 | -0.732 | 55 | ↓ |
| Malta | -1.352 | 119 | ↓ | -0.212 | -1.307 | -0.815 | 102 | ↓ |
| Marshall Islands | 19.196 | 32 | ↑ | 0.603 | -0.237 | 0.046 | 37 | ↑ |
| Mauritania | -12.772 | 88 | ↓ | -0.366 | -1.868 | -1.487 | 94 | ↓ |
| Mauritius | -32.747 | 27 | ↓ | -1.977 | -3.049 | -1.649 | 20 | ↓ |
| Mexico | -8.489 | 102 | ↓ | 0.147 | 0.534 | 0.970 | 67 | ↑ |
| Moldova | -0.084 | 125 | ↓ | -0.543 | -2.070 | -1.258 | 86 | ↓ |
| Mongolia | -14.368 | 80 | ↓ | -1.223 | 0.147 | 0.387 | 53 | ↓ |
| Montenegro | -4.171 | 112 | ↓ | -0.354 | -0.350 | -0.177 | 95 | ↓ |
| Morocco | -12.375 | 91 | ↓ | -0.468 | -0.460 | -0.318 | 88 | ↓ |
| Mozambique | -23.162 | 54 | ↓ | -1.061 | 0.032 | 0.283 | 62 | ↓ |
| Myanmar | -34.450 | 22 | ↓ | -1.678 | -1.053 | -0.721 | 33 | ↓ |
| Namibia | -37.270 | 18 | ↓ | -2.352 | -0.586 | -0.309 | 15 | ↓ |
| Nepal | -34.988 | 21 | ↓ | -1.665 | -0.055 | 0.314 | 35 | ↓ |
| Netherlands | 8.116 | 51 | ↑ | 0.267 | -2.578 | -2.215 | 57 | ↑ |
| New Zealand | -19.623 | 67 | ↓ | -0.636 | -0.300 | 0.116 | 79 | ↓ |
| Nicaragua | -32.744 | 28 | ↓ | -1.149 | 0.136 | 0.353 | 56 | ↓ |
| Niger | -7.055 | 105 | ↓ | -0.263 | -2.412 | -1.891 | 100 | ↓ |
| Nigeria | -7.730 | 103 | ↓ | -0.389 | -2.684 | -1.950 | 92 | ↓ |
| North Korea | 4.068 | 61 | ↑ | 0.158 | -0.353 | 0.297 | 64 | ↑ |
| Northern Mariana Islands | 6.110 | 53 | ↑ | 0.824 | -2.193 | -1.478 | 28 | ↑ |
| Norway | -21.763 | 58 | ↓ | -0.887 | -1.737 | -0.933 | 67 | ↓ |
| Oman | -13.643 | 83 | ↓ | -0.447 | -1.394 | -1.135 | 90 | ↓ |
| Pakistan | 12.815 | 42 | ↑ | 0.130 | 0.540 | 0.766 | 70 | ↑ |
| Palestine | -10.382 | 99 | ↓ | -0.092 | -0.116 | 0.149 | 112 | ↓ |
| Panama | 13.879 | 41 | ↑ | 1.092 | -0.531 | 0.403 | 21 | ↑ |
| Papua New Guinea | 1.559 | 67 | ↑ | 0.245 | 0.493 | 1.293 | 60 | ↑ |
| Paraguay | -31.236 | 31 | ↓ | -2.152 | 0.668 | 1.477 | 18 | ↓ |
| Peru | -37.747 | 17 | ↓ | -2.318 | -1.343 | -0.864 | 16 | ↓ |
| Philippines | -17.759 | 73 | ↓ | -1.336 | -1.922 | -1.078 | 46 | ↓ |
| Poland | -0.052 | 126 | ↓ | 0.244 | -1.159 | -1.045 | 61 | ↑ |
| Portugal | -27.968 | 39 | ↓ | -1.264 | -3.205 | -2.630 | 50 | ↓ |
| Puerto Rico | -0.196 | 124 | ↓ | 0.016 | 0.387 | 0.709 | 74 | ↑ |
| Qatar | 29.234 | 19 | ↑ | 1.072 | -1.576 | -1.326 | 23 | ↑ |
| Romania | -19.432 | 70 | ↓ | -1.104 | -0.396 | -0.018 | 60 | ↓ |
| Russian Federation | -25.246 | 51 | ↓ | -1.501 | -0.204 | 0.049 | 40 | ↓ |
| Rwanda | -46.774 | 7 | ↓ | -2.918 | 1.343 | 1.974 | 5 | ↓ |
| Saint Lucia | 1.467 | 68 | ↑ | -0.207 | 0.417 | 0.565 | 104 | ↓ |
| Saint Vincent and the Grenadines | 21.403 | 29 | ↑ | 0.254 | 0.893 | 1.322 | 59 | ↑ |
| Samoa | -2.104 | 118 | ↓ | -0.077 | -1.366 | -1.032 | 114 | ↓ |
| Sao Tome and Principe | 20.110 | 30 | ↑ | 0.595 | -0.221 | 0.076 | 39 | ↑ |
| Saudi Arabia | 31.420 | 15 | ↑ | 1.658 | -1.072 | -0.834 | 11 | ↑ |
| Senegal | 10.217 | 45 | ↑ | 0.491 | 0.091 | 1.089 | 44 | ↑ |
| Serbia | 18.422 | 34 | ↑ | 1.107 | -0.913 | -0.625 | 19 | ↑ |
| Seychelles | -25.054 | 52 | ↓ | -1.199 | -0.571 | -0.027 | 54 | ↓ |
| Sierra Leone | 22.924 | 27 | ↑ | 1.107 | -2.659 | -2.325 | 20 | ↑ |
| Singapore | -9.644 | 100 | ↓ | -0.299 | -0.021 | 0.194 | 98 | ↓ |
| Slovakia | -25.733 | 50 | ↓ | -1.142 | -1.294 | -0.973 | 57 | ↓ |
| Slovenia | -30.555 | 32 | ↓ | -1.763 | -0.244 | 0.821 | 26 | ↓ |
| Solomon Islands | -0.895 | 121 | ↓ | 0.086 | 1.441 | 1.962 | 72 | ↑ |
| Somalia | -19.393 | 71 | ↓ | -1.133 | -1.707 | -1.298 | 58 | ↓ |
| South Africa | 9.637 | 46 | ↑ | 0.288 | 0.083 | 0.714 | 55 | ↑ |
| South Korea | -74.758 | 1 | ↓ | -5.053 | -0.206 | 0.022 | 1 | ↓ |
| South Sudan | -26.421 | 46 | ↓ | -1.503 | 0.480 | 1.117 | 39 | ↓ |
| Spain | -7.209 | 104 | ↓ | -0.092 | 0.033 | 0.699 | 111 | ↓ |
| Sri Lanka | 5.251 | 57 | ↑ | 0.798 | 1.871 | 4.062 | 29 | ↑ |
| Sudan | -14.816 | 79 | ↓ | -0.447 | -0.949 | -0.709 | 91 | ↓ |
| Suriname | 18.741 | 33 | ↑ | 0.365 | -1.523 | -1.258 | 53 | ↑ |
| Swaziland | -6.160 | 109 | ↓ | 0.013 | -0.703 | -0.475 | 75 | ↑ |
| Sweden | -20.515 | 64 | ↓ | -0.829 | -1.117 | -0.641 | 71 | ↓ |
| Switzerland | -20.725 | 63 | ↓ | -0.879 | -1.517 | -0.548 | 68 | ↓ |
| Syria | -21.378 | 61 | ↓ | -1.034 | 2.704 | 3.591 | 63 | ↓ |
| Taiwan | 61.641 | 5 | ↑ | 3.147 | -0.486 | 0.034 | 1 | ↑ |
| Tajikistan | 100.885 | 2 | ↑ | 2.810 | -1.954 | -1.571 | 3 | ↑ |
| Tanzania | -29.742 | 33 | ↓ | -1.787 | 2.411 | 3.209 | 24 | ↓ |
| Thailand | -28.648 | 36 | ↓ | -1.763 | -1.080 | -0.867 | 27 | ↓ |
| The Bahamas | 15.516 | 38 | ↑ | 0.528 | 0.340 | 0.716 | 42 | ↑ |
| The Gambia | 8.568 | 48 | ↑ | 0.434 | -2.070 | -1.503 | 48 | ↑ |
| Timor-Leste | -21.962 | 57 | ↓ | -0.973 | -0.114 | -0.058 | 65 | ↓ |
| Togo | -2.478 | 117 | ↓ | -0.086 | 0.063 | 0.244 | 113 | ↓ |
| Tonga | 3.764 | 63 | ↑ | 0.153 | -1.641 | -1.386 | 66 | ↑ |
| Trinidad and Tobago | 11.305 | 43 | ↑ | 0.261 | 0.313 | 0.688 | 58 | ↑ |
| Tunisia | -10.684 | 97 | ↓ | -0.703 | -0.826 | -0.580 | 78 | ↓ |
| Turkey | -35.878 | 20 | ↓ | -1.844 | -3.965 | -2.420 | 21 | ↓ |
| Turkmenistan | -52.330 | 4 | ↓ | -3.195 | 0.619 | 1.393 | 3 | ↓ |
| Uganda | -28.047 | 38 | ↓ | -1.750 | -0.167 | 0.240 | 28 | ↓ |
| Ukraine | 45.260 | 7 | ↑ | 1.005 | -0.709 | -0.564 | 25 | ↑ |
| United Arab Emirates | 2.650 | 64 | ↑ | 0.036 | -2.559 | -2.289 | 73 | ↑ |
| United Kingdom | 23.920 | 23 | ↑ | 1.565 | -1.041 | -0.591 | 12 | ↑ |
| United States | 15.322 | 39 | ↑ | 0.603 | -1.695 | -0.601 | 38 | ↑ |
| Uruguay | -27.635 | 41 | ↓ | -1.317 | 1.234 | 1.897 | 48 | ↓ |
| Uzbekistan | 38.336 | 11 | ↑ | 2.127 | -5.958 | -4.138 | 5 | ↑ |
| Vanuatu | 16.674 | 36 | ↑ | 0.653 | -0.934 | -0.690 | 36 | ↑ |
| Venezuela | -36.417 | 19 | ↓ | -1.666 | 0.523 | 0.682 | 34 | ↓ |
| Vietnam | -10.753 | 96 | ↓ | -0.344 | -0.396 | -0.292 | 96 | ↓ |
| Virgin Islands, US | 1.418 | 69 | ↑ | 0.132 | 0.374 | 0.494 | 69 | ↑ |
| Yemen | -3.563 | 116 | ↓ | -0.126 | 0.021 | 0.242 | 108 | ↓ |
| Zambia | -41.817 | 11 | ↓ | -2.727 | 0.911 | 2.916 | 7 | ↓ |
| Zimbabwe | 44.664 | 8 | ↑ | 1.908 | -0.564 | -0.047 | 7 | ↑ |

a: disability adjusted life years b: percent change.

c: annual percent change d: confidence interval

2

| **Characteristics** | **PC^b^(%)** | | | **APC^c^ (%)** | | | | |
| --- | --- | --- | --- | --- | --- | --- | --- | --- |
|  | **Value** | **Rank** | | **Value** | **95%CI^d^** | **95%CI** | **Rank** | |
| **Global** | 18.417 |  |  | 0.672 | 0.610 | 0.734 |  |  |
| **Sociodemographic index** |  |  |  |  |  |  |  |  |
| Low | -5.615 | 1 | ↓ | -0.377 | -0.618 | -0.135 | 1 | ↓ |
| Low-middle | 21.939 | 3 | ↑ | 0.556 | 0.441 | 0.672 | 3 | ↑ |
| Middle | 26.758 | 2 | ↑ | 0.928 | 0.808 | 1.047 | 2 | ↑ |
| High-Middle | 13.426 | 4 | ↑ | 0.529 | 0.411 | 0.647 | 4 | ↑ |
| High | 44.849 | 1 | ↑ | 1.466 | 1.407 | 1.525 | 1 | ↑ |
| **Region** |  |  |  |  |  |  |  |  |
| Central Asia | 53.043 | 5 | ↑ | 1.726 | 1.604 | 1.848 | 4 | ↑ |
| East Asia | 21.878 | 14 | ↑ | 1.024 | 0.765 | 1.284 | 10 | ↑ |
| High-income Asia Pacific | 56.444 | 3 | ↑ | 2.193 | 1.923 | 2.463 | 2 | ↑ |
| South Asia | 23.489 | 13 | ↑ | 0.616 | 0.283 | 0.951 | 14 | ↑ |
| Southeast Asia | 14.708 | 18 | ↑ | 0.351 | 0.258 | 0.444 | 19 | ↑ |
| Central Europe | 55.117 | 4 | ↑ | 1.854 | 1.725 | 1.984 | 3 | ↑ |
| Eastern Europe | 28.213 | 11 | ↑ | 0.818 | 0.575 | 1.062 | 11 | ↑ |
| Western Europe | 44.681 | 8 | ↑ | 1.488 | 1.427 | 1.549 | 7 | ↑ |
| Andean Latin America | 24.412 | 12 | ↑ | 0.607 | 0.367 | 0.847 | 15 | ↑ |
| Central Latin America | 50.545 | 6 | ↑ | 1.572 | 1.330 | 1.815 | 5 | ↑ |
| Southern Latin America | 18.820 | 17 | ↑ | 0.421 | 0.199 | 0.644 | 18 | ↑ |
| Tropical Latin America | 33.668 | 10 | ↑ | 0.789 | 0.687 | 0.891 | 12 | ↑ |
| High income North America | 45.310 | 7 | ↑ | 1.279 | 1.190 | 1.369 | 8 | ↑ |
| Central Sub-Saharan Africa | -0.234 | 2 | ↓ | -0.086 | -0.189 | 0.018 | 2 | ↓ |
| Eastern Sub-Saharan Africa | -15.692 | 1 | ↓ | -0.943 | -1.091 | -0.794 | 1 | ↓ |
| Southern Sub-Saharan Africa | 36.292 | 9 | ↑ | 1.130 | 0.753 | 1.508 | 9 | ↑ |
| Western Sub-Saharan Africa | 20.729 | 16 | ↑ | 0.745 | 0.727 | 0.764 | 13 | ↑ |
| North Africa and Middle East | 71.328 | 1 | ↑ | 2.247 | 2.116 | 2.379 | 1 | ↑ |
| Oceania | 13.591 | 19 | ↑ | 0.600 | 0.541 | 0.660 | 16 | ↑ |
| Australasia | 21.792 | 15 | ↑ | 0.574 | 0.469 | 0.679 | 17 | ↑ |
| Caribbean | 64.660 | 2 | ↑ | 1.533 | 1.288 | 1.779 | 6 | ↑ |

a: years lived with disability b: percent change.

c: annual percent change d: confidence interval

**Supplemental Table 23. The relative contributions of each geographical locations in trends of endometrial cancer YLDs^a^ from 1990-2017**

|  | **Increasing trend** | **Decreasing trend** |
| --- | --- | --- |
| **Characteristics** | **Contribution rate (%)** | **Contribution rate (%)** |
| **Sociodemographic index** |  |  |
| Low | - | 100.00 |
| Low-middle | 15.99 | - |
| Middle | 26.67 | - |
| Middle-High | 15.20 |  |
| High | 42.14 |  |
| **Region** |  |  |
| Central Asia | 8.00 | - |
| Eastern Asia | 4.75 | - |
| High-income Asia Pacific | 10.17 | - |
| South Asia | 2.86 | - |
| Southeast Asia | 1.63 | - |
| Central Europe | 8.60 | - |
| Eastern Europe | 3.79 |  |
| Western Europe | 6.90 |  |
| Andean Latin America | 2.81 | - |
| Central Latin America | 7.29 | - |
| Southern Latin America | 1.95 | - |
| Tropical Latin America | 3.66 | - |
| North America | 5.93 |  |
| Central Sub-Saharan Africa | - | 8.34 |
| Eastern Sub-Saharan Africa | - | 91.66 |
| Southern Sub-Saharan Africa | 5.24 | - |
| Western Sub-Saharan Africa | 3.46 | - |
| North Africa and Middle East | 10.42 | - |
| Oceania | 2.78 | - |
| Australasia | 2.66 | - |
| Caribbean | 7.11 | - |

a: years lived with disability

# Supplemental Table 24. Trends in endometrial cancer age-standardized YLDs^a^ rate of 195 countries and territories from 1990-2017

| **Countries and territories** | **PC^b^** | | | **APC^c^** | | | | |
| --- | --- | --- | --- | --- | --- | --- | --- | --- |
|  | **Value** | **Rank** | | **Value** | **95%CI^d^** | **95%CI** | **Rank** | |
| Afghanistan | 9.706 | 146 | ↑ | 0.580 | 0.449 | 0.711 | 124 | ↑ |
| Albania | 87.247 | 30 | ↑ | 2.470 | 2.228 | 2.712 | 32 | ↑ |
| Algeria | 57.756 | 60 | ↑ | 2.069 | 1.905 | 2.234 | 44 | ↑ |
| American Samoa | 87.224 | 31 | ↑ | 3.076 | 2.633 | 3.520 | 17 | ↑ |
| Andorra | 45.126 | 72 | ↑ | 1.310 | 1.222 | 1.397 | 77 | ↑ |
| Angola | -5.625 | 24 | ↓ | -0.351 | -0.424 | -0.277 | 29 | ↓ |
| Antigua and Barbuda | 98.262 | 21 | ↑ | 2.212 | 2.016 | 2.408 | 39 | ↑ |
| Argentina | 4.489 | 155 | ↑ | -0.250 | -0.499 | 0.000 | 33 | ↓ |
| Armenia | 111.329 | 14 | ↑ | 3.431 | 3.063 | 3.800 | 11 | ↑ |
| Australia | 26.441 | 101 | ↑ | 0.653 | 0.534 | 0.772 | 116 | ↑ |
| Austria | 0.191 | 163 | ↑ | -0.054 | -0.154 | 0.047 | 38 | ↓ |
| Azerbaijan | 82.657 | 36 | ↑ | 2.059 | 1.866 | 2.251 | 45 | ↑ |
| Bahrain | 61.869 | 55 | ↑ | 1.302 | 1.049 | 1.555 | 78 | ↑ |
| Bangladesh | -15.945 | 8 | ↓ | -0.262 | -0.545 | 0.022 | 32 | ↓ |
| Barbados | 77.539 | 42 | ↑ | 1.888 | 1.502 | 2.274 | 53 | ↑ |
| Belarus | 18.267 | 123 | ↑ | 0.664 | 0.016 | 1.315 | 114 | ↑ |
| Belgium | 25.812 | 103 | ↑ | 0.751 | 0.573 | 0.930 | 109 | ↑ |
| Belize | 38.971 | 78 | ↑ | 1.008 | 0.733 | 1.284 | 95 | ↑ |
| Benin | 26.025 | 102 | ↑ | 1.051 | 0.979 | 1.123 | 93 | ↑ |
| Bermuda | 20.496 | 117 | ↑ | 0.053 | -0.441 | 0.549 | 151 | ↑ |
| Bhutan | -10.069 | 18 | ↓ | -0.516 | -0.749 | -0.283 | 22 | ↓ |
| Bolivia | 20.620 | 114 | ↑ | 0.607 | 0.555 | 0.658 | 121 | ↑ |
| Bosnia and Herzegovina | 175.453 | 6 | ↑ | 4.207 | 3.625 | 4.793 | 4 | ↑ |
| Botswana | 49.947 | 68 | ↑ | 2.519 | 2.004 | 3.036 | 30 | ↑ |
| Brazil | 34.857 | 87 | ↑ | 0.844 | 0.747 | 0.942 | 104 | ↑ |
| Brunei | 44.808 | 73 | ↑ | 2.055 | 1.735 | 2.376 | 46 | ↑ |
| Bulgaria | 45.600 | 71 | ↑ | 2.000 | 1.527 | 2.474 | 51 | ↑ |
| Burkina Faso | 14.423 | 131 | ↑ | 0.649 | 0.500 | 0.799 | 117 | ↑ |
| Burundi | -28.588 | 3 | ↓ | -1.534 | -1.690 | -1.378 | 2 | ↓ |
| Cambodia | -4.081 | 28 | ↓ | -0.178 | -0.365 | 0.009 | 34 | ↓ |
| Cameroon | 8.882 | 149 | ↑ | 0.386 | 0.224 | 0.548 | 133 | ↑ |
| Canada | 34.453 | 91 | ↑ | 1.405 | 1.237 | 1.573 | 73 | ↑ |
| Cape Verde | 77.858 | 40 | ↑ | 2.217 | 2.114 | 2.319 | 38 | ↑ |
| Central African Republic | -11.093 | 16 | ↓ | -0.437 | -0.540 | -0.334 | 26 | ↓ |
| Chad | 26.760 | 100 | ↑ | 1.090 | 1.026 | 1.154 | 90 | ↑ |
| Chile | 99.596 | 19 | ↑ | 2.909 | 2.728 | 3.090 | 23 | ↑ |
| China | 19.422 | 119 | ↑ | 0.928 | 0.655 | 1.201 | 99 | ↑ |
| Colombia | 10.656 | 143 | ↑ | 0.206 | -0.226 | 0.640 | 147 | ↑ |
| Comoros | -14.375 | 10 | ↓ | -0.692 | -0.817 | -0.568 | 16 | ↓ |
| Congo | 9.018 | 148 | ↑ | 0.249 | 0.154 | 0.344 | 143 | ↑ |
| Costa Rica | 86.053 | 32 | ↑ | 2.505 | 2.247 | 2.764 | 31 | ↑ |
| Cote d'Ivoire | 10.887 | 142 | ↑ | 0.374 | 0.247 | 0.501 | 135 | ↑ |
| Croatia | 55.463 | 62 | ↑ | 2.421 | 2.106 | 2.737 | 34 | ↑ |
| Cuba | 89.299 | 26 | ↑ | 1.960 | 1.652 | 2.268 | 52 | ↑ |
| Cyprus | 64.820 | 52 | ↑ | 2.138 | 1.683 | 2.595 | 43 | ↑ |
| Czech Republic | 8.364 | 150 | ↑ | 0.157 | 0.002 | 0.313 | 149 | ↑ |
| Democratic Republic of the Congo | 0.432 | 161 | ↑ | -0.063 | -0.216 | 0.090 | 37 | ↓ |
| Denmark | 14.998 | 130 | ↑ | 0.304 | 0.127 | 0.481 | 140 | ↑ |
| Djibouti | -9.526 | 19 | ↓ | -0.631 | -0.766 | -0.495 | 19 | ↓ |
| Dominica | 79.611 | 39 | ↑ | 2.035 | 1.790 | 2.281 | 49 | ↑ |
| Dominican Republic | 13.765 | 133 | ↑ | -0.360 | -0.766 | 0.047 | 27 | ↓ |
| Ecuador | 18.449 | 122 | ↑ | 1.063 | 0.622 | 1.507 | 92 | ↑ |
| Egypt | 81.714 | 38 | ↑ | 2.283 | 2.174 | 2.392 | 36 | ↑ |
| El Salvador | 70.883 | 46 | ↑ | 1.169 | 0.881 | 1.459 | 86 | ↑ |
| Equatorial Guinea | 25.088 | 104 | ↑ | 1.193 | 1.009 | 1.378 | 85 | ↑ |
| Eritrea | -4.313 | 27 | ↓ | -0.491 | -0.593 | -0.389 | 23 | ↓ |
| Estonia | 65.080 | 51 | ↑ | 2.022 | 1.693 | 2.353 | 50 | ↑ |
| Ethiopia | -25.054 | 5 | ↓ | -1.386 | -1.507 | -1.263 | 4 | ↓ |
| Federated States of Micronesia | 17.298 | 124 | ↑ | 0.620 | 0.546 | 0.693 | 120 | ↑ |
| Fiji | 19.966 | 118 | ↑ | 1.042 | 0.691 | 1.394 | 94 | ↑ |
| Finland | 23.435 | 111 | ↑ | 0.631 | 0.418 | 0.845 | 119 | ↑ |
| France | 32.247 | 93 | ↑ | 1.264 | 1.106 | 1.422 | 83 | ↑ |
| Gabon | 3.629 | 157 | ↑ | 0.051 | -0.155 | 0.257 | 152 | ↑ |
| Georgia | 146.779 | 8 | ↑ | 3.452 | 2.409 | 4.505 | 10 | ↑ |
| Germany | -0.618 | 32 | ↓ | -0.272 | -0.681 | 0.140 | 31 | ↓ |
| Ghana | 24.253 | 109 | ↑ | 0.716 | 0.675 | 0.757 | 110 | ↑ |
| Greece | 96.677 | 22 | ↑ | 3.041 | 2.762 | 3.320 | 19 | ↑ |
| Greenland | 16.063 | 125 | ↑ | 0.306 | -0.006 | 0.618 | 138 | ↑ |
| Grenada | 74.543 | 45 | ↑ | 2.442 | 2.241 | 2.643 | 33 | ↑ |
| Guam | 15.910 | 126 | ↑ | 0.582 | 0.285 | 0.881 | 123 | ↑ |
| Guatemala | 30.337 | 94 | ↑ | -0.545 | -1.082 | -0.006 | 20 | ↓ |
| Guinea | 13.827 | 132 | ↑ | 0.548 | 0.497 | 0.599 | 126 | ↑ |
| Guinea-Bissau | 18.977 | 120 | ↑ | 0.888 | 0.785 | 0.990 | 101 | ↑ |
| Guyana | 77.652 | 41 | ↑ | 1.689 | 1.235 | 2.145 | 60 | ↑ |
| Haiti | 12.474 | 138 | ↑ | 0.417 | 0.381 | 0.452 | 130 | ↑ |
| Honduras | 112.141 | 13 | ↑ | 2.589 | 2.348 | 2.830 | 28 | ↑ |
| Hungary | -8.264 | 22 | ↓ | -0.878 | -1.431 | -0.321 | 12 | ↓ |
| Iceland | -8.286 | 21 | ↓ | -0.538 | -0.757 | -0.318 | 21 | ↓ |
| India | 27.210 | 99 | ↑ | 0.688 | 0.231 | 1.147 | 112 | ↑ |
| Indonesia | 5.669 | 153 | ↑ | 0.149 | 0.091 | 0.207 | 150 | ↑ |
| Iran | 112.221 | 12 | ↑ | 3.564 | 2.996 | 4.135 | 9 | ↑ |
| Iraq | 9.033 | 147 | ↑ | 0.213 | -0.002 | 0.428 | 145 | ↑ |
| Ireland | 59.170 | 57 | ↑ | 2.612 | 2.285 | 2.941 | 27 | ↑ |
| Israel | 89.347 | 25 | ↑ | 2.314 | 1.934 | 2.696 | 35 | ↑ |
| Italy | 216.792 | 1 | ↑ | 3.313 | 2.477 | 4.156 | 12 | ↑ |
| Jamaica | 181.015 | 4 | ↑ | 3.572 | 3.090 | 4.057 | 8 | ↑ |
| Japan | 95.983 | 23 | ↑ | 2.975 | 2.667 | 3.283 | 21 | ↑ |
| Jordan | 34.730 | 88 | ↑ | 0.506 | 0.172 | 0.841 | 127 | ↑ |
| Kazakhstan | 15.427 | 129 | ↑ | 0.050 | -0.379 | 0.482 | 153 | ↑ |
| Kenya | 11.916 | 139 | ↑ | 0.304 | 0.246 | 0.361 | 141 | ↑ |
| Kiribati | -0.717 | 31 | ↓ | 0.201 | 0.026 | 0.376 | 148 | ↑ |
| Kuwait | 27.991 | 98 | ↑ | 1.710 | 1.061 | 2.363 | 59 | ↑ |
| Kyrgyzstan | 36.675 | 82 | ↑ | 1.310 | 0.908 | 1.714 | 76 | ↑ |
| Laos | -12.799 | 12 | ↓ | -0.663 | -0.806 | -0.519 | 18 | ↓ |
| Latvia | 100.289 | 17 | ↑ | 3.087 | 2.736 | 3.439 | 16 | ↑ |
| Lebanon | 119.368 | 10 | ↑ | 3.133 | 2.986 | 3.281 | 14 | ↑ |
| Lesotho | 38.774 | 79 | ↑ | 1.681 | 1.227 | 2.138 | 61 | ↑ |
| Liberia | 20.500 | 116 | ↑ | 1.088 | 0.889 | 1.287 | 91 | ↑ |
| Libya | 89.233 | 27 | ↑ | 2.733 | 2.450 | 3.017 | 24 | ↑ |
| Lithuania | 81.875 | 37 | ↑ | 1.815 | 1.534 | 2.097 | 56 | ↑ |
| Luxembourg | 30.237 | 95 | ↑ | 0.803 | 0.550 | 1.056 | 106 | ↑ |
| Macedonia | 131.307 | 9 | ↑ | 3.575 | 3.153 | 3.998 | 7 | ↑ |
| Madagascar | -14.934 | 9 | ↓ | -0.771 | -0.868 | -0.674 | 15 | ↓ |
| Malawi | -8.736 | 20 | ↓ | -1.056 | -1.367 | -0.743 | 8 | ↓ |
| Malaysia | 57.969 | 59 | ↑ | 2.037 | 1.719 | 2.357 | 48 | ↑ |
| Maldives | -1.781 | 30 | ↓ | -0.485 | -0.718 | -0.252 | 24 | ↓ |
| Mali | -4.786 | 26 | ↓ | -0.116 | -0.245 | 0.014 | 36 | ↓ |
| Malta | 61.116 | 56 | ↑ | 1.658 | 1.512 | 1.804 | 64 | ↑ |
| Marshall Islands | 37.147 | 81 | ↑ | 1.168 | 1.078 | 1.258 | 87 | ↑ |
| Mauritania | 18.827 | 121 | ↑ | 0.763 | 0.664 | 0.863 | 108 | ↑ |
| Mauritius | -5.730 | 23 | ↓ | -0.991 | -1.242 | -0.739 | 9 | ↓ |
| Mexico | 85.314 | 33 | ↑ | 2.679 | 2.431 | 2.927 | 25 | ↑ |
| Moldova | 35.868 | 84 | ↑ | 0.708 | 0.360 | 1.058 | 111 | ↑ |
| Mongolia | 36.174 | 83 | ↑ | 0.876 | 0.428 | 1.327 | 103 | ↑ |
| Montenegro | 52.341 | 66 | ↑ | 1.572 | 1.417 | 1.726 | 68 | ↑ |
| Morocco | 49.223 | 69 | ↑ | 1.559 | 1.465 | 1.654 | 69 | ↑ |
| Mozambique | -2.966 | 29 | ↓ | -0.127 | -0.316 | 0.063 | 35 | ↓ |
| Myanmar | -5.447 | 25 | ↓ | -0.304 | -0.408 | -0.200 | 30 | ↓ |
| Namibia | -11.914 | 15 | ↓ | -1.151 | -1.728 | -0.570 | 5 | ↓ |
| Nepal | -10.811 | 17 | ↓ | -0.355 | -0.831 | 0.123 | 28 | ↓ |
| Netherlands | 56.708 | 61 | ↑ | 1.721 | 1.600 | 1.842 | 57 | ↑ |
| New Zealand | 3.221 | 158 | ↑ | 0.211 | 0.121 | 0.302 | 146 | ↑ |
| Nicaragua | 24.895 | 106 | ↑ | 1.577 | 1.006 | 2.152 | 66 | ↑ |
| Niger | 7.825 | 151 | ↑ | 0.354 | 0.280 | 0.429 | 137 | ↑ |
| Nigeria | 20.678 | 113 | ↑ | 0.803 | 0.704 | 0.901 | 107 | ↑ |
| North Korea | 0.397 | 162 | ↑ | -0.031 | -0.278 | 0.216 | 39 | ↓ |
| Northern Mariana Islands | 34.500 | 90 | ↑ | 1.273 | 0.840 | 1.708 | 80 | ↑ |
| Norway | 35.135 | 86 | ↑ | 1.146 | 0.839 | 1.454 | 89 | ↑ |
| Oman | 85.067 | 34 | ↑ | 2.225 | 2.068 | 2.383 | 37 | ↑ |
| Pakistan | 37.996 | 80 | ↑ | 1.002 | 0.908 | 1.096 | 96 | ↑ |
| Palestine | 15.670 | 128 | ↑ | 0.603 | 0.360 | 0.848 | 122 | ↑ |
| Panama | 100.361 | 16 | ↑ | 3.094 | 2.827 | 3.361 | 15 | ↑ |
| Papua New Guinea | 10.487 | 144 | ↑ | 0.460 | 0.410 | 0.510 | 129 | ↑ |
| Paraguay | 2.200 | 159 | ↑ | -0.846 | -1.139 | -0.552 | 13 | ↓ |
| Peru | 28.493 | 97 | ↑ | 0.275 | -0.173 | 0.725 | 142 | ↑ |
| Philippines | 4.368 | 156 | ↑ | -0.470 | -0.788 | -0.150 | 25 | ↓ |
| Poland | 98.931 | 20 | ↑ | 2.952 | 2.808 | 3.097 | 22 | ↑ |
| Portugal | 32.505 | 92 | ↑ | 0.884 | 0.742 | 1.026 | 102 | ↑ |
| Puerto Rico | 75.586 | 44 | ↑ | 2.181 | 1.972 | 2.391 | 42 | ↑ |
| Qatar | 178.605 | 5 | ↑ | 4.245 | 3.782 | 4.709 | 3 | ↑ |
| Romania | 64.291 | 53 | ↑ | 1.711 | 1.551 | 1.872 | 58 | ↑ |
| Russian Federation | 9.942 | 145 | ↑ | 0.225 | -0.074 | 0.525 | 144 | ↑ |
| Rwanda | -26.603 | 4 | ↓ | -1.532 | -1.758 | -1.305 | 3 | ↓ |
| Saint Lucia | 58.232 | 58 | ↑ | 1.384 | 1.209 | 1.558 | 74 | ↑ |
| Saint Vincent and the Grenadines | 54.347 | 63 | ↑ | 1.238 | 1.013 | 1.463 | 84 | ↑ |
| Samoa | 13.224 | 136 | ↑ | 0.417 | 0.320 | 0.513 | 131 | ↑ |
| Sao Tome and Principe | 51.557 | 67 | ↑ | 1.496 | 1.432 | 1.559 | 72 | ↑ |
| Saudi Arabia | 210.766 | 3 | ↑ | 5.301 | 4.927 | 5.677 | 2 | ↑ |
| Senegal | 25.033 | 105 | ↑ | 0.926 | 0.834 | 1.019 | 100 | ↑ |
| Serbia | 87.745 | 29 | ↑ | 3.147 | 2.840 | 3.453 | 13 | ↑ |
| Seychelles | 21.881 | 112 | ↑ | 0.376 | 0.203 | 0.550 | 134 | ↑ |
| Sierra Leone | 41.209 | 76 | ↑ | 1.650 | 1.523 | 1.777 | 65 | ↑ |
| Singapore | 100.793 | 15 | ↑ | 2.573 | 2.083 | 3.066 | 29 | ↑ |
| Slovakia | 42.201 | 74 | ↑ | 1.313 | 1.204 | 1.423 | 75 | ↑ |
| Slovenia | 20.587 | 115 | ↑ | 0.398 | 0.229 | 0.567 | 132 | ↑ |
| Solomon Islands | 2.006 | 160 | ↑ | 0.037 | -0.023 | 0.096 | 154 | ↑ |
| Somalia | -12.771 | 13 | ↓ | -0.793 | -0.961 | -0.624 | 14 | ↓ |
| South Africa | 41.561 | 75 | ↑ | 1.148 | 0.776 | 1.522 | 88 | ↑ |
| South Korea | -28.683 | 2 | ↓ | -0.677 | -1.502 | 0.155 | 17 | ↓ |
| South Sudan | -18.532 | 7 | ↓ | -1.085 | -1.243 | -0.926 | 7 | ↓ |
| Spain | 40.241 | 77 | ↑ | 1.504 | 1.361 | 1.648 | 71 | ↑ |
| Sri Lanka | 89.229 | 28 | ↑ | 3.073 | 2.866 | 3.280 | 18 | ↑ |
| Sudan | 23.503 | 110 | ↑ | 0.938 | 0.750 | 1.126 | 98 | ↑ |
| Suriname | 67.557 | 48 | ↑ | 1.680 | 1.435 | 1.926 | 63 | ↑ |
| Swaziland | 10.902 | 141 | ↑ | 0.366 | -0.063 | 0.797 | 136 | ↑ |
| Sweden | 5.808 | 152 | ↑ | -0.028 | -0.275 | 0.220 | 40 | ↓ |
| Switzerland | 4.514 | 154 | ↑ | 0.027 | -0.299 | 0.353 | 155 | ↑ |
| Syria | 52.717 | 65 | ↑ | 1.576 | 1.093 | 2.061 | 67 | ↑ |
| Taiwan | 214.720 | 2 | ↑ | 6.112 | 5.550 | 6.677 | 1 | ↑ |
| Tajikistan | 174.004 | 7 | ↑ | 4.150 | 3.654 | 4.649 | 5 | ↑ |
| Tanzania | -12.849 | 11 | ↓ | -0.929 | -1.185 | -0.673 | 11 | ↓ |
| Thailand | 35.444 | 85 | ↑ | 0.661 | 0.477 | 0.845 | 115 | ↑ |
| The Bahamas | 62.072 | 54 | ↑ | 1.680 | 1.331 | 2.031 | 62 | ↑ |
| The Gambia | 24.791 | 108 | ↑ | 0.967 | 0.894 | 1.041 | 97 | ↑ |
| Timor-Leste | 11.442 | 140 | ↑ | 0.632 | 0.376 | 0.889 | 118 | ↑ |
| Togo | 13.311 | 135 | ↑ | 0.492 | 0.438 | 0.546 | 128 | ↑ |
| Tonga | 24.801 | 107 | ↑ | 0.836 | 0.773 | 0.899 | 105 | ↑ |
| Trinidad and Tobago | 65.232 | 50 | ↑ | 1.865 | 1.601 | 2.129 | 54 | ↑ |
| Tunisia | 70.019 | 47 | ↑ | 1.554 | 1.359 | 1.750 | 70 | ↑ |
| Turkey | 66.986 | 49 | ↑ | 2.188 | 1.987 | 2.389 | 41 | ↑ |
| Turkmenistan | -19.423 | 6 | ↓ | -1.098 | -1.970 | -0.218 | 6 | ↓ |
| Uganda | -12.528 | 14 | ↓ | -0.977 | -1.187 | -0.767 | 10 | ↓ |
| Ukraine | 99.949 | 18 | ↑ | 2.661 | 2.218 | 3.106 | 26 | ↑ |
| United Arab Emirates | 93.859 | 24 | ↑ | 2.054 | 1.637 | 2.474 | 47 | ↑ |
| United Kingdom | 82.937 | 35 | ↑ | 3.028 | 2.721 | 3.336 | 20 | ↑ |
| United States | 46.861 | 70 | ↑ | 1.287 | 1.190 | 1.385 | 79 | ↑ |
| Uruguay | 13.201 | 137 | ↑ | 0.304 | 0.167 | 0.441 | 139 | ↑ |
| Uzbekistan | 114.462 | 11 | ↑ | 3.922 | 3.359 | 4.488 | 6 | ↑ |
| Vanuatu | 15.878 | 127 | ↑ | 0.673 | 0.628 | 0.718 | 113 | ↑ |
| Venezuela | 13.333 | 134 | ↑ | 0.576 | 0.203 | 0.950 | 125 | ↑ |
| Vietnam | 54.131 | 64 | ↑ | 1.833 | 1.692 | 1.973 | 55 | ↑ |
| Virgin Islands, US | 76.063 | 43 | ↑ | 2.195 | 1.769 | 2.622 | 40 | ↑ |
| Yemen | 34.643 | 89 | ↑ | 1.264 | 1.181 | 1.347 | 82 | ↑ |
| Zambia | -32.269 | 1 | ↓ | -2.029 | -2.519 | -1.537 | 1 | ↓ |
| Zimbabwe | 28.871 | 96 | ↑ | 1.270 | 0.572 | 1.974 | 81 | ↑ |

a: years lived with disability b: percent change.

c: annual percent change d: confidence interval

5

| **Characteristics** | **PC^b^(%)** | | | **APC^c^ (%)** | | | | |
| --- | --- | --- | --- | --- | --- | --- | --- | --- |
|  | **Value** | **Rank** | | **Value** | **95%CI^d^** | **95%CI** | **Rank** | |
| **Global** | -28.535 |  |  | -1.365 | -1.427 | -1.304 |  |  |
| **Sociodemographic index** |  |  |  |  |  |  |  |  |
| Low | -26.478 | 3 | ↓ | -1.356 | -1.552 | -1.161 | 3 | ↓ |
| Low-middle | -13.788 | 4 | ↓ | -0.811 | -0.923 | -0.698 | 4 | ↓ |
| Middle | -37.505 | 2 | ↓ | -1.845 | -1.957 | -1.733 | 2 | ↓ |
| High-Middle | -41.044 | 1 | ↓ | -2.175 | -2.334 | -2.015 | 1 | ↓ |
| High | -11.275 | 5 | ↓ | -0.276 | -0.401 | -0.150 | 5 | ↓ |
| **Region** |  |  |  |  |  |  |  |  |
| Central Asia | 1.858 | 5 | ↑ | 0.048 | -0.088 | 0.185 | 4 | ↑ |
| East Asia | -55.085 | 1 | ↓ | -2.912 | -3.140 | -2.684 | 1 | ↓ |
| High-income Asia Pacific | -29.718 | 5 | ↓ | -0.849 | -1.122 | -0.576 | 10 | ↓ |
| South Asia | -13.113 | 14 | ↓ | -0.794 | -1.008 | -0.579 | 11 | ↓ |
| Southeast Asia | -24.299 | 7 | ↓ | -1.188 | -1.332 | -1.044 | 5 | ↓ |
| Central Europe | -18.995 | 10 | ↓ | -0.712 | -0.885 | -0.539 | 14 | ↓ |
| Eastern Europe | -14.905 | 13 | ↓ | -1.054 | -1.428 | -0.679 | 8 | ↓ |
| Western Europe | -9.703 | 15 | ↓ | -0.220 | -0.323 | -0.116 | 15 | ↓ |
| Andean Latin America | -37.439 | 2 | ↓ | -1.882 | -2.000 | -1.764 | 2 | ↓ |
| Central Latin America | -24.462 | 6 | ↓ | -1.081 | -1.347 | -0.815 | 7 | ↓ |
| Southern Latin America | -31.265 | 4 | ↓ | -1.559 | -1.790 | -1.329 | 4 | ↓ |
| Tropical Latin America | -20.473 | 9 | ↓ | -1.113 | -1.234 | -0.991 | 6 | ↓ |
| High income North America | 6.479 | 2 | ↑ | 0.353 | 0.240 | 0.466 | 3 | ↑ |
| Central Sub-Saharan Africa | -16.527 | 12 | ↓ | -0.748 | -0.803 | -0.694 | 13 | ↓ |
| Eastern Sub-Saharan Africa | -32.645 | 3 | ↓ | -1.832 | -2.007 | -1.656 | 3 | ↓ |
| Southern Sub-Saharan Africa | 12.789 | 1 | ↑ | 0.559 | -0.016 | 1.138 | 1 | ↑ |
| Western Sub-Saharan Africa | -3.150 | 16 | ↓ | -0.130 | -0.167 | -0.092 | 16 | ↓ |
| North Africa and Middle East | -21.269 | 8 | ↓ | -0.922 | -1.039 | -0.805 | 9 | ↓ |
| Oceania | 4.494 | 3 | ↑ | 0.382 | 0.280 | 0.484 | 2 | ↑ |
| Australasia | -17.358 | 11 | ↓ | -0.785 | -0.870 | -0.700 | 12 | ↓ |
| Caribbean | 2.925 | 4 | ↑ | -0.111 | -0.283 | 0.062 | 17 | ↓ |

a: years of life lost. b: percent change.

c: annual percent change d: confidence interval

# Supplemental Table 26. The relative contributions of each geographical locations in trends of endometrial cancer YLLs^a^ from 1990-2017

|  | **Increasing trend** | **Decreasing trend** |
| --- | --- | --- |
| **Characteristics** | **Contribution rate (%)** | **Contribution rate (%)** |
| **Sociodemographic index** |  |  |
| Low | - | 20.99 |
| Low-middle | - | 12.55 |
| Middle | - | 28.55 |
| Middle-High | - | 33.65 |
| High | - | 4.27 |
| **Region** |  |  |
| Central Asia | 3.60 | - |
| Eastern Asia | - | 16.28 |
| High-income Asia Pacific | - | 4.75 |
| South Asia | - | 4.44 |
| Southeast Asia | - | 6.64 |
| Central Europe | - | 3.98 |
| Eastern Europe | - | 5.89 |
| Western Europe | - | 1.23 |
| Andean Latin America | - | 10.52 |
| Central Latin America | - | 6.04 |
| Southern Latin America | - | 8.72 |
| Tropical Latin America | - | 6.22 |
| North America | 26.29 | - |
| Central Sub-Saharan Africa | - | 4.18 |
| Eastern Sub-Saharan Africa | - | 10.24 |
| Southern Sub-Saharan Africa | 41.67 | - |
| Western Sub-Saharan Africa | - | 0.72 |
| North Africa and Middle East | - | 5.15 |
| Oceania | 28.45 | - |
| Australasia | - | 4.39 |
| Caribbean | - | 0.62 |

a: years of life lost.

# Supplemental Table 27. Trends in endometrial cancer age-standardized YLLs^a^ rate of 195 countries and territories from 1990-2017

| **Countries and territories** | **PC^b^** | | | **APC^c^** | | | | |
| --- | --- | --- | --- | --- | --- | --- | --- | --- |
|  | **Value** | **Rank** | | **Value** | **95%CI^d^** | **95%CI** | **Rank** | |
| Afghanistan | -4.036 | 119 | ↓ | -0.038 | -0.105 | 0.029 | 126 | ↓ |
| Albania | -17.172 | 83 | ↓ | -0.524 | -0.707 | -0.340 | 92 | ↓ |
| Algeria | -14.118 | 95 | ↓ | -0.190 | -0.338 | -0.041 | 110 | ↓ |
| American Samoa | 44.345 | 7 | ↑ | 2.054 | 1.671 | 2.439 | 5 | ↑ |
| Andorra | -1.308 | 130 | ↓ | 0.057 | -0.268 | 0.382 | 66 | ↑ |
| Angola | -26.326 | 55 | ↓ | -1.270 | -1.372 | -1.168 | 58 | ↓ |
| Antigua and Barbuda | 30.535 | 13 | ↑ | 0.642 | 0.420 | 0.864 | 32 | ↑ |
| Argentina | -34.844 | 27 | ↓ | -1.869 | -2.108 | -1.629 | 28 | ↓ |
| Armenia | 25.644 | 16 | ↑ | 1.175 | 0.872 | 1.478 | 16 | ↑ |
| Australia | -15.915 | 88 | ↓ | -0.795 | -0.901 | -0.689 | 80 | ↓ |
| Austria | -36.855 | 22 | ↓ | -1.707 | -1.839 | -1.574 | 38 | ↓ |
| Azerbaijan | 4.988 | 51 | ↑ | -0.122 | -0.339 | 0.094 | 116 | ↓ |
| Bahrain | -21.298 | 71 | ↓ | -1.518 | -1.785 | -1.250 | 44 | ↓ |
| Bangladesh | -51.627 | 6 | ↓ | -2.468 | -2.645 | -2.292 | 13 | ↓ |
| Barbados | 15.989 | 34 | ↑ | 0.554 | 0.337 | 0.771 | 39 | ↑ |
| Belarus | -24.824 | 62 | ↓ | -0.866 | -1.671 | -0.055 | 75 | ↓ |
| Belgium | -18.797 | 77 | ↓ | -0.811 | -0.961 | -0.660 | 79 | ↓ |
| Belize | 3.451 | 56 | ↑ | -0.081 | -0.409 | 0.248 | 122 | ↓ |
| Benin | 8.141 | 47 | ↑ | 0.469 | 0.408 | 0.531 | 42 | ↑ |
| Bermuda | -42.897 | 11 | ↓ | -2.551 | -2.938 | -2.162 | 12 | ↓ |
| Bhutan | -46.281 | 9 | ↓ | -2.560 | -2.724 | -2.397 | 11 | ↓ |
| Bolivia | -27.475 | 53 | ↓ | -1.293 | -1.352 | -1.234 | 57 | ↓ |
| Bosnia and Herzegovina | 33.103 | 11 | ↑ | 1.298 | 0.938 | 1.660 | 14 | ↑ |
| Botswana | 6.669 | 49 | ↑ | 1.216 | 0.705 | 1.729 | 15 | ↑ |
| Brazil | -19.907 | 74 | ↓ | -1.064 | -1.184 | -0.944 | 69 | ↓ |
| Brunei | -18.481 | 81 | ↓ | -0.200 | -0.672 | 0.275 | 109 | ↓ |
| Bulgaria | 1.757 | 59 | ↑ | 0.772 | 0.376 | 1.169 | 27 | ↑ |
| Burkina Faso | -6.165 | 115 | ↓ | -0.127 | -0.326 | 0.072 | 115 | ↓ |
| Burundi | -41.641 | 15 | ↓ | -2.435 | -2.654 | -2.216 | 16 | ↓ |
| Cambodia | -30.451 | 38 | ↓ | -1.463 | -1.528 | -1.397 | 47 | ↓ |
| Cameroon | -6.719 | 112 | ↓ | -0.227 | -0.380 | -0.075 | 108 | ↓ |
| Canada | -18.281 | 82 | ↓ | -0.507 | -0.782 | -0.230 | 93 | ↓ |
| Cape Verde | 15.554 | 36 | ↑ | 0.389 | 0.252 | 0.525 | 46 | ↑ |
| Central African Republic | -14.449 | 93 | ↓ | -0.614 | -0.701 | -0.528 | 87 | ↓ |
| Chad | 15.725 | 35 | ↑ | 0.762 | 0.670 | 0.853 | 29 | ↑ |
| Chile | -2.762 | 123 | ↓ | 0.188 | -0.050 | 0.428 | 55 | ↑ |
| China | -56.658 | 3 | ↓ | -3.039 | -3.280 | -2.797 | 5 | ↓ |
| Colombia | -46.927 | 8 | ↓ | -2.671 | -3.042 | -2.298 | 9 | ↓ |
| Comoros | -33.116 | 33 | ↓ | -1.722 | -1.870 | -1.573 | 35 | ↓ |
| Congo | -13.627 | 98 | ↓ | -0.732 | -0.872 | -0.591 | 84 | ↓ |
| Costa Rica | 9.419 | 43 | ↑ | 0.204 | -0.006 | 0.414 | 53 | ↑ |
| Cote d'Ivoire | -1.675 | 128 | ↓ | -0.109 | -0.251 | 0.032 | 118 | ↓ |
| Croatia | -8.464 | 108 | ↓ | 0.102 | -0.212 | 0.417 | 61 | ↑ |
| Cuba | 18.636 | 27 | ↑ | 0.256 | -0.064 | 0.576 | 50 | ↑ |
| Cyprus | -28.218 | 46 | ↓ | -1.195 | -1.356 | -1.035 | 62 | ↓ |
| Czech Republic | -42.401 | 12 | ↓ | -2.062 | -2.138 | -1.986 | 21 | ↓ |
| Democratic Republic of the Congo | -13.557 | 99 | ↓ | -0.604 | -0.717 | -0.492 | 88 | ↓ |
| Denmark | -33.856 | 31 | ↓ | -1.970 | -2.244 | -1.695 | 24 | ↓ |
| Djibouti | -27.775 | 48 | ↓ | -1.540 | -1.667 | -1.412 | 41 | ↓ |
| Dominica | 32.749 | 12 | ↑ | 0.953 | 0.845 | 1.060 | 20 | ↑ |
| Dominican Republic | -42.230 | 13 | ↓ | -2.639 | -2.975 | -2.301 | 10 | ↓ |
| Ecuador | -41.321 | 16 | ↓ | -1.527 | -2.022 | -1.029 | 43 | ↓ |
| Egypt | -9.089 | 105 | ↓ | -0.248 | -0.362 | -0.135 | 105 | ↓ |
| El Salvador | -25.286 | 56 | ↓ | -1.898 | -2.169 | -1.626 | 27 | ↓ |
| Equatorial Guinea | -34.226 | 30 | ↓ | -1.536 | -1.679 | -1.393 | 42 | ↓ |
| Eritrea | -22.616 | 68 | ↓ | -1.119 | -1.176 | -1.062 | 65 | ↓ |
| Estonia | -15.113 | 90 | ↓ | -0.975 | -1.306 | -0.644 | 72 | ↓ |
| Ethiopia | -43.378 | 10 | ↓ | -2.453 | -2.589 | -2.317 | 15 | ↓ |
| Federated States of Micronesia | -5.270 | 116 | ↓ | -0.165 | -0.195 | -0.136 | 112 | ↓ |
| Fiji | 10.742 | 40 | ↑ | 0.763 | 0.494 | 1.033 | 28 | ↑ |
| Finland | -18.768 | 78 | ↓ | -0.768 | -0.921 | -0.615 | 81 | ↓ |
| France | -22.460 | 69 | ↓ | -0.755 | -0.976 | -0.533 | 82 | ↓ |
| Gabon | -20.286 | 73 | ↓ | -0.873 | -1.201 | -0.543 | 74 | ↓ |
| Georgia | 93.173 | 3 | ↑ | 2.923 | 1.829 | 4.028 | 1 | ↑ |
| Germany | -31.244 | 37 | ↓ | -1.487 | -1.878 | -1.094 | 46 | ↓ |
| Ghana | -4.472 | 118 | ↓ | -0.229 | -0.288 | -0.169 | 107 | ↓ |
| Greece | 30.314 | 14 | ↑ | 1.460 | 1.199 | 1.722 | 9 | ↑ |
| Greenland | -25.029 | 58 | ↓ | -1.215 | -1.461 | -0.969 | 60 | ↓ |
| Grenada | 21.459 | 22 | ↑ | 1.108 | 0.815 | 1.402 | 17 | ↑ |
| Guam | 4.635 | 52 | ↑ | 0.588 | 0.067 | 1.112 | 36 | ↑ |
| Guatemala | -29.841 | 42 | ↓ | -2.823 | -3.351 | -2.291 | 7 | ↓ |
| Guinea | 1.557 | 60 | ↑ | 0.126 | 0.044 | 0.207 | 58 | ↑ |
| Guinea-Bissau | 3.749 | 54 | ↑ | 0.354 | 0.255 | 0.452 | 47 | ↑ |
| Guyana | 29.008 | 15 | ↑ | 0.689 | 0.254 | 1.127 | 30 | ↑ |
| Haiti | -11.139 | 103 | ↓ | -0.396 | -0.427 | -0.365 | 101 | ↓ |
| Honduras | 22.173 | 21 | ↑ | 0.612 | 0.420 | 0.805 | 35 | ↑ |
| Hungary | -52.699 | 5 | ↓ | -3.418 | -4.034 | -2.798 | 3 | ↓ |
| Iceland | -38.242 | 20 | ↓ | -1.963 | -2.062 | -1.864 | 25 | ↓ |
| India | -14.159 | 94 | ↓ | -0.903 | -1.230 | -0.574 | 73 | ↓ |
| Indonesia | -20.360 | 72 | ↓ | -0.850 | -0.979 | -0.721 | 78 | ↓ |
| Iran | 16.219 | 33 | ↑ | 1.309 | 0.622 | 2.001 | 13 | ↑ |
| Iraq | -15.929 | 87 | ↓ | -0.861 | -1.087 | -0.635 | 77 | ↓ |
| Ireland | -19.618 | 75 | ↓ | -0.082 | -0.405 | 0.241 | 121 | ↓ |
| Israel | 17.218 | 29 | ↑ | 0.639 | 0.362 | 0.917 | 33 | ↑ |
| Italy | 74.964 | 4 | ↑ | 1.371 | 0.653 | 2.095 | 11 | ↑ |
| Jamaica | 101.538 | 1 | ↑ | 2.493 | 2.049 | 2.940 | 4 | ↑ |
| Japan | -2.712 | 125 | ↓ | 0.281 | 0.126 | 0.436 | 49 | ↑ |
| Jordan | -29.155 | 43 | ↓ | -1.800 | -2.234 | -1.364 | 32 | ↓ |
| Kazakhstan | -31.293 | 36 | ↓ | -2.180 | -2.506 | -1.853 | 19 | ↓ |
| Kenya | -10.076 | 104 | ↓ | -0.498 | -0.572 | -0.424 | 94 | ↓ |
| Kiribati | -3.879 | 120 | ↓ | 0.084 | -0.064 | 0.232 | 64 | ↑ |
| Kuwait | -23.557 | 66 | ↓ | -0.157 | -0.892 | 0.585 | 113 | ↓ |
| Kyrgyzstan | -2.235 | 126 | ↓ | 0.088 | -0.341 | 0.519 | 62 | ↑ |
| Laos | -34.562 | 28 | ↓ | -1.747 | -1.841 | -1.652 | 34 | ↓ |
| Latvia | 24.019 | 17 | ↑ | 0.836 | 0.497 | 1.177 | 24 | ↑ |
| Lebanon | -27.542 | 51 | ↓ | -1.380 | -1.606 | -1.153 | 50 | ↓ |
| Lesotho | 33.294 | 10 | ↑ | 1.665 | 1.113 | 2.220 | 8 | ↑ |
| Liberia | 5.375 | 50 | ↑ | 0.482 | 0.295 | 0.670 | 40 | ↑ |
| Libya | 2.263 | 58 | ↑ | 0.060 | -0.066 | 0.186 | 65 | ↑ |
| Lithuania | 22.402 | 20 | ↑ | -0.025 | -0.397 | 0.348 | 128 | ↓ |
| Luxembourg | -24.935 | 59 | ↓ | -1.092 | -1.239 | -0.945 | 66 | ↓ |
| Macedonia | 20.688 | 23 | ↑ | 0.953 | 0.655 | 1.253 | 19 | ↑ |
| Madagascar | -27.952 | 47 | ↓ | -1.408 | -1.542 | -1.274 | 49 | ↓ |
| Malawi | -24.870 | 61 | ↓ | -1.860 | -2.218 | -1.500 | 29 | ↓ |
| Malaysia | -16.037 | 85 | ↓ | -0.462 | -0.712 | -0.212 | 97 | ↓ |
| Maldives | -60.540 | 2 | ↓ | -4.003 | -4.232 | -3.773 | 2 | ↓ |
| Mali | -27.552 | 50 | ↓ | -1.195 | -1.339 | -1.050 | 63 | ↓ |
| Malta | -8.229 | 109 | ↓ | -0.491 | -0.588 | -0.394 | 96 | ↓ |
| Marshall Islands | 18.548 | 28 | ↑ | 0.582 | 0.339 | 0.826 | 37 | ↑ |
| Mauritania | -13.678 | 96 | ↓ | -0.404 | -0.485 | -0.323 | 100 | ↓ |
| Mauritius | -34.457 | 29 | ↓ | -2.053 | -2.241 | -1.865 | 23 | ↓ |
| Mexico | -13.085 | 101 | ↓ | -0.053 | -0.423 | 0.319 | 123 | ↓ |
| Moldova | -2.189 | 127 | ↓ | -0.625 | -1.069 | -0.179 | 86 | ↓ |
| Mongolia | -16.055 | 84 | ↓ | -1.312 | -1.808 | -0.814 | 56 | ↓ |
| Montenegro | -9.026 | 106 | ↓ | -0.566 | -0.813 | -0.318 | 89 | ↓ |
| Morocco | -14.571 | 91 | ↓ | -0.562 | -0.596 | -0.527 | 90 | ↓ |
| Mozambique | -23.666 | 65 | ↓ | -1.087 | -1.334 | -0.840 | 67 | ↓ |
| Myanmar | -35.290 | 25 | ↓ | -1.721 | -1.920 | -1.522 | 36 | ↓ |
| Namibia | -38.029 | 21 | ↓ | -2.393 | -3.095 | -1.685 | 17 | ↓ |
| Nepal | -35.662 | 24 | ↓ | -1.707 | -2.109 | -1.303 | 37 | ↓ |
| Netherlands | 0.510 | 63 | ↑ | -0.028 | -0.155 | 0.100 | 127 | ↓ |
| New Zealand | -21.891 | 70 | ↓ | -0.734 | -0.807 | -0.662 | 83 | ↓ |
| Nicaragua | -35.263 | 26 | ↓ | -1.315 | -1.854 | -0.773 | 54 | ↓ |
| Niger | -7.454 | 110 | ↓ | -0.281 | -0.369 | -0.194 | 104 | ↓ |
| Nigeria | -8.599 | 107 | ↓ | -0.430 | -0.502 | -0.359 | 99 | ↓ |
| North Korea | 4.311 | 53 | ↑ | 0.168 | 0.021 | 0.316 | 56 | ↑ |
| Northern Mariana Islands | 3.742 | 55 | ↑ | 0.774 | 0.465 | 1.085 | 26 | ↑ |
| Norway | -28.438 | 45 | ↓ | -1.235 | -1.391 | -1.080 | 59 | ↓ |
| Oman | -18.685 | 80 | ↓ | -0.668 | -0.810 | -0.525 | 85 | ↓ |
| Pakistan | 12.097 | 39 | ↑ | 0.103 | -0.084 | 0.291 | 60 | ↑ |
| Palestine | -11.550 | 102 | ↓ | -0.129 | -0.337 | 0.079 | 114 | ↓ |
| Panama | 8.706 | 45 | ↑ | 0.927 | 0.649 | 1.207 | 21 | ↑ |
| Papua New Guinea | 1.295 | 61 | ↑ | 0.238 | 0.128 | 0.349 | 52 | ↑ |
| Paraguay | -32.555 | 34 | ↓ | -2.216 | -2.475 | -1.956 | 18 | ↓ |
| Peru | -40.153 | 17 | ↓ | -2.460 | -2.828 | -2.090 | 14 | ↓ |
| Philippines | -18.739 | 79 | ↓ | -1.378 | -1.784 | -0.969 | 51 | ↓ |
| Poland | -6.892 | 111 | ↓ | -0.041 | -0.202 | 0.120 | 125 | ↓ |
| Portugal | -33.710 | 32 | ↓ | -1.569 | -1.704 | -1.434 | 40 | ↓ |
| Puerto Rico | -6.246 | 114 | ↓ | -0.235 | -0.368 | -0.102 | 106 | ↓ |
| Qatar | 20.683 | 24 | ↑ | 0.777 | 0.367 | 1.189 | 25 | ↑ |
| Romania | -24.606 | 63 | ↓ | -1.366 | -1.606 | -1.126 | 53 | ↓ |
| Russian Federation | -27.501 | 52 | ↓ | -1.626 | -2.063 | -1.186 | 39 | ↓ |
| Rwanda | -47.289 | 7 | ↓ | -2.958 | -3.248 | -2.668 | 6 | ↓ |
| Saint Lucia | -1.158 | 131 | ↓ | -0.305 | -0.495 | -0.115 | 103 | ↓ |
| Saint Vincent and the Grenadines | 19.860 | 25 | ↑ | 0.201 | -0.085 | 0.487 | 54 | ↑ |
| Samoa | -2.716 | 124 | ↓ | -0.099 | -0.227 | 0.029 | 120 | ↓ |
| Sao Tome and Principe | 19.170 | 26 | ↑ | 0.565 | 0.493 | 0.636 | 38 | ↑ |
| Saudi Arabia | 22.821 | 18 | ↑ | 1.391 | 1.066 | 1.718 | 10 | ↑ |
| Senegal | 9.792 | 41 | ↑ | 0.478 | 0.404 | 0.551 | 41 | ↑ |
| Serbia | 12.828 | 38 | ↑ | 0.898 | 0.694 | 1.102 | 22 | ↑ |
| Seychelles | -27.600 | 49 | ↓ | -1.313 | -1.481 | -1.143 | 55 | ↓ |
| Sierra Leone | 22.405 | 19 | ↑ | 1.091 | 0.961 | 1.220 | 18 | ↑ |
| Singapore | -15.301 | 89 | ↓ | -0.550 | -0.815 | -0.284 | 91 | ↓ |
| Slovakia | -30.056 | 40 | ↓ | -1.370 | -1.446 | -1.294 | 52 | ↓ |
| Slovenia | -35.714 | 23 | ↓ | -2.060 | -2.265 | -1.855 | 22 | ↓ |
| Solomon Islands | -0.986 | 132 | ↓ | 0.088 | -0.023 | 0.199 | 63 | ↑ |
| Somalia | -19.558 | 76 | ↓ | -1.142 | -1.303 | -0.981 | 64 | ↓ |
| South Africa | 8.407 | 46 | ↑ | 0.251 | -0.288 | 0.793 | 51 | ↑ |
| South Korea | -76.713 | 1 | ↓ | -5.402 | -6.301 | -4.494 | 1 | ↓ |
| South Sudan | -26.633 | 54 | ↓ | -1.515 | -1.720 | -1.309 | 45 | ↓ |
| Spain | -13.652 | 97 | ↓ | -0.371 | -0.476 | -0.266 | 102 | ↓ |
| Sri Lanka | 0.650 | 62 | ↑ | 0.633 | 0.301 | 0.967 | 34 | ↑ |
| Sudan | -15.989 | 86 | ↓ | -0.497 | -0.619 | -0.375 | 95 | ↓ |
| Suriname | 16.817 | 31 | ↑ | 0.304 | -0.033 | 0.642 | 48 | ↑ |
| Swaziland | -6.709 | 113 | ↓ | 0.001 | -0.601 | 0.606 | 67 | ↑ |
| Sweden | -24.877 | 60 | ↓ | -0.999 | -1.103 | -0.896 | 71 | ↓ |
| Switzerland | -25.035 | 57 | ↓ | -1.071 | -1.295 | -0.847 | 68 | ↓ |
| Syria | -24.557 | 64 | ↓ | -1.201 | -1.683 | -0.718 | 61 | ↓ |
| Taiwan | 48.380 | 5 | ↑ | 2.762 | 2.339 | 3.186 | 2 | ↑ |
| Tajikistan | 98.101 | 2 | ↑ | 2.750 | 2.357 | 3.145 | 3 | ↑ |
| Tanzania | -30.206 | 39 | ↓ | -1.813 | -2.096 | -1.528 | 31 | ↓ |
| Thailand | -31.943 | 35 | ↓ | -1.939 | -2.132 | -1.746 | 26 | ↓ |
| The Bahamas | 12.864 | 37 | ↑ | 0.443 | 0.262 | 0.624 | 44 | ↑ |
| The Gambia | 8.071 | 48 | ↑ | 0.417 | 0.357 | 0.476 | 45 | ↑ |
| Timor-Leste | -22.976 | 67 | ↓ | -1.030 | -1.132 | -0.928 | 70 | ↓ |
| Togo | -2.928 | 121 | ↓ | -0.104 | -0.132 | -0.076 | 119 | ↓ |
| Tonga | 2.904 | 57 | ↑ | 0.124 | 0.031 | 0.217 | 59 | ↑ |
| Trinidad and Tobago | 8.760 | 44 | ↑ | 0.166 | -0.045 | 0.377 | 57 | ↑ |
| Tunisia | -14.539 | 92 | ↓ | -0.863 | -0.985 | -0.740 | 76 | ↓ |
| Turkey | -39.902 | 18 | ↓ | -2.112 | -2.282 | -1.941 | 20 | ↓ |
| Turkmenistan | -53.738 | 4 | ↓ | -3.309 | -4.071 | -2.540 | 4 | ↓ |
| Uganda | -28.459 | 44 | ↓ | -1.773 | -1.992 | -1.553 | 33 | ↓ |
| Ukraine | 41.521 | 8 | ↑ | 0.880 | 0.492 | 1.269 | 23 | ↑ |
| United Arab Emirates | -1.668 | 129 | ↓ | -0.116 | -0.309 | 0.078 | 117 | ↓ |
| United Kingdom | 17.102 | 30 | ↑ | 1.345 | 1.010 | 1.681 | 12 | ↑ |
| United States | 9.450 | 42 | ↑ | 0.443 | 0.341 | 0.545 | 43 | ↑ |
| Uruguay | -29.889 | 41 | ↓ | -1.431 | -1.586 | -1.277 | 48 | ↓ |
| Uzbekistan | 34.607 | 9 | ↑ | 2.016 | 1.514 | 2.521 | 6 | ↑ |
| Vanuatu | 16.700 | 32 | ↑ | 0.652 | 0.536 | 0.768 | 31 | ↑ |
| Venezuela | -39.035 | 19 | ↓ | -1.838 | -2.257 | -1.418 | 30 | ↓ |
| Vietnam | -13.405 | 100 | ↓ | -0.459 | -0.510 | -0.408 | 98 | ↓ |
| Virgin Islands, US | -2.922 | 122 | ↓ | -0.051 | -0.152 | 0.049 | 124 | ↓ |
| Yemen | -4.681 | 117 | ↓ | -0.174 | -0.210 | -0.138 | 111 | ↓ |
| Zambia | -42.071 | 14 | ↓ | -2.747 | -3.202 | -2.290 | 8 | ↓ |
| Zimbabwe | 45.222 | 6 | ↑ | 1.928 | 0.921 | 2.946 | 7 | ↑ |

a: years of life lost. b: percent change.

c: annual percent change d: confidence interval

# Supplemental Figure 6. Trend of global incidence of endometrial cancer by age group, 1990−2017





#

Supplemental Figure 7. Trend of global prevalence of endometrial cancer by age group, 1990−2017

Supplemental Figure 8. Trends in the global disease burden of endometrial cancer mortality from 1990−2017. (a. Trends in the global disease burden of endometrial cancer mortality by sociodemographic index from 1990−2017; b. Trends in the global disease burden of endometrial cancer mortality by region from 1990−2017).





Supplemental Figure 9. The global disease burden of endometrial cancer mortality in 195 countries and territories. (a. The percent change in the age-standardized mortality rate of endometrial cancer between 1990 and 2017; b. The estimated annual percentage change in the endometrial cancer age-standardized mortality rate from 1990 to 2017.).


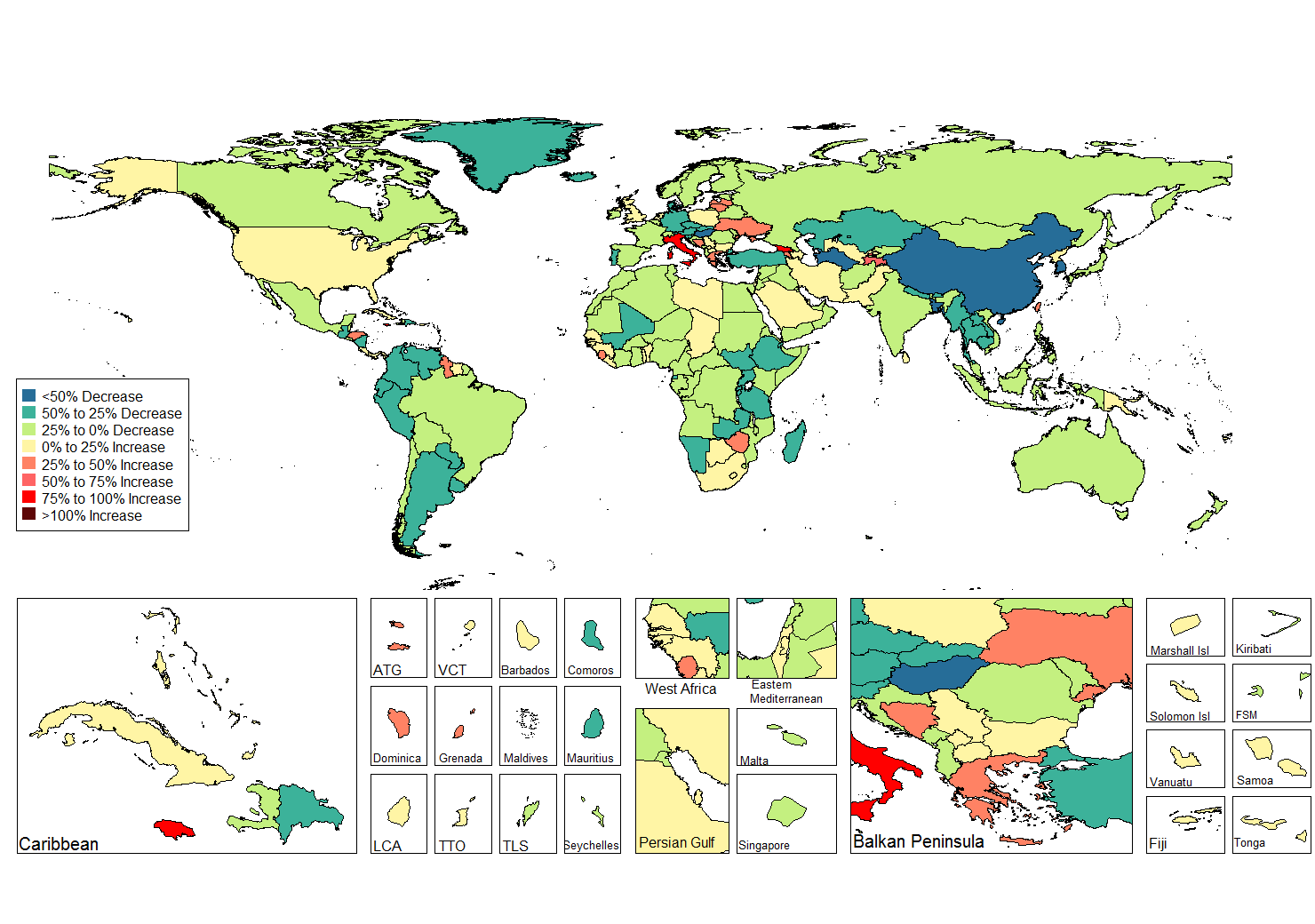

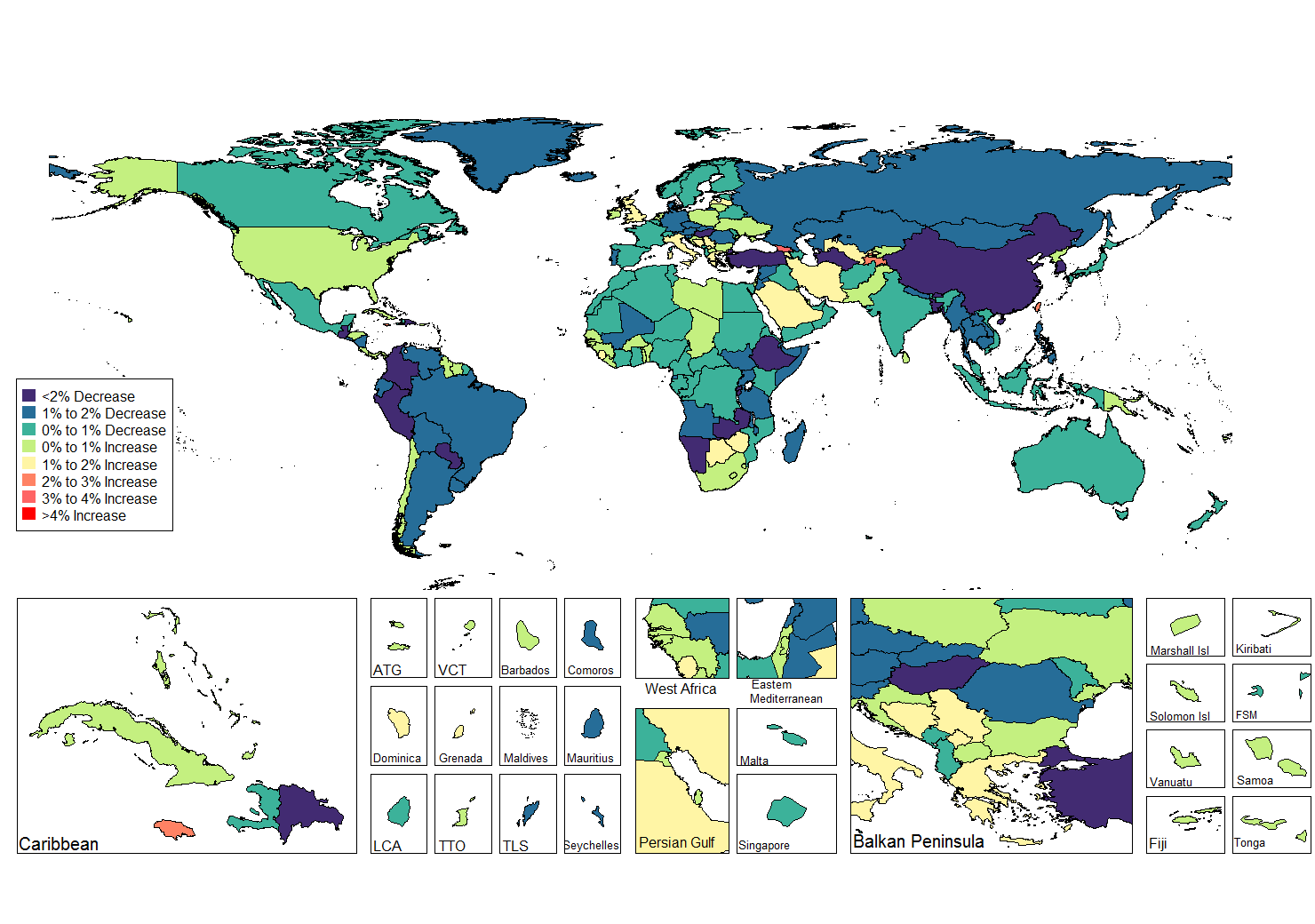


#

Supplemental Figure 10. Trend of global mortality of endometrial cancer by age group, 1990−2017

Supplemental Figure 11. Trends in global disease burden of endometrial cancer DALYs^a^ from 1990−2017. (a. Trends in the global disease burden of endometrial cancer DALYs by the sociodemographic index from 1990−2017; b. Trends in the global disease burden of endometrial cancer DALYs by region from 1990 to 2017).



a: disability adjusted life years

Supplemental Figure 12. The global disease burden of endometrial cancer DALYs^a^ in 195 countries and territories. (a. The percent change in the age-standardized DALYs rate of endometrial cancer between 1990 and 2017; b. The estimated annual percentage change of endometrial cancer age-standardized DALYs rate from 1990 to 2017.).


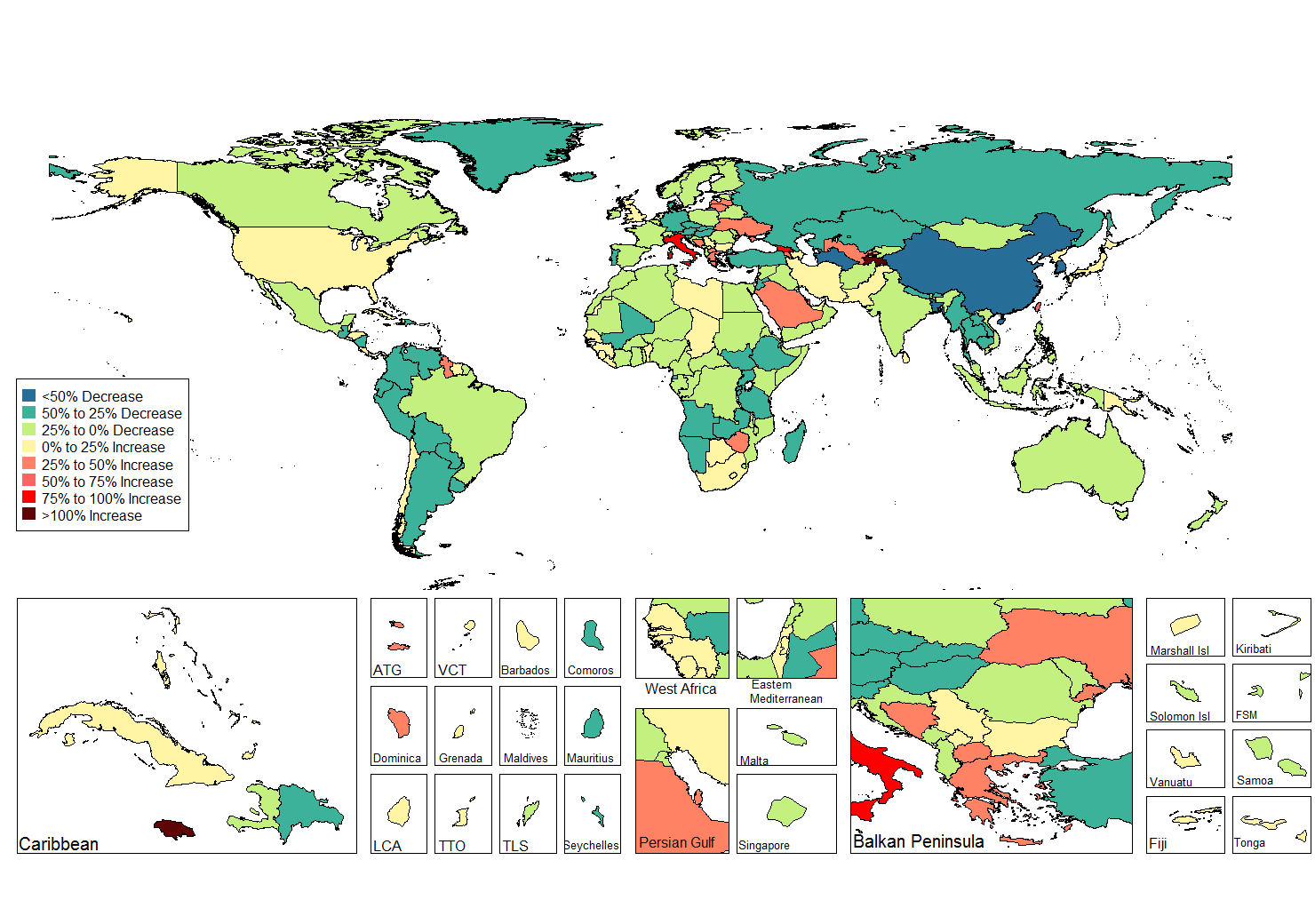


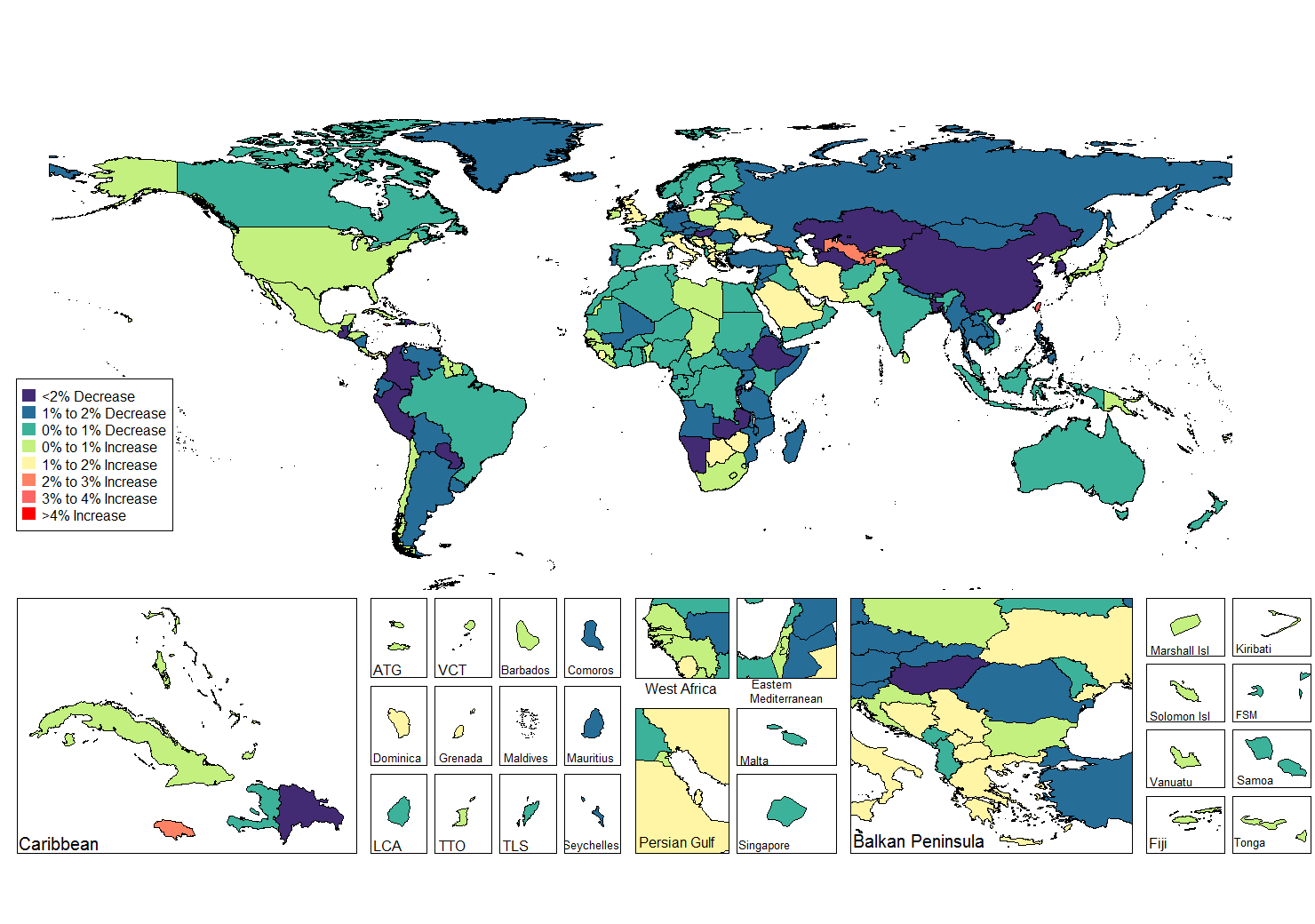


a: disability adjusted life years

Supplemental Figure 13. Trend of global DALYs^a^ of endometrial cancer by age group,1990−2017



a: disability adjusted life years

Supplemental Figure 14. Trends in the global disease burden of endometrial cancer YLDs^a^ from 1990−2017. (a. Trends in the global disease burden of endometrial cancer YLDs by sociodemographic index from 1990 to 2017; b. Trends in the global disease burden of endometrial cancer YLDs by region from 1990 to 2017).





a: years lived with disability

Supplemental Figure 15. The global disease burden of endometrial cancer YLDs^a^ in 195 countries and territories. (a. The percent change in the age-standardized YLDs rate of endometrial cancer between 1990 and 2017; b. The estimated annual percentage change of endometrial cancer age-standardized YLDs rate from 1990 to 2017).


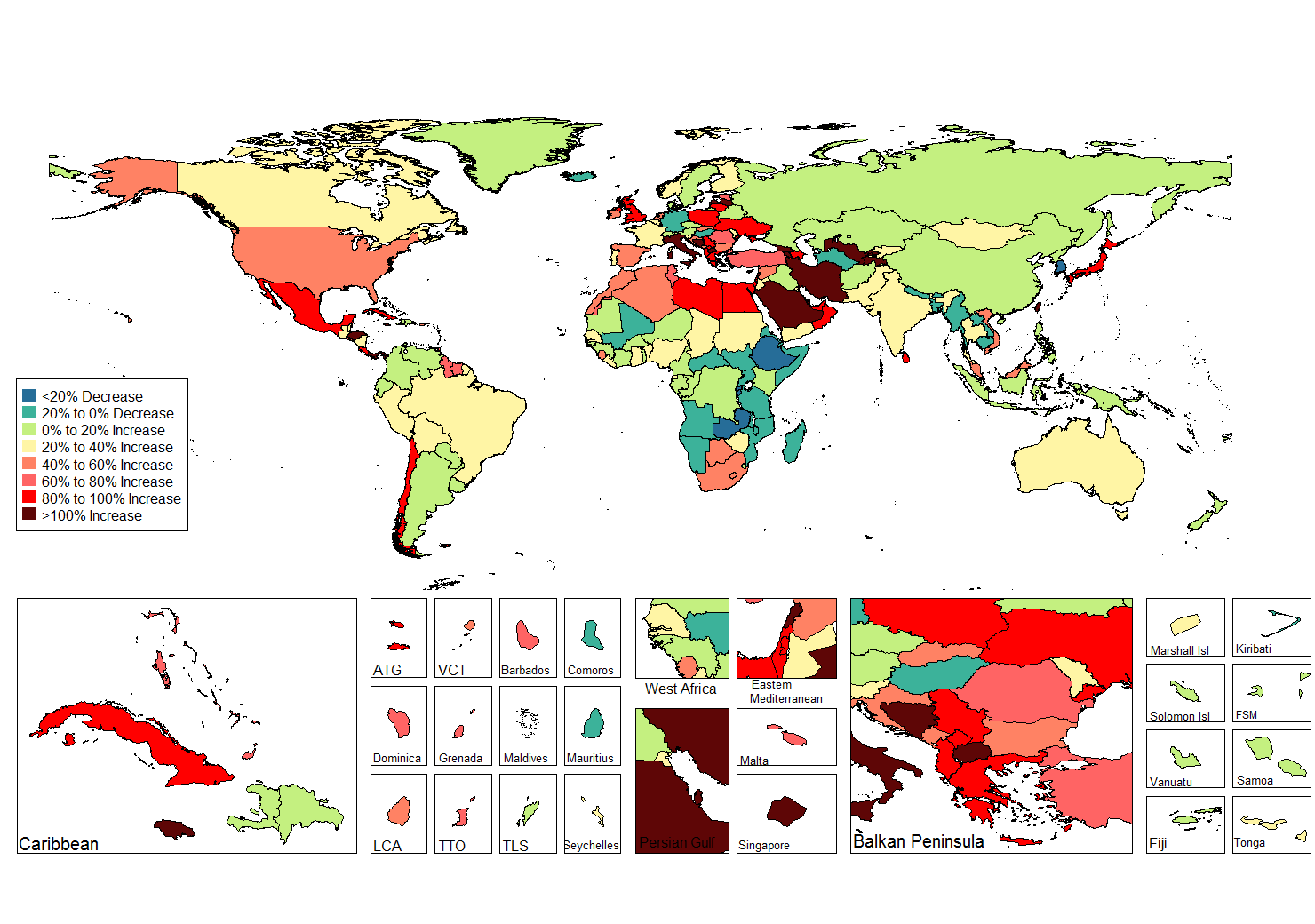


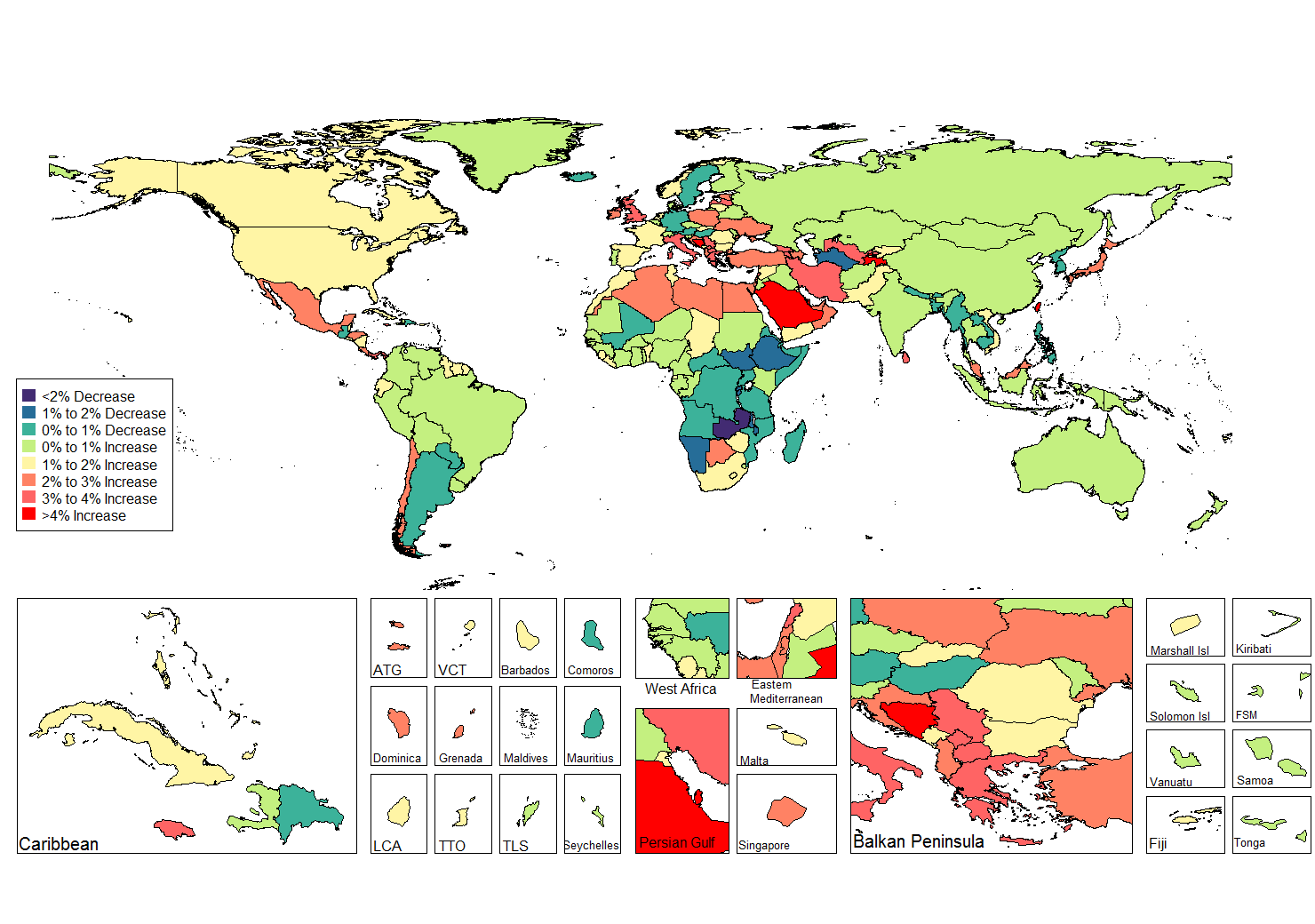


a: years lived with disability

# Supplemental Figure 16. Trend of global YLDs^a^ of endometrial cancer by age group, 1990−2017





a: years lived with disability

Supplemental Figure 17. Trends in the global disease burden of endometrial cancer YLLs^a^ from 1990 to 2017. (a. Trends in the global disease burden of endometrial cancer YLLs by sociodemographic index from 1990 to 2017; b. Trends in the global disease burden of endometrial cancer YLLs by region from 1990 to 2017).





a: years of life lost

Supplemental Figure 18. The global disease burden of endometrial cancer YLLs^a^ in 195 countries and territories. (a. The percent change in the age-standardized YLLs rate of endometrial cancer between 1990 and 2017; b. The estimated annual percentage change of endometrial cancer age-standardized YLLs rate from 1990 to 2017).


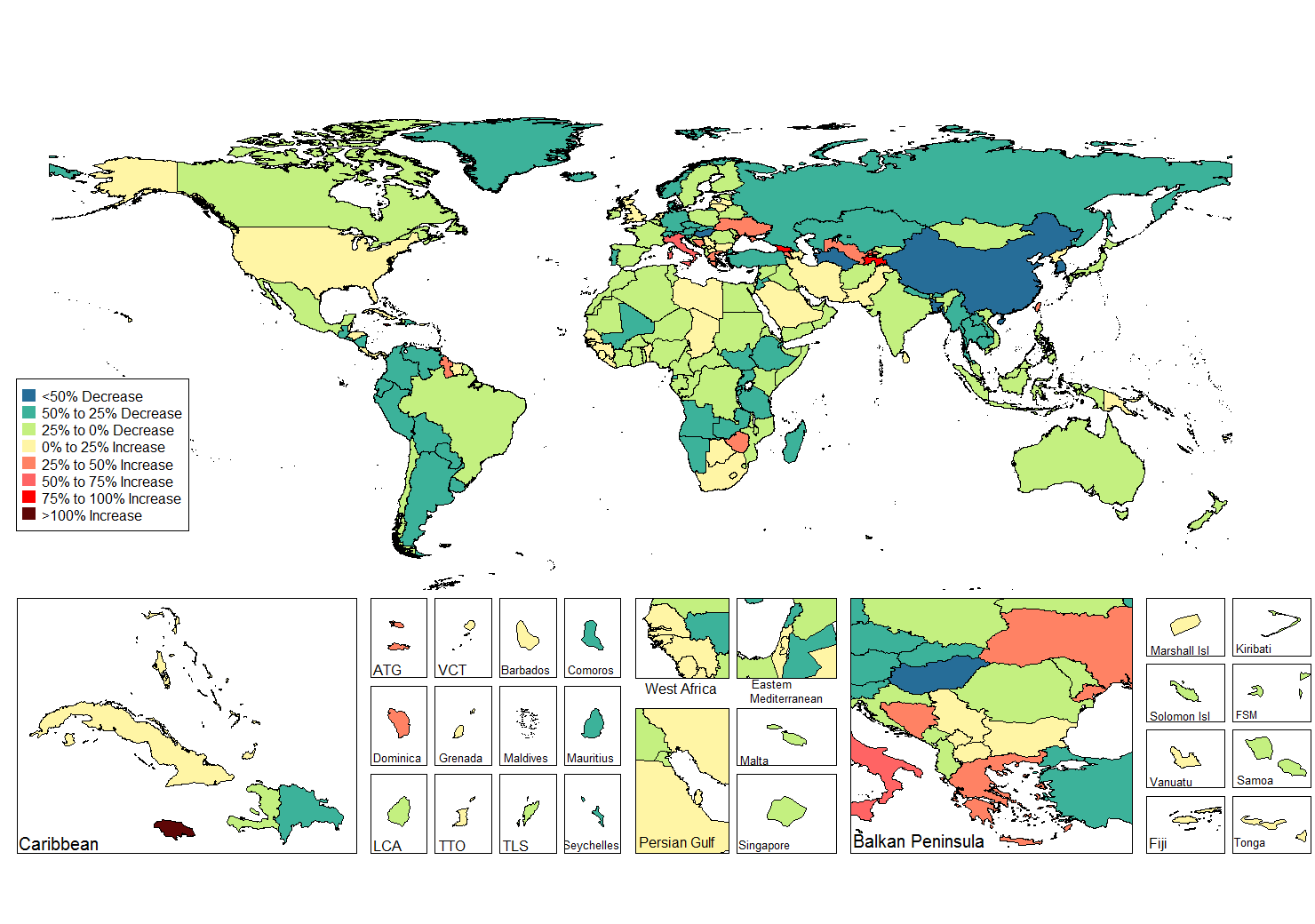


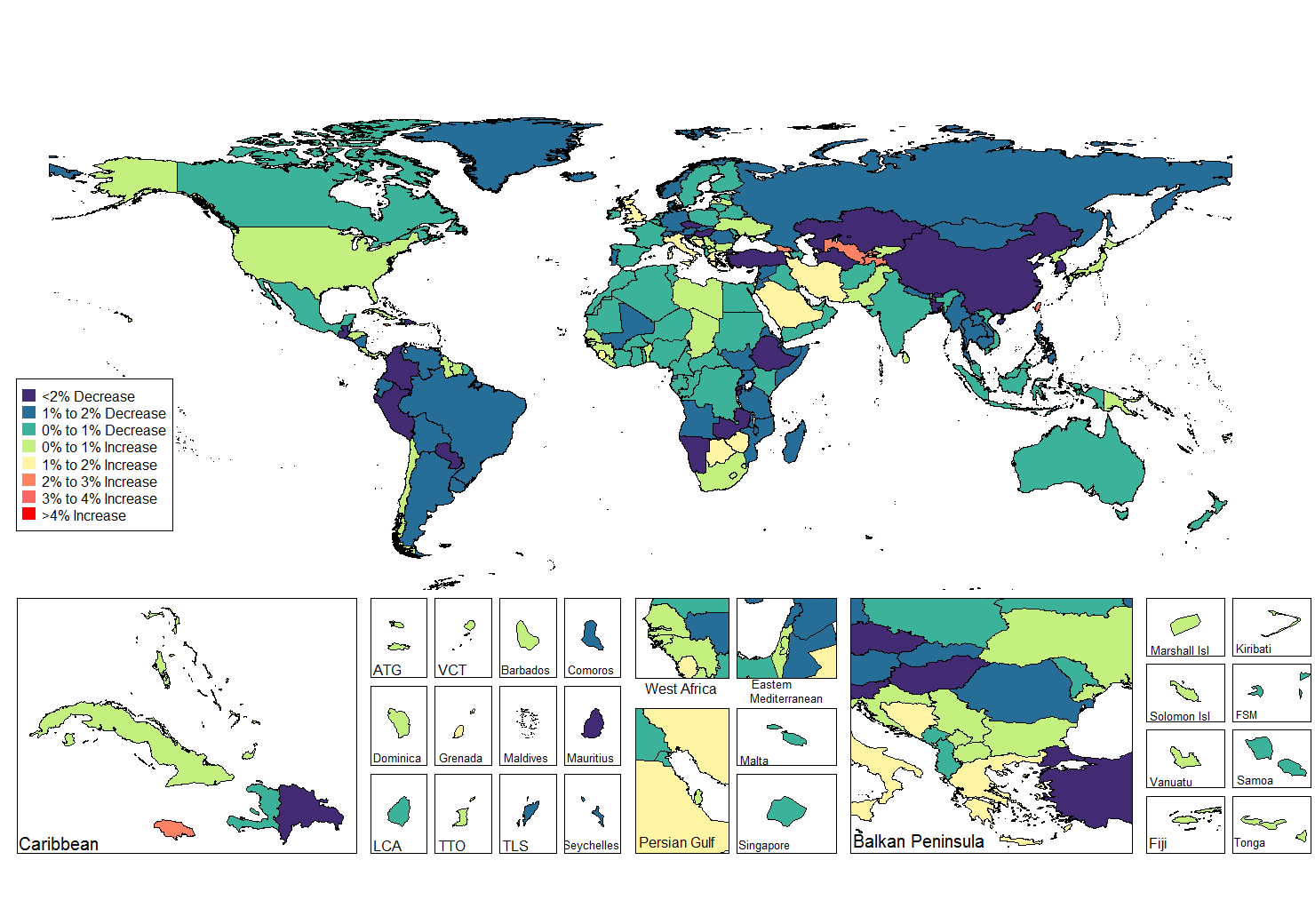


a: years of life lost

# Supplemental Figure 19. Trend of global YLLs^a^ of endometrial cancer by age group, 1990−2017





a: years of life lost

Supplemental Figure 20. Co-evolution of global age-standardized DALYs^a^ with high body-mass index and five SDI quintiles and for GBD regions for endometrial cancer, 1990−2017.



a: disability adjusted life years
